# Supplementary material for: Single cell autofluorescence imaging reveals immediate metabolic shifts of neutrophils with activation across biological systems
Source: Front Immunol. 2025 Aug 7;16:1617993. doi: 10.3389/fimmu.2025.1617993 (PMC12367685; doi:10.3389/fimmu.2025.1617993)
Supplement: Supplementary file 1 [file DataSheet1.pdf]

Supplementary Figures & Tables

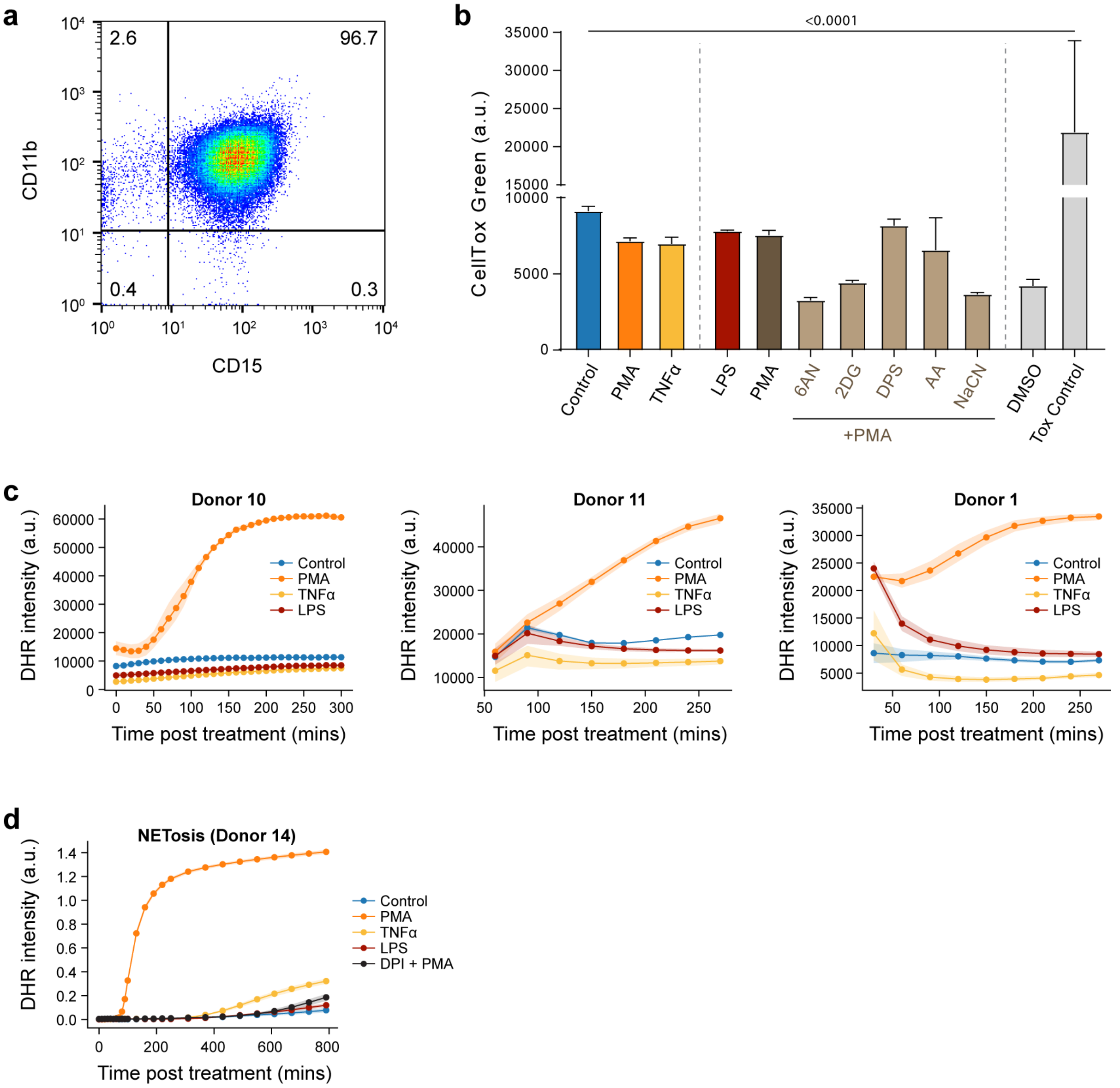

Supplementary Figure 1: **(a)** Quantification of neutrophil isolation purity by flow cytometry using labels for CD11b and CD15. **(b)** Cytotoxicity assay of neutrophils using membrane-impermeable dye Celltox green. The conditions include unstimulated control, (100nM), LPS (20µg/L) and TNFα (5µg/L) treatment for 60 mins and PMA, PMA along with inhibitors 100mM 2DG, 5mM 6AN, 10µM DPI, and 1µM AA treatment for 15 mins. **(c)** Quantification of fluorescence intensity of DHR indicating intracellular ROS in unstimulated control and PMA (100nM), TNFα (5µg/L) and LPS (20µg/L) treated neutrophils. These are repeats of from 3 distinct donors (Donor 10, 11 and 1) compared to data presented in Fig 1a. **(d)** Quantification of NET release assay. A subset of this data was presented in Fig 1b.

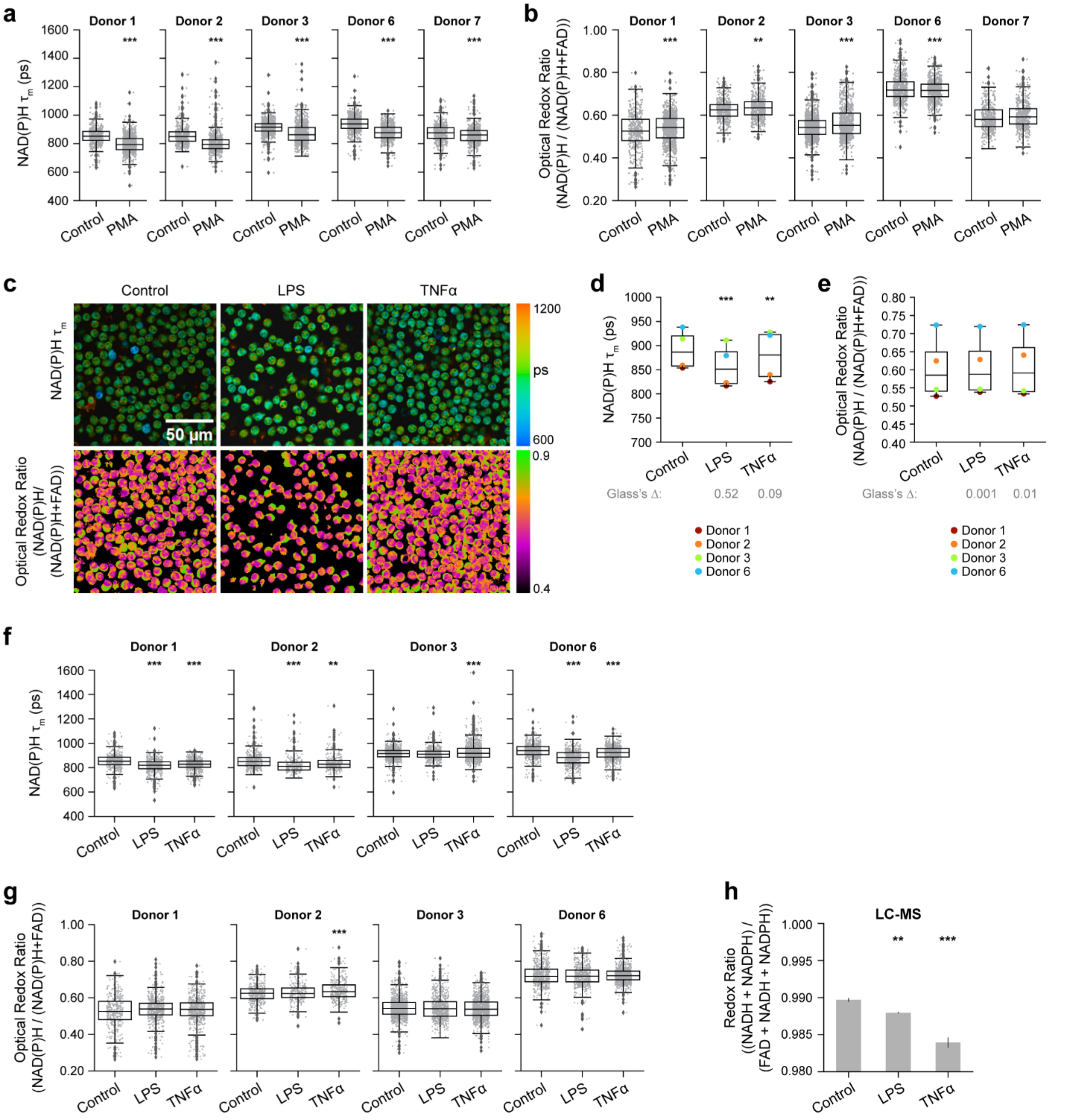

Supplementary Figure 2. Single cell quantification of **(a)** NAD(P)H mean lifetime and **(b)** optical redox ratio of control and PMA (100nM) treated neutrophils separately plotted for 5 distinct donors (Donors 1, 2, 3, 6, 7) Single cell quantification for Donors 4-5 is presented in Supp. Fig 5d-e and for Donor 8 in Supp. Fig 7a. At least 5 images were acquired per condition and each data point is a single cell: n = 400 - 900 cells/condition/donor; Table S1. Data presented in Fig 1d and 1e are derived from (a) and (b) respectively. **(c)** Representative images of NAD(P)H mean lifetime and optical redox ratio of control, LPS (20µg/L for 60 mins) and TNFα (5µg/L for 60 mins) treated neutrophils. Donor-level average of **(d)** NAD(P)H mean lifetime and **(e)** optical redox ratio of control, LPS and TNFα treated neutrophils from 4 distinct donors (Donors 1, 2, 3, and 6). Each point represents the average value for a single donor (n=400 - 900 cells/condition/donor, Table

S1). Corresponding single cell quantification of **(f)** NAD(P)H mean lifetime and **(g)** optical redox ratio of control and LPS and TNFα treated neutrophils plotted separately for the 4 distinct donors where each point is a single cell. Data presented in (d) and (e) are derived from (f) and (g) respectively. **(h)** Redox ratio computed from molar concentration measured by LC-MS from 2 technical replicates for indicated conditions (Donor 8). The control from this data was presented in Fig 1f. The control data for Donor 1-3, and 6 in (d-g) are also presented in (a-b) and Fig 1d-e. Significance was determined for (a-b) using Student's T test and (d-h) using ANOVA with *post hoc* Tukey's test (*** $p < 0.001$; ** $p < 0.01$; * $p < 0.05$). Error bars represent the 95% confidence interval. Glass's Deltas for (d-e) were calculated with respect to control cells as the effect size.

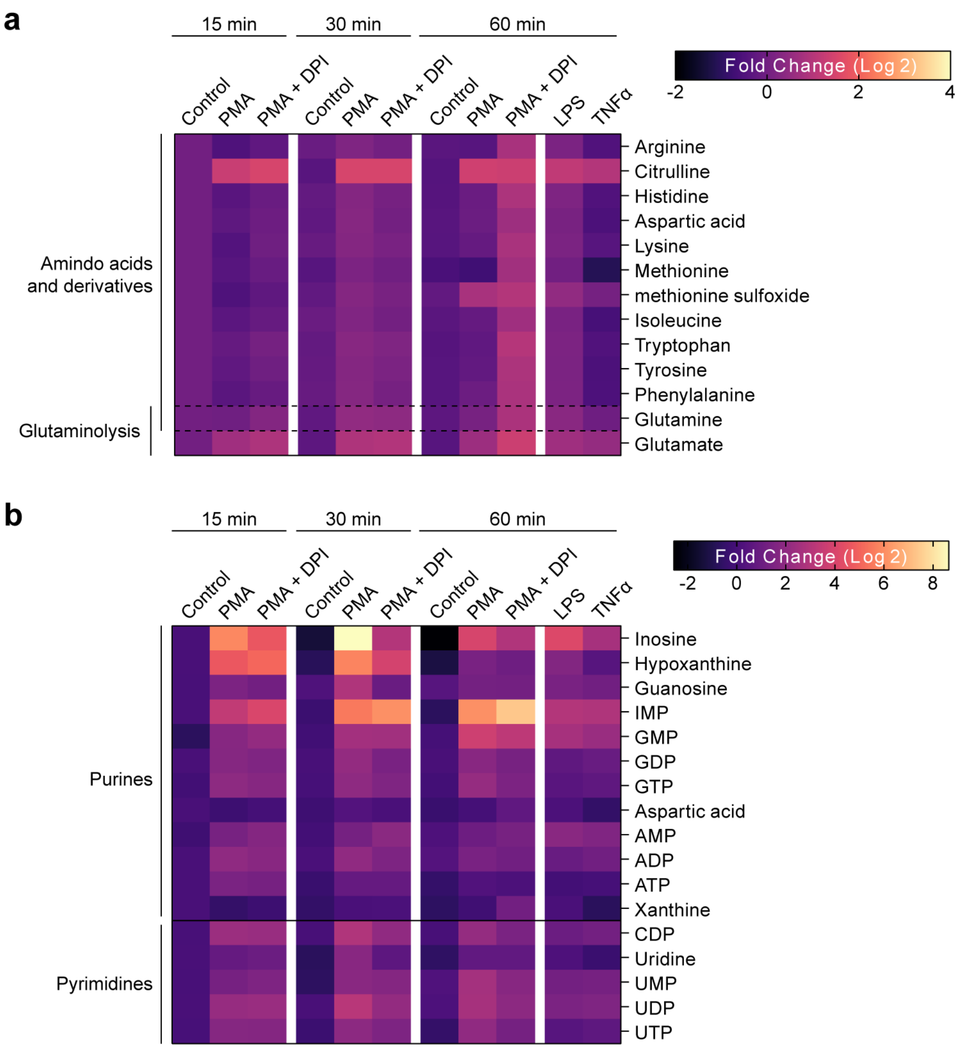

Supplementary Figure 3: Heat map representation of metabolomic variations across the unstimulated control, PMA treatment (100nM) and PMA together with NOX2 inhibitor DPI (10μM) treatment (PMA+DPI). Heatmap also includes stimulation with LPS (20μg/L) and TNFα (5μg/L). To align with the OMI conditions, metabolites were extracted at 15, 30 and 60 mins for all the conditions except for LPS and TNFα, which were extracted at 60 mins. Each metabolite abundance is normalized to the control abundance at 15 mins then log base 2 transformed (Log2

(measured Abundance/Control Abundance)) for each experimental condition. Metabolites have been grouped based on (a) amino acids and derivatives and glutaminolysis, and (b) nucleotides. Significance was determined using one-way analysis of variance (ANOVA) with *post hoc* Dunnett's test and presented in Table S3.

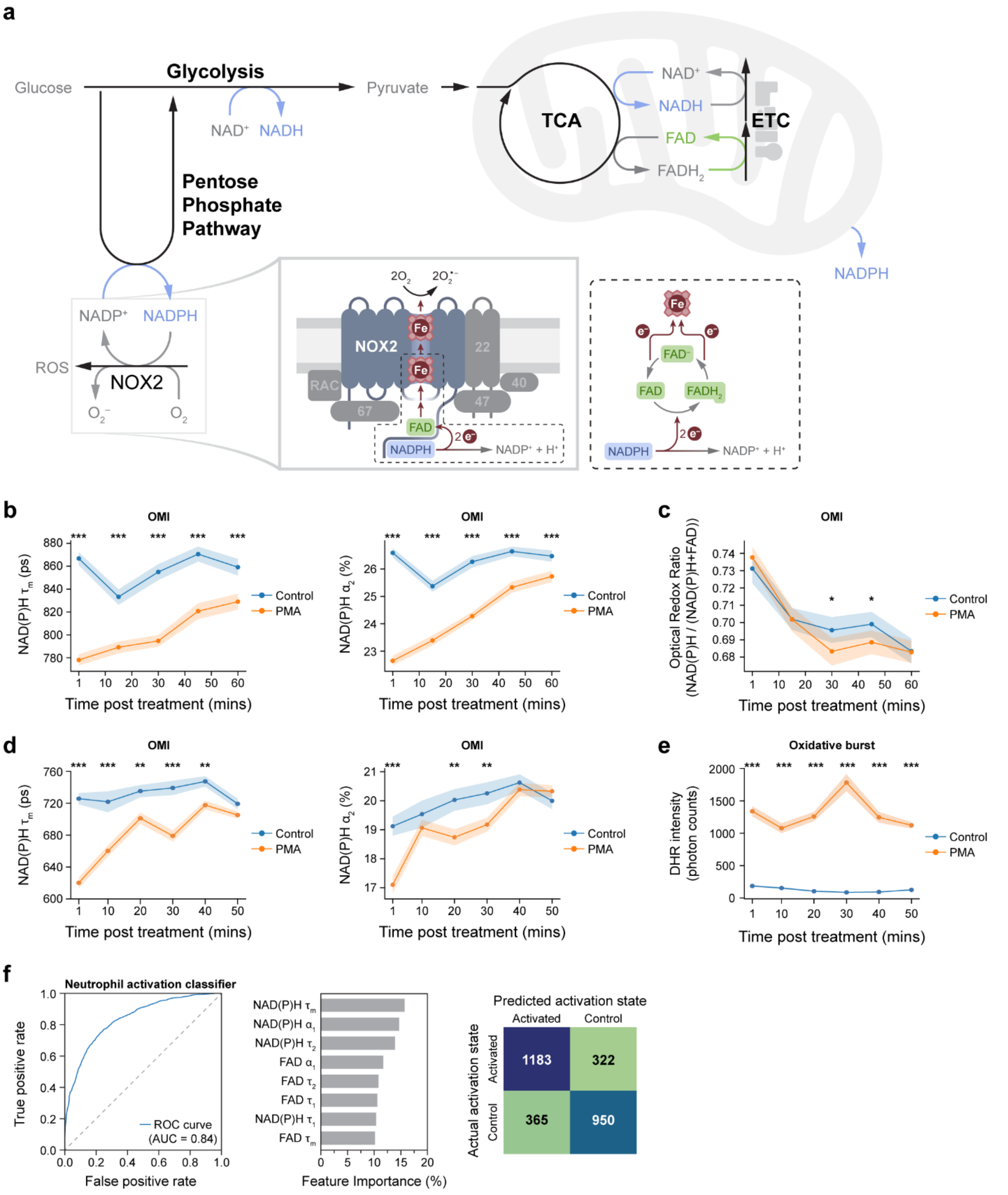

Supplementary Figure 4: (a) Schematic of major metabolic pathways. Inset shows the route of

electron transfer in the activated NOX2 enzyme complex consisting of integral transmembrane proteins NOX2, p22phox (22) and associated cytosolic proteins p40phox (40), p47phox (47), p67phox (67) and RAC (RAC1 or RAC 2). NADPH in the cytosol donates 2 electrons which are transferred by FAD to two heme (Fe) groups and ultimately to molecular oxygen to generate superoxide ($2O_2^-$). In this process, NADPH is reduced to NADP+ while FAD cycles between FADH2 (by receiving 2 electrons) and FAD (by donating 2 electrons in a 2-step process). **(b)** Single cell quantification of NAD(P)H mean lifetime (left), bound NAD(P)H α_2 percentage (right) and **(c)** optical redox ratio of control and PMA (100nM) treated neutrophils acquired from at least 5 images per condition every 15 mins from 0 mins (after addition of PMA) to 60 mins post-treatment. Each point represents the average for all cells at the indicated timepoint, n = 200-300 cells/condition per time point, (see Table S2) and error bars represent the 95% confidence interval. This is the second repeat on neutrophils from a distinct donor (Donor 9) compared to data presented in Fig 2b-c. (d) Single cell quantification of NAD(P)H mean lifetime (left), and bound NAD(P)H α2 percentage (right) and (e) fluorescence intensity of DHR (imaged sequentially from the same field of view) indicating intracellular ROS of control and PMA (100nM) treated neutrophils acquired from at least 3 images per condition every 10 mins from 1 mins (after addition of PMA) to 50 mins post-treatment. Each point represents the average for all cells at the indicated timepoint, n = 35-75 cells/condition per time point, (see Table S2) and error bars represent the 95% confidence interval. This is the third repeat on neutrophils from a distinct donor (Donor 18) compared to data presented in (b) and Fig 2b. Significance for (b-e) was determined using Student's T test.

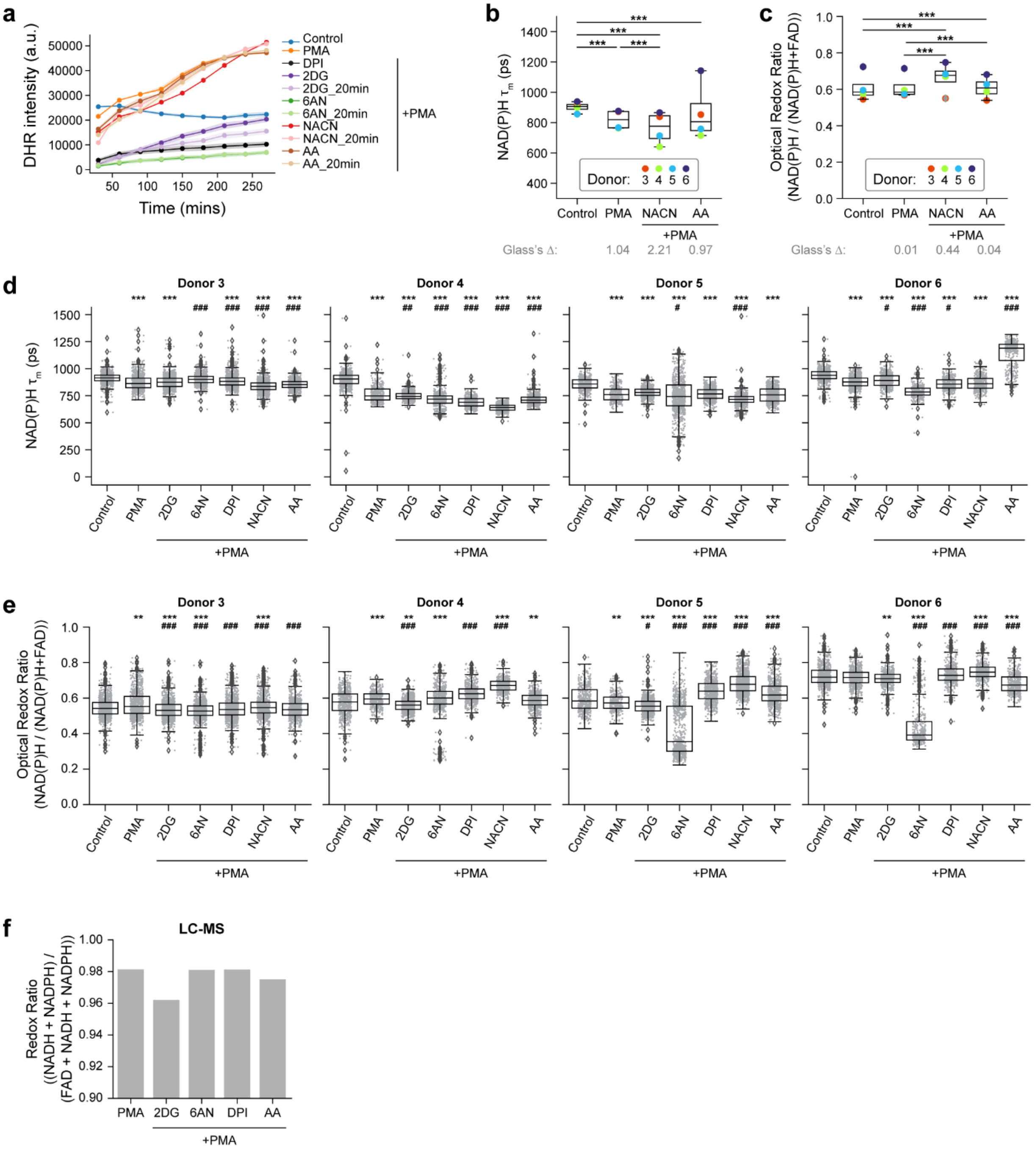

Supplementary Figure 5: (a) Quantification of fluorescence intensity of Dihydrorhodamine 123 (DHR) indicating intracellular ROS in unstimulated control, PMA treated, PMA along with 100mM 2DG, 5mM 6AN, 10µM DPI, and1µM AA treated neutrophils for 15 minutes. Inhibitor labels ending with '20' indicate pre-incubation with the inhibitor for 20 minutes before PMA treatment. A subset of conditions are repeats of Fig 3b but on neutrophils from a different donor (Donor 12). (b) NAD(P)H mean lifetime and (c) optical redox ratio of control, PMA treated, and PMA plus inhibitor (NaCN, AA) treated (15 mins) neutrophils from 4 distinct donors (Donor 3-6). Each dot represents the average across all cells per donor (n= 400-900 cells/condition/donor, Table S1). Corresponding single cell quantification of (d) NAD(P)H mean lifetime and (e) optical

redox ratio separately plotted for the 4 distinct donors (Donor 3-6) where each point is a single cell. Data presented in (b) and (c) are derived from (d) and (e) respectively. Symbols for statistical significance in d-e: * indicates comparison to control, # indicates comparison to PMA. (f) Redox ratio computed from LC-MS measurements. Statistical significance of differences between multiple conditions for data presented in (b-e) were tested using ANOVA with *post hoc* Tukey's test (***/### p< 0.001; **/## p< 0.01; */# p< 0.05). Error bars represent the 95% confidence interval.

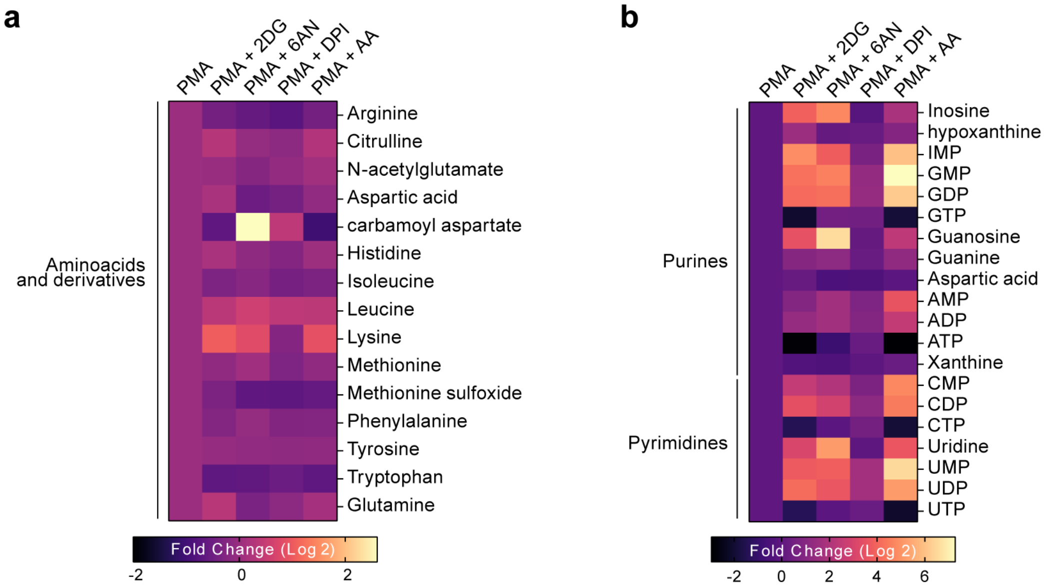

Supplementary Figure 6: Heat map representation of metabolomic variations across PMA (100nM) treatment and PMA together with 100mM 2DG (PMA+2DG), 5mM 6AN (PMA+6AN), 10µM DPI (PMA+DPI), and 1µM AA (PMA+AA) (Donor 13). To align with the OMI conditions, metabolites were extracted at 15 minutes. Each metabolite abundance is normalized to the control abundance then log base 2 transformed (Log_2 (measured Abundance/Control Abundance)) for each experimental condition. Metabolites have been grouped based on (a) amino acids and derivatives, and (b) nucleotides. Significance was determined using one-way analysis of variance (ANOVA) with *post hoc* Dunnett's test and are presented in Table S5

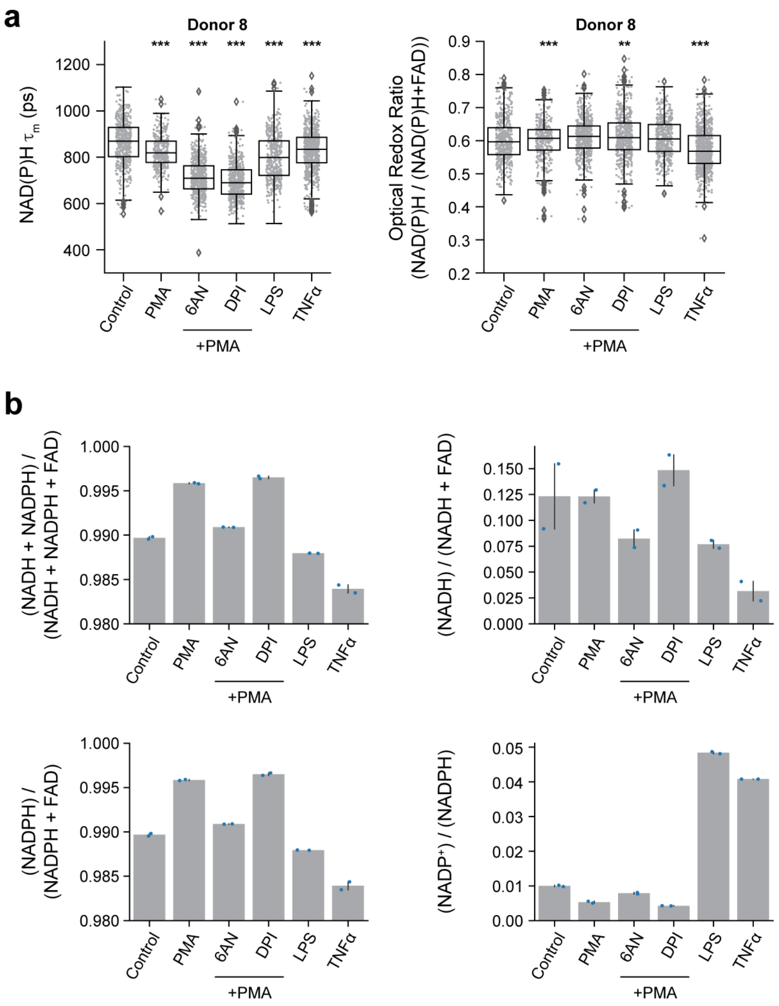

Supplementary Figure 7: Paired OMI and LC-MS measurement of cells from the same donor (Donor 8). (a) Single cell quantification of NAD(P)H mean lifetime (left) and optical redox ratio (right) of control and PMA (100nM) and PMA plus inhibitor (5mM 6AN or 10µM DPI), LPS (20µg/L) and TNFα (5µg/L) treated neutrophils where each point is a single cell. Statistical significance of differences between multiple conditions for data presented in (a) were tested using ANOVA with *post hoc* Tukey's test (*** $p< 0.001$; ** $p< 0.01$; * $p< 0.05$). Error bars represent the 95% confidence interval. (b) Redox ratios computed from LCMS measurements for the indicated conditions for two technical replicates. All data was collected 15 minutes after treatment.

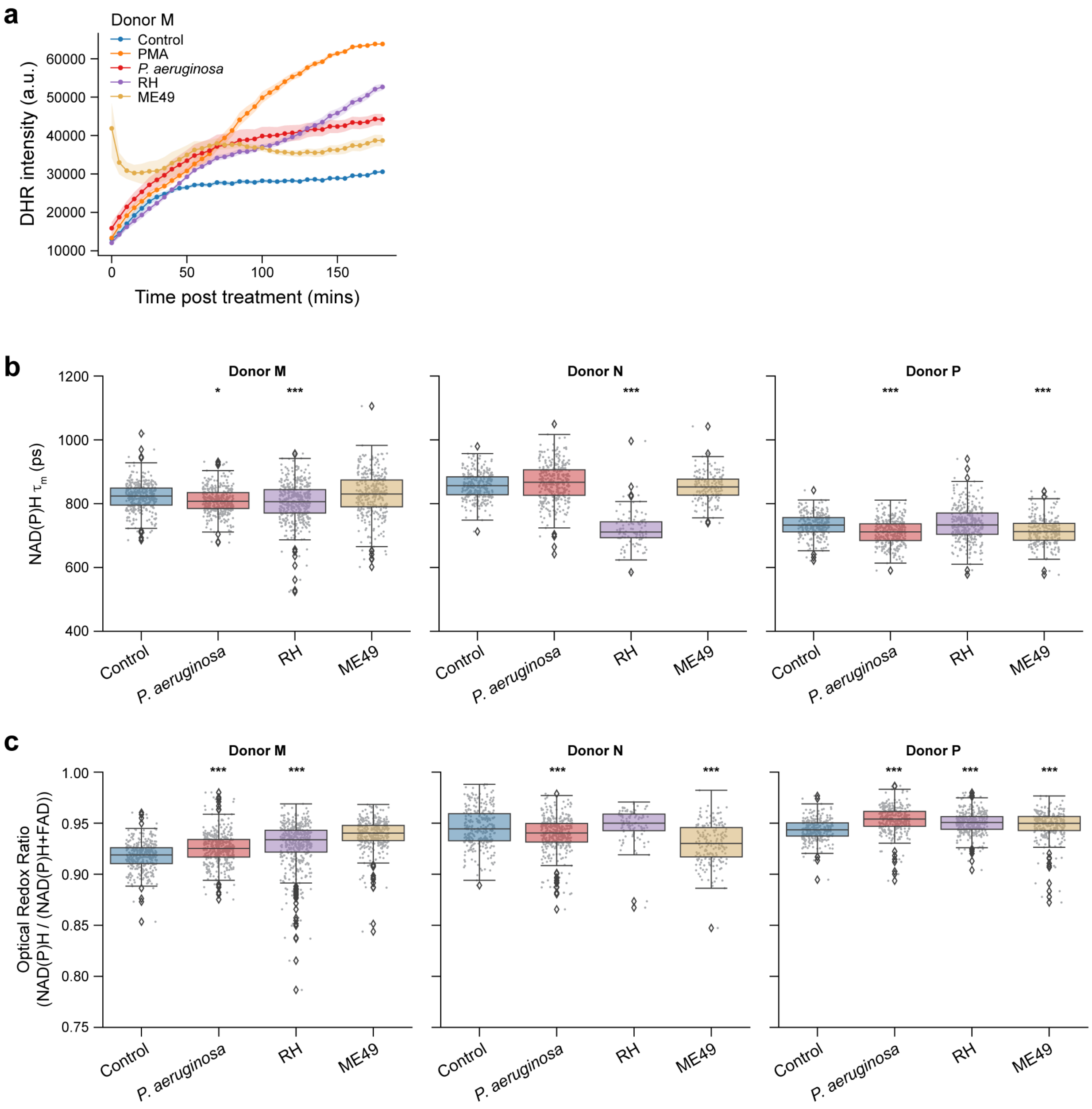

Supplementary Figure 8 (a) Quantification of fluorescence intensity of Dihydrorhodamine 123 (DHR) indicating intracellular ROS in unstimulated control, PMA, coculture with *P. aeruginosa*, and *T. gondii* strains RH and ME49. This is the 2nd repeat of the experiment with a different donor (Donor M) compared to data presented in Fig. 4a. (b) NAD(P)H mean lifetime and (c) optical redox ratio separately plotted for the 3 distinct donors (Donor M, N and P) for indicated conditions. At least 5 images were acquired per condition and each data point is a single cell. n = 100 - 300 cells/condition/donor; Table S1. Significance is tested using ANOVA with *post hoc* Tukey's test (*** $p < 0.001$; ** $p < 0.01$; * $p < 0.05$). Data presented in Figs. 4b and 4c are derived from (b) and (c) respectively. Data in (b-c) were collected 15 minutes after treatment.

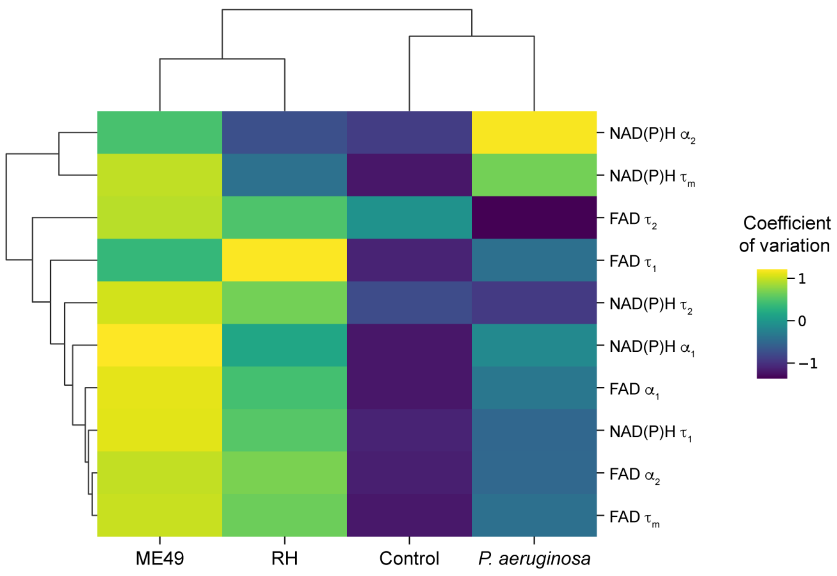

Supplementary Figure 9 Heatmap showing coefficient of variation of OMI variables (subset of variables presented in Fig. 4e) in unstimulated control, and neutrophils activated with *P. aeruginosa*, and *T. gondii* strains RH and ME49. All plots include data from 3 distinct donors (Donor M, N and P, number of cells per donor in Table S1). All data collected at 15 minutes after infection.

Table S1. Cells analyzed per donor per condition

Donor #	Condition	n = cells analyzed	Donor #	Condition	n = cells
Donor 1	Control	402	Donor 6	Control	628
	PMA	938		PMA	649
	LPS	562		LPS	577
	TNFα	635		TNFα	842
Donor 2	Control	429		PMA +2DG	567
	PMA	538		PMA + 6AN	341
	LPS	240		PMA + DPI	391
	TNFα	320		PMA + NaCN	383
Donor 3	Control	985		PMA + AA	407
	PMA	801		PMA + IAA	441
	LPS	679	Donor 7	Control	429
	TNFα	1128		PMA	457
	PMA +2DG	1065	Donor 8	Control	515
	PMA + 6AN	907		PMA	888
	PMA + DPI	999		LPS	458
	PMA + NaCN	938		TNFα	810
	PMA + AA	739		PMA + 6AN	595
	PMA + IAA	736		PMA + DPI	530
Donor 4	Control	557	Donor M	Control	343
	PMA	437		ME49	331
	PMA +2DG	282		PAO	370
	PMA + 6AN	560		RH	445
	PMA + DPI	590	Donor N	Control	282
	PMA + NaCN	481		ME49	179
	PMA + AA	624		PAO	357
	PMA + IAA	551		RH	122
Donor 5	Control	434	Donor P	Control	355
	PMA	312		ME49	247
	PMA +2DG	688		PAO	305
	PMA + 6AN	794		RH	323
	PMA + DPI	653			
	PMA + NaCN	927			
	PMA + AA	615			
	PMA + IAA	595			

Table S2. Cells analyzed per donor per condition in timeseries OMI

Donor #	Condition	Timepoint (min)	n = cells analyzed
Donor 10	Control	1	185
		15	203
		30	246
		45	236
		60	232
	PMA	1	256
		15	267
		30	281
		45	249
		60	270
Donor 9	Control	1	238
		15	332
		30	280
		45	272
		60	254
	PMA	1	261
		15	265
		30	281
		45	276
		60	261
Donor 18	Control	1	56
		10	49
		20	55
		30	49
		40	73
		50	60
	PMA	1	50
		10	35
		20	34
		30	52
		40	67
		50	66

Table S4. Variables included in UMAP and classifier

**Figure**	**Variables**
UMAP (Fig 2e)	NAD(P)H α1,<br>NAD(P)H τ1,<br>NAD(P)H τ2,<br>NAD(P)H τm,<br>FAD α1,<br>FAD τ1,<br>FAD τ2,<br>FAD τm,<br>area,<br>perimeter<br>eccentricity
Random forest Classifier (Fig2f) and Coefficient of variation heatmap (Fig 4e)	NAD(P)H α1,<br>NAD(P)H τ1,<br>NAD(P)H τ2,<br>NAD(P)H τm,<br>FAD α1,<br>FAD τ1,<br>FAD τ2,<br>FAD τm,<br>area,<br>perimeter<br>eccentricity
Random forest Classifier (Supp Fig 4f) and Coefficient of variation heatmap (Supp Fig 9)	NAD(P)H α1,<br>NAD(P)H τ1,<br>NAD(P)H τ2,<br>NAD(P)H τm,<br>FAD α1,<br>FAD τ1,<br>FAD τ2,<br>FAD τm,

Table S6. Cells analyzed across all zebrafish larvae and replicates per condition

**Fig #**	**Condition**	**n = cells analyzed**
Fig 5 d-e	Control	213
	PMA	109
Fig 5f-g	Control	162
	PMA	217
	2DG + PMA	189
Fig 5h-i	Control	237
	PMA	211
	6AN + PMA	222
