# Supplementary material for: Single cell autofluorescence imaging reveals immediate metabolic shifts of neutrophils with activation across biological systems
Source: Front Immunol. 2025 Aug 7;16:1617993. doi: 10.3389/fimmu.2025.1617993 (PMC12367685; doi:10.3389/fimmu.2025.1617993)
Supplement: Supplementary file 2 [file DataSheet2.pdf]

Table S 3. LC-MS data P values as a result of ANOVA test with *post hoc* Dunnett’s test comparing to control for each timepoint

Within each row,
compare columns (simple effects within rows)

| | |
|---|---|
| Number of families | 55 |
| Number of comparisons per family | 5 |
| Alpha | 0.05 |

| Dunnett's multiple comparisons test | Summary | Adjusted P Value |
|---|---|---|
| FAD | | |
| unstim_60m_A vs. PMA_60m_A | **** | <0.0001 |
| unstim_60m_A vs. PMA_DPI_60m_A | ns | >0.9999 |
| unstim_60m_A vs. PMA_6AN_60m_A | **** | <0.0001 |
| unstim_60m_A vs. LPS_60m_A | **** | <0.0001 |
| unstim_60m_A vs. TNFa_60m_A | **** | <0.0001 |
| NADH | | |
| unstim_60m_A vs. PMA_60m_A | ns | 0.3297 |
| unstim_60m_A vs. PMA_DPI_60m_A | **** | <0.0001 |
| unstim_60m_A vs. PMA_6AN_60m_A | **** | <0.0001 |
| unstim_60m_A vs. LPS_60m_A | **** | <0.0001 |
| unstim_60m_A vs. TNFa_60m_A | ns | >0.9999 |
| NADP+ | | |
| unstim_60m_A vs. PMA_60m_A | **** | <0.0001 |
| unstim_60m_A vs. PMA_DPI_60m_A | **** | <0.0001 |
| unstim_60m_A vs. PMA_6AN_60m_A | **** | <0.0001 |
| unstim_60m_A vs. LPS_60m_A | **** | <0.0001 |
| unstim_60m_A vs. TNFa_60m_A | **** | <0.0001 |
| NADPH | | |
| unstim_60m_A vs. PMA_60m_A | ns | 0.9179 |
| unstim_60m_A vs. PMA_DPI_60m_A | **** | <0.0001 |
| unstim_60m_A vs. PMA_6AN_60m_A | **** | <0.0001 |
| unstim_60m_A vs. LPS_60m_A | **** | <0.0001 |
| unstim_60m_A vs. TNFa_60m_A | **** | <0.0001 |
| 2-hydroxyglutarate | | |
| unstim_60m_A vs. PMA_60m_A | **** | <0.0001 |
| unstim_60m_A vs. PMA_DPI_60m_A | **** | <0.0001 |
| unstim_60m_A vs. PMA_6AN_60m_A | **** | <0.0001 |
| unstim_60m_A vs. LPS_60m_A | **** | <0.0001 |
| unstim_60m_A vs. TNFa_60m_A | **** | <0.0001 |

3PG

| | | |
|---|---|---|
| unstim_60m_A vs. PMA_60m_A | **** | <0.0001 |
| unstim_60m_A vs. PMA_DPI_60m_A | **** | <0.0001 |
| unstim_60m_A vs. PMA_6AN_60m_A | **** | <0.0001 |
| unstim_60m_A vs. LPS_60m_A | **** | <0.0001 |
| unstim_60m_A vs. TNFa_60m_A | **** | <0.0001 |

6-phosphogluconate

| | | |
|---|---|---|
| unstim_60m_A vs. PMA_60m_A | **** | <0.0001 |
| unstim_60m_A vs. PMA_DPI_60m_A | **** | <0.0001 |
| unstim_60m_A vs. PMA_6AN_60m_A | **** | <0.0001 |
| unstim_60m_A vs. LPS_60m_A | **** | <0.0001 |
| unstim_60m_A vs. TNFa_60m_A | **** | <0.0001 |

Acetyl Coenzyme A

| | | |
|---|---|---|
| unstim_60m_A vs. PMA_60m_A | **** | <0.0001 |
| unstim_60m_A vs. PMA_DPI_60m_A | **** | <0.0001 |
| unstim_60m_A vs. PMA_6AN_60m_A | **** | <0.0001 |
| unstim_60m_A vs. LPS_60m_A | **** | <0.0001 |
| unstim_60m_A vs. TNFa_60m_A | **** | <0.0001 |

ADP

| | | |
|---|---|---|
| unstim_60m_A vs. PMA_60m_A | **** | <0.0001 |
| unstim_60m_A vs. PMA_DPI_60m_A | **** | <0.0001 |
| unstim_60m_A vs. PMA_6AN_60m_A | **** | <0.0001 |
| unstim_60m_A vs. LPS_60m_A | ** | 0.0072 |
| unstim_60m_A vs. TNFa_60m_A | **** | <0.0001 |

ADP-D-glucose

| | | |
|---|---|---|
| unstim_60m_A vs. PMA_60m_A | **** | <0.0001 |
| unstim_60m_A vs. PMA_DPI_60m_A | **** | <0.0001 |
| unstim_60m_A vs. PMA_6AN_60m_A | **** | <0.0001 |
| unstim_60m_A vs. LPS_60m_A | **** | <0.0001 |
| unstim_60m_A vs. TNFa_60m_A | **** | <0.0001 |

alpha-ketoglutarate

| | | |
|---|---|---|
| unstim_60m_A vs. PMA_60m_A | **** | <0.0001 |
| unstim_60m_A vs. PMA_DPI_60m_A | **** | <0.0001 |
| unstim_60m_A vs. PMA_6AN_60m_A | **** | <0.0001 |
| unstim_60m_A vs. LPS_60m_A | **** | <0.0001 |
| unstim_60m_A vs. TNFa_60m_A | **** | <0.0001 |

AMP

| | | |
|---|---|---|
| unstim_60m_A vs. PMA_60m_A | **** | <0.0001 |
| unstim_60m_A vs. PMA_DPI_60m_A | **** | <0.0001 |
| unstim_60m_A vs. PMA_6AN_60m_A | **** | <0.0001 |
| unstim_60m_A vs. LPS_60m_A | **** | <0.0001 |
| unstim_60m_A vs. TNFa_60m_A | **** | <0.0001 |

Arginine

| | | |
|---|---|---|
| unstim_60m_A vs. PMA_60m_A | ns | 0.999 |
| unstim_60m_A vs. PMA_DPI_60m_A | **** | <0.0001 |
| unstim_60m_A vs. PMA_6AN_60m_A | ns | 0.2332 |
| unstim_60m_A vs. LPS_60m_A | * | 0.0269 |
| unstim_60m_A vs. TNFa_60m_A | ns | 0.8786 |

Aspartic acid

| | | |
|---|---|---|
| unstim_60m_A vs. PMA_60m_A | ns | 0.3491 |
| unstim_60m_A vs. PMA_DPI_60m_A | **** | <0.0001 |
| unstim_60m_A vs. PMA_6AN_60m_A | ns | 0.7996 |
| unstim_60m_A vs. LPS_60m_A | * | 0.0303 |
| unstim_60m_A vs. TNFa_60m_A | ns | 0.8069 |

ATP

| | | |
|---|---|---|
| unstim_60m_A vs. PMA_60m_A | *** | 0.0002 |
| unstim_60m_A vs. PMA_DPI_60m_A | ** | 0.003 |
| unstim_60m_A vs. PMA_6AN_60m_A | ns | 0.0686 |
| unstim_60m_A vs. LPS_60m_A | ns | 0.1301 |
| unstim_60m_A vs. TNFa_60m_A | * | 0.0349 |

CDP

| | | |
|---|---|---|
| unstim_60m_A vs. PMA_60m_A | **** | <0.0001 |
| unstim_60m_A vs. PMA_DPI_60m_A | **** | <0.0001 |
| unstim_60m_A vs. PMA_6AN_60m_A | **** | <0.0001 |
| unstim_60m_A vs. LPS_60m_A | **** | <0.0001 |
| unstim_60m_A vs. TNFa_60m_A | **** | <0.0001 |

Citrate

| | | |
|---|---|---|
| unstim_60m_A vs. PMA_60m_A | **** | <0.0001 |
| unstim_60m_A vs. PMA_DPI_60m_A | ** | 0.0036 |
| unstim_60m_A vs. PMA_6AN_60m_A | ns | 0.3628 |
| unstim_60m_A vs. LPS_60m_A | ** | 0.0082 |
| unstim_60m_A vs. TNFa_60m_A | ** | 0.0022 |

Citrulline

| | | |
|---|---|---|
| unstim_60m_A vs. PMA_60m_A | **** | <0.0001 |
| unstim_60m_A vs. PMA_DPI_60m_A | **** | <0.0001 |

| | | |
|---|---|---|
| unstim_60m_A vs. PMA_6AN_60m_A | **** | <0.0001 |
| unstim_60m_A vs. LPS_60m_A | **** | <0.0001 |
| unstim_60m_A vs. TNFa_60m_A | **** | <0.0001 |
| | | |
| Dihydroxyacetone phosphate | | |
| unstim_60m_A vs. PMA_60m_A | **** | <0.0001 |
| unstim_60m_A vs. PMA_DPI_60m_A | **** | <0.0001 |
| unstim_60m_A vs. PMA_6AN_60m_A | **** | <0.0001 |
| unstim_60m_A vs. LPS_60m_A | **** | <0.0001 |
| unstim_60m_A vs. TNFa_60m_A | **** | <0.0001 |
| | | |
| Fructose 1_6-bisphosphate | | |
| unstim_60m_A vs. PMA_60m_A | **** | <0.0001 |
| unstim_60m_A vs. PMA_DPI_60m_A | **** | <0.0001 |
| unstim_60m_A vs. PMA_6AN_60m_A | **** | <0.0001 |
| unstim_60m_A vs. LPS_60m_A | **** | <0.0001 |
| unstim_60m_A vs. TNFa_60m_A | **** | <0.0001 |
| | | |
| GDP | | |
| unstim_60m_A vs. PMA_60m_A | **** | <0.0001 |
| unstim_60m_A vs. PMA_DPI_60m_A | **** | <0.0001 |
| unstim_60m_A vs. PMA_6AN_60m_A | **** | <0.0001 |
| unstim_60m_A vs. LPS_60m_A | ** | 0.0026 |
| unstim_60m_A vs. TNFa_60m_A | **** | <0.0001 |
| | | |
| Gluconate | | |
| unstim_60m_A vs. PMA_60m_A | **** | <0.0001 |
| unstim_60m_A vs. PMA_DPI_60m_A | **** | <0.0001 |
| unstim_60m_A vs. PMA_6AN_60m_A | **** | <0.0001 |
| unstim_60m_A vs. LPS_60m_A | **** | <0.0001 |
| unstim_60m_A vs. TNFa_60m_A | **** | <0.0001 |
| | | |
| Glucose-6-phosphate | | |
| unstim_60m_A vs. PMA_60m_A | **** | <0.0001 |
| unstim_60m_A vs. PMA_DPI_60m_A | **** | <0.0001 |
| unstim_60m_A vs. PMA_6AN_60m_A | **** | <0.0001 |
| unstim_60m_A vs. LPS_60m_A | **** | <0.0001 |
| unstim_60m_A vs. TNFa_60m_A | **** | <0.0001 |
| | | |
| Glutamate | | |
| unstim_60m_A vs. PMA_60m_A | **** | <0.0001 |
| unstim_60m_A vs. PMA_DPI_60m_A | **** | <0.0001 |
| unstim_60m_A vs. PMA_6AN_60m_A | **** | <0.0001 |
| unstim_60m_A vs. LPS_60m_A | **** | <0.0001 |

| | | |
|---|---|---|
| unstim_60m_A vs. TNFa_60m_A | **** | <0.0001 |
| | | |
| Glutamine | | |
| unstim_60m_A vs. PMA_60m_A | ns | 0.1195 |
| unstim_60m_A vs. PMA_DPI_60m_A | **** | <0.0001 |
| unstim_60m_A vs. PMA_6AN_60m_A | *** | 0.0007 |
| unstim_60m_A vs. LPS_60m_A | ** | 0.0013 |
| unstim_60m_A vs. TNFa_60m_A | ns | 0.4949 |
| | | |
| Glutathione disulfide | | |
| unstim_60m_A vs. PMA_60m_A | **** | <0.0001 |
| unstim_60m_A vs. PMA_DPI_60m_A | **** | <0.0001 |
| unstim_60m_A vs. PMA_6AN_60m_A | **** | <0.0001 |
| unstim_60m_A vs. LPS_60m_A | **** | <0.0001 |
| unstim_60m_A vs. TNFa_60m_A | **** | <0.0001 |
| | | |
| Glutatione (reduced) | | |
| unstim_60m_A vs. PMA_60m_A | **** | <0.0001 |
| unstim_60m_A vs. PMA_DPI_60m_A | **** | <0.0001 |
| unstim_60m_A vs. PMA_6AN_60m_A | **** | <0.0001 |
| unstim_60m_A vs. LPS_60m_A | **** | <0.0001 |
| unstim_60m_A vs. TNFa_60m_A | **** | <0.0001 |
| | | |
| Glycerol-3-phosphate | | |
| unstim_60m_A vs. PMA_60m_A | ns | >0.9999 |
| unstim_60m_A vs. PMA_DPI_60m_A | *** | 0.0002 |
| unstim_60m_A vs. PMA_6AN_60m_A | ns | 0.082 |
| unstim_60m_A vs. LPS_60m_A | ns | 0.589 |
| unstim_60m_A vs. TNFa_60m_A | ** | 0.0037 |
| | | |
| GMP | | |
| unstim_60m_A vs. PMA_60m_A | **** | <0.0001 |
| unstim_60m_A vs. PMA_DPI_60m_A | **** | <0.0001 |
| unstim_60m_A vs. PMA_6AN_60m_A | **** | <0.0001 |
| unstim_60m_A vs. LPS_60m_A | **** | <0.0001 |
| unstim_60m_A vs. TNFa_60m_A | **** | <0.0001 |
| | | |
| GTP | | |
| unstim_60m_A vs. PMA_60m_A | **** | <0.0001 |
| unstim_60m_A vs. PMA_DPI_60m_A | **** | <0.0001 |
| unstim_60m_A vs. PMA_6AN_60m_A | **** | <0.0001 |
| unstim_60m_A vs. LPS_60m_A | ** | 0.0039 |
| unstim_60m_A vs. TNFa_60m_A | **** | <0.0001 |

Guanosine

| | | |
|---|---|---|
| unstim_60m_A vs. PMA_60m_A | **** | <0.0001 |
| unstim_60m_A vs. PMA_DPI_60m_A | **** | <0.0001 |
| unstim_60m_A vs. PMA_6AN_60m_A | **** | <0.0001 |
| unstim_60m_A vs. LPS_60m_A | **** | <0.0001 |
| unstim_60m_A vs. TNFa_60m_A | *** | 0.0003 |

Histidine

| | | |
|---|---|---|
| unstim_60m_A vs. PMA_60m_A | ns | 0.3214 |
| unstim_60m_A vs. PMA_DPI_60m_A | **** | <0.0001 |
| unstim_60m_A vs. PMA_6AN_60m_A | ns | 0.074 |
| unstim_60m_A vs. LPS_60m_A | ** | 0.0033 |
| unstim_60m_A vs. TNFa_60m_A | ns | 0.9932 |

hypoxanthine

| | | |
|---|---|---|
| unstim_60m_A vs. PMA_60m_A | **** | <0.0001 |
| unstim_60m_A vs. PMA_DPI_60m_A | **** | <0.0001 |
| unstim_60m_A vs. PMA_6AN_60m_A | **** | <0.0001 |
| unstim_60m_A vs. LPS_60m_A | **** | <0.0001 |
| unstim_60m_A vs. TNFa_60m_A | **** | <0.0001 |

inosine

| | | |
|---|---|---|
| unstim_60m_A vs. PMA_60m_A | **** | <0.0001 |
| unstim_60m_A vs. PMA_DPI_60m_A | **** | <0.0001 |
| unstim_60m_A vs. PMA_6AN_60m_A | **** | <0.0001 |
| unstim_60m_A vs. LPS_60m_A | **** | <0.0001 |
| unstim_60m_A vs. TNFa_60m_A | **** | <0.0001 |

Inosine monophosphate

| | | |
|---|---|---|
| unstim_60m_A vs. PMA_60m_A | **** | <0.0001 |
| unstim_60m_A vs. PMA_DPI_60m_A | **** | <0.0001 |
| unstim_60m_A vs. PMA_6AN_60m_A | **** | <0.0001 |
| unstim_60m_A vs. LPS_60m_A | **** | <0.0001 |
| unstim_60m_A vs. TNFa_60m_A | **** | <0.0001 |

Isoleucine

| | | |
|---|---|---|
| unstim_60m_A vs. PMA_60m_A | ns | 0.8718 |
| unstim_60m_A vs. PMA_DPI_60m_A | **** | <0.0001 |
| unstim_60m_A vs. PMA_6AN_60m_A | ns | 0.1509 |
| unstim_60m_A vs. LPS_60m_A | * | 0.0347 |
| unstim_60m_A vs. TNFa_60m_A | ns | 0.3402 |

itaconic acid

| | | |
|---|---|---|
| unstim_60m_A vs. PMA_60m_A | **** | <0.0001 |

| | | |
|---|---|---|
| unstim_60m_A vs. PMA_DPI_60m_A | **** | <0.0001 |
| unstim_60m_A vs. PMA_6AN_60m_A | **** | <0.0001 |
| unstim_60m_A vs. LPS_60m_A | **** | <0.0001 |
| unstim_60m_A vs. TNFa_60m_A | **** | <0.0001 |
| | | |
| Lactate | | |
| unstim_60m_A vs. PMA_60m_A | **** | <0.0001 |
| unstim_60m_A vs. PMA_DPI_60m_A | **** | <0.0001 |
| unstim_60m_A vs. PMA_6AN_60m_A | **** | <0.0001 |
| unstim_60m_A vs. LPS_60m_A | **** | <0.0001 |
| unstim_60m_A vs. TNFa_60m_A | **** | <0.0001 |
| | | |
| Lysine | | |
| unstim_60m_A vs. PMA_60m_A | ns | 0.6558 |
| unstim_60m_A vs. PMA_DPI_60m_A | **** | <0.0001 |
| unstim_60m_A vs. PMA_6AN_60m_A | * | 0.0331 |
| unstim_60m_A vs. LPS_60m_A | ** | 0.0097 |
| unstim_60m_A vs. TNFa_60m_A | ns | >0.9999 |
| | | |
| Malate | | |
| unstim_60m_A vs. PMA_60m_A | **** | <0.0001 |
| unstim_60m_A vs. PMA_DPI_60m_A | **** | <0.0001 |
| unstim_60m_A vs. PMA_6AN_60m_A | **** | <0.0001 |
| unstim_60m_A vs. LPS_60m_A | **** | <0.0001 |
| unstim_60m_A vs. TNFa_60m_A | ns | 0.195 |
| | | |
| Methionine | | |
| unstim_60m_A vs. PMA_60m_A | ns | 0.8831 |
| unstim_60m_A vs. PMA_DPI_60m_A | **** | <0.0001 |
| unstim_60m_A vs. PMA_6AN_60m_A | ns | 0.2753 |
| unstim_60m_A vs. LPS_60m_A | ** | 0.0062 |
| unstim_60m_A vs. TNFa_60m_A | * | 0.0168 |
| | | |
| methionine sulfoxide | | |
| unstim_60m_A vs. PMA_60m_A | **** | <0.0001 |
| unstim_60m_A vs. PMA_DPI_60m_A | **** | <0.0001 |
| unstim_60m_A vs. PMA_6AN_60m_A | ns | 0.0731 |
| unstim_60m_A vs. LPS_60m_A | *** | 0.0005 |
| unstim_60m_A vs. TNFa_60m_A | ns | 0.3493 |
| | | |
| Phenylalanine | | |
| unstim_60m_A vs. PMA_60m_A | ns | 0.2211 |
| unstim_60m_A vs. PMA_DPI_60m_A | **** | <0.0001 |
| unstim_60m_A vs. PMA_6AN_60m_A | ns | 0.2185 |

| | | |
|---|---|---|
| unstim_60m_A vs. LPS_60m_A | ** | 0.0064 |
| unstim_60m_A vs. TNFa_60m_A | ns | 0.8878 |

PRPP

| | | |
|---|---|---|
| unstim_60m_A vs. PMA_60m_A | **** | <0.0001 |
| unstim_60m_A vs. PMA_DPI_60m_A | **** | <0.0001 |
| unstim_60m_A vs. PMA_6AN_60m_A | **** | <0.0001 |
| unstim_60m_A vs. LPS_60m_A | **** | <0.0001 |
| unstim_60m_A vs. TNFa_60m_A | **** | <0.0001 |

Ribose 5-phosphate

| | | |
|---|---|---|
| unstim_60m_A vs. PMA_60m_A | **** | <0.0001 |
| unstim_60m_A vs. PMA_DPI_60m_A | **** | <0.0001 |
| unstim_60m_A vs. PMA_6AN_60m_A | **** | <0.0001 |
| unstim_60m_A vs. LPS_60m_A | **** | <0.0001 |
| unstim_60m_A vs. TNFa_60m_A | **** | <0.0001 |

Ribulose 5-phosphate

| | | |
|---|---|---|
| unstim_60m_A vs. PMA_60m_A | **** | <0.0001 |
| unstim_60m_A vs. PMA_DPI_60m_A | **** | <0.0001 |
| unstim_60m_A vs. PMA_6AN_60m_A | **** | <0.0001 |
| unstim_60m_A vs. LPS_60m_A | **** | <0.0001 |
| unstim_60m_A vs. TNFa_60m_A | **** | <0.0001 |

Sedheptulose 7-phosphate

| | | |
|---|---|---|
| unstim_60m_A vs. PMA_60m_A | **** | <0.0001 |
| unstim_60m_A vs. PMA_DPI_60m_A | **** | <0.0001 |
| unstim_60m_A vs. PMA_6AN_60m_A | **** | <0.0001 |
| unstim_60m_A vs. LPS_60m_A | **** | <0.0001 |
| unstim_60m_A vs. TNFa_60m_A | **** | <0.0001 |

Succinate

| | | |
|---|---|---|
| unstim_60m_A vs. PMA_60m_A | **** | <0.0001 |
| unstim_60m_A vs. PMA_DPI_60m_A | **** | <0.0001 |
| unstim_60m_A vs. PMA_6AN_60m_A | ns | 0.8236 |
| unstim_60m_A vs. LPS_60m_A | **** | <0.0001 |
| unstim_60m_A vs. TNFa_60m_A | ns | 0.1439 |

Tryptophan

| | | |
|---|---|---|
| unstim_60m_A vs. PMA_60m_A | ns | 0.7878 |
| unstim_60m_A vs. PMA_DPI_60m_A | **** | <0.0001 |
| unstim_60m_A vs. PMA_6AN_60m_A | *** | 0.0006 |
| unstim_60m_A vs. LPS_60m_A | ** | 0.0047 |
| unstim_60m_A vs. TNFa_60m_A | ns | 0.9964 |

Tyrosine

| | | |
|---|---|---|
| unstim_60m_A vs. PMA_60m_A | ns | 0.7569 |
| unstim_60m_A vs. PMA_DPI_60m_A | **** | <0.0001 |
| unstim_60m_A vs. PMA_6AN_60m_A | ns | 0.1485 |
| unstim_60m_A vs. LPS_60m_A | ** | 0.0088 |
| unstim_60m_A vs. TNFa_60m_A | ns | 0.8743 |

UDP

| | | |
|---|---|---|
| unstim_60m_A vs. PMA_60m_A | **** | <0.0001 |
| unstim_60m_A vs. PMA_DPI_60m_A | **** | <0.0001 |
| unstim_60m_A vs. PMA_6AN_60m_A | **** | <0.0001 |
| unstim_60m_A vs. LPS_60m_A | **** | <0.0001 |
| unstim_60m_A vs. TNFa_60m_A | **** | <0.0001 |

UMP

| | | |
|---|---|---|
| unstim_60m_A vs. PMA_60m_A | **** | <0.0001 |
| unstim_60m_A vs. PMA_DPI_60m_A | **** | <0.0001 |
| unstim_60m_A vs. PMA_6AN_60m_A | **** | <0.0001 |
| unstim_60m_A vs. LPS_60m_A | **** | <0.0001 |
| unstim_60m_A vs. TNFa_60m_A | **** | <0.0001 |

Uridine

| | | |
|---|---|---|
| unstim_60m_A vs. PMA_60m_A | **** | <0.0001 |
| unstim_60m_A vs. PMA_DPI_60m_A | **** | <0.0001 |
| unstim_60m_A vs. PMA_6AN_60m_A | **** | <0.0001 |
| unstim_60m_A vs. LPS_60m_A | **** | <0.0001 |
| unstim_60m_A vs. TNFa_60m_A | ns | 0.3658 |

UTP

| | | |
|---|---|---|
| unstim_60m_A vs. PMA_60m_A | **** | <0.0001 |
| unstim_60m_A vs. PMA_DPI_60m_A | **** | <0.0001 |
| unstim_60m_A vs. PMA_6AN_60m_A | **** | <0.0001 |
| unstim_60m_A vs. LPS_60m_A | **** | <0.0001 |
| unstim_60m_A vs. TNFa_60m_A | **** | <0.0001 |

xanthine

| | | |
|---|---|---|
| unstim_60m_A vs. PMA_60m_A | ns | 0.0503 |
| unstim_60m_A vs. PMA_DPI_60m_A | **** | <0.0001 |
| unstim_60m_A vs. PMA_6AN_60m_A | *** | 0.0007 |
| unstim_60m_A vs. LPS_60m_A | *** | 0.0003 |
| unstim_60m_A vs. TNFa_60m_A | ns | 0.9824 |
