# Supplementary material for: Single cell autofluorescence imaging reveals immediate metabolic shifts of neutrophils with activation across biological systems
Source: Front Immunol. 2025 Aug 7;16:1617993. doi: 10.3389/fimmu.2025.1617993 (PMC12367685; doi:10.3389/fimmu.2025.1617993)
Supplement: Supplementary file 3 [file DataSheet3.pdf]

Table S5. LC-MS data P values as a result of ANOVA test with post hoc Dunnett's test comaping to PMA for each timepoint
Compare cell means regardless of rows and columns

Number of families	1
Number of comparisons per family	7021
Alpha	0.05

Tukey's multiple comparisons test	Summary	Adjusted P Value
Inosine:PMA_15m vs. Inosine:PMA_2DG_15m	ns	0.7475
Inosine:PMA_15m vs. Inosine:PMA_6AN_15m	ns	0.1037
Inosine:PMA_15m vs. Inosine:PMA_DPI_15m	ns	>0.9999
Inosine:PMA_15m vs. Inosine:PMA_AA_15m	*	0.0409
Inosine:PMA_15m vs. Inosine:PMA_IAA_15m	*	0.0236
Inosine:PMA_15m vs. hypoxanthine:PMA_15m	ns	>0.9999
Inosine:PMA_15m vs. hypoxanthine:PMA_2DG_15m	ns	>0.9999
Inosine:PMA_15m vs. hypoxanthine:PMA_6AN_15m	ns	>0.9999
Inosine:PMA_15m vs. hypoxanthine:PMA_DPI_15m	ns	>0.9999
Inosine:PMA_15m vs. hypoxanthine:PMA_AA_15m	ns	>0.9999
Inosine:PMA_15m vs. hypoxanthine:PMA_IAA_15m	ns	0.1306
Inosine:PMA_15m vs. IMP:PMA_15m	ns	>0.9999
Inosine:PMA_15m vs. IMP:PMA_2DG_15m	ns	0.9994
Inosine:PMA_15m vs. IMP:PMA_6AN_15m	ns	0.9619
Inosine:PMA_15m vs. IMP:PMA_DPI_15m	ns	>0.9999
Inosine:PMA_15m vs. IMP:PMA_AA_15m	ns	0.9171
Inosine:PMA_15m vs. IMP:PMA_IAA_15m	ns	>0.9999
Inosine:PMA_15m vs. GMP:PMA_15m	ns	>0.9999
Inosine:PMA_15m vs. GMP:PMA_2DG_15m	ns	0.7573
Inosine:PMA_15m vs. GMP:PMA_6AN_15m	ns	0.3771
Inosine:PMA_15m vs. GMP:PMA_DPI_15m	ns	>0.9999
Inosine:PMA_15m vs. GMP:PMA_AA_15m	ns	0.2779
Inosine:PMA_15m vs. GMP:PMA_IAA_15m	ns	>0.9999
Inosine:PMA_15m vs. GDP:PMA_15m	ns	>0.9999
Inosine:PMA_15m vs. GDP:PMA_2DG_15m	ns	0.8095
Inosine:PMA_15m vs. GDP:PMA_6AN_15m	ns	0.4352
Inosine:PMA_15m vs. GDP:PMA_DPI_15m	ns	>0.9999
Inosine:PMA_15m vs. GDP:PMA_AA_15m	ns	0.3277
Inosine:PMA_15m vs. GDP:PMA_IAA_15m	ns	>0.9999
Inosine:PMA_15m vs. GTP:PMA_15m	ns	0.9374
Inosine:PMA_15m vs. GTP:PMA_2DG_15m	ns	>0.9999
Inosine:PMA_15m vs. GTP:PMA_6AN_15m	ns	>0.9999
Inosine:PMA_15m vs. GTP:PMA_DPI_15m	ns	>0.9999
Inosine:PMA_15m vs. GTP:PMA_AA_15m	ns	>0.9999
Inosine:PMA_15m vs. GTP:PMA_IAA_15m	*	0.0153
Inosine:PMA_15m vs. Guanosine:PMA_15m	ns	>0.9999
Inosine:PMA_15m vs. Guanosine:PMA_2DG_15m	ns	>0.9999
Inosine:PMA_15m vs. Guanosine:PMA_6AN_15m	ns	0.9936

Inosine:PMA_15m vs. Guanosine:PMA_DPI_15m	ns	>0.9999
Inosine:PMA_15m vs. Guanosine:PMA_AA_15m	ns	0.9801
Inosine:PMA_15m vs. Guanosine:PMA_IAA_15m	ns	>0.9999
Inosine:PMA_15m vs. Guanine:PMA_15m	ns	>0.9999
Inosine:PMA_15m vs. Guanine:PMA_2DG_15m	ns	>0.9999
Inosine:PMA_15m vs. Guanine:PMA_6AN_15m	ns	>0.9999
Inosine:PMA_15m vs. Guanine:PMA_DPI_15m	ns	>0.9999
Inosine:PMA_15m vs. Guanine:PMA_AA_15m	ns	>0.9999
Inosine:PMA_15m vs. Guanine:PMA_IAA_15m	ns	0.2507
Inosine:PMA_15m vs. Aspartic acid:PMA_15m	ns	>0.9999
Inosine:PMA_15m vs. Aspartic acid:PMA_2DG_15m	ns	>0.9999
Inosine:PMA_15m vs. Aspartic acid:PMA_6AN_15m	ns	>0.9999
Inosine:PMA_15m vs. Aspartic acid:PMA_DPI_15m	ns	>0.9999
Inosine:PMA_15m vs. Aspartic acid:PMA_AA_15m	ns	>0.9999
Inosine:PMA_15m vs. Aspartic acid:PMA_IAA_15m	ns	0.4518
Inosine:PMA_15m vs. AMP:PMA_15m	ns	>0.9999
Inosine:PMA_15m vs. AMP:PMA_2DG_15m	ns	>0.9999
Inosine:PMA_15m vs. AMP:PMA_6AN_15m	ns	>0.9999
Inosine:PMA_15m vs. AMP:PMA_DPI_15m	ns	>0.9999
Inosine:PMA_15m vs. AMP:PMA_AA_15m	ns	0.9997
Inosine:PMA_15m vs. AMP:PMA_IAA_15m	ns	>0.9999
Inosine:PMA_15m vs. ADP:PMA_15m	ns	>0.9999
Inosine:PMA_15m vs. ADP:PMA_2DG_15m	ns	>0.9999
Inosine:PMA_15m vs. ADP:PMA_6AN_15m	ns	>0.9999
Inosine:PMA_15m vs. ADP:PMA_DPI_15m	ns	>0.9999
Inosine:PMA_15m vs. ADP:PMA_AA_15m	ns	>0.9999
Inosine:PMA_15m vs. ADP:PMA_IAA_15m	ns	0.9948
Inosine:PMA_15m vs. ATP:PMA_15m	ns	0.6959
Inosine:PMA_15m vs. ATP:PMA_2DG_15m	ns	>0.9999
Inosine:PMA_15m vs. ATP:PMA_6AN_15m	ns	>0.9999
Inosine:PMA_15m vs. ATP:PMA_DPI_15m	ns	0.9989
Inosine:PMA_15m vs. ATP:PMA_AA_15m	ns	>0.9999
Inosine:PMA_15m vs. ATP:PMA_IAA_15m	**	0.0039
Inosine:PMA_15m vs. Xanthine:PMA_15m	ns	>0.9999
Inosine:PMA_15m vs. Xanthine:PMA_2DG_15m	ns	>0.9999
Inosine:PMA_15m vs. Xanthine:PMA_6AN_15m	ns	>0.9999
Inosine:PMA_15m vs. Xanthine:PMA_DPI_15m	ns	>0.9999
Inosine:PMA_15m vs. Xanthine:PMA_AA_15m	ns	>0.9999
Inosine:PMA_15m vs. CMP:PMA_15m	ns	>0.9999
Inosine:PMA_15m vs. CMP:PMA_2DG_15m	ns	>0.9999
Inosine:PMA_15m vs. CMP:PMA_6AN_15m	ns	0.9998
Inosine:PMA_15m vs. CMP:PMA_DPI_15m	ns	>0.9999
Inosine:PMA_15m vs. CMP:PMA_AA_15m	ns	0.9986
Inosine:PMA_15m vs. CMP:PMA_IAA_15m	ns	>0.9999
Inosine:PMA_15m vs. CDP:PMA_15m	ns	>0.9999
Inosine:PMA_15m vs. CDP:PMA_2DG_15m	ns	0.999
Inosine:PMA_15m vs. CDP:PMA_6AN_15m	ns	0.9517

Inosine:PMA_15m vs. CDP:PMA_DPI_15m	ns	>0.9999
Inosine:PMA_15m vs. CDP:PMA_AA_15m	ns	0.8997
Inosine:PMA_15m vs. CDP:PMA_IAA_15m	ns	>0.9999
Inosine:PMA_15m vs. CTP:PMA_15m	ns	0.9943
Inosine:PMA_15m vs. CTP:PMA_2DG_15m	ns	>0.9999
Inosine:PMA_15m vs. CTP:PMA_6AN_15m	ns	>0.9999
Inosine:PMA_15m vs. CTP:PMA_DPI_15m	ns	>0.9999
Inosine:PMA_15m vs. CTP:PMA_AA_15m	ns	>0.9999
Inosine:PMA_15m vs. CTP:PMA_IAA_15m	*	0.0431
Inosine:PMA_15m vs. Uridine:PMA_15m	ns	>0.9999
Inosine:PMA_15m vs. Uridine:PMA_2DG_15m	ns	>0.9999
Inosine:PMA_15m vs. Uridine:PMA_6AN_15m	ns	0.9973
Inosine:PMA_15m vs. Uridine:PMA_DPI_15m	ns	>0.9999
Inosine:PMA_15m vs. Uridine:PMA_AA_15m	ns	0.9901
Inosine:PMA_15m vs. Uridine:PMA_IAA_15m	ns	>0.9999
Inosine:PMA_15m vs. UMP:PMA_15m	ns	>0.9999
Inosine:PMA_15m vs. UMP:PMA_2DG_15m	ns	0.9678
Inosine:PMA_15m vs. UMP:PMA_6AN_15m	ns	0.7404
Inosine:PMA_15m vs. UMP:PMA_DPI_15m	ns	>0.9999
Inosine:PMA_15m vs. UMP:PMA_AA_15m	ns	0.6258
Inosine:PMA_15m vs. UMP:PMA_IAA_15m	ns	>0.9999
Inosine:PMA_15m vs. UDP:PMA_15m	ns	>0.9999
Inosine:PMA_15m vs. UDP:PMA_2DG_15m	ns	0.9717
Inosine:PMA_15m vs. UDP:PMA_6AN_15m	ns	0.7556
Inosine:PMA_15m vs. UDP:PMA_DPI_15m	ns	>0.9999
Inosine:PMA_15m vs. UDP:PMA_AA_15m	ns	0.6427
Inosine:PMA_15m vs. UDP:PMA_IAA_15m	ns	>0.9999
Inosine:PMA_15m vs. UTP:PMA_15m	ns	0.9675
Inosine:PMA_15m vs. UTP:PMA_2DG_15m	ns	>0.9999
Inosine:PMA_15m vs. UTP:PMA_6AN_15m	ns	>0.9999
Inosine:PMA_15m vs. UTP:PMA_DPI_15m	ns	>0.9999
Inosine:PMA_15m vs. UTP:PMA_AA_15m	ns	>0.9999
Inosine:PMA_15m vs. UTP:PMA_IAA_15m	*	0.0217
Inosine:PMA_2DG_15m vs. Inosine:PMA_6AN_15m	ns	>0.9999
Inosine:PMA_2DG_15m vs. Inosine:PMA_DPI_15m	ns	>0.9999
Inosine:PMA_2DG_15m vs. Inosine:PMA_AA_15m	ns	>0.9999
Inosine:PMA_2DG_15m vs. Inosine:PMA_IAA_15m	****	<0.0001
Inosine:PMA_2DG_15m vs. hypoxanthine:PMA_15m	ns	0.5971
Inosine:PMA_2DG_15m vs. hypoxanthine:PMA_2DG_15m	ns	>0.9999
Inosine:PMA_2DG_15m vs. hypoxanthine:PMA_6AN_15m	ns	>0.9999
Inosine:PMA_2DG_15m vs. hypoxanthine:PMA_DPI_15m	ns	0.9294
Inosine:PMA_2DG_15m vs. hypoxanthine:PMA_AA_15m	ns	>0.9999
Inosine:PMA_2DG_15m vs. hypoxanthine:PMA_IAA_15m	***	0.0005
Inosine:PMA_2DG_15m vs. IMP:PMA_15m	ns	>0.9999
Inosine:PMA_2DG_15m vs. IMP:PMA_2DG_15m	ns	>0.9999
Inosine:PMA_2DG_15m vs. IMP:PMA_6AN_15m	ns	>0.9999
Inosine:PMA_2DG_15m vs. IMP:PMA_DPI_15m	ns	>0.9999

Inosine:PMA_2DG_15m vs. IMP:PMA_AA_15m	ns	>0.9999
Inosine:PMA_2DG_15m vs. IMP:PMA_IAA_15m	ns	0.8695
Inosine:PMA_2DG_15m vs. GMP:PMA_15m	ns	>0.9999
Inosine:PMA_2DG_15m vs. GMP:PMA_2DG_15m	ns	>0.9999
Inosine:PMA_2DG_15m vs. GMP:PMA_6AN_15m	ns	>0.9999
Inosine:PMA_2DG_15m vs. GMP:PMA_DPI_15m	ns	>0.9999
Inosine:PMA_2DG_15m vs. GMP:PMA_AA_15m	ns	0.9997
Inosine:PMA_2DG_15m vs. GMP:PMA_IAA_15m	ns	>0.9999
Inosine:PMA_2DG_15m vs. GDP:PMA_15m	ns	>0.9999
Inosine:PMA_2DG_15m vs. GDP:PMA_2DG_15m	ns	>0.9999
Inosine:PMA_2DG_15m vs. GDP:PMA_6AN_15m	ns	>0.9999
Inosine:PMA_2DG_15m vs. GDP:PMA_DPI_15m	ns	>0.9999
Inosine:PMA_2DG_15m vs. GDP:PMA_AA_15m	ns	0.9999
Inosine:PMA_2DG_15m vs. GDP:PMA_IAA_15m	ns	0.9998
Inosine:PMA_2DG_15m vs. GTP:PMA_15m	ns	0.1479
Inosine:PMA_2DG_15m vs. GTP:PMA_2DG_15m	ns	0.9374
Inosine:PMA_2DG_15m vs. GTP:PMA_6AN_15m	ns	>0.9999
Inosine:PMA_2DG_15m vs. GTP:PMA_DPI_15m	ns	0.4669
Inosine:PMA_2DG_15m vs. GTP:PMA_AA_15m	ns	>0.9999
Inosine:PMA_2DG_15m vs. GTP:PMA_IAA_15m	****	<0.0001
Inosine:PMA_2DG_15m vs. Guanosine:PMA_15m	ns	>0.9999
Inosine:PMA_2DG_15m vs. Guanosine:PMA_2DG_15m	ns	>0.9999
Inosine:PMA_2DG_15m vs. Guanosine:PMA_6AN_15m	ns	>0.9999
Inosine:PMA_2DG_15m vs. Guanosine:PMA_DPI_15m	ns	>0.9999
Inosine:PMA_2DG_15m vs. Guanosine:PMA_AA_15m	ns	>0.9999
Inosine:PMA_2DG_15m vs. Guanosine:PMA_IAA_15m	ns	0.7075
Inosine:PMA_2DG_15m vs. Guanine:PMA_15m	ns	0.7955
Inosine:PMA_2DG_15m vs. Guanine:PMA_2DG_15m	ns	>0.9999
Inosine:PMA_2DG_15m vs. Guanine:PMA_6AN_15m	ns	>0.9999
Inosine:PMA_2DG_15m vs. Guanine:PMA_DPI_15m	ns	0.9856
Inosine:PMA_2DG_15m vs. Guanine:PMA_AA_15m	ns	>0.9999
Inosine:PMA_2DG_15m vs. Guanine:PMA_IAA_15m	**	0.0013
Inosine:PMA_2DG_15m vs. Aspartic acid:PMA_15m	ns	0.9393
Inosine:PMA_2DG_15m vs. Aspartic acid:PMA_2DG_15m	ns	>0.9999
Inosine:PMA_2DG_15m vs. Aspartic acid:PMA_6AN_15m	ns	>0.9999
Inosine:PMA_2DG_15m vs. Aspartic acid:PMA_DPI_15m	ns	0.999
Inosine:PMA_2DG_15m vs. Aspartic acid:PMA_AA_15m	ns	>0.9999
Inosine:PMA_2DG_15m vs. Aspartic acid:PMA_IAA_15m	**	0.0041
Inosine:PMA_2DG_15m vs. AMP:PMA_15m	ns	>0.9999
Inosine:PMA_2DG_15m vs. AMP:PMA_2DG_15m	ns	>0.9999
Inosine:PMA_2DG_15m vs. AMP:PMA_6AN_15m	ns	>0.9999
Inosine:PMA_2DG_15m vs. AMP:PMA_DPI_15m	ns	>0.9999
Inosine:PMA_2DG_15m vs. AMP:PMA_AA_15m	ns	>0.9999
Inosine:PMA_2DG_15m vs. AMP:PMA_IAA_15m	ns	0.3567
Inosine:PMA_2DG_15m vs. ADP:PMA_15m	ns	>0.9999
Inosine:PMA_2DG_15m vs. ADP:PMA_2DG_15m	ns	>0.9999
Inosine:PMA_2DG_15m vs. ADP:PMA_6AN_15m	ns	>0.9999

Inosine:PMA_2DG_15m vs. ADP:PMA_DPI_15m	ns	>0.9999
Inosine:PMA_2DG_15m vs. ADP:PMA_AA_15m	ns	>0.9999
Inosine:PMA_2DG_15m vs. ADP:PMA_IAA_15m	ns	0.1492
Inosine:PMA_2DG_15m vs. ATP:PMA_15m	ns	0.0502
Inosine:PMA_2DG_15m vs. ATP:PMA_2DG_15m	ns	0.6959
Inosine:PMA_2DG_15m vs. ATP:PMA_6AN_15m	ns	0.9982
Inosine:PMA_2DG_15m vs. ATP:PMA_DPI_15m	ns	0.2168
Inosine:PMA_2DG_15m vs. ATP:PMA_AA_15m	ns	0.9997
Inosine:PMA_2DG_15m vs. ATP:PMA_IAA_15m	****	<0.0001
Inosine:PMA_2DG_15m vs. Xanthine:PMA_15m	ns	0.7557
Inosine:PMA_2DG_15m vs. Xanthine:PMA_2DG_15m	ns	>0.9999
Inosine:PMA_2DG_15m vs. Xanthine:PMA_6AN_15m	ns	>0.9999
Inosine:PMA_2DG_15m vs. Xanthine:PMA_DPI_15m	ns	0.9753
Inosine:PMA_2DG_15m vs. Xanthine:PMA_AA_15m	ns	>0.9999
Inosine:PMA_2DG_15m vs. CMP:PMA_15m	ns	>0.9999
Inosine:PMA_2DG_15m vs. CMP:PMA_2DG_15m	ns	>0.9999
Inosine:PMA_2DG_15m vs. CMP:PMA_6AN_15m	ns	>0.9999
Inosine:PMA_2DG_15m vs. CMP:PMA_DPI_15m	ns	>0.9999
Inosine:PMA_2DG_15m vs. CMP:PMA_AA_15m	ns	>0.9999
Inosine:PMA_2DG_15m vs. CMP:PMA_IAA_15m	ns	0.4598
Inosine:PMA_2DG_15m vs. CDP:PMA_15m	ns	>0.9999
Inosine:PMA_2DG_15m vs. CDP:PMA_2DG_15m	ns	>0.9999
Inosine:PMA_2DG_15m vs. CDP:PMA_6AN_15m	ns	>0.9999
Inosine:PMA_2DG_15m vs. CDP:PMA_DPI_15m	ns	>0.9999
Inosine:PMA_2DG_15m vs. CDP:PMA_AA_15m	ns	>0.9999
Inosine:PMA_2DG_15m vs. CDP:PMA_IAA_15m	ns	0.89
Inosine:PMA_2DG_15m vs. CTP:PMA_15m	ns	0.3097
Inosine:PMA_2DG_15m vs. CTP:PMA_2DG_15m	ns	0.9943
Inosine:PMA_2DG_15m vs. CTP:PMA_6AN_15m	ns	>0.9999
Inosine:PMA_2DG_15m vs. CTP:PMA_DPI_15m	ns	0.7158
Inosine:PMA_2DG_15m vs. CTP:PMA_AA_15m	ns	>0.9999
Inosine:PMA_2DG_15m vs. CTP:PMA_IAA_15m	****	<0.0001
Inosine:PMA_2DG_15m vs. Uridine:PMA_15m	ns	>0.9999
Inosine:PMA_2DG_15m vs. Uridine:PMA_2DG_15m	ns	>0.9999
Inosine:PMA_2DG_15m vs. Uridine:PMA_6AN_15m	ns	>0.9999
Inosine:PMA_2DG_15m vs. Uridine:PMA_DPI_15m	ns	>0.9999
Inosine:PMA_2DG_15m vs. Uridine:PMA_AA_15m	ns	>0.9999
Inosine:PMA_2DG_15m vs. Uridine:PMA_IAA_15m	ns	0.6327
Inosine:PMA_2DG_15m vs. UMP:PMA_15m	ns	>0.9999
Inosine:PMA_2DG_15m vs. UMP:PMA_2DG_15m	ns	>0.9999
Inosine:PMA_2DG_15m vs. UMP:PMA_6AN_15m	ns	>0.9999
Inosine:PMA_2DG_15m vs. UMP:PMA_DPI_15m	ns	>0.9999
Inosine:PMA_2DG_15m vs. UMP:PMA_AA_15m	ns	>0.9999
Inosine:PMA_2DG_15m vs. UMP:PMA_IAA_15m	ns	0.9906
Inosine:PMA_2DG_15m vs. UDP:PMA_15m	ns	>0.9999
Inosine:PMA_2DG_15m vs. UDP:PMA_2DG_15m	ns	>0.9999
Inosine:PMA_2DG_15m vs. UDP:PMA_6AN_15m	ns	>0.9999

Inosine:PMA_2DG_15m vs. UDP:PMA_DPI_15m	ns	>0.9999
Inosine:PMA_2DG_15m vs. UDP:PMA_AA_15m	ns	>0.9999
Inosine:PMA_2DG_15m vs. UDP:PMA_IAA_15m	ns	0.9889
Inosine:PMA_2DG_15m vs. UTP:PMA_15m	ns	0.192
Inosine:PMA_2DG_15m vs. UTP:PMA_2DG_15m	ns	0.9675
Inosine:PMA_2DG_15m vs. UTP:PMA_6AN_15m	ns	>0.9999
Inosine:PMA_2DG_15m vs. UTP:PMA_DPI_15m	ns	0.5493
Inosine:PMA_2DG_15m vs. UTP:PMA_AA_15m	ns	>0.9999
Inosine:PMA_2DG_15m vs. UTP:PMA_IAA_15m	****	<0.0001
Inosine:PMA_6AN_15m vs. Inosine:PMA_DPI_15m	ns	0.8059
Inosine:PMA_6AN_15m vs. Inosine:PMA_AA_15m	ns	>0.9999
Inosine:PMA_6AN_15m vs. Inosine:PMA_IAA_15m	****	<0.0001
Inosine:PMA_6AN_15m vs. hypoxanthine:PMA_15m	ns	0.2435
Inosine:PMA_6AN_15m vs. hypoxanthine:PMA_2DG_15m	ns	0.9994
Inosine:PMA_6AN_15m vs. hypoxanthine:PMA_6AN_15m	ns	>0.9999
Inosine:PMA_6AN_15m vs. hypoxanthine:PMA_DPI_15m	ns	0.6304
Inosine:PMA_6AN_15m vs. hypoxanthine:PMA_AA_15m	ns	>0.9999
Inosine:PMA_6AN_15m vs. hypoxanthine:PMA_IAA_15m	****	<0.0001
Inosine:PMA_6AN_15m vs. IMP:PMA_15m	ns	>0.9999
Inosine:PMA_6AN_15m vs. IMP:PMA_2DG_15m	ns	>0.9999
Inosine:PMA_6AN_15m vs. IMP:PMA_6AN_15m	ns	>0.9999
Inosine:PMA_6AN_15m vs. IMP:PMA_DPI_15m	ns	>0.9999
Inosine:PMA_6AN_15m vs. IMP:PMA_AA_15m	ns	>0.9999
Inosine:PMA_6AN_15m vs. IMP:PMA_IAA_15m	ns	0.5191
Inosine:PMA_6AN_15m vs. GMP:PMA_15m	ns	>0.9999
Inosine:PMA_6AN_15m vs. GMP:PMA_2DG_15m	ns	>0.9999
Inosine:PMA_6AN_15m vs. GMP:PMA_6AN_15m	ns	>0.9999
Inosine:PMA_6AN_15m vs. GMP:PMA_DPI_15m	ns	>0.9999
Inosine:PMA_6AN_15m vs. GMP:PMA_AA_15m	ns	>0.9999
Inosine:PMA_6AN_15m vs. GMP:PMA_IAA_15m	ns	0.9887
Inosine:PMA_6AN_15m vs. GDP:PMA_15m	ns	>0.9999
Inosine:PMA_6AN_15m vs. GDP:PMA_2DG_15m	ns	>0.9999
Inosine:PMA_6AN_15m vs. GDP:PMA_6AN_15m	ns	>0.9999
Inosine:PMA_6AN_15m vs. GDP:PMA_DPI_15m	ns	>0.9999
Inosine:PMA_6AN_15m vs. GDP:PMA_AA_15m	ns	>0.9999
Inosine:PMA_6AN_15m vs. GDP:PMA_IAA_15m	ns	0.9802
Inosine:PMA_6AN_15m vs. GTP:PMA_15m	*	0.0351
Inosine:PMA_6AN_15m vs. GTP:PMA_2DG_15m	ns	0.891
Inosine:PMA_6AN_15m vs. GTP:PMA_6AN_15m	ns	0.9374
Inosine:PMA_6AN_15m vs. GTP:PMA_DPI_15m	ns	0.1646
Inosine:PMA_6AN_15m vs. GTP:PMA_AA_15m	ns	0.9988
Inosine:PMA_6AN_15m vs. GTP:PMA_IAA_15m	****	<0.0001
Inosine:PMA_6AN_15m vs. Guanosine:PMA_15m	ns	>0.9999
Inosine:PMA_6AN_15m vs. Guanosine:PMA_2DG_15m	ns	>0.9999
Inosine:PMA_6AN_15m vs. Guanosine:PMA_6AN_15m	ns	>0.9999
Inosine:PMA_6AN_15m vs. Guanosine:PMA_DPI_15m	ns	>0.9999
Inosine:PMA_6AN_15m vs. Guanosine:PMA_AA_15m	ns	>0.9999

Inosine:PMA_6AN_15m vs. Guanosine:PMA_IAA_15m	ns	0.3308
Inosine:PMA_6AN_15m vs. Guanine:PMA_15m	ns	0.4187
Inosine:PMA_6AN_15m vs. Guanine:PMA_2DG_15m	ns	>0.9999
Inosine:PMA_6AN_15m vs. Guanine:PMA_6AN_15m	ns	>0.9999
Inosine:PMA_6AN_15m vs. Guanine:PMA_DPI_15m	ns	0.8217
Inosine:PMA_6AN_15m vs. Guanine:PMA_AA_15m	ns	>0.9999
Inosine:PMA_6AN_15m vs. Guanine:PMA_IAA_15m	***	0.0002
Inosine:PMA_6AN_15m vs. Aspartic acid:PMA_15m	ns	0.6542
Inosine:PMA_6AN_15m vs. Aspartic acid:PMA_2DG_15m	ns	>0.9999
Inosine:PMA_6AN_15m vs. Aspartic acid:PMA_6AN_15m	ns	>0.9999
Inosine:PMA_6AN_15m vs. Aspartic acid:PMA_DPI_15m	ns	0.9512
Inosine:PMA_6AN_15m vs. Aspartic acid:PMA_AA_15m	ns	>0.9999
Inosine:PMA_6AN_15m vs. Aspartic acid:PMA_IAA_15m	***	0.0006
Inosine:PMA_6AN_15m vs. AMP:PMA_15m	ns	>0.9999
Inosine:PMA_6AN_15m vs. AMP:PMA_2DG_15m	ns	>0.9999
Inosine:PMA_6AN_15m vs. AMP:PMA_6AN_15m	ns	>0.9999
Inosine:PMA_6AN_15m vs. AMP:PMA_DPI_15m	ns	>0.9999
Inosine:PMA_6AN_15m vs. AMP:PMA_AA_15m	ns	>0.9999
Inosine:PMA_6AN_15m vs. AMP:PMA_IAA_15m	ns	0.1117
Inosine:PMA_6AN_15m vs. ADP:PMA_15m	ns	0.9996
Inosine:PMA_6AN_15m vs. ADP:PMA_2DG_15m	ns	>0.9999
Inosine:PMA_6AN_15m vs. ADP:PMA_6AN_15m	ns	>0.9999
Inosine:PMA_6AN_15m vs. ADP:PMA_DPI_15m	ns	>0.9999
Inosine:PMA_6AN_15m vs. ADP:PMA_AA_15m	ns	>0.9999
Inosine:PMA_6AN_15m vs. ADP:PMA_IAA_15m	*	0.0357
Inosine:PMA_6AN_15m vs. ATP:PMA_15m	**	0.0097
Inosine:PMA_6AN_15m vs. ATP:PMA_2DG_15m	ns	0.6437
Inosine:PMA_6AN_15m vs. ATP:PMA_6AN_15m	ns	0.6959
Inosine:PMA_6AN_15m vs. ATP:PMA_DPI_15m	ns	0.057
Inosine:PMA_6AN_15m vs. ATP:PMA_AA_15m	ns	0.972
Inosine:PMA_6AN_15m vs. ATP:PMA_IAA_15m	****	<0.0001
Inosine:PMA_6AN_15m vs. Xanthine:PMA_15m	ns	0.389
Inosine:PMA_6AN_15m vs. Xanthine:PMA_2DG_15m	ns	>0.9999
Inosine:PMA_6AN_15m vs. Xanthine:PMA_6AN_15m	ns	>0.9999
Inosine:PMA_6AN_15m vs. Xanthine:PMA_DPI_15m	ns	0.7831
Inosine:PMA_6AN_15m vs. Xanthine:PMA_AA_15m	ns	>0.9999
Inosine:PMA_6AN_15m vs. CMP:PMA_15m	ns	>0.9999
Inosine:PMA_6AN_15m vs. CMP:PMA_2DG_15m	ns	>0.9999
Inosine:PMA_6AN_15m vs. CMP:PMA_6AN_15m	ns	>0.9999
Inosine:PMA_6AN_15m vs. CMP:PMA_DPI_15m	ns	>0.9999
Inosine:PMA_6AN_15m vs. CMP:PMA_AA_15m	ns	>0.9999
Inosine:PMA_6AN_15m vs. CMP:PMA_IAA_15m	ns	0.1615
Inosine:PMA_6AN_15m vs. CDP:PMA_15m	ns	>0.9999
Inosine:PMA_6AN_15m vs. CDP:PMA_2DG_15m	ns	>0.9999
Inosine:PMA_6AN_15m vs. CDP:PMA_6AN_15m	ns	>0.9999
Inosine:PMA_6AN_15m vs. CDP:PMA_DPI_15m	ns	>0.9999
Inosine:PMA_6AN_15m vs. CDP:PMA_AA_15m	ns	>0.9999

Inosine:PMA_6AN_15m vs. CDP:PMA_IAA_15m	ns	0.553
Inosine:PMA_6AN_15m vs. CTP:PMA_15m	ns	0.0914
Inosine:PMA_6AN_15m vs. CTP:PMA_2DG_15m	ns	0.9801
Inosine:PMA_6AN_15m vs. CTP:PMA_6AN_15m	ns	0.9943
Inosine:PMA_6AN_15m vs. CTP:PMA_DPI_15m	ns	0.3371
Inosine:PMA_6AN_15m vs. CTP:PMA_AA_15m	ns	>0.9999
Inosine:PMA_6AN_15m vs. CTP:PMA_IAA_15m	****	<0.0001
Inosine:PMA_6AN_15m vs. Uridine:PMA_15m	ns	>0.9999
Inosine:PMA_6AN_15m vs. Uridine:PMA_2DG_15m	ns	>0.9999
Inosine:PMA_6AN_15m vs. Uridine:PMA_6AN_15m	ns	>0.9999
Inosine:PMA_6AN_15m vs. Uridine:PMA_DPI_15m	ns	>0.9999
Inosine:PMA_6AN_15m vs. Uridine:PMA_AA_15m	ns	>0.9999
Inosine:PMA_6AN_15m vs. Uridine:PMA_IAA_15m	ns	0.27
Inosine:PMA_6AN_15m vs. UMP:PMA_15m	ns	>0.9999
Inosine:PMA_6AN_15m vs. UMP:PMA_2DG_15m	ns	>0.9999
Inosine:PMA_6AN_15m vs. UMP:PMA_6AN_15m	ns	>0.9999
Inosine:PMA_6AN_15m vs. UMP:PMA_DPI_15m	ns	>0.9999
Inosine:PMA_6AN_15m vs. UMP:PMA_AA_15m	ns	>0.9999
Inosine:PMA_6AN_15m vs. UMP:PMA_IAA_15m	ns	0.8547
Inosine:PMA_6AN_15m vs. UDP:PMA_15m	ns	>0.9999
Inosine:PMA_6AN_15m vs. UDP:PMA_2DG_15m	ns	>0.9999
Inosine:PMA_6AN_15m vs. UDP:PMA_6AN_15m	ns	>0.9999
Inosine:PMA_6AN_15m vs. UDP:PMA_DPI_15m	ns	>0.9999
Inosine:PMA_6AN_15m vs. UDP:PMA_AA_15m	ns	>0.9999
Inosine:PMA_6AN_15m vs. UDP:PMA_IAA_15m	ns	0.843
Inosine:PMA_6AN_15m vs. UTP:PMA_15m	*	0.0487
Inosine:PMA_6AN_15m vs. UTP:PMA_2DG_15m	ns	0.932
Inosine:PMA_6AN_15m vs. UTP:PMA_6AN_15m	ns	0.9675
Inosine:PMA_6AN_15m vs. UTP:PMA_DPI_15m	ns	0.2121
Inosine:PMA_6AN_15m vs. UTP:PMA_AA_15m	ns	0.9996
Inosine:PMA_6AN_15m vs. UTP:PMA_IAA_15m	****	<0.0001
Inosine:PMA_DPI_15m vs. Inosine:PMA_AA_15m	ns	0.5731
Inosine:PMA_DPI_15m vs. Inosine:PMA_IAA_15m	***	0.0004
Inosine:PMA_DPI_15m vs. hypoxanthine:PMA_15m	ns	0.999
Inosine:PMA_DPI_15m vs. hypoxanthine:PMA_2DG_15m	ns	>0.9999
Inosine:PMA_DPI_15m vs. hypoxanthine:PMA_6AN_15m	ns	>0.9999
Inosine:PMA_DPI_15m vs. hypoxanthine:PMA_DPI_15m	ns	>0.9999
Inosine:PMA_DPI_15m vs. hypoxanthine:PMA_AA_15m	ns	>0.9999
Inosine:PMA_DPI_15m vs. hypoxanthine:PMA_IAA_15m	*	0.0265
Inosine:PMA_DPI_15m vs. IMP:PMA_15m	ns	>0.9999
Inosine:PMA_DPI_15m vs. IMP:PMA_2DG_15m	ns	>0.9999
Inosine:PMA_DPI_15m vs. IMP:PMA_6AN_15m	ns	0.9996
Inosine:PMA_DPI_15m vs. IMP:PMA_DPI_15m	ns	>0.9999
Inosine:PMA_DPI_15m vs. IMP:PMA_AA_15m	ns	0.9981
Inosine:PMA_DPI_15m vs. IMP:PMA_IAA_15m	ns	>0.9999
Inosine:PMA_DPI_15m vs. GMP:PMA_15m	ns	>0.9999
Inosine:PMA_DPI_15m vs. GMP:PMA_2DG_15m	ns	0.9787

Inosine:PMA_DPI_15m vs. GMP:PMA_6AN_15m	ns	0.7857
Inosine:PMA_DPI_15m vs. GMP:PMA_DPI_15m	ns	>0.9999
Inosine:PMA_DPI_15m vs. GMP:PMA_AA_15m	ns	0.6771
Inosine:PMA_DPI_15m vs. GMP:PMA_IAA_15m	ns	>0.9999
Inosine:PMA_DPI_15m vs. GDP:PMA_15m	ns	>0.9999
Inosine:PMA_DPI_15m vs. GDP:PMA_2DG_15m	ns	0.9878
Inosine:PMA_DPI_15m vs. GDP:PMA_6AN_15m	ns	0.8347
Inosine:PMA_DPI_15m vs. GDP:PMA_DPI_15m	ns	>0.9999
Inosine:PMA_DPI_15m vs. GDP:PMA_AA_15m	ns	0.736
Inosine:PMA_DPI_15m vs. GDP:PMA_IAA_15m	ns	>0.9999
Inosine:PMA_DPI_15m vs. GTP:PMA_15m	ns	0.8709
Inosine:PMA_DPI_15m vs. GTP:PMA_2DG_15m	ns	>0.9999
Inosine:PMA_DPI_15m vs. GTP:PMA_6AN_15m	ns	>0.9999
Inosine:PMA_DPI_15m vs. GTP:PMA_DPI_15m	ns	0.9374
Inosine:PMA_DPI_15m vs. GTP:PMA_AA_15m	ns	>0.9999
Inosine:PMA_DPI_15m vs. GTP:PMA_IAA_15m	**	0.0022
Inosine:PMA_DPI_15m vs. Guanosine:PMA_15m	ns	>0.9999
Inosine:PMA_DPI_15m vs. Guanosine:PMA_2DG_15m	ns	>0.9999
Inosine:PMA_DPI_15m vs. Guanosine:PMA_6AN_15m	ns	>0.9999
Inosine:PMA_DPI_15m vs. Guanosine:PMA_DPI_15m	ns	>0.9999
Inosine:PMA_DPI_15m vs. Guanosine:PMA_AA_15m	ns	>0.9999
Inosine:PMA_DPI_15m vs. Guanosine:PMA_IAA_15m	ns	0.9998
Inosine:PMA_DPI_15m vs. Guanine:PMA_15m	ns	>0.9999
Inosine:PMA_DPI_15m vs. Guanine:PMA_2DG_15m	ns	>0.9999
Inosine:PMA_DPI_15m vs. Guanine:PMA_6AN_15m	ns	>0.9999
Inosine:PMA_DPI_15m vs. Guanine:PMA_DPI_15m	ns	>0.9999
Inosine:PMA_DPI_15m vs. Guanine:PMA_AA_15m	ns	>0.9999
Inosine:PMA_DPI_15m vs. Guanine:PMA_IAA_15m	ns	0.0612
Inosine:PMA_DPI_15m vs. Aspartic acid:PMA_15m	ns	>0.9999
Inosine:PMA_DPI_15m vs. Aspartic acid:PMA_2DG_15m	ns	>0.9999
Inosine:PMA_DPI_15m vs. Aspartic acid:PMA_6AN_15m	ns	>0.9999
Inosine:PMA_DPI_15m vs. Aspartic acid:PMA_DPI_15m	ns	>0.9999
Inosine:PMA_DPI_15m vs. Aspartic acid:PMA_AA_15m	ns	>0.9999
Inosine:PMA_DPI_15m vs. Aspartic acid:PMA_IAA_15m	ns	0.1413
Inosine:PMA_DPI_15m vs. AMP:PMA_15m	ns	>0.9999
Inosine:PMA_DPI_15m vs. AMP:PMA_2DG_15m	ns	>0.9999
Inosine:PMA_DPI_15m vs. AMP:PMA_6AN_15m	ns	>0.9999
Inosine:PMA_DPI_15m vs. AMP:PMA_DPI_15m	ns	>0.9999
Inosine:PMA_DPI_15m vs. AMP:PMA_AA_15m	ns	>0.9999
Inosine:PMA_DPI_15m vs. AMP:PMA_IAA_15m	ns	0.9832
Inosine:PMA_DPI_15m vs. ADP:PMA_15m	ns	>0.9999
Inosine:PMA_DPI_15m vs. ADP:PMA_2DG_15m	ns	>0.9999
Inosine:PMA_DPI_15m vs. ADP:PMA_6AN_15m	ns	>0.9999
Inosine:PMA_DPI_15m vs. ADP:PMA_DPI_15m	ns	>0.9999
Inosine:PMA_DPI_15m vs. ADP:PMA_AA_15m	ns	>0.9999
Inosine:PMA_DPI_15m vs. ADP:PMA_IAA_15m	ns	0.8706
Inosine:PMA_DPI_15m vs. ATP:PMA_15m	ns	0.6106

Inosine:PMA_DPI_15m vs. ATP:PMA_2DG_15m	ns	>0.9999
Inosine:PMA_DPI_15m vs. ATP:PMA_6AN_15m	ns	>0.9999
Inosine:PMA_DPI_15m vs. ATP:PMA_DPI_15m	ns	0.6959
Inosine:PMA_DPI_15m vs. ATP:PMA_AA_15m	ns	>0.9999
Inosine:PMA_DPI_15m vs. ATP:PMA_IAA_15m	***	0.0005
Inosine:PMA_DPI_15m vs. Xanthine:PMA_15m	ns	0.9999
Inosine:PMA_DPI_15m vs. Xanthine:PMA_2DG_15m	ns	>0.9999
Inosine:PMA_DPI_15m vs. Xanthine:PMA_6AN_15m	ns	>0.9999
Inosine:PMA_DPI_15m vs. Xanthine:PMA_DPI_15m	ns	>0.9999
Inosine:PMA_DPI_15m vs. Xanthine:PMA_AA_15m	ns	>0.9999
Inosine:PMA_DPI_15m vs. CMP:PMA_15m	ns	>0.9999
Inosine:PMA_DPI_15m vs. CMP:PMA_2DG_15m	ns	>0.9999
Inosine:PMA_DPI_15m vs. CMP:PMA_6AN_15m	ns	>0.9999
Inosine:PMA_DPI_15m vs. CMP:PMA_DPI_15m	ns	>0.9999
Inosine:PMA_DPI_15m vs. CMP:PMA_AA_15m	ns	>0.9999
Inosine:PMA_DPI_15m vs. CMP:PMA_IAA_15m	ns	0.9943
Inosine:PMA_DPI_15m vs. CDP:PMA_15m	ns	>0.9999
Inosine:PMA_DPI_15m vs. CDP:PMA_2DG_15m	ns	>0.9999
Inosine:PMA_DPI_15m vs. CDP:PMA_6AN_15m	ns	0.9994
Inosine:PMA_DPI_15m vs. CDP:PMA_DPI_15m	ns	>0.9999
Inosine:PMA_DPI_15m vs. CDP:PMA_AA_15m	ns	0.9971
Inosine:PMA_DPI_15m vs. CDP:PMA_IAA_15m	ns	>0.9999
Inosine:PMA_DPI_15m vs. CTP:PMA_15m	ns	0.9738
Inosine:PMA_DPI_15m vs. CTP:PMA_2DG_15m	ns	>0.9999
Inosine:PMA_DPI_15m vs. CTP:PMA_6AN_15m	ns	>0.9999
Inosine:PMA_DPI_15m vs. CTP:PMA_DPI_15m	ns	0.9943
Inosine:PMA_DPI_15m vs. CTP:PMA_AA_15m	ns	>0.9999
Inosine:PMA_DPI_15m vs. CTP:PMA_IAA_15m	**	0.007
Inosine:PMA_DPI_15m vs. Uridine:PMA_15m	ns	>0.9999
Inosine:PMA_DPI_15m vs. Uridine:PMA_2DG_15m	ns	>0.9999
Inosine:PMA_DPI_15m vs. Uridine:PMA_6AN_15m	ns	>0.9999
Inosine:PMA_DPI_15m vs. Uridine:PMA_DPI_15m	ns	>0.9999
Inosine:PMA_DPI_15m vs. Uridine:PMA_AA_15m	ns	>0.9999
Inosine:PMA_DPI_15m vs. Uridine:PMA_IAA_15m	ns	0.9993
Inosine:PMA_DPI_15m vs. UMP:PMA_15m	ns	>0.9999
Inosine:PMA_DPI_15m vs. UMP:PMA_2DG_15m	ns	0.9997
Inosine:PMA_DPI_15m vs. UMP:PMA_6AN_15m	ns	0.9752
Inosine:PMA_DPI_15m vs. UMP:PMA_DPI_15m	ns	>0.9999
Inosine:PMA_DPI_15m vs. UMP:PMA_AA_15m	ns	0.941
Inosine:PMA_DPI_15m vs. UMP:PMA_IAA_15m	ns	>0.9999
Inosine:PMA_DPI_15m vs. UDP:PMA_15m	ns	>0.9999
Inosine:PMA_DPI_15m vs. UDP:PMA_2DG_15m	ns	0.9998
Inosine:PMA_DPI_15m vs. UDP:PMA_6AN_15m	ns	0.9784
Inosine:PMA_DPI_15m vs. UDP:PMA_DPI_15m	ns	>0.9999
Inosine:PMA_DPI_15m vs. UDP:PMA_AA_15m	ns	0.9472
Inosine:PMA_DPI_15m vs. UDP:PMA_IAA_15m	ns	>0.9999
Inosine:PMA_DPI_15m vs. UTP:PMA_15m	ns	0.9171

Inosine:PMA_DPI_15m vs. UTP:PMA_2DG_15m	ns	>0.9999
Inosine:PMA_DPI_15m vs. UTP:PMA_6AN_15m	ns	>0.9999
Inosine:PMA_DPI_15m vs. UTP:PMA_DPI_15m	ns	0.9675
Inosine:PMA_DPI_15m vs. UTP:PMA_AA_15m	ns	>0.9999
Inosine:PMA_DPI_15m vs. UTP:PMA_IAA_15m	**	0.0032
Inosine:PMA_AA_15m vs. Inosine:PMA_IAA_15m	****	<0.0001
Inosine:PMA_AA_15m vs. hypoxanthine:PMA_15m	ns	0.1701
Inosine:PMA_AA_15m vs. hypoxanthine:PMA_2DG_15m	ns	0.9969
Inosine:PMA_AA_15m vs. hypoxanthine:PMA_6AN_15m	ns	>0.9999
Inosine:PMA_AA_15m vs. hypoxanthine:PMA_DPI_15m	ns	0.5101
Inosine:PMA_AA_15m vs. hypoxanthine:PMA_AA_15m	ns	>0.9999
Inosine:PMA_AA_15m vs. hypoxanthine:PMA_IAA_15m	****	<0.0001
Inosine:PMA_AA_15m vs. IMP:PMA_15m	ns	>0.9999
Inosine:PMA_AA_15m vs. IMP:PMA_2DG_15m	ns	>0.9999
Inosine:PMA_AA_15m vs. IMP:PMA_6AN_15m	ns	>0.9999
Inosine:PMA_AA_15m vs. IMP:PMA_DPI_15m	ns	>0.9999
Inosine:PMA_AA_15m vs. IMP:PMA_AA_15m	ns	>0.9999
Inosine:PMA_AA_15m vs. IMP:PMA_IAA_15m	ns	0.4035
Inosine:PMA_AA_15m vs. GMP:PMA_15m	ns	>0.9999
Inosine:PMA_AA_15m vs. GMP:PMA_2DG_15m	ns	>0.9999
Inosine:PMA_AA_15m vs. GMP:PMA_6AN_15m	ns	>0.9999
Inosine:PMA_AA_15m vs. GMP:PMA_DPI_15m	ns	>0.9999
Inosine:PMA_AA_15m vs. GMP:PMA_AA_15m	ns	>0.9999
Inosine:PMA_AA_15m vs. GMP:PMA_IAA_15m	ns	0.9687
Inosine:PMA_AA_15m vs. GDP:PMA_15m	ns	>0.9999
Inosine:PMA_AA_15m vs. GDP:PMA_2DG_15m	ns	>0.9999
Inosine:PMA_AA_15m vs. GDP:PMA_6AN_15m	ns	>0.9999
Inosine:PMA_AA_15m vs. GDP:PMA_DPI_15m	ns	>0.9999
Inosine:PMA_AA_15m vs. GDP:PMA_AA_15m	ns	>0.9999
Inosine:PMA_AA_15m vs. GDP:PMA_IAA_15m	ns	0.9509
Inosine:PMA_AA_15m vs. GTP:PMA_15m	*	0.0215
Inosine:PMA_AA_15m vs. GTP:PMA_2DG_15m	ns	0.8094
Inosine:PMA_AA_15m vs. GTP:PMA_6AN_15m	ns	0.9835
Inosine:PMA_AA_15m vs. GTP:PMA_DPI_15m	ns	0.1107
Inosine:PMA_AA_15m vs. GTP:PMA_AA_15m	ns	0.9374
Inosine:PMA_AA_15m vs. GTP:PMA_IAA_15m	****	<0.0001
Inosine:PMA_AA_15m vs. Guanosine:PMA_15m	ns	>0.9999
Inosine:PMA_AA_15m vs. Guanosine:PMA_2DG_15m	ns	>0.9999
Inosine:PMA_AA_15m vs. Guanosine:PMA_6AN_15m	ns	>0.9999
Inosine:PMA_AA_15m vs. Guanosine:PMA_DPI_15m	ns	>0.9999
Inosine:PMA_AA_15m vs. Guanosine:PMA_AA_15m	ns	>0.9999
Inosine:PMA_AA_15m vs. Guanosine:PMA_IAA_15m	ns	0.2398
Inosine:PMA_AA_15m vs. Guanine:PMA_15m	ns	0.3134
Inosine:PMA_AA_15m vs. Guanine:PMA_2DG_15m	ns	0.9998
Inosine:PMA_AA_15m vs. Guanine:PMA_6AN_15m	ns	>0.9999
Inosine:PMA_AA_15m vs. Guanine:PMA_DPI_15m	ns	0.72
Inosine:PMA_AA_15m vs. Guanine:PMA_AA_15m	ns	>0.9999

Inosine:PMA_AA_15m vs. Guanine:PMA_IAA_15m	****	<0.0001
Inosine:PMA_AA_15m vs. Aspartic acid:PMA_15m	ns	0.5342
Inosine:PMA_AA_15m vs. Aspartic acid:PMA_2DG_15m	ns	>0.9999
Inosine:PMA_AA_15m vs. Aspartic acid:PMA_6AN_15m	ns	>0.9999
Inosine:PMA_AA_15m vs. Aspartic acid:PMA_DPI_15m	ns	0.899
Inosine:PMA_AA_15m vs. Aspartic acid:PMA_AA_15m	ns	>0.9999
Inosine:PMA_AA_15m vs. Aspartic acid:PMA_IAA_15m	***	0.0003
Inosine:PMA_AA_15m vs. AMP:PMA_15m	ns	>0.9999
Inosine:PMA_AA_15m vs. AMP:PMA_2DG_15m	ns	>0.9999
Inosine:PMA_AA_15m vs. AMP:PMA_6AN_15m	ns	>0.9999
Inosine:PMA_AA_15m vs. AMP:PMA_DPI_15m	ns	>0.9999
Inosine:PMA_AA_15m vs. AMP:PMA_AA_15m	ns	>0.9999
Inosine:PMA_AA_15m vs. AMP:PMA_IAA_15m	ns	0.0731
Inosine:PMA_AA_15m vs. ADP:PMA_15m	ns	0.9981
Inosine:PMA_AA_15m vs. ADP:PMA_2DG_15m	ns	>0.9999
Inosine:PMA_AA_15m vs. ADP:PMA_6AN_15m	ns	>0.9999
Inosine:PMA_AA_15m vs. ADP:PMA_DPI_15m	ns	>0.9999
Inosine:PMA_AA_15m vs. ADP:PMA_AA_15m	ns	>0.9999
Inosine:PMA_AA_15m vs. ADP:PMA_IAA_15m	*	0.0219
Inosine:PMA_AA_15m vs. ATP:PMA_15m	**	0.0056
Inosine:PMA_AA_15m vs. ATP:PMA_2DG_15m	ns	0.5235
Inosine:PMA_AA_15m vs. ATP:PMA_6AN_15m	ns	0.8732
Inosine:PMA_AA_15m vs. ATP:PMA_DPI_15m	*	0.0357
Inosine:PMA_AA_15m vs. ATP:PMA_AA_15m	ns	0.6959
Inosine:PMA_AA_15m vs. ATP:PMA_IAA_15m	****	<0.0001
Inosine:PMA_AA_15m vs. Xanthine:PMA_15m	ns	0.2915
Inosine:PMA_AA_15m vs. Xanthine:PMA_2DG_15m	ns	0.9995
Inosine:PMA_AA_15m vs. Xanthine:PMA_6AN_15m	ns	>0.9999
Inosine:PMA_AA_15m vs. Xanthine:PMA_DPI_15m	ns	0.6785
Inosine:PMA_AA_15m vs. Xanthine:PMA_AA_15m	ns	>0.9999
Inosine:PMA_AA_15m vs. CMP:PMA_15m	ns	>0.9999
Inosine:PMA_AA_15m vs. CMP:PMA_2DG_15m	ns	>0.9999
Inosine:PMA_AA_15m vs. CMP:PMA_6AN_15m	ns	>0.9999
Inosine:PMA_AA_15m vs. CMP:PMA_DPI_15m	ns	>0.9999
Inosine:PMA_AA_15m vs. CMP:PMA_AA_15m	ns	>0.9999
Inosine:PMA_AA_15m vs. CMP:PMA_IAA_15m	ns	0.1087
Inosine:PMA_AA_15m vs. CDP:PMA_15m	ns	>0.9999
Inosine:PMA_AA_15m vs. CDP:PMA_2DG_15m	ns	>0.9999
Inosine:PMA_AA_15m vs. CDP:PMA_6AN_15m	ns	>0.9999
Inosine:PMA_AA_15m vs. CDP:PMA_DPI_15m	ns	>0.9999
Inosine:PMA_AA_15m vs. CDP:PMA_AA_15m	ns	>0.9999
Inosine:PMA_AA_15m vs. CDP:PMA_IAA_15m	ns	0.4352
Inosine:PMA_AA_15m vs. CTP:PMA_15m	ns	0.0589
Inosine:PMA_AA_15m vs. CTP:PMA_2DG_15m	ns	0.9505
Inosine:PMA_AA_15m vs. CTP:PMA_6AN_15m	ns	0.999
Inosine:PMA_AA_15m vs. CTP:PMA_DPI_15m	ns	0.2447
Inosine:PMA_AA_15m vs. CTP:PMA_AA_15m	ns	0.9943

Inosine:PMA_AA_15m vs. CTP:PMA_IAA_15m	****	<0.0001
Inosine:PMA_AA_15m vs. Uridine:PMA_15m	ns	>0.9999
Inosine:PMA_AA_15m vs. Uridine:PMA_2DG_15m	ns	>0.9999
Inosine:PMA_AA_15m vs. Uridine:PMA_6AN_15m	ns	>0.9999
Inosine:PMA_AA_15m vs. Uridine:PMA_DPI_15m	ns	>0.9999
Inosine:PMA_AA_15m vs. Uridine:PMA_AA_15m	ns	>0.9999
Inosine:PMA_AA_15m vs. Uridine:PMA_IAA_15m	ns	0.191
Inosine:PMA_AA_15m vs. UMP:PMA_15m	ns	>0.9999
Inosine:PMA_AA_15m vs. UMP:PMA_2DG_15m	ns	>0.9999
Inosine:PMA_AA_15m vs. UMP:PMA_6AN_15m	ns	>0.9999
Inosine:PMA_AA_15m vs. UMP:PMA_DPI_15m	ns	>0.9999
Inosine:PMA_AA_15m vs. UMP:PMA_AA_15m	ns	>0.9999
Inosine:PMA_AA_15m vs. UMP:PMA_IAA_15m	ns	0.7617
Inosine:PMA_AA_15m vs. UDP:PMA_15m	ns	>0.9999
Inosine:PMA_AA_15m vs. UDP:PMA_2DG_15m	ns	>0.9999
Inosine:PMA_AA_15m vs. UDP:PMA_6AN_15m	ns	>0.9999
Inosine:PMA_AA_15m vs. UDP:PMA_DPI_15m	ns	>0.9999
Inosine:PMA_AA_15m vs. UDP:PMA_AA_15m	ns	>0.9999
Inosine:PMA_AA_15m vs. UDP:PMA_IAA_15m	ns	0.7468
Inosine:PMA_AA_15m vs. UTP:PMA_15m	*	0.0302
Inosine:PMA_AA_15m vs. UTP:PMA_2DG_15m	ns	0.8685
Inosine:PMA_AA_15m vs. UTP:PMA_6AN_15m	ns	0.9925
Inosine:PMA_AA_15m vs. UTP:PMA_DPI_15m	ns	0.1461
Inosine:PMA_AA_15m vs. UTP:PMA_AA_15m	ns	0.9675
Inosine:PMA_AA_15m vs. UTP:PMA_IAA_15m	****	<0.0001
Inosine:PMA_IAA_15m vs. hypoxanthine:PMA_15m	ns	>0.9999
Inosine:PMA_IAA_15m vs. hypoxanthine:PMA_2DG_15m	ns	>0.9999
Inosine:PMA_IAA_15m vs. hypoxanthine:PMA_6AN_15m	ns	0.9982
Inosine:PMA_IAA_15m vs. hypoxanthine:PMA_DPI_15m	ns	>0.9999
Inosine:PMA_IAA_15m vs. hypoxanthine:PMA_AA_15m	ns	0.9929
Inosine:PMA_IAA_15m vs. hypoxanthine:PMA_IAA_15m	ns	>0.9999
Inosine:PMA_IAA_15m vs. IMP:PMA_15m	ns	0.8696
Inosine:PMA_IAA_15m vs. IMP:PMA_2DG_15m	*	0.031
Inosine:PMA_IAA_15m vs. IMP:PMA_6AN_15m	**	0.0056
Inosine:PMA_IAA_15m vs. IMP:PMA_DPI_15m	ns	0.4862
Inosine:PMA_IAA_15m vs. IMP:PMA_AA_15m	**	0.0032
Inosine:PMA_IAA_15m vs. IMP:PMA_IAA_15m	ns	>0.9999
Inosine:PMA_IAA_15m vs. GMP:PMA_15m	ns	0.2203
Inosine:PMA_IAA_15m vs. GMP:PMA_2DG_15m	**	0.001
Inosine:PMA_IAA_15m vs. GMP:PMA_6AN_15m	***	0.0001
Inosine:PMA_IAA_15m vs. GMP:PMA_DPI_15m	ns	0.0515
Inosine:PMA_IAA_15m vs. GMP:PMA_AA_15m	****	<0.0001
Inosine:PMA_IAA_15m vs. GMP:PMA_IAA_15m	ns	>0.9999
Inosine:PMA_IAA_15m vs. GDP:PMA_15m	ns	0.2632
Inosine:PMA_IAA_15m vs. GDP:PMA_2DG_15m	**	0.0014
Inosine:PMA_IAA_15m vs. GDP:PMA_6AN_15m	***	0.0002
Inosine:PMA_IAA_15m vs. GDP:PMA_DPI_15m	ns	0.0653

Inosine:PMA_IAA_15m vs. GDP:PMA_AA_15m	***	0.0001
Inosine:PMA_IAA_15m vs. GDP:PMA_IAA_15m	ns	>0.9999
Inosine:PMA_IAA_15m vs. GTP:PMA_15m	ns	>0.9999
Inosine:PMA_IAA_15m vs. GTP:PMA_2DG_15m	ns	>0.9999
Inosine:PMA_IAA_15m vs. GTP:PMA_6AN_15m	ns	>0.9999
Inosine:PMA_IAA_15m vs. GTP:PMA_DPI_15m	ns	>0.9999
Inosine:PMA_IAA_15m vs. GTP:PMA_AA_15m	ns	>0.9999
Inosine:PMA_IAA_15m vs. GTP:PMA_IAA_15m	ns	0.9374
Inosine:PMA_IAA_15m vs. Guanosine:PMA_15m	ns	0.9611
Inosine:PMA_IAA_15m vs. Guanosine:PMA_2DG_15m	ns	0.0681
Inosine:PMA_IAA_15m vs. Guanosine:PMA_6AN_15m	*	0.0139
Inosine:PMA_IAA_15m vs. Guanosine:PMA_DPI_15m	ns	0.687
Inosine:PMA_IAA_15m vs. Guanosine:PMA_AA_15m	**	0.0082
Inosine:PMA_IAA_15m vs. Guanosine:PMA_IAA_15m	ns	>0.9999
Inosine:PMA_IAA_15m vs. Guanine:PMA_15m	ns	>0.9999
Inosine:PMA_IAA_15m vs. Guanine:PMA_2DG_15m	ns	0.9999
Inosine:PMA_IAA_15m vs. Guanine:PMA_6AN_15m	ns	0.9829
Inosine:PMA_IAA_15m vs. Guanine:PMA_DPI_15m	ns	>0.9999
Inosine:PMA_IAA_15m vs. Guanine:PMA_AA_15m	ns	0.9564
Inosine:PMA_IAA_15m vs. Guanine:PMA_IAA_15m	ns	>0.9999
Inosine:PMA_IAA_15m vs. Aspartic acid:PMA_15m	ns	>0.9999
Inosine:PMA_IAA_15m vs. Aspartic acid:PMA_2DG_15m	ns	0.9964
Inosine:PMA_IAA_15m vs. Aspartic acid:PMA_6AN_15m	ns	0.9088
Inosine:PMA_IAA_15m vs. Aspartic acid:PMA_DPI_15m	ns	>0.9999
Inosine:PMA_IAA_15m vs. Aspartic acid:PMA_AA_15m	ns	0.8346
Inosine:PMA_IAA_15m vs. Aspartic acid:PMA_IAA_15m	ns	>0.9999
Inosine:PMA_IAA_15m vs. AMP:PMA_15m	ns	0.999
Inosine:PMA_IAA_15m vs. AMP:PMA_2DG_15m	ns	0.2271
Inosine:PMA_IAA_15m vs. AMP:PMA_6AN_15m	ns	0.0608
Inosine:PMA_IAA_15m vs. AMP:PMA_DPI_15m	ns	0.94
Inosine:PMA_IAA_15m vs. AMP:PMA_AA_15m	*	0.0383
Inosine:PMA_IAA_15m vs. AMP:PMA_IAA_15m	ns	>0.9999
Inosine:PMA_IAA_15m vs. ADP:PMA_15m	ns	>0.9999
Inosine:PMA_IAA_15m vs. ADP:PMA_2DG_15m	ns	0.4845
Inosine:PMA_IAA_15m vs. ADP:PMA_6AN_15m	ns	0.1749
Inosine:PMA_IAA_15m vs. ADP:PMA_DPI_15m	ns	0.9957
Inosine:PMA_IAA_15m vs. ADP:PMA_AA_15m	ns	0.1185
Inosine:PMA_IAA_15m vs. ADP:PMA_IAA_15m	ns	>0.9999
Inosine:PMA_IAA_15m vs. ATP:PMA_15m	ns	>0.9999
Inosine:PMA_IAA_15m vs. ATP:PMA_2DG_15m	ns	>0.9999
Inosine:PMA_IAA_15m vs. ATP:PMA_6AN_15m	ns	>0.9999
Inosine:PMA_IAA_15m vs. ATP:PMA_DPI_15m	ns	>0.9999
Inosine:PMA_IAA_15m vs. ATP:PMA_AA_15m	ns	>0.9999
Inosine:PMA_IAA_15m vs. ATP:PMA_IAA_15m	ns	0.6959
Inosine:PMA_IAA_15m vs. Xanthine:PMA_15m	ns	>0.9999
Inosine:PMA_IAA_15m vs. Xanthine:PMA_2DG_15m	ns	>0.9999
Inosine:PMA_IAA_15m vs. Xanthine:PMA_6AN_15m	ns	0.9975

Inosine:PMA_IAA_15m vs. Xanthine:PMA_DPI_15m	ns	>0.9999
Inosine:PMA_IAA_15m vs. Xanthine:PMA_AA_15m	ns	0.991
Inosine:PMA_IAA_15m vs. CMP:PMA_15m	ns	0.9961
Inosine:PMA_IAA_15m vs. CMP:PMA_2DG_15m	ns	0.1618
Inosine:PMA_IAA_15m vs. CMP:PMA_6AN_15m	*	0.0395
Inosine:PMA_IAA_15m vs. CMP:PMA_DPI_15m	ns	0.8861
Inosine:PMA_IAA_15m vs. CMP:PMA_AA_15m	*	0.0243
Inosine:PMA_IAA_15m vs. CMP:PMA_IAA_15m	ns	>0.9999
Inosine:PMA_IAA_15m vs. CDP:PMA_15m	ns	0.847
Inosine:PMA_IAA_15m vs. CDP:PMA_2DG_15m	*	0.027
Inosine:PMA_IAA_15m vs. CDP:PMA_6AN_15m	**	0.0048
Inosine:PMA_IAA_15m vs. CDP:PMA_DPI_15m	ns	0.4532
Inosine:PMA_IAA_15m vs. CDP:PMA_AA_15m	**	0.0027
Inosine:PMA_IAA_15m vs. CDP:PMA_IAA_15m	ns	>0.9999
Inosine:PMA_IAA_15m vs. CTP:PMA_15m	ns	>0.9999
Inosine:PMA_IAA_15m vs. CTP:PMA_2DG_15m	ns	>0.9999
Inosine:PMA_IAA_15m vs. CTP:PMA_6AN_15m	ns	>0.9999
Inosine:PMA_IAA_15m vs. CTP:PMA_DPI_15m	ns	>0.9999
Inosine:PMA_IAA_15m vs. CTP:PMA_AA_15m	ns	0.9999
Inosine:PMA_IAA_15m vs. CTP:PMA_IAA_15m	ns	0.9943
Inosine:PMA_IAA_15m vs. Uridine:PMA_15m	ns	0.9786
Inosine:PMA_IAA_15m vs. Uridine:PMA_2DG_15m	ns	0.0902
Inosine:PMA_IAA_15m vs. Uridine:PMA_6AN_15m	*	0.0194
Inosine:PMA_IAA_15m vs. Uridine:PMA_DPI_15m	ns	0.7579
Inosine:PMA_IAA_15m vs. Uridine:PMA_AA_15m	*	0.0116
Inosine:PMA_IAA_15m vs. Uridine:PMA_IAA_15m	ns	>0.9999
Inosine:PMA_IAA_15m vs. UMP:PMA_15m	ns	0.5417
Inosine:PMA_IAA_15m vs. UMP:PMA_2DG_15m	**	0.0062
Inosine:PMA_IAA_15m vs. UMP:PMA_6AN_15m	***	0.001
Inosine:PMA_IAA_15m vs. UMP:PMA_DPI_15m	ns	0.1885
Inosine:PMA_IAA_15m vs. UMP:PMA_AA_15m	***	0.0005
Inosine:PMA_IAA_15m vs. UMP:PMA_IAA_15m	ns	>0.9999
Inosine:PMA_IAA_15m vs. UDP:PMA_15m	ns	0.5588
Inosine:PMA_IAA_15m vs. UDP:PMA_2DG_15m	**	0.0067
Inosine:PMA_IAA_15m vs. UDP:PMA_6AN_15m	**	0.001
Inosine:PMA_IAA_15m vs. UDP:PMA_DPI_15m	ns	0.1984
Inosine:PMA_IAA_15m vs. UDP:PMA_AA_15m	***	0.0006
Inosine:PMA_IAA_15m vs. UDP:PMA_IAA_15m	ns	>0.9999
Inosine:PMA_IAA_15m vs. UTP:PMA_15m	ns	>0.9999
Inosine:PMA_IAA_15m vs. UTP:PMA_2DG_15m	ns	>0.9999
Inosine:PMA_IAA_15m vs. UTP:PMA_6AN_15m	ns	>0.9999
Inosine:PMA_IAA_15m vs. UTP:PMA_DPI_15m	ns	>0.9999
Inosine:PMA_IAA_15m vs. UTP:PMA_AA_15m	ns	>0.9999
Inosine:PMA_IAA_15m vs. UTP:PMA_IAA_15m	ns	0.9675
hypoxanthine:PMA_15m vs. hypoxanthine:PMA_2DG_15m	ns	0.7475
hypoxanthine:PMA_15m vs. hypoxanthine:PMA_6AN_15m	ns	0.1037
hypoxanthine:PMA_15m vs. hypoxanthine:PMA_DPI_15m	ns	>0.9999

hypoxanthine:PMA_15m vs. hypoxanthine:PMA_AA_15m	*	0.0409
hypoxanthine:PMA_15m vs. hypoxanthine:PMA_IAA_15m	*	0.0236
hypoxanthine:PMA_15m vs. IMP:PMA_15m	ns	0.8852
hypoxanthine:PMA_15m vs. IMP:PMA_2DG_15m	ns	0.1083
hypoxanthine:PMA_15m vs. IMP:PMA_6AN_15m	*	0.024
hypoxanthine:PMA_15m vs. IMP:PMA_DPI_15m	ns	0.8042
hypoxanthine:PMA_15m vs. IMP:PMA_AA_15m	*	0.0144
hypoxanthine:PMA_15m vs. IMP:PMA_IAA_15m	ns	>0.9999
hypoxanthine:PMA_15m vs. GMP:PMA_15m	ns	0.172
hypoxanthine:PMA_15m vs. GMP:PMA_2DG_15m	**	0.0051
hypoxanthine:PMA_15m vs. GMP:PMA_6AN_15m	***	0.0008
hypoxanthine:PMA_15m vs. GMP:PMA_DPI_15m	ns	0.1656
hypoxanthine:PMA_15m vs. GMP:PMA_AA_15m	***	0.0004
hypoxanthine:PMA_15m vs. GMP:PMA_IAA_15m	ns	>0.9999
hypoxanthine:PMA_15m vs. GDP:PMA_15m	ns	0.2138
hypoxanthine:PMA_15m vs. GDP:PMA_2DG_15m	**	0.0068
hypoxanthine:PMA_15m vs. GDP:PMA_6AN_15m	**	0.001
hypoxanthine:PMA_15m vs. GDP:PMA_DPI_15m	ns	0.2009
hypoxanthine:PMA_15m vs. GDP:PMA_AA_15m	***	0.0006
hypoxanthine:PMA_15m vs. GDP:PMA_IAA_15m	ns	>0.9999
hypoxanthine:PMA_15m vs. GTP:PMA_15m	ns	>0.9999
hypoxanthine:PMA_15m vs. GTP:PMA_2DG_15m	ns	>0.9999
hypoxanthine:PMA_15m vs. GTP:PMA_6AN_15m	ns	>0.9999
hypoxanthine:PMA_15m vs. GTP:PMA_DPI_15m	ns	>0.9999
hypoxanthine:PMA_15m vs. GTP:PMA_AA_15m	ns	>0.9999
hypoxanthine:PMA_15m vs. GTP:PMA_IAA_15m	ns	0.9207
hypoxanthine:PMA_15m vs. Guanosine:PMA_15m	ns	0.9743
hypoxanthine:PMA_15m vs. Guanosine:PMA_2DG_15m	ns	0.2079
hypoxanthine:PMA_15m vs. Guanosine:PMA_6AN_15m	ns	0.054
hypoxanthine:PMA_15m vs. Guanosine:PMA_DPI_15m	ns	0.9291
hypoxanthine:PMA_15m vs. Guanosine:PMA_AA_15m	*	0.0337
hypoxanthine:PMA_15m vs. Guanosine:PMA_IAA_15m	ns	>0.9999
hypoxanthine:PMA_15m vs. Guanine:PMA_15m	ns	>0.9999
hypoxanthine:PMA_15m vs. Guanine:PMA_2DG_15m	ns	>0.9999
hypoxanthine:PMA_15m vs. Guanine:PMA_6AN_15m	ns	0.9997
hypoxanthine:PMA_15m vs. Guanine:PMA_DPI_15m	ns	>0.9999
hypoxanthine:PMA_15m vs. Guanine:PMA_AA_15m	ns	0.9983
hypoxanthine:PMA_15m vs. Guanine:PMA_IAA_15m	ns	>0.9999
hypoxanthine:PMA_15m vs. Aspartic acid:PMA_15m	ns	>0.9999
hypoxanthine:PMA_15m vs. Aspartic acid:PMA_2DG_15m	ns	>0.9999
hypoxanthine:PMA_15m vs. Aspartic acid:PMA_6AN_15m	ns	0.9934
hypoxanthine:PMA_15m vs. Aspartic acid:PMA_DPI_15m	ns	>0.9999
hypoxanthine:PMA_15m vs. Aspartic acid:PMA_AA_15m	ns	0.9796
hypoxanthine:PMA_15m vs. Aspartic acid:PMA_IAA_15m	ns	>0.9999
hypoxanthine:PMA_15m vs. AMP:PMA_15m	ns	0.9998
hypoxanthine:PMA_15m vs. AMP:PMA_2DG_15m	ns	0.512
hypoxanthine:PMA_15m vs. AMP:PMA_6AN_15m	ns	0.1897

hypoxanthine:PMA_15m vs. AMP:PMA_DPI_15m	ns	0.997
hypoxanthine:PMA_15m vs. AMP:PMA_AA_15m	ns	0.1293
hypoxanthine:PMA_15m vs. AMP:PMA_IAA_15m	ns	>0.9999
hypoxanthine:PMA_15m vs. ADP:PMA_15m	ns	>0.9999
hypoxanthine:PMA_15m vs. ADP:PMA_2DG_15m	ns	0.8028
hypoxanthine:PMA_15m vs. ADP:PMA_6AN_15m	ns	0.4272
hypoxanthine:PMA_15m vs. ADP:PMA_DPI_15m	ns	>0.9999
hypoxanthine:PMA_15m vs. ADP:PMA_AA_15m	ns	0.3207
hypoxanthine:PMA_15m vs. ADP:PMA_IAA_15m	ns	>0.9999
hypoxanthine:PMA_15m vs. ATP:PMA_15m	ns	>0.9999
hypoxanthine:PMA_15m vs. ATP:PMA_2DG_15m	ns	>0.9999
hypoxanthine:PMA_15m vs. ATP:PMA_6AN_15m	ns	>0.9999
hypoxanthine:PMA_15m vs. ATP:PMA_DPI_15m	ns	>0.9999
hypoxanthine:PMA_15m vs. ATP:PMA_AA_15m	ns	>0.9999
hypoxanthine:PMA_15m vs. ATP:PMA_IAA_15m	ns	0.7004
hypoxanthine:PMA_15m vs. Xanthine:PMA_15m	ns	>0.9999
hypoxanthine:PMA_15m vs. Xanthine:PMA_2DG_15m	ns	>0.9999
hypoxanthine:PMA_15m vs. Xanthine:PMA_6AN_15m	ns	>0.9999
hypoxanthine:PMA_15m vs. Xanthine:PMA_DPI_15m	ns	>0.9999
hypoxanthine:PMA_15m vs. Xanthine:PMA_AA_15m	ns	>0.9999
hypoxanthine:PMA_15m vs. CMP:PMA_15m	ns	0.9987
hypoxanthine:PMA_15m vs. CMP:PMA_2DG_15m	ns	0.404
hypoxanthine:PMA_15m vs. CMP:PMA_6AN_15m	ns	0.1328
hypoxanthine:PMA_15m vs. CMP:PMA_DPI_15m	ns	0.9899
hypoxanthine:PMA_15m vs. CMP:PMA_AA_15m	ns	0.0878
hypoxanthine:PMA_15m vs. CMP:PMA_IAA_15m	ns	>0.9999
hypoxanthine:PMA_15m vs. CDP:PMA_15m	ns	0.861
hypoxanthine:PMA_15m vs. CDP:PMA_2DG_15m	ns	0.0962
hypoxanthine:PMA_15m vs. CDP:PMA_6AN_15m	*	0.0208
hypoxanthine:PMA_15m vs. CDP:PMA_DPI_15m	ns	0.7764
hypoxanthine:PMA_15m vs. CDP:PMA_AA_15m	*	0.0124
hypoxanthine:PMA_15m vs. CDP:PMA_IAA_15m	ns	>0.9999
hypoxanthine:PMA_15m vs. CTP:PMA_15m	ns	>0.9999
hypoxanthine:PMA_15m vs. CTP:PMA_2DG_15m	ns	>0.9999
hypoxanthine:PMA_15m vs. CTP:PMA_6AN_15m	ns	>0.9999
hypoxanthine:PMA_15m vs. CTP:PMA_DPI_15m	ns	>0.9999
hypoxanthine:PMA_15m vs. CTP:PMA_AA_15m	ns	>0.9999
hypoxanthine:PMA_15m vs. CTP:PMA_IAA_15m	ns	0.9879
hypoxanthine:PMA_15m vs. Uridine:PMA_15m	ns	0.988
hypoxanthine:PMA_15m vs. Uridine:PMA_2DG_15m	ns	0.2598
hypoxanthine:PMA_15m vs. Uridine:PMA_6AN_15m	ns	0.0722
hypoxanthine:PMA_15m vs. Uridine:PMA_DPI_15m	ns	0.9573
hypoxanthine:PMA_15m vs. Uridine:PMA_AA_15m	*	0.0458
hypoxanthine:PMA_15m vs. Uridine:PMA_IAA_15m	ns	>0.9999
hypoxanthine:PMA_15m vs. UMP:PMA_15m	ns	0.5138
hypoxanthine:PMA_15m vs. UMP:PMA_2DG_15m	*	0.0264
hypoxanthine:PMA_15m vs. UMP:PMA_6AN_15m	**	0.0046

hypoxanthine:PMA_15m vs. UMP:PMA_DPI_15m	ns	0.4504
hypoxanthine:PMA_15m vs. UMP:PMA_AA_15m	**	0.0026
hypoxanthine:PMA_15m vs. UMP:PMA_IAA_15m	ns	>0.9999
hypoxanthine:PMA_15m vs. UDP:PMA_15m	ns	0.5332
hypoxanthine:PMA_15m vs. UDP:PMA_2DG_15m	*	0.0283
hypoxanthine:PMA_15m vs. UDP:PMA_6AN_15m	**	0.005
hypoxanthine:PMA_15m vs. UDP:PMA_DPI_15m	ns	0.4669
hypoxanthine:PMA_15m vs. UDP:PMA_AA_15m	**	0.0029
hypoxanthine:PMA_15m vs. UDP:PMA_IAA_15m	ns	>0.9999
hypoxanthine:PMA_15m vs. UTP:PMA_15m	ns	>0.9999
hypoxanthine:PMA_15m vs. UTP:PMA_2DG_15m	ns	>0.9999
hypoxanthine:PMA_15m vs. UTP:PMA_6AN_15m	ns	>0.9999
hypoxanthine:PMA_15m vs. UTP:PMA_DPI_15m	ns	>0.9999
hypoxanthine:PMA_15m vs. UTP:PMA_AA_15m	ns	>0.9999
hypoxanthine:PMA_15m vs. UTP:PMA_IAA_15m	ns	0.9531
hypoxanthine:PMA_2DG_15m vs. hypoxanthine:PMA_6AN_15m	ns	>0.9999
hypoxanthine:PMA_2DG_15m vs. hypoxanthine:PMA_DPI_15m	ns	>0.9999
hypoxanthine:PMA_2DG_15m vs. hypoxanthine:PMA_AA_15m	ns	>0.9999
hypoxanthine:PMA_2DG_15m vs. hypoxanthine:PMA_IAA_15m	****	<0.0001
hypoxanthine:PMA_2DG_15m vs. IMP:PMA_15m	ns	>0.9999
hypoxanthine:PMA_2DG_15m vs. IMP:PMA_2DG_15m	ns	0.8852
hypoxanthine:PMA_2DG_15m vs. IMP:PMA_6AN_15m	ns	0.8298
hypoxanthine:PMA_2DG_15m vs. IMP:PMA_DPI_15m	ns	>0.9999
hypoxanthine:PMA_2DG_15m vs. IMP:PMA_AA_15m	ns	0.7299
hypoxanthine:PMA_2DG_15m vs. IMP:PMA_IAA_15m	ns	>0.9999
hypoxanthine:PMA_2DG_15m vs. GMP:PMA_15m	ns	>0.9999
hypoxanthine:PMA_2DG_15m vs. GMP:PMA_2DG_15m	ns	0.172
hypoxanthine:PMA_2DG_15m vs. GMP:PMA_6AN_15m	ns	0.1837
hypoxanthine:PMA_2DG_15m vs. GMP:PMA_DPI_15m	ns	0.9966
hypoxanthine:PMA_2DG_15m vs. GMP:PMA_AA_15m	ns	0.1248
hypoxanthine:PMA_2DG_15m vs. GMP:PMA_IAA_15m	ns	>0.9999
hypoxanthine:PMA_2DG_15m vs. GDP:PMA_15m	ns	>0.9999
hypoxanthine:PMA_2DG_15m vs. GDP:PMA_2DG_15m	ns	0.2138
hypoxanthine:PMA_2DG_15m vs. GDP:PMA_6AN_15m	ns	0.2218
hypoxanthine:PMA_2DG_15m vs. GDP:PMA_DPI_15m	ns	0.9984
hypoxanthine:PMA_2DG_15m vs. GDP:PMA_AA_15m	ns	0.1534
hypoxanthine:PMA_2DG_15m vs. GDP:PMA_IAA_15m	ns	>0.9999
hypoxanthine:PMA_2DG_15m vs. GTP:PMA_15m	ns	0.9999
hypoxanthine:PMA_2DG_15m vs. GTP:PMA_2DG_15m	ns	>0.9999
hypoxanthine:PMA_2DG_15m vs. GTP:PMA_6AN_15m	ns	>0.9999
hypoxanthine:PMA_2DG_15m vs. GTP:PMA_DPI_15m	ns	>0.9999
hypoxanthine:PMA_2DG_15m vs. GTP:PMA_AA_15m	ns	>0.9999
hypoxanthine:PMA_2DG_15m vs. GTP:PMA_IAA_15m	*	0.045
hypoxanthine:PMA_2DG_15m vs. Guanosine:PMA_15m	ns	>0.9999
hypoxanthine:PMA_2DG_15m vs. Guanosine:PMA_2DG_15m	ns	0.9743
hypoxanthine:PMA_2DG_15m vs. Guanosine:PMA_6AN_15m	ns	0.9425
hypoxanthine:PMA_2DG_15m vs. Guanosine:PMA_DPI_15m	ns	>0.9999

hypoxanthine:PMA_2DG_15m vs. Guanosine:PMA_AA_15m	ns	0.8849
hypoxanthine:PMA_2DG_15m vs. Guanosine:PMA_IAA_15m	ns	>0.9999
hypoxanthine:PMA_2DG_15m vs. Guanine:PMA_15m	ns	>0.9999
hypoxanthine:PMA_2DG_15m vs. Guanine:PMA_2DG_15m	ns	>0.9999
hypoxanthine:PMA_2DG_15m vs. Guanine:PMA_6AN_15m	ns	>0.9999
hypoxanthine:PMA_2DG_15m vs. Guanine:PMA_DPI_15m	ns	>0.9999
hypoxanthine:PMA_2DG_15m vs. Guanine:PMA_AA_15m	ns	>0.9999
hypoxanthine:PMA_2DG_15m vs. Guanine:PMA_IAA_15m	ns	0.4756
hypoxanthine:PMA_2DG_15m vs. Aspartic acid:PMA_15m	ns	>0.9999
hypoxanthine:PMA_2DG_15m vs. Aspartic acid:PMA_2DG_15m	ns	>0.9999
hypoxanthine:PMA_2DG_15m vs. Aspartic acid:PMA_6AN_15m	ns	>0.9999
hypoxanthine:PMA_2DG_15m vs. Aspartic acid:PMA_DPI_15m	ns	>0.9999
hypoxanthine:PMA_2DG_15m vs. Aspartic acid:PMA_AA_15m	ns	>0.9999
hypoxanthine:PMA_2DG_15m vs. Aspartic acid:PMA_IAA_15m	ns	0.7104
hypoxanthine:PMA_2DG_15m vs. AMP:PMA_15m	ns	>0.9999
hypoxanthine:PMA_2DG_15m vs. AMP:PMA_2DG_15m	ns	0.9998
hypoxanthine:PMA_2DG_15m vs. AMP:PMA_6AN_15m	ns	0.998
hypoxanthine:PMA_2DG_15m vs. AMP:PMA_DPI_15m	ns	>0.9999
hypoxanthine:PMA_2DG_15m vs. AMP:PMA_AA_15m	ns	0.9922
hypoxanthine:PMA_2DG_15m vs. AMP:PMA_IAA_15m	ns	>0.9999
hypoxanthine:PMA_2DG_15m vs. ADP:PMA_15m	ns	>0.9999
hypoxanthine:PMA_2DG_15m vs. ADP:PMA_2DG_15m	ns	>0.9999
hypoxanthine:PMA_2DG_15m vs. ADP:PMA_6AN_15m	ns	>0.9999
hypoxanthine:PMA_2DG_15m vs. ADP:PMA_DPI_15m	ns	>0.9999
hypoxanthine:PMA_2DG_15m vs. ADP:PMA_AA_15m	ns	0.9999
hypoxanthine:PMA_2DG_15m vs. ADP:PMA_IAA_15m	ns	0.9999
hypoxanthine:PMA_2DG_15m vs. ATP:PMA_15m	ns	0.9922
hypoxanthine:PMA_2DG_15m vs. ATP:PMA_2DG_15m	ns	>0.9999
hypoxanthine:PMA_2DG_15m vs. ATP:PMA_6AN_15m	ns	>0.9999
hypoxanthine:PMA_2DG_15m vs. ATP:PMA_DPI_15m	ns	>0.9999
hypoxanthine:PMA_2DG_15m vs. ATP:PMA_AA_15m	ns	>0.9999
hypoxanthine:PMA_2DG_15m vs. ATP:PMA_IAA_15m	*	0.0128
hypoxanthine:PMA_2DG_15m vs. Xanthine:PMA_15m	ns	>0.9999
hypoxanthine:PMA_2DG_15m vs. Xanthine:PMA_2DG_15m	ns	>0.9999
hypoxanthine:PMA_2DG_15m vs. Xanthine:PMA_6AN_15m	ns	>0.9999
hypoxanthine:PMA_2DG_15m vs. Xanthine:PMA_DPI_15m	ns	>0.9999
hypoxanthine:PMA_2DG_15m vs. Xanthine:PMA_AA_15m	ns	>0.9999
hypoxanthine:PMA_2DG_15m vs. CMP:PMA_15m	ns	>0.9999
hypoxanthine:PMA_2DG_15m vs. CMP:PMA_2DG_15m	ns	0.9987
hypoxanthine:PMA_2DG_15m vs. CMP:PMA_6AN_15m	ns	0.9928
hypoxanthine:PMA_2DG_15m vs. CMP:PMA_DPI_15m	ns	>0.9999
hypoxanthine:PMA_2DG_15m vs. CMP:PMA_AA_15m	ns	0.9781
hypoxanthine:PMA_2DG_15m vs. CMP:PMA_IAA_15m	ns	>0.9999
hypoxanthine:PMA_2DG_15m vs. CDP:PMA_15m	ns	>0.9999
hypoxanthine:PMA_2DG_15m vs. CDP:PMA_2DG_15m	ns	0.861
hypoxanthine:PMA_2DG_15m vs. CDP:PMA_6AN_15m	ns	0.8037
hypoxanthine:PMA_2DG_15m vs. CDP:PMA_DPI_15m	ns	>0.9999

hypoxanthine:PMA_2DG_15m vs. CDP:PMA_AA_15m	ns	0.6983
hypoxanthine:PMA_2DG_15m vs. CDP:PMA_IAA_15m	ns	>0.9999
hypoxanthine:PMA_2DG_15m vs. CTP:PMA_15m	ns	>0.9999
hypoxanthine:PMA_2DG_15m vs. CTP:PMA_2DG_15m	ns	>0.9999
hypoxanthine:PMA_2DG_15m vs. CTP:PMA_6AN_15m	ns	>0.9999
hypoxanthine:PMA_2DG_15m vs. CTP:PMA_DPI_15m	ns	>0.9999
hypoxanthine:PMA_2DG_15m vs. CTP:PMA_AA_15m	ns	>0.9999
hypoxanthine:PMA_2DG_15m vs. CTP:PMA_IAA_15m	ns	0.1132
hypoxanthine:PMA_2DG_15m vs. Uridine:PMA_15m	ns	>0.9999
hypoxanthine:PMA_2DG_15m vs. Uridine:PMA_2DG_15m	ns	0.988
hypoxanthine:PMA_2DG_15m vs. Uridine:PMA_6AN_15m	ns	0.9665
hypoxanthine:PMA_2DG_15m vs. Uridine:PMA_DPI_15m	ns	>0.9999
hypoxanthine:PMA_2DG_15m vs. Uridine:PMA_AA_15m	ns	0.9251
hypoxanthine:PMA_2DG_15m vs. Uridine:PMA_IAA_15m	ns	>0.9999
hypoxanthine:PMA_2DG_15m vs. UMP:PMA_15m	ns	>0.9999
hypoxanthine:PMA_2DG_15m vs. UMP:PMA_2DG_15m	ns	0.5138
hypoxanthine:PMA_2DG_15m vs. UMP:PMA_6AN_15m	ns	0.4828
hypoxanthine:PMA_2DG_15m vs. UMP:PMA_DPI_15m	ns	>0.9999
hypoxanthine:PMA_2DG_15m vs. UMP:PMA_AA_15m	ns	0.37
hypoxanthine:PMA_2DG_15m vs. UMP:PMA_IAA_15m	ns	>0.9999
hypoxanthine:PMA_2DG_15m vs. UDP:PMA_15m	ns	>0.9999
hypoxanthine:PMA_2DG_15m vs. UDP:PMA_2DG_15m	ns	0.5332
hypoxanthine:PMA_2DG_15m vs. UDP:PMA_6AN_15m	ns	0.4997
hypoxanthine:PMA_2DG_15m vs. UDP:PMA_DPI_15m	ns	>0.9999
hypoxanthine:PMA_2DG_15m vs. UDP:PMA_AA_15m	ns	0.3852
hypoxanthine:PMA_2DG_15m vs. UDP:PMA_IAA_15m	ns	>0.9999
hypoxanthine:PMA_2DG_15m vs. UTP:PMA_15m	ns	>0.9999
hypoxanthine:PMA_2DG_15m vs. UTP:PMA_2DG_15m	ns	>0.9999
hypoxanthine:PMA_2DG_15m vs. UTP:PMA_6AN_15m	ns	>0.9999
hypoxanthine:PMA_2DG_15m vs. UTP:PMA_DPI_15m	ns	>0.9999
hypoxanthine:PMA_2DG_15m vs. UTP:PMA_AA_15m	ns	>0.9999
hypoxanthine:PMA_2DG_15m vs. UTP:PMA_IAA_15m	ns	0.0617
hypoxanthine:PMA_6AN_15m vs. hypoxanthine:PMA_DPI_15m	ns	0.8059
hypoxanthine:PMA_6AN_15m vs. hypoxanthine:PMA_AA_15m	ns	>0.9999
hypoxanthine:PMA_6AN_15m vs. hypoxanthine:PMA_IAA_15m	****	<0.0001
hypoxanthine:PMA_6AN_15m vs. IMP:PMA_15m	ns	>0.9999
hypoxanthine:PMA_6AN_15m vs. IMP:PMA_2DG_15m	ns	>0.9999
hypoxanthine:PMA_6AN_15m vs. IMP:PMA_6AN_15m	ns	0.8852
hypoxanthine:PMA_6AN_15m vs. IMP:PMA_DPI_15m	ns	>0.9999
hypoxanthine:PMA_6AN_15m vs. IMP:PMA_AA_15m	ns	0.9649
hypoxanthine:PMA_6AN_15m vs. IMP:PMA_IAA_15m	ns	>0.9999
hypoxanthine:PMA_6AN_15m vs. GMP:PMA_15m	ns	>0.9999
hypoxanthine:PMA_6AN_15m vs. GMP:PMA_2DG_15m	ns	0.859
hypoxanthine:PMA_6AN_15m vs. GMP:PMA_6AN_15m	ns	0.172
hypoxanthine:PMA_6AN_15m vs. GMP:PMA_DPI_15m	ns	>0.9999
hypoxanthine:PMA_6AN_15m vs. GMP:PMA_AA_15m	ns	0.387
hypoxanthine:PMA_6AN_15m vs. GMP:PMA_IAA_15m	ns	>0.9999

hypoxanthine:PMA_6AN_15m vs. GDP:PMA_15m	ns	>0.9999
hypoxanthine:PMA_6AN_15m vs. GDP:PMA_2DG_15m	ns	0.8972
hypoxanthine:PMA_6AN_15m vs. GDP:PMA_6AN_15m	ns	0.2138
hypoxanthine:PMA_6AN_15m vs. GDP:PMA_DPI_15m	ns	>0.9999
hypoxanthine:PMA_6AN_15m vs. GDP:PMA_AA_15m	ns	0.4457
hypoxanthine:PMA_6AN_15m vs. GDP:PMA_IAA_15m	ns	>0.9999
hypoxanthine:PMA_6AN_15m vs. GTP:PMA_15m	ns	0.9818
hypoxanthine:PMA_6AN_15m vs. GTP:PMA_2DG_15m	ns	>0.9999
hypoxanthine:PMA_6AN_15m vs. GTP:PMA_6AN_15m	ns	>0.9999
hypoxanthine:PMA_6AN_15m vs. GTP:PMA_DPI_15m	ns	>0.9999
hypoxanthine:PMA_6AN_15m vs. GTP:PMA_AA_15m	ns	>0.9999
hypoxanthine:PMA_6AN_15m vs. GTP:PMA_IAA_15m	**	0.0086
hypoxanthine:PMA_6AN_15m vs. Guanosine:PMA_15m	ns	>0.9999
hypoxanthine:PMA_6AN_15m vs. Guanosine:PMA_2DG_15m	ns	>0.9999
hypoxanthine:PMA_6AN_15m vs. Guanosine:PMA_6AN_15m	ns	0.9743
hypoxanthine:PMA_6AN_15m vs. Guanosine:PMA_DPI_15m	ns	>0.9999
hypoxanthine:PMA_6AN_15m vs. Guanosine:PMA_AA_15m	ns	0.9943
hypoxanthine:PMA_6AN_15m vs. Guanosine:PMA_IAA_15m	ns	>0.9999
hypoxanthine:PMA_6AN_15m vs. Guanine:PMA_15m	ns	>0.9999
hypoxanthine:PMA_6AN_15m vs. Guanine:PMA_2DG_15m	ns	>0.9999
hypoxanthine:PMA_6AN_15m vs. Guanine:PMA_6AN_15m	ns	>0.9999
hypoxanthine:PMA_6AN_15m vs. Guanine:PMA_DPI_15m	ns	>0.9999
hypoxanthine:PMA_6AN_15m vs. Guanine:PMA_AA_15m	ns	>0.9999
hypoxanthine:PMA_6AN_15m vs. Guanine:PMA_IAA_15m	ns	0.17
hypoxanthine:PMA_6AN_15m vs. Aspartic acid:PMA_15m	ns	>0.9999
hypoxanthine:PMA_6AN_15m vs. Aspartic acid:PMA_2DG_15m	ns	>0.9999
hypoxanthine:PMA_6AN_15m vs. Aspartic acid:PMA_6AN_15m	ns	>0.9999
hypoxanthine:PMA_6AN_15m vs. Aspartic acid:PMA_DPI_15m	ns	>0.9999
hypoxanthine:PMA_6AN_15m vs. Aspartic acid:PMA_AA_15m	ns	>0.9999
hypoxanthine:PMA_6AN_15m vs. Aspartic acid:PMA_IAA_15m	ns	0.3334
hypoxanthine:PMA_6AN_15m vs. AMP:PMA_15m	ns	>0.9999
hypoxanthine:PMA_6AN_15m vs. AMP:PMA_2DG_15m	ns	>0.9999
hypoxanthine:PMA_6AN_15m vs. AMP:PMA_6AN_15m	ns	0.9998
hypoxanthine:PMA_6AN_15m vs. AMP:PMA_DPI_15m	ns	>0.9999
hypoxanthine:PMA_6AN_15m vs. AMP:PMA_AA_15m	ns	>0.9999
hypoxanthine:PMA_6AN_15m vs. AMP:PMA_IAA_15m	ns	0.9994
hypoxanthine:PMA_6AN_15m vs. ADP:PMA_15m	ns	>0.9999
hypoxanthine:PMA_6AN_15m vs. ADP:PMA_2DG_15m	ns	>0.9999
hypoxanthine:PMA_6AN_15m vs. ADP:PMA_6AN_15m	ns	>0.9999
hypoxanthine:PMA_6AN_15m vs. ADP:PMA_DPI_15m	ns	>0.9999
hypoxanthine:PMA_6AN_15m vs. ADP:PMA_AA_15m	ns	>0.9999
hypoxanthine:PMA_6AN_15m vs. ADP:PMA_IAA_15m	ns	0.9815
hypoxanthine:PMA_6AN_15m vs. ATP:PMA_15m	ns	0.8662
hypoxanthine:PMA_6AN_15m vs. ATP:PMA_2DG_15m	ns	>0.9999
hypoxanthine:PMA_6AN_15m vs. ATP:PMA_6AN_15m	ns	>0.9999
hypoxanthine:PMA_6AN_15m vs. ATP:PMA_DPI_15m	ns	0.9945
hypoxanthine:PMA_6AN_15m vs. ATP:PMA_AA_15m	ns	>0.9999

hypoxanthine:PMA_6AN_15m vs. ATP:PMA_IAA_15m	**	0.0021
hypoxanthine:PMA_6AN_15m vs. Xanthine:PMA_15m	ns	>0.9999
hypoxanthine:PMA_6AN_15m vs. Xanthine:PMA_2DG_15m	ns	>0.9999
hypoxanthine:PMA_6AN_15m vs. Xanthine:PMA_6AN_15m	ns	>0.9999
hypoxanthine:PMA_6AN_15m vs. Xanthine:PMA_DPI_15m	ns	>0.9999
hypoxanthine:PMA_6AN_15m vs. Xanthine:PMA_AA_15m	ns	>0.9999
hypoxanthine:PMA_6AN_15m vs. CMP:PMA_15m	ns	>0.9999
hypoxanthine:PMA_6AN_15m vs. CMP:PMA_2DG_15m	ns	>0.9999
hypoxanthine:PMA_6AN_15m vs. CMP:PMA_6AN_15m	ns	0.9987
hypoxanthine:PMA_6AN_15m vs. CMP:PMA_DPI_15m	ns	>0.9999
hypoxanthine:PMA_6AN_15m vs. CMP:PMA_AA_15m	ns	0.9998
hypoxanthine:PMA_6AN_15m vs. CMP:PMA_IAA_15m	ns	>0.9999
hypoxanthine:PMA_6AN_15m vs. CDP:PMA_15m	ns	>0.9999
hypoxanthine:PMA_6AN_15m vs. CDP:PMA_2DG_15m	ns	0.9999
hypoxanthine:PMA_6AN_15m vs. CDP:PMA_6AN_15m	ns	0.861
hypoxanthine:PMA_6AN_15m vs. CDP:PMA_DPI_15m	ns	>0.9999
hypoxanthine:PMA_6AN_15m vs. CDP:PMA_AA_15m	ns	0.9552
hypoxanthine:PMA_6AN_15m vs. CDP:PMA_IAA_15m	ns	>0.9999
hypoxanthine:PMA_6AN_15m vs. CTP:PMA_15m	ns	0.9988
hypoxanthine:PMA_6AN_15m vs. CTP:PMA_2DG_15m	ns	>0.9999
hypoxanthine:PMA_6AN_15m vs. CTP:PMA_6AN_15m	ns	>0.9999
hypoxanthine:PMA_6AN_15m vs. CTP:PMA_DPI_15m	ns	>0.9999
hypoxanthine:PMA_6AN_15m vs. CTP:PMA_AA_15m	ns	>0.9999
hypoxanthine:PMA_6AN_15m vs. CTP:PMA_IAA_15m	*	0.0255
hypoxanthine:PMA_6AN_15m vs. Uridine:PMA_15m	ns	>0.9999
hypoxanthine:PMA_6AN_15m vs. Uridine:PMA_2DG_15m	ns	>0.9999
hypoxanthine:PMA_6AN_15m vs. Uridine:PMA_6AN_15m	ns	0.988
hypoxanthine:PMA_6AN_15m vs. Uridine:PMA_DPI_15m	ns	>0.9999
hypoxanthine:PMA_6AN_15m vs. Uridine:PMA_AA_15m	ns	0.9976
hypoxanthine:PMA_6AN_15m vs. Uridine:PMA_IAA_15m	ns	>0.9999
hypoxanthine:PMA_6AN_15m vs. UMP:PMA_15m	ns	>0.9999
hypoxanthine:PMA_6AN_15m vs. UMP:PMA_2DG_15m	ns	0.9895
hypoxanthine:PMA_6AN_15m vs. UMP:PMA_6AN_15m	ns	0.5138
hypoxanthine:PMA_6AN_15m vs. UMP:PMA_DPI_15m	ns	>0.9999
hypoxanthine:PMA_6AN_15m vs. UMP:PMA_AA_15m	ns	0.7502
hypoxanthine:PMA_6AN_15m vs. UMP:PMA_IAA_15m	ns	>0.9999
hypoxanthine:PMA_6AN_15m vs. UDP:PMA_15m	ns	>0.9999
hypoxanthine:PMA_6AN_15m vs. UDP:PMA_2DG_15m	ns	0.9911
hypoxanthine:PMA_6AN_15m vs. UDP:PMA_6AN_15m	ns	0.5332
hypoxanthine:PMA_6AN_15m vs. UDP:PMA_DPI_15m	ns	>0.9999
hypoxanthine:PMA_6AN_15m vs. UDP:PMA_AA_15m	ns	0.7651
hypoxanthine:PMA_6AN_15m vs. UDP:PMA_IAA_15m	ns	>0.9999
hypoxanthine:PMA_6AN_15m vs. UTP:PMA_15m	ns	0.9916
hypoxanthine:PMA_6AN_15m vs. UTP:PMA_2DG_15m	ns	>0.9999
hypoxanthine:PMA_6AN_15m vs. UTP:PMA_6AN_15m	ns	>0.9999
hypoxanthine:PMA_6AN_15m vs. UTP:PMA_DPI_15m	ns	>0.9999
hypoxanthine:PMA_6AN_15m vs. UTP:PMA_AA_15m	ns	>0.9999

hypoxanthine:PMA_6AN_15m vs. UTP:PMA_IAA_15m	*	0.0124
hypoxanthine:PMA_DPI_15m vs. hypoxanthine:PMA_AA_15m	ns	0.5731
hypoxanthine:PMA_DPI_15m vs. hypoxanthine:PMA_IAA_15m	***	0.0004
hypoxanthine:PMA_DPI_15m vs. IMP:PMA_15m	ns	>0.9999
hypoxanthine:PMA_DPI_15m vs. IMP:PMA_2DG_15m	ns	0.3793
hypoxanthine:PMA_DPI_15m vs. IMP:PMA_6AN_15m	ns	0.1213
hypoxanthine:PMA_DPI_15m vs. IMP:PMA_DPI_15m	ns	0.8852
hypoxanthine:PMA_DPI_15m vs. IMP:PMA_AA_15m	ns	0.0797
hypoxanthine:PMA_DPI_15m vs. IMP:PMA_IAA_15m	ns	>0.9999
hypoxanthine:PMA_DPI_15m vs. GMP:PMA_15m	ns	0.8802
hypoxanthine:PMA_DPI_15m vs. GMP:PMA_2DG_15m	*	0.0326
hypoxanthine:PMA_DPI_15m vs. GMP:PMA_6AN_15m	**	0.0059
hypoxanthine:PMA_DPI_15m vs. GMP:PMA_DPI_15m	ns	0.172
hypoxanthine:PMA_DPI_15m vs. GMP:PMA_AA_15m	**	0.0034
hypoxanthine:PMA_DPI_15m vs. GMP:PMA_IAA_15m	ns	>0.9999
hypoxanthine:PMA_DPI_15m vs. GDP:PMA_15m	ns	0.9145
hypoxanthine:PMA_DPI_15m vs. GDP:PMA_2DG_15m	*	0.0419
hypoxanthine:PMA_DPI_15m vs. GDP:PMA_6AN_15m	**	0.0079
hypoxanthine:PMA_DPI_15m vs. GDP:PMA_DPI_15m	ns	0.2138
hypoxanthine:PMA_DPI_15m vs. GDP:PMA_AA_15m	**	0.0045
hypoxanthine:PMA_DPI_15m vs. GDP:PMA_IAA_15m	ns	>0.9999
hypoxanthine:PMA_DPI_15m vs. GTP:PMA_15m	ns	>0.9999
hypoxanthine:PMA_DPI_15m vs. GTP:PMA_2DG_15m	ns	>0.9999
hypoxanthine:PMA_DPI_15m vs. GTP:PMA_6AN_15m	ns	>0.9999
hypoxanthine:PMA_DPI_15m vs. GTP:PMA_DPI_15m	ns	>0.9999
hypoxanthine:PMA_DPI_15m vs. GTP:PMA_AA_15m	ns	>0.9999
hypoxanthine:PMA_DPI_15m vs. GTP:PMA_IAA_15m	ns	0.5791
hypoxanthine:PMA_DPI_15m vs. Guanosine:PMA_15m	ns	>0.9999
hypoxanthine:PMA_DPI_15m vs. Guanosine:PMA_2DG_15m	ns	0.576
hypoxanthine:PMA_DPI_15m vs. Guanosine:PMA_6AN_15m	ns	0.2293
hypoxanthine:PMA_DPI_15m vs. Guanosine:PMA_DPI_15m	ns	0.9743
hypoxanthine:PMA_DPI_15m vs. Guanosine:PMA_AA_15m	ns	0.1592
hypoxanthine:PMA_DPI_15m vs. Guanosine:PMA_IAA_15m	ns	>0.9999
hypoxanthine:PMA_DPI_15m vs. Guanine:PMA_15m	ns	>0.9999
hypoxanthine:PMA_DPI_15m vs. Guanine:PMA_2DG_15m	ns	>0.9999
hypoxanthine:PMA_DPI_15m vs. Guanine:PMA_6AN_15m	ns	>0.9999
hypoxanthine:PMA_DPI_15m vs. Guanine:PMA_DPI_15m	ns	>0.9999
hypoxanthine:PMA_DPI_15m vs. Guanine:PMA_AA_15m	ns	>0.9999
hypoxanthine:PMA_DPI_15m vs. Guanine:PMA_IAA_15m	ns	0.9952
hypoxanthine:PMA_DPI_15m vs. Aspartic acid:PMA_15m	ns	>0.9999
hypoxanthine:PMA_DPI_15m vs. Aspartic acid:PMA_2DG_15m	ns	>0.9999
hypoxanthine:PMA_DPI_15m vs. Aspartic acid:PMA_6AN_15m	ns	>0.9999
hypoxanthine:PMA_DPI_15m vs. Aspartic acid:PMA_DPI_15m	ns	>0.9999
hypoxanthine:PMA_DPI_15m vs. Aspartic acid:PMA_AA_15m	ns	>0.9999
hypoxanthine:PMA_DPI_15m vs. Aspartic acid:PMA_IAA_15m	ns	0.9998
hypoxanthine:PMA_DPI_15m vs. AMP:PMA_15m	ns	>0.9999
hypoxanthine:PMA_DPI_15m vs. AMP:PMA_2DG_15m	ns	0.8864

hypoxanthine:PMA_DPI_15m vs. AMP:PMA_6AN_15m	ns	0.5454
hypoxanthine:PMA_DPI_15m vs. AMP:PMA_DPI_15m	ns	0.9998
hypoxanthine:PMA_DPI_15m vs. AMP:PMA_AA_15m	ns	0.4276
hypoxanthine:PMA_DPI_15m vs. AMP:PMA_IAA_15m	ns	>0.9999
hypoxanthine:PMA_DPI_15m vs. ADP:PMA_15m	ns	>0.9999
hypoxanthine:PMA_DPI_15m vs. ADP:PMA_2DG_15m	ns	0.9868
hypoxanthine:PMA_DPI_15m vs. ADP:PMA_6AN_15m	ns	0.8285
hypoxanthine:PMA_DPI_15m vs. ADP:PMA_DPI_15m	ns	>0.9999
hypoxanthine:PMA_DPI_15m vs. ADP:PMA_AA_15m	ns	0.7283
hypoxanthine:PMA_DPI_15m vs. ADP:PMA_IAA_15m	ns	>0.9999
hypoxanthine:PMA_DPI_15m vs. ATP:PMA_15m	ns	>0.9999
hypoxanthine:PMA_DPI_15m vs. ATP:PMA_2DG_15m	ns	>0.9999
hypoxanthine:PMA_DPI_15m vs. ATP:PMA_6AN_15m	ns	>0.9999
hypoxanthine:PMA_DPI_15m vs. ATP:PMA_DPI_15m	ns	>0.9999
hypoxanthine:PMA_DPI_15m vs. ATP:PMA_AA_15m	ns	>0.9999
hypoxanthine:PMA_DPI_15m vs. ATP:PMA_IAA_15m	ns	0.2978
hypoxanthine:PMA_DPI_15m vs. Xanthine:PMA_15m	ns	>0.9999
hypoxanthine:PMA_DPI_15m vs. Xanthine:PMA_2DG_15m	ns	>0.9999
hypoxanthine:PMA_DPI_15m vs. Xanthine:PMA_6AN_15m	ns	>0.9999
hypoxanthine:PMA_DPI_15m vs. Xanthine:PMA_DPI_15m	ns	>0.9999
hypoxanthine:PMA_DPI_15m vs. Xanthine:PMA_AA_15m	ns	>0.9999
hypoxanthine:PMA_DPI_15m vs. CMP:PMA_15m	ns	>0.9999
hypoxanthine:PMA_DPI_15m vs. CMP:PMA_2DG_15m	ns	0.8095
hypoxanthine:PMA_DPI_15m vs. CMP:PMA_6AN_15m	ns	0.4352
hypoxanthine:PMA_DPI_15m vs. CMP:PMA_DPI_15m	ns	0.9987
hypoxanthine:PMA_DPI_15m vs. CMP:PMA_AA_15m	ns	0.3278
hypoxanthine:PMA_DPI_15m vs. CMP:PMA_IAA_15m	ns	>0.9999
hypoxanthine:PMA_DPI_15m vs. CDP:PMA_15m	ns	>0.9999
hypoxanthine:PMA_DPI_15m vs. CDP:PMA_2DG_15m	ns	0.3495
hypoxanthine:PMA_DPI_15m vs. CDP:PMA_6AN_15m	ns	0.108
hypoxanthine:PMA_DPI_15m vs. CDP:PMA_DPI_15m	ns	0.861
hypoxanthine:PMA_DPI_15m vs. CDP:PMA_AA_15m	ns	0.0704
hypoxanthine:PMA_DPI_15m vs. CDP:PMA_IAA_15m	ns	>0.9999
hypoxanthine:PMA_DPI_15m vs. CTP:PMA_15m	ns	>0.9999
hypoxanthine:PMA_DPI_15m vs. CTP:PMA_2DG_15m	ns	>0.9999
hypoxanthine:PMA_DPI_15m vs. CTP:PMA_6AN_15m	ns	>0.9999
hypoxanthine:PMA_DPI_15m vs. CTP:PMA_DPI_15m	ns	>0.9999
hypoxanthine:PMA_DPI_15m vs. CTP:PMA_AA_15m	ns	>0.9999
hypoxanthine:PMA_DPI_15m vs. CTP:PMA_IAA_15m	ns	0.8118
hypoxanthine:PMA_DPI_15m vs. Uridine:PMA_15m	ns	>0.9999
hypoxanthine:PMA_DPI_15m vs. Uridine:PMA_2DG_15m	ns	0.6533
hypoxanthine:PMA_DPI_15m vs. Uridine:PMA_6AN_15m	ns	0.2846
hypoxanthine:PMA_DPI_15m vs. Uridine:PMA_DPI_15m	ns	0.988
hypoxanthine:PMA_DPI_15m vs. Uridine:PMA_AA_15m	ns	0.2023
hypoxanthine:PMA_DPI_15m vs. Uridine:PMA_IAA_15m	ns	>0.9999
hypoxanthine:PMA_DPI_15m vs. UMP:PMA_15m	ns	0.9924
hypoxanthine:PMA_DPI_15m vs. UMP:PMA_2DG_15m	ns	0.1308

hypoxanthine:PMA_DPI_15m vs. UMP:PMA_6AN_15m	*	0.0302
hypoxanthine:PMA_DPI_15m vs. UMP:PMA_DPI_15m	ns	0.5138
hypoxanthine:PMA_DPI_15m vs. UMP:PMA_AA_15m	*	0.0183
hypoxanthine:PMA_DPI_15m vs. UMP:PMA_IAA_15m	ns	>0.9999
hypoxanthine:PMA_DPI_15m vs. UDP:PMA_15m	ns	0.9937
hypoxanthine:PMA_DPI_15m vs. UDP:PMA_2DG_15m	ns	0.1384
hypoxanthine:PMA_DPI_15m vs. UDP:PMA_6AN_15m	*	0.0324
hypoxanthine:PMA_DPI_15m vs. UDP:PMA_DPI_15m	ns	0.5332
hypoxanthine:PMA_DPI_15m vs. UDP:PMA_AA_15m	*	0.0197
hypoxanthine:PMA_DPI_15m vs. UDP:PMA_IAA_15m	ns	>0.9999
hypoxanthine:PMA_DPI_15m vs. UTP:PMA_15m	ns	>0.9999
hypoxanthine:PMA_DPI_15m vs. UTP:PMA_2DG_15m	ns	>0.9999
hypoxanthine:PMA_DPI_15m vs. UTP:PMA_6AN_15m	ns	>0.9999
hypoxanthine:PMA_DPI_15m vs. UTP:PMA_DPI_15m	ns	>0.9999
hypoxanthine:PMA_DPI_15m vs. UTP:PMA_AA_15m	ns	>0.9999
hypoxanthine:PMA_DPI_15m vs. UTP:PMA_IAA_15m	ns	0.6614
hypoxanthine:PMA_AA_15m vs. hypoxanthine:PMA_IAA_15m	****	<0.0001
hypoxanthine:PMA_AA_15m vs. IMP:PMA_15m	ns	>0.9999
hypoxanthine:PMA_AA_15m vs. IMP:PMA_2DG_15m	ns	>0.9999
hypoxanthine:PMA_AA_15m vs. IMP:PMA_6AN_15m	ns	0.9962
hypoxanthine:PMA_AA_15m vs. IMP:PMA_DPI_15m	ns	>0.9999
hypoxanthine:PMA_AA_15m vs. IMP:PMA_AA_15m	ns	0.8852
hypoxanthine:PMA_AA_15m vs. IMP:PMA_IAA_15m	ns	>0.9999
hypoxanthine:PMA_AA_15m vs. GMP:PMA_15m	ns	>0.9999
hypoxanthine:PMA_AA_15m vs. GMP:PMA_2DG_15m	ns	0.9257
hypoxanthine:PMA_AA_15m vs. GMP:PMA_6AN_15m	ns	0.6219
hypoxanthine:PMA_AA_15m vs. GMP:PMA_DPI_15m	ns	>0.9999
hypoxanthine:PMA_AA_15m vs. GMP:PMA_AA_15m	ns	0.172
hypoxanthine:PMA_AA_15m vs. GMP:PMA_IAA_15m	ns	>0.9999
hypoxanthine:PMA_AA_15m vs. GDP:PMA_15m	ns	>0.9999
hypoxanthine:PMA_AA_15m vs. GDP:PMA_2DG_15m	ns	0.9502
hypoxanthine:PMA_AA_15m vs. GDP:PMA_6AN_15m	ns	0.6835
hypoxanthine:PMA_AA_15m vs. GDP:PMA_DPI_15m	ns	>0.9999
hypoxanthine:PMA_AA_15m vs. GDP:PMA_AA_15m	ns	0.2138
hypoxanthine:PMA_AA_15m vs. GDP:PMA_IAA_15m	ns	>0.9999
hypoxanthine:PMA_AA_15m vs. GTP:PMA_15m	ns	0.954
hypoxanthine:PMA_AA_15m vs. GTP:PMA_2DG_15m	ns	>0.9999
hypoxanthine:PMA_AA_15m vs. GTP:PMA_6AN_15m	ns	>0.9999
hypoxanthine:PMA_AA_15m vs. GTP:PMA_DPI_15m	ns	0.9995
hypoxanthine:PMA_AA_15m vs. GTP:PMA_AA_15m	ns	>0.9999
hypoxanthine:PMA_AA_15m vs. GTP:PMA_IAA_15m	**	0.005
hypoxanthine:PMA_AA_15m vs. Guanosine:PMA_15m	ns	>0.9999
hypoxanthine:PMA_AA_15m vs. Guanosine:PMA_2DG_15m	ns	>0.9999
hypoxanthine:PMA_AA_15m vs. Guanosine:PMA_6AN_15m	ns	0.9998
hypoxanthine:PMA_AA_15m vs. Guanosine:PMA_DPI_15m	ns	>0.9999
hypoxanthine:PMA_AA_15m vs. Guanosine:PMA_AA_15m	ns	0.9743
hypoxanthine:PMA_AA_15m vs. Guanosine:PMA_IAA_15m	ns	>0.9999

hypoxanthine:PMA_AA_15m vs. Guanine:PMA_15m	ns	>0.9999
hypoxanthine:PMA_AA_15m vs. Guanine:PMA_2DG_15m	ns	>0.9999
hypoxanthine:PMA_AA_15m vs. Guanine:PMA_6AN_15m	ns	>0.9999
hypoxanthine:PMA_AA_15m vs. Guanine:PMA_DPI_15m	ns	>0.9999
hypoxanthine:PMA_AA_15m vs. Guanine:PMA_AA_15m	ns	>0.9999
hypoxanthine:PMA_AA_15m vs. Guanine:PMA_IAA_15m	ns	0.1149
hypoxanthine:PMA_AA_15m vs. Aspartic acid:PMA_15m	ns	>0.9999
hypoxanthine:PMA_AA_15m vs. Aspartic acid:PMA_2DG_15m	ns	>0.9999
hypoxanthine:PMA_AA_15m vs. Aspartic acid:PMA_6AN_15m	ns	>0.9999
hypoxanthine:PMA_AA_15m vs. Aspartic acid:PMA_DPI_15m	ns	>0.9999
hypoxanthine:PMA_AA_15m vs. Aspartic acid:PMA_AA_15m	ns	>0.9999
hypoxanthine:PMA_AA_15m vs. Aspartic acid:PMA_IAA_15m	ns	0.242
hypoxanthine:PMA_AA_15m vs. AMP:PMA_15m	ns	>0.9999
hypoxanthine:PMA_AA_15m vs. AMP:PMA_2DG_15m	ns	>0.9999
hypoxanthine:PMA_AA_15m vs. AMP:PMA_6AN_15m	ns	>0.9999
hypoxanthine:PMA_AA_15m vs. AMP:PMA_DPI_15m	ns	>0.9999
hypoxanthine:PMA_AA_15m vs. AMP:PMA_AA_15m	ns	0.9998
hypoxanthine:PMA_AA_15m vs. AMP:PMA_IAA_15m	ns	0.9972
hypoxanthine:PMA_AA_15m vs. ADP:PMA_15m	ns	>0.9999
hypoxanthine:PMA_AA_15m vs. ADP:PMA_2DG_15m	ns	>0.9999
hypoxanthine:PMA_AA_15m vs. ADP:PMA_6AN_15m	ns	>0.9999
hypoxanthine:PMA_AA_15m vs. ADP:PMA_DPI_15m	ns	>0.9999
hypoxanthine:PMA_AA_15m vs. ADP:PMA_AA_15m	ns	>0.9999
hypoxanthine:PMA_AA_15m vs. ADP:PMA_IAA_15m	ns	0.9536
hypoxanthine:PMA_AA_15m vs. ATP:PMA_15m	ns	0.7762
hypoxanthine:PMA_AA_15m vs. ATP:PMA_2DG_15m	ns	>0.9999
hypoxanthine:PMA_AA_15m vs. ATP:PMA_6AN_15m	ns	>0.9999
hypoxanthine:PMA_AA_15m vs. ATP:PMA_DPI_15m	ns	0.9824
hypoxanthine:PMA_AA_15m vs. ATP:PMA_AA_15m	ns	>0.9999
hypoxanthine:PMA_AA_15m vs. ATP:PMA_IAA_15m	**	0.0012
hypoxanthine:PMA_AA_15m vs. Xanthine:PMA_15m	ns	>0.9999
hypoxanthine:PMA_AA_15m vs. Xanthine:PMA_2DG_15m	ns	>0.9999
hypoxanthine:PMA_AA_15m vs. Xanthine:PMA_6AN_15m	ns	>0.9999
hypoxanthine:PMA_AA_15m vs. Xanthine:PMA_DPI_15m	ns	>0.9999
hypoxanthine:PMA_AA_15m vs. Xanthine:PMA_AA_15m	ns	>0.9999
hypoxanthine:PMA_AA_15m vs. CMP:PMA_15m	ns	>0.9999
hypoxanthine:PMA_AA_15m vs. CMP:PMA_2DG_15m	ns	>0.9999
hypoxanthine:PMA_AA_15m vs. CMP:PMA_6AN_15m	ns	>0.9999
hypoxanthine:PMA_AA_15m vs. CMP:PMA_DPI_15m	ns	>0.9999
hypoxanthine:PMA_AA_15m vs. CMP:PMA_AA_15m	ns	0.9987
hypoxanthine:PMA_AA_15m vs. CMP:PMA_IAA_15m	ns	0.9994
hypoxanthine:PMA_AA_15m vs. CDP:PMA_15m	ns	>0.9999
hypoxanthine:PMA_AA_15m vs. CDP:PMA_2DG_15m	ns	>0.9999
hypoxanthine:PMA_AA_15m vs. CDP:PMA_6AN_15m	ns	0.9945
hypoxanthine:PMA_AA_15m vs. CDP:PMA_DPI_15m	ns	>0.9999
hypoxanthine:PMA_AA_15m vs. CDP:PMA_AA_15m	ns	0.861
hypoxanthine:PMA_AA_15m vs. CDP:PMA_IAA_15m	ns	>0.9999

hypoxanthine:PMA_AA_15m vs. CTP:PMA_15m	ns	0.995
hypoxanthine:PMA_AA_15m vs. CTP:PMA_2DG_15m	ns	>0.9999
hypoxanthine:PMA_AA_15m vs. CTP:PMA_6AN_15m	ns	>0.9999
hypoxanthine:PMA_AA_15m vs. CTP:PMA_DPI_15m	ns	>0.9999
hypoxanthine:PMA_AA_15m vs. CTP:PMA_AA_15m	ns	>0.9999
hypoxanthine:PMA_AA_15m vs. CTP:PMA_IAA_15m	*	0.0153
hypoxanthine:PMA_AA_15m vs. Uridine:PMA_15m	ns	>0.9999
hypoxanthine:PMA_AA_15m vs. Uridine:PMA_2DG_15m	ns	>0.9999
hypoxanthine:PMA_AA_15m vs. Uridine:PMA_6AN_15m	ns	>0.9999
hypoxanthine:PMA_AA_15m vs. Uridine:PMA_DPI_15m	ns	>0.9999
hypoxanthine:PMA_AA_15m vs. Uridine:PMA_AA_15m	ns	0.988
hypoxanthine:PMA_AA_15m vs. Uridine:PMA_IAA_15m	ns	>0.9999
hypoxanthine:PMA_AA_15m vs. UMP:PMA_15m	ns	>0.9999
hypoxanthine:PMA_AA_15m vs. UMP:PMA_2DG_15m	ns	0.9971
hypoxanthine:PMA_AA_15m vs. UMP:PMA_6AN_15m	ns	0.9169
hypoxanthine:PMA_AA_15m vs. UMP:PMA_DPI_15m	ns	>0.9999
hypoxanthine:PMA_AA_15m vs. UMP:PMA_AA_15m	ns	0.5138
hypoxanthine:PMA_AA_15m vs. UMP:PMA_IAA_15m	ns	>0.9999
hypoxanthine:PMA_AA_15m vs. UDP:PMA_15m	ns	>0.9999
hypoxanthine:PMA_AA_15m vs. UDP:PMA_2DG_15m	ns	0.9976
hypoxanthine:PMA_AA_15m vs. UDP:PMA_6AN_15m	ns	0.9248
hypoxanthine:PMA_AA_15m vs. UDP:PMA_DPI_15m	ns	>0.9999
hypoxanthine:PMA_AA_15m vs. UDP:PMA_AA_15m	ns	0.5332
hypoxanthine:PMA_AA_15m vs. UDP:PMA_IAA_15m	ns	>0.9999
hypoxanthine:PMA_AA_15m vs. UTP:PMA_15m	ns	0.9752
hypoxanthine:PMA_AA_15m vs. UTP:PMA_2DG_15m	ns	>0.9999
hypoxanthine:PMA_AA_15m vs. UTP:PMA_6AN_15m	ns	>0.9999
hypoxanthine:PMA_AA_15m vs. UTP:PMA_DPI_15m	ns	0.9999
hypoxanthine:PMA_AA_15m vs. UTP:PMA_AA_15m	ns	>0.9999
hypoxanthine:PMA_AA_15m vs. UTP:PMA_IAA_15m	**	0.0073
hypoxanthine:PMA_IAA_15m vs. IMP:PMA_15m	*	0.0102
hypoxanthine:PMA_IAA_15m vs. IMP:PMA_2DG_15m	****	<0.0001
hypoxanthine:PMA_IAA_15m vs. IMP:PMA_6AN_15m	****	<0.0001
hypoxanthine:PMA_IAA_15m vs. IMP:PMA_DPI_15m	**	0.0014
hypoxanthine:PMA_IAA_15m vs. IMP:PMA_AA_15m	****	<0.0001
hypoxanthine:PMA_IAA_15m vs. IMP:PMA_IAA_15m	ns	0.8852
hypoxanthine:PMA_IAA_15m vs. GMP:PMA_15m	***	0.0003
hypoxanthine:PMA_IAA_15m vs. GMP:PMA_2DG_15m	****	<0.0001
hypoxanthine:PMA_IAA_15m vs. GMP:PMA_6AN_15m	****	<0.0001
hypoxanthine:PMA_IAA_15m vs. GMP:PMA_DPI_15m	****	<0.0001
hypoxanthine:PMA_IAA_15m vs. GMP:PMA_AA_15m	****	<0.0001
hypoxanthine:PMA_IAA_15m vs. GMP:PMA_IAA_15m	ns	0.172
hypoxanthine:PMA_IAA_15m vs. GDP:PMA_15m	***	0.0004
hypoxanthine:PMA_IAA_15m vs. GDP:PMA_2DG_15m	****	<0.0001
hypoxanthine:PMA_IAA_15m vs. GDP:PMA_6AN_15m	****	<0.0001
hypoxanthine:PMA_IAA_15m vs. GDP:PMA_DPI_15m	****	<0.0001
hypoxanthine:PMA_IAA_15m vs. GDP:PMA_AA_15m	****	<0.0001

hypoxanthine:PMA_IAA_15m vs. GDP:PMA_IAA_15m	ns	0.2138
hypoxanthine:PMA_IAA_15m vs. GTP:PMA_15m	ns	>0.9999
hypoxanthine:PMA_IAA_15m vs. GTP:PMA_2DG_15m	ns	0.8027
hypoxanthine:PMA_IAA_15m vs. GTP:PMA_6AN_15m	ns	0.4284
hypoxanthine:PMA_IAA_15m vs. GTP:PMA_DPI_15m	ns	>0.9999
hypoxanthine:PMA_IAA_15m vs. GTP:PMA_AA_15m	ns	0.3222
hypoxanthine:PMA_IAA_15m vs. GTP:PMA_IAA_15m	ns	>0.9999
hypoxanthine:PMA_IAA_15m vs. Guanosine:PMA_15m	*	0.0243
hypoxanthine:PMA_IAA_15m vs. Guanosine:PMA_2DG_15m	****	<0.0001
hypoxanthine:PMA_IAA_15m vs. Guanosine:PMA_6AN_15m	****	<0.0001
hypoxanthine:PMA_IAA_15m vs. Guanosine:PMA_DPI_15m	**	0.0036
hypoxanthine:PMA_IAA_15m vs. Guanosine:PMA_AA_15m	****	<0.0001
hypoxanthine:PMA_IAA_15m vs. Guanosine:PMA_IAA_15m	ns	0.9743
hypoxanthine:PMA_IAA_15m vs. Guanine:PMA_15m	ns	0.9953
hypoxanthine:PMA_IAA_15m vs. Guanine:PMA_2DG_15m	ns	0.1536
hypoxanthine:PMA_IAA_15m vs. Guanine:PMA_6AN_15m	*	0.037
hypoxanthine:PMA_IAA_15m vs. Guanine:PMA_DPI_15m	ns	0.8763
hypoxanthine:PMA_IAA_15m vs. Guanine:PMA_AA_15m	*	0.0227
hypoxanthine:PMA_IAA_15m vs. Guanine:PMA_IAA_15m	ns	>0.9999
hypoxanthine:PMA_IAA_15m vs. Aspartic acid:PMA_15m	ns	0.9602
hypoxanthine:PMA_IAA_15m vs. Aspartic acid:PMA_2DG_15m	ns	0.0673
hypoxanthine:PMA_IAA_15m vs. Aspartic acid:PMA_6AN_15m	*	0.0137
hypoxanthine:PMA_IAA_15m vs. Aspartic acid:PMA_DPI_15m	ns	0.684
hypoxanthine:PMA_IAA_15m vs. Aspartic acid:PMA_AA_15m	**	0.0081
hypoxanthine:PMA_IAA_15m vs. Aspartic acid:PMA_IAA_15m	ns	>0.9999
hypoxanthine:PMA_IAA_15m vs. AMP:PMA_15m	ns	0.0978
hypoxanthine:PMA_IAA_15m vs. AMP:PMA_2DG_15m	***	0.0003
hypoxanthine:PMA_IAA_15m vs. AMP:PMA_6AN_15m	****	<0.0001
hypoxanthine:PMA_IAA_15m vs. AMP:PMA_DPI_15m	*	0.0186
hypoxanthine:PMA_IAA_15m vs. AMP:PMA_AA_15m	****	<0.0001
hypoxanthine:PMA_IAA_15m vs. AMP:PMA_IAA_15m	ns	0.9998
hypoxanthine:PMA_IAA_15m vs. ADP:PMA_15m	ns	0.2571
hypoxanthine:PMA_IAA_15m vs. ADP:PMA_2DG_15m	**	0.0014
hypoxanthine:PMA_IAA_15m vs. ADP:PMA_6AN_15m	***	0.0002
hypoxanthine:PMA_IAA_15m vs. ADP:PMA_DPI_15m	ns	0.0633
hypoxanthine:PMA_IAA_15m vs. ADP:PMA_AA_15m	****	<0.0001
hypoxanthine:PMA_IAA_15m vs. ADP:PMA_IAA_15m	ns	>0.9999
hypoxanthine:PMA_IAA_15m vs. ATP:PMA_15m	ns	>0.9999
hypoxanthine:PMA_IAA_15m vs. ATP:PMA_2DG_15m	ns	0.9631
hypoxanthine:PMA_IAA_15m vs. ATP:PMA_6AN_15m	ns	0.7252
hypoxanthine:PMA_IAA_15m vs. ATP:PMA_DPI_15m	ns	>0.9999
hypoxanthine:PMA_IAA_15m vs. ATP:PMA_AA_15m	ns	0.6095
hypoxanthine:PMA_IAA_15m vs. ATP:PMA_IAA_15m	ns	>0.9999
hypoxanthine:PMA_IAA_15m vs. Xanthine:PMA_15m	ns	0.9996
hypoxanthine:PMA_IAA_15m vs. Xanthine:PMA_2DG_15m	ns	0.2819
hypoxanthine:PMA_IAA_15m vs. Xanthine:PMA_6AN_15m	ns	0.0827
hypoxanthine:PMA_IAA_15m vs. Xanthine:PMA_DPI_15m	ns	0.9611

hypoxanthine:PMA_IAA_15m vs. Xanthine:PMA_AA_15m	ns	0.0534
hypoxanthine:PMA_IAA_15m vs. CMP:PMA_15m	ns	0.0653
hypoxanthine:PMA_IAA_15m vs. CMP:PMA_2DG_15m	***	0.0002
hypoxanthine:PMA_IAA_15m vs. CMP:PMA_6AN_15m	****	<0.0001
hypoxanthine:PMA_IAA_15m vs. CMP:PMA_DPI_15m	*	0.0115
hypoxanthine:PMA_IAA_15m vs. CMP:PMA_AA_15m	****	<0.0001
hypoxanthine:PMA_IAA_15m vs. CMP:PMA_IAA_15m	ns	0.9987
hypoxanthine:PMA_IAA_15m vs. CDP:PMA_15m	**	0.0087
hypoxanthine:PMA_IAA_15m vs. CDP:PMA_2DG_15m	****	<0.0001
hypoxanthine:PMA_IAA_15m vs. CDP:PMA_6AN_15m	****	<0.0001
hypoxanthine:PMA_IAA_15m vs. CDP:PMA_DPI_15m	**	0.0012
hypoxanthine:PMA_IAA_15m vs. CDP:PMA_AA_15m	****	<0.0001
hypoxanthine:PMA_IAA_15m vs. CDP:PMA_IAA_15m	ns	0.861
hypoxanthine:PMA_IAA_15m vs. CTP:PMA_15m	ns	>0.9999
hypoxanthine:PMA_IAA_15m vs. CTP:PMA_2DG_15m	ns	0.5675
hypoxanthine:PMA_IAA_15m vs. CTP:PMA_6AN_15m	ns	0.2247
hypoxanthine:PMA_IAA_15m vs. CTP:PMA_DPI_15m	ns	0.9984
hypoxanthine:PMA_IAA_15m vs. CTP:PMA_AA_15m	ns	0.1559
hypoxanthine:PMA_IAA_15m vs. CTP:PMA_IAA_15m	ns	>0.9999
hypoxanthine:PMA_IAA_15m vs. Uridine:PMA_15m	*	0.0333
hypoxanthine:PMA_IAA_15m vs. Uridine:PMA_2DG_15m	****	<0.0001
hypoxanthine:PMA_IAA_15m vs. Uridine:PMA_6AN_15m	****	<0.0001
hypoxanthine:PMA_IAA_15m vs. Uridine:PMA_DPI_15m	**	0.0052
hypoxanthine:PMA_IAA_15m vs. Uridine:PMA_AA_15m	****	<0.0001
hypoxanthine:PMA_IAA_15m vs. Uridine:PMA_IAA_15m	ns	0.988
hypoxanthine:PMA_IAA_15m vs. UMP:PMA_15m	**	0.0018
hypoxanthine:PMA_IAA_15m vs. UMP:PMA_2DG_15m	****	<0.0001
hypoxanthine:PMA_IAA_15m vs. UMP:PMA_6AN_15m	****	<0.0001
hypoxanthine:PMA_IAA_15m vs. UMP:PMA_DPI_15m	***	0.0002
hypoxanthine:PMA_IAA_15m vs. UMP:PMA_AA_15m	****	<0.0001
hypoxanthine:PMA_IAA_15m vs. UMP:PMA_IAA_15m	ns	0.5138
hypoxanthine:PMA_IAA_15m vs. UDP:PMA_15m	**	0.002
hypoxanthine:PMA_IAA_15m vs. UDP:PMA_2DG_15m	****	<0.0001
hypoxanthine:PMA_IAA_15m vs. UDP:PMA_6AN_15m	****	<0.0001
hypoxanthine:PMA_IAA_15m vs. UDP:PMA_DPI_15m	***	0.0002
hypoxanthine:PMA_IAA_15m vs. UDP:PMA_AA_15m	****	<0.0001
hypoxanthine:PMA_IAA_15m vs. UDP:PMA_IAA_15m	ns	0.5332
hypoxanthine:PMA_IAA_15m vs. UTP:PMA_15m	ns	>0.9999
hypoxanthine:PMA_IAA_15m vs. UTP:PMA_2DG_15m	ns	0.7319
hypoxanthine:PMA_IAA_15m vs. UTP:PMA_6AN_15m	ns	0.3534
hypoxanthine:PMA_IAA_15m vs. UTP:PMA_DPI_15m	ns	0.9999
hypoxanthine:PMA_IAA_15m vs. UTP:PMA_AA_15m	ns	0.2584
hypoxanthine:PMA_IAA_15m vs. UTP:PMA_IAA_15m	ns	>0.9999
IMP:PMA_15m vs. IMP:PMA_2DG_15m	ns	0.7475
IMP:PMA_15m vs. IMP:PMA_6AN_15m	ns	0.1037
IMP:PMA_15m vs. IMP:PMA_DPI_15m	ns	>0.9999
IMP:PMA_15m vs. IMP:PMA_AA_15m	*	0.0409

IMP:PMA_15m vs. IMP:PMA_IAA_15m	*	0.0236
IMP:PMA_15m vs. GMP:PMA_15m	ns	>0.9999
IMP:PMA_15m vs. GMP:PMA_2DG_15m	ns	0.9978
IMP:PMA_15m vs. GMP:PMA_6AN_15m	ns	0.9283
IMP:PMA_15m vs. GMP:PMA_DPI_15m	ns	>0.9999
IMP:PMA_15m vs. GMP:PMA_AA_15m	ns	0.863
IMP:PMA_15m vs. GMP:PMA_IAA_15m	ns	>0.9999
IMP:PMA_15m vs. GDP:PMA_15m	ns	>0.9999
IMP:PMA_15m vs. GDP:PMA_2DG_15m	ns	0.9991
IMP:PMA_15m vs. GDP:PMA_6AN_15m	ns	0.9521
IMP:PMA_15m vs. GDP:PMA_DPI_15m	ns	>0.9999
IMP:PMA_15m vs. GDP:PMA_AA_15m	ns	0.9005
IMP:PMA_15m vs. GDP:PMA_IAA_15m	ns	>0.9999
IMP:PMA_15m vs. GTP:PMA_15m	ns	0.3178
IMP:PMA_15m vs. GTP:PMA_2DG_15m	ns	>0.9999
IMP:PMA_15m vs. GTP:PMA_6AN_15m	ns	>0.9999
IMP:PMA_15m vs. GTP:PMA_DPI_15m	ns	0.9621
IMP:PMA_15m vs. GTP:PMA_AA_15m	ns	>0.9999
IMP:PMA_15m vs. GTP:PMA_IAA_15m	***	0.0007
IMP:PMA_15m vs. Guanosine:PMA_15m	ns	>0.9999
IMP:PMA_15m vs. Guanosine:PMA_2DG_15m	ns	>0.9999
IMP:PMA_15m vs. Guanosine:PMA_6AN_15m	ns	>0.9999
IMP:PMA_15m vs. Guanosine:PMA_DPI_15m	ns	>0.9999
IMP:PMA_15m vs. Guanosine:PMA_AA_15m	ns	>0.9999
IMP:PMA_15m vs. Guanosine:PMA_IAA_15m	ns	0.9958
IMP:PMA_15m vs. Guanine:PMA_15m	ns	0.9766
IMP:PMA_15m vs. Guanine:PMA_2DG_15m	ns	>0.9999
IMP:PMA_15m vs. Guanine:PMA_6AN_15m	ns	>0.9999
IMP:PMA_15m vs. Guanine:PMA_DPI_15m	ns	>0.9999
IMP:PMA_15m vs. Guanine:PMA_AA_15m	ns	>0.9999
IMP:PMA_15m vs. Guanine:PMA_IAA_15m	*	0.0253
IMP:PMA_15m vs. Aspartic acid:PMA_15m	ns	0.9986
IMP:PMA_15m vs. Aspartic acid:PMA_2DG_15m	ns	>0.9999
IMP:PMA_15m vs. Aspartic acid:PMA_6AN_15m	ns	>0.9999
IMP:PMA_15m vs. Aspartic acid:PMA_DPI_15m	ns	>0.9999
IMP:PMA_15m vs. Aspartic acid:PMA_AA_15m	ns	>0.9999
IMP:PMA_15m vs. Aspartic acid:PMA_IAA_15m	ns	0.0646
IMP:PMA_15m vs. AMP:PMA_15m	ns	>0.9999
IMP:PMA_15m vs. AMP:PMA_2DG_15m	ns	>0.9999
IMP:PMA_15m vs. AMP:PMA_6AN_15m	ns	>0.9999
IMP:PMA_15m vs. AMP:PMA_DPI_15m	ns	>0.9999
IMP:PMA_15m vs. AMP:PMA_AA_15m	ns	>0.9999
IMP:PMA_15m vs. AMP:PMA_IAA_15m	ns	0.9167
IMP:PMA_15m vs. ADP:PMA_15m	ns	>0.9999
IMP:PMA_15m vs. ADP:PMA_2DG_15m	ns	>0.9999
IMP:PMA_15m vs. ADP:PMA_6AN_15m	ns	>0.9999
IMP:PMA_15m vs. ADP:PMA_DPI_15m	ns	>0.9999

IMP:PMA_15m vs. ADP:PMA_AA_15m	ns	>0.9999
IMP:PMA_15m vs. ADP:PMA_IAA_15m	ns	0.6893
IMP:PMA_15m vs. ATP:PMA_15m	ns	0.1118
IMP:PMA_15m vs. ATP:PMA_2DG_15m	ns	>0.9999
IMP:PMA_15m vs. ATP:PMA_6AN_15m	ns	>0.9999
IMP:PMA_15m vs. ATP:PMA_DPI_15m	ns	0.7987
IMP:PMA_15m vs. ATP:PMA_AA_15m	ns	>0.9999
IMP:PMA_15m vs. ATP:PMA_IAA_15m	***	0.0002
IMP:PMA_15m vs. Xanthine:PMA_15m	ns	0.9639
IMP:PMA_15m vs. Xanthine:PMA_2DG_15m	ns	>0.9999
IMP:PMA_15m vs. Xanthine:PMA_6AN_15m	ns	>0.9999
IMP:PMA_15m vs. Xanthine:PMA_DPI_15m	ns	>0.9999
IMP:PMA_15m vs. Xanthine:PMA_AA_15m	ns	>0.9999
IMP:PMA_15m vs. CMP:PMA_15m	ns	>0.9999
IMP:PMA_15m vs. CMP:PMA_2DG_15m	ns	>0.9999
IMP:PMA_15m vs. CMP:PMA_6AN_15m	ns	>0.9999
IMP:PMA_15m vs. CMP:PMA_DPI_15m	ns	>0.9999
IMP:PMA_15m vs. CMP:PMA_AA_15m	ns	>0.9999
IMP:PMA_15m vs. CMP:PMA_IAA_15m	ns	0.9593
IMP:PMA_15m vs. CDP:PMA_15m	ns	>0.9999
IMP:PMA_15m vs. CDP:PMA_2DG_15m	ns	>0.9999
IMP:PMA_15m vs. CDP:PMA_6AN_15m	ns	>0.9999
IMP:PMA_15m vs. CDP:PMA_DPI_15m	ns	>0.9999
IMP:PMA_15m vs. CDP:PMA_AA_15m	ns	0.9999
IMP:PMA_15m vs. CDP:PMA_IAA_15m	ns	0.9998
IMP:PMA_15m vs. CTP:PMA_15m	ns	0.5892
IMP:PMA_15m vs. CTP:PMA_2DG_15m	ns	>0.9999
IMP:PMA_15m vs. CTP:PMA_6AN_15m	ns	>0.9999
IMP:PMA_15m vs. CTP:PMA_DPI_15m	ns	0.9963
IMP:PMA_15m vs. CTP:PMA_AA_15m	ns	>0.9999
IMP:PMA_15m vs. CTP:PMA_IAA_15m	**	0.0025
IMP:PMA_15m vs. Uridine:PMA_15m	ns	>0.9999
IMP:PMA_15m vs. Uridine:PMA_2DG_15m	ns	>0.9999
IMP:PMA_15m vs. Uridine:PMA_6AN_15m	ns	>0.9999
IMP:PMA_15m vs. Uridine:PMA_DPI_15m	ns	>0.9999
IMP:PMA_15m vs. Uridine:PMA_AA_15m	ns	>0.9999
IMP:PMA_15m vs. Uridine:PMA_IAA_15m	ns	0.9905
IMP:PMA_15m vs. UMP:PMA_15m	ns	>0.9999
IMP:PMA_15m vs. UMP:PMA_2DG_15m	ns	>0.9999
IMP:PMA_15m vs. UMP:PMA_6AN_15m	ns	0.9973
IMP:PMA_15m vs. UMP:PMA_DPI_15m	ns	>0.9999
IMP:PMA_15m vs. UMP:PMA_AA_15m	ns	0.9901
IMP:PMA_15m vs. UMP:PMA_IAA_15m	ns	>0.9999
IMP:PMA_15m vs. UDP:PMA_15m	ns	>0.9999
IMP:PMA_15m vs. UDP:PMA_2DG_15m	ns	>0.9999
IMP:PMA_15m vs. UDP:PMA_6AN_15m	ns	0.9978
IMP:PMA_15m vs. UDP:PMA_DPI_15m	ns	>0.9999

IMP:PMA_15m vs. UDP:PMA_AA_15m	ns	0.9916
IMP:PMA_15m vs. UDP:PMA_IAA_15m	ns	>0.9999
IMP:PMA_15m vs. UTP:PMA_15m	ns	0.4001
IMP:PMA_15m vs. UTP:PMA_2DG_15m	ns	>0.9999
IMP:PMA_15m vs. UTP:PMA_6AN_15m	ns	>0.9999
IMP:PMA_15m vs. UTP:PMA_DPI_15m	ns	0.9802
IMP:PMA_15m vs. UTP:PMA_AA_15m	ns	>0.9999
IMP:PMA_15m vs. UTP:PMA_IAA_15m	**	0.0011
IMP:PMA_2DG_15m vs. IMP:PMA_6AN_15m	ns	>0.9999
IMP:PMA_2DG_15m vs. IMP:PMA_DPI_15m	ns	>0.9999
IMP:PMA_2DG_15m vs. IMP:PMA_AA_15m	ns	>0.9999
IMP:PMA_2DG_15m vs. IMP:PMA_IAA_15m	****	<0.0001
IMP:PMA_2DG_15m vs. GMP:PMA_15m	ns	>0.9999
IMP:PMA_2DG_15m vs. GMP:PMA_2DG_15m	ns	>0.9999
IMP:PMA_2DG_15m vs. GMP:PMA_6AN_15m	ns	>0.9999
IMP:PMA_2DG_15m vs. GMP:PMA_DPI_15m	ns	>0.9999
IMP:PMA_2DG_15m vs. GMP:PMA_AA_15m	ns	>0.9999
IMP:PMA_2DG_15m vs. GMP:PMA_IAA_15m	ns	0.9216
IMP:PMA_2DG_15m vs. GDP:PMA_15m	ns	>0.9999
IMP:PMA_2DG_15m vs. GDP:PMA_2DG_15m	ns	>0.9999
IMP:PMA_2DG_15m vs. GDP:PMA_6AN_15m	ns	>0.9999
IMP:PMA_2DG_15m vs. GDP:PMA_DPI_15m	ns	>0.9999
IMP:PMA_2DG_15m vs. GDP:PMA_AA_15m	ns	>0.9999
IMP:PMA_2DG_15m vs. GDP:PMA_IAA_15m	ns	0.8892
IMP:PMA_2DG_15m vs. GTP:PMA_15m	*	0.0119
IMP:PMA_2DG_15m vs. GTP:PMA_2DG_15m	ns	0.3178
IMP:PMA_2DG_15m vs. GTP:PMA_6AN_15m	ns	0.952
IMP:PMA_2DG_15m vs. GTP:PMA_DPI_15m	ns	0.0679
IMP:PMA_2DG_15m vs. GTP:PMA_AA_15m	ns	0.9808
IMP:PMA_2DG_15m vs. GTP:PMA_IAA_15m	****	<0.0001
IMP:PMA_2DG_15m vs. Guanosine:PMA_15m	ns	>0.9999
IMP:PMA_2DG_15m vs. Guanosine:PMA_2DG_15m	ns	>0.9999
IMP:PMA_2DG_15m vs. Guanosine:PMA_6AN_15m	ns	>0.9999
IMP:PMA_2DG_15m vs. Guanosine:PMA_DPI_15m	ns	>0.9999
IMP:PMA_2DG_15m vs. Guanosine:PMA_AA_15m	ns	>0.9999
IMP:PMA_2DG_15m vs. Guanosine:PMA_IAA_15m	ns	0.1587
IMP:PMA_2DG_15m vs. Guanine:PMA_15m	ns	0.2144
IMP:PMA_2DG_15m vs. Guanine:PMA_2DG_15m	ns	0.9766
IMP:PMA_2DG_15m vs. Guanine:PMA_6AN_15m	ns	>0.9999
IMP:PMA_2DG_15m vs. Guanine:PMA_DPI_15m	ns	0.5863
IMP:PMA_2DG_15m vs. Guanine:PMA_AA_15m	ns	>0.9999
IMP:PMA_2DG_15m vs. Guanine:PMA_IAA_15m	****	<0.0001
IMP:PMA_2DG_15m vs. Aspartic acid:PMA_15m	ns	0.4013
IMP:PMA_2DG_15m vs. Aspartic acid:PMA_2DG_15m	ns	0.9986
IMP:PMA_2DG_15m vs. Aspartic acid:PMA_6AN_15m	ns	>0.9999
IMP:PMA_2DG_15m vs. Aspartic acid:PMA_DPI_15m	ns	0.8072
IMP:PMA_2DG_15m vs. Aspartic acid:PMA_AA_15m	ns	>0.9999

IMP:PMA_2DG_15m vs. Aspartic acid:PMA_IAA_15m	***	0.0002
IMP:PMA_2DG_15m vs. AMP:PMA_15m	ns	0.9998
IMP:PMA_2DG_15m vs. AMP:PMA_2DG_15m	ns	>0.9999
IMP:PMA_2DG_15m vs. AMP:PMA_6AN_15m	ns	>0.9999
IMP:PMA_2DG_15m vs. AMP:PMA_DPI_15m	ns	>0.9999
IMP:PMA_2DG_15m vs. AMP:PMA_AA_15m	ns	>0.9999
IMP:PMA_2DG_15m vs. AMP:PMA_IAA_15m	*	0.0435
IMP:PMA_2DG_15m vs. ADP:PMA_15m	ns	0.9912
IMP:PMA_2DG_15m vs. ADP:PMA_2DG_15m	ns	>0.9999
IMP:PMA_2DG_15m vs. ADP:PMA_6AN_15m	ns	>0.9999
IMP:PMA_2DG_15m vs. ADP:PMA_DPI_15m	ns	>0.9999
IMP:PMA_2DG_15m vs. ADP:PMA_AA_15m	ns	>0.9999
IMP:PMA_2DG_15m vs. ADP:PMA_IAA_15m	*	0.0122
IMP:PMA_2DG_15m vs. ATP:PMA_15m	**	0.003
IMP:PMA_2DG_15m vs. ATP:PMA_2DG_15m	ns	0.1118
IMP:PMA_2DG_15m vs. ATP:PMA_6AN_15m	ns	0.7711
IMP:PMA_2DG_15m vs. ATP:PMA_DPI_15m	*	0.0203
IMP:PMA_2DG_15m vs. ATP:PMA_AA_15m	ns	0.8623
IMP:PMA_2DG_15m vs. ATP:PMA_IAA_15m	****	<0.0001
IMP:PMA_2DG_15m vs. Xanthine:PMA_15m	ns	0.2006
IMP:PMA_2DG_15m vs. Xanthine:PMA_2DG_15m	ns	0.9639
IMP:PMA_2DG_15m vs. Xanthine:PMA_6AN_15m	ns	>0.9999
IMP:PMA_2DG_15m vs. Xanthine:PMA_DPI_15m	ns	0.5475
IMP:PMA_2DG_15m vs. Xanthine:PMA_AA_15m	ns	>0.9999
IMP:PMA_2DG_15m vs. CMP:PMA_15m	ns	>0.9999
IMP:PMA_2DG_15m vs. CMP:PMA_2DG_15m	ns	>0.9999
IMP:PMA_2DG_15m vs. CMP:PMA_6AN_15m	ns	>0.9999
IMP:PMA_2DG_15m vs. CMP:PMA_DPI_15m	ns	>0.9999
IMP:PMA_2DG_15m vs. CMP:PMA_AA_15m	ns	>0.9999
IMP:PMA_2DG_15m vs. CMP:PMA_IAA_15m	ns	0.0666
IMP:PMA_2DG_15m vs. CDP:PMA_15m	ns	>0.9999
IMP:PMA_2DG_15m vs. CDP:PMA_2DG_15m	ns	>0.9999
IMP:PMA_2DG_15m vs. CDP:PMA_6AN_15m	ns	>0.9999
IMP:PMA_2DG_15m vs. CDP:PMA_DPI_15m	ns	>0.9999
IMP:PMA_2DG_15m vs. CDP:PMA_AA_15m	ns	>0.9999
IMP:PMA_2DG_15m vs. CDP:PMA_IAA_15m	ns	0.3141
IMP:PMA_2DG_15m vs. CTP:PMA_15m	*	0.0345
IMP:PMA_2DG_15m vs. CTP:PMA_2DG_15m	ns	0.5892
IMP:PMA_2DG_15m vs. CTP:PMA_6AN_15m	ns	0.9946
IMP:PMA_2DG_15m vs. CTP:PMA_DPI_15m	ns	0.1621
IMP:PMA_2DG_15m vs. CTP:PMA_AA_15m	ns	0.9987
IMP:PMA_2DG_15m vs. CTP:PMA_IAA_15m	****	<0.0001
IMP:PMA_2DG_15m vs. Uridine:PMA_15m	ns	>0.9999
IMP:PMA_2DG_15m vs. Uridine:PMA_2DG_15m	ns	>0.9999
IMP:PMA_2DG_15m vs. Uridine:PMA_6AN_15m	ns	>0.9999
IMP:PMA_2DG_15m vs. Uridine:PMA_DPI_15m	ns	>0.9999
IMP:PMA_2DG_15m vs. Uridine:PMA_AA_15m	ns	>0.9999

IMP:PMA_2DG_15m vs. Uridine:PMA_IAA_15m	ns	0.1233
IMP:PMA_2DG_15m vs. UMP:PMA_15m	ns	>0.9999
IMP:PMA_2DG_15m vs. UMP:PMA_2DG_15m	ns	>0.9999
IMP:PMA_2DG_15m vs. UMP:PMA_6AN_15m	ns	>0.9999
IMP:PMA_2DG_15m vs. UMP:PMA_DPI_15m	ns	>0.9999
IMP:PMA_2DG_15m vs. UMP:PMA_AA_15m	ns	>0.9999
IMP:PMA_2DG_15m vs. UMP:PMA_IAA_15m	ns	0.6333
IMP:PMA_2DG_15m vs. UDP:PMA_15m	ns	>0.9999
IMP:PMA_2DG_15m vs. UDP:PMA_2DG_15m	ns	>0.9999
IMP:PMA_2DG_15m vs. UDP:PMA_6AN_15m	ns	>0.9999
IMP:PMA_2DG_15m vs. UDP:PMA_DPI_15m	ns	>0.9999
IMP:PMA_2DG_15m vs. UDP:PMA_AA_15m	ns	>0.9999
IMP:PMA_2DG_15m vs. UDP:PMA_IAA_15m	ns	0.6164
IMP:PMA_2DG_15m vs. UTP:PMA_15m	*	0.0171
IMP:PMA_2DG_15m vs. UTP:PMA_2DG_15m	ns	0.4001
IMP:PMA_2DG_15m vs. UTP:PMA_6AN_15m	ns	0.974
IMP:PMA_2DG_15m vs. UTP:PMA_DPI_15m	ns	0.0917
IMP:PMA_2DG_15m vs. UTP:PMA_AA_15m	ns	0.991
IMP:PMA_2DG_15m vs. UTP:PMA_IAA_15m	****	<0.0001
IMP:PMA_6AN_15m vs. IMP:PMA_DPI_15m	ns	0.8059
IMP:PMA_6AN_15m vs. IMP:PMA_AA_15m	ns	>0.9999
IMP:PMA_6AN_15m vs. IMP:PMA_IAA_15m	****	<0.0001
IMP:PMA_6AN_15m vs. GMP:PMA_15m	ns	>0.9999
IMP:PMA_6AN_15m vs. GMP:PMA_2DG_15m	ns	>0.9999
IMP:PMA_6AN_15m vs. GMP:PMA_6AN_15m	ns	>0.9999
IMP:PMA_6AN_15m vs. GMP:PMA_DPI_15m	ns	>0.9999
IMP:PMA_6AN_15m vs. GMP:PMA_AA_15m	ns	>0.9999
IMP:PMA_6AN_15m vs. GMP:PMA_IAA_15m	ns	0.6144
IMP:PMA_6AN_15m vs. GDP:PMA_15m	ns	>0.9999
IMP:PMA_6AN_15m vs. GDP:PMA_2DG_15m	ns	>0.9999
IMP:PMA_6AN_15m vs. GDP:PMA_6AN_15m	ns	>0.9999
IMP:PMA_6AN_15m vs. GDP:PMA_DPI_15m	ns	>0.9999
IMP:PMA_6AN_15m vs. GDP:PMA_AA_15m	ns	>0.9999
IMP:PMA_6AN_15m vs. GDP:PMA_IAA_15m	ns	0.5516
IMP:PMA_6AN_15m vs. GTP:PMA_15m	**	0.0019
IMP:PMA_6AN_15m vs. GTP:PMA_2DG_15m	ns	0.3136
IMP:PMA_6AN_15m vs. GTP:PMA_6AN_15m	ns	0.3178
IMP:PMA_6AN_15m vs. GTP:PMA_DPI_15m	*	0.0138
IMP:PMA_6AN_15m vs. GTP:PMA_AA_15m	ns	0.7958
IMP:PMA_6AN_15m vs. GTP:PMA_IAA_15m	****	<0.0001
IMP:PMA_6AN_15m vs. Guanosine:PMA_15m	ns	0.9997
IMP:PMA_6AN_15m vs. Guanosine:PMA_2DG_15m	ns	>0.9999
IMP:PMA_6AN_15m vs. Guanosine:PMA_6AN_15m	ns	>0.9999
IMP:PMA_6AN_15m vs. Guanosine:PMA_DPI_15m	ns	>0.9999
IMP:PMA_6AN_15m vs. Guanosine:PMA_AA_15m	ns	>0.9999
IMP:PMA_6AN_15m vs. Guanosine:PMA_IAA_15m	*	0.0386
IMP:PMA_6AN_15m vs. Guanine:PMA_15m	ns	0.0561

IMP:PMA_6AN_15m vs. Guanine:PMA_2DG_15m	ns	0.9462
IMP:PMA_6AN_15m vs. Guanine:PMA_6AN_15m	ns	0.9766
IMP:PMA_6AN_15m vs. Guanine:PMA_DPI_15m	ns	0.2362
IMP:PMA_6AN_15m vs. Guanine:PMA_AA_15m	ns	0.9998
IMP:PMA_6AN_15m vs. Guanine:PMA_IAA_15m	****	<0.0001
IMP:PMA_6AN_15m vs. Aspartic acid:PMA_15m	ns	0.1315
IMP:PMA_6AN_15m vs. Aspartic acid:PMA_2DG_15m	ns	0.9926
IMP:PMA_6AN_15m vs. Aspartic acid:PMA_6AN_15m	ns	0.9986
IMP:PMA_6AN_15m vs. Aspartic acid:PMA_DPI_15m	ns	0.4324
IMP:PMA_6AN_15m vs. Aspartic acid:PMA_AA_15m	ns	>0.9999
IMP:PMA_6AN_15m vs. Aspartic acid:PMA_IAA_15m	****	<0.0001
IMP:PMA_6AN_15m vs. AMP:PMA_15m	ns	0.9804
IMP:PMA_6AN_15m vs. AMP:PMA_2DG_15m	ns	>0.9999
IMP:PMA_6AN_15m vs. AMP:PMA_6AN_15m	ns	>0.9999
IMP:PMA_6AN_15m vs. AMP:PMA_DPI_15m	ns	>0.9999
IMP:PMA_6AN_15m vs. AMP:PMA_AA_15m	ns	>0.9999
IMP:PMA_6AN_15m vs. AMP:PMA_IAA_15m	**	0.0083
IMP:PMA_6AN_15m vs. ADP:PMA_15m	ns	0.8585
IMP:PMA_6AN_15m vs. ADP:PMA_2DG_15m	ns	>0.9999
IMP:PMA_6AN_15m vs. ADP:PMA_6AN_15m	ns	>0.9999
IMP:PMA_6AN_15m vs. ADP:PMA_DPI_15m	ns	0.9937
IMP:PMA_6AN_15m vs. ADP:PMA_AA_15m	ns	>0.9999
IMP:PMA_6AN_15m vs. ADP:PMA_IAA_15m	**	0.002
IMP:PMA_6AN_15m vs. ATP:PMA_15m	***	0.0004
IMP:PMA_6AN_15m vs. ATP:PMA_2DG_15m	ns	0.1269
IMP:PMA_6AN_15m vs. ATP:PMA_6AN_15m	ns	0.1118
IMP:PMA_6AN_15m vs. ATP:PMA_DPI_15m	**	0.0035
IMP:PMA_6AN_15m vs. ATP:PMA_AA_15m	ns	0.5065
IMP:PMA_6AN_15m vs. ATP:PMA_IAA_15m	****	<0.0001
IMP:PMA_6AN_15m vs. Xanthine:PMA_15m	ns	0.0546
IMP:PMA_6AN_15m vs. Xanthine:PMA_2DG_15m	ns	0.9232
IMP:PMA_6AN_15m vs. Xanthine:PMA_6AN_15m	ns	0.9639
IMP:PMA_6AN_15m vs. Xanthine:PMA_DPI_15m	ns	0.2206
IMP:PMA_6AN_15m vs. Xanthine:PMA_AA_15m	ns	0.9993
IMP:PMA_6AN_15m vs. CMP:PMA_15m	ns	0.9932
IMP:PMA_6AN_15m vs. CMP:PMA_2DG_15m	ns	>0.9999
IMP:PMA_6AN_15m vs. CMP:PMA_6AN_15m	ns	>0.9999
IMP:PMA_6AN_15m vs. CMP:PMA_DPI_15m	ns	>0.9999
IMP:PMA_6AN_15m vs. CMP:PMA_AA_15m	ns	>0.9999
IMP:PMA_6AN_15m vs. CMP:PMA_IAA_15m	*	0.0136
IMP:PMA_6AN_15m vs. CDP:PMA_15m	ns	>0.9999
IMP:PMA_6AN_15m vs. CDP:PMA_2DG_15m	ns	>0.9999
IMP:PMA_6AN_15m vs. CDP:PMA_6AN_15m	ns	>0.9999
IMP:PMA_6AN_15m vs. CDP:PMA_DPI_15m	ns	>0.9999
IMP:PMA_6AN_15m vs. CDP:PMA_AA_15m	ns	>0.9999
IMP:PMA_6AN_15m vs. CDP:PMA_IAA_15m	ns	0.0937
IMP:PMA_6AN_15m vs. CTP:PMA_15m	**	0.0063

IMP:PMA_6AN_15m vs. CTP:PMA_2DG_15m	ns	0.5485
IMP:PMA_6AN_15m vs. CTP:PMA_6AN_15m	ns	0.5892
IMP:PMA_6AN_15m vs. CTP:PMA_DPI_15m	*	0.0394
IMP:PMA_6AN_15m vs. CTP:PMA_AA_15m	ns	0.9446
IMP:PMA_6AN_15m vs. CTP:PMA_IAA_15m	****	<0.0001
IMP:PMA_6AN_15m vs. Uridine:PMA_15m	ns	0.9992
IMP:PMA_6AN_15m vs. Uridine:PMA_2DG_15m	ns	>0.9999
IMP:PMA_6AN_15m vs. Uridine:PMA_6AN_15m	ns	>0.9999
IMP:PMA_6AN_15m vs. Uridine:PMA_DPI_15m	ns	>0.9999
IMP:PMA_6AN_15m vs. Uridine:PMA_AA_15m	ns	>0.9999
IMP:PMA_6AN_15m vs. Uridine:PMA_IAA_15m	*	0.0282
IMP:PMA_6AN_15m vs. UMP:PMA_15m	ns	>0.9999
IMP:PMA_6AN_15m vs. UMP:PMA_2DG_15m	ns	>0.9999
IMP:PMA_6AN_15m vs. UMP:PMA_6AN_15m	ns	>0.9999
IMP:PMA_6AN_15m vs. UMP:PMA_DPI_15m	ns	>0.9999
IMP:PMA_6AN_15m vs. UMP:PMA_AA_15m	ns	>0.9999
IMP:PMA_6AN_15m vs. UMP:PMA_IAA_15m	ns	0.2704
IMP:PMA_6AN_15m vs. UDP:PMA_15m	ns	>0.9999
IMP:PMA_6AN_15m vs. UDP:PMA_2DG_15m	ns	>0.9999
IMP:PMA_6AN_15m vs. UDP:PMA_6AN_15m	ns	>0.9999
IMP:PMA_6AN_15m vs. UDP:PMA_DPI_15m	ns	>0.9999
IMP:PMA_6AN_15m vs. UDP:PMA_AA_15m	ns	>0.9999
IMP:PMA_6AN_15m vs. UDP:PMA_IAA_15m	ns	0.258
IMP:PMA_6AN_15m vs. UTP:PMA_15m	**	0.0029
IMP:PMA_6AN_15m vs. UTP:PMA_2DG_15m	ns	0.3848
IMP:PMA_6AN_15m vs. UTP:PMA_6AN_15m	ns	0.4001
IMP:PMA_6AN_15m vs. UTP:PMA_DPI_15m	*	0.0197
IMP:PMA_6AN_15m vs. UTP:PMA_AA_15m	ns	0.8574
IMP:PMA_6AN_15m vs. UTP:PMA_IAA_15m	****	<0.0001
IMP:PMA_DPI_15m vs. IMP:PMA_AA_15m	ns	0.5731
IMP:PMA_DPI_15m vs. IMP:PMA_IAA_15m	***	0.0004
IMP:PMA_DPI_15m vs. GMP:PMA_15m	ns	>0.9999
IMP:PMA_DPI_15m vs. GMP:PMA_2DG_15m	ns	>0.9999
IMP:PMA_DPI_15m vs. GMP:PMA_6AN_15m	ns	0.9986
IMP:PMA_DPI_15m vs. GMP:PMA_DPI_15m	ns	>0.9999
IMP:PMA_DPI_15m vs. GMP:PMA_AA_15m	ns	0.9942
IMP:PMA_DPI_15m vs. GMP:PMA_IAA_15m	ns	>0.9999
IMP:PMA_DPI_15m vs. GDP:PMA_15m	ns	>0.9999
IMP:PMA_DPI_15m vs. GDP:PMA_2DG_15m	ns	>0.9999
IMP:PMA_DPI_15m vs. GDP:PMA_6AN_15m	ns	0.9994
IMP:PMA_DPI_15m vs. GDP:PMA_DPI_15m	ns	>0.9999
IMP:PMA_DPI_15m vs. GDP:PMA_AA_15m	ns	0.9971
IMP:PMA_DPI_15m vs. GDP:PMA_IAA_15m	ns	>0.9999
IMP:PMA_DPI_15m vs. GTP:PMA_15m	ns	0.2873
IMP:PMA_DPI_15m vs. GTP:PMA_2DG_15m	ns	0.9997
IMP:PMA_DPI_15m vs. GTP:PMA_6AN_15m	ns	>0.9999
IMP:PMA_DPI_15m vs. GTP:PMA_DPI_15m	ns	0.3178

IMP:PMA_DPI_15m vs. GTP:PMA_AA_15m	ns	>0.9999
IMP:PMA_DPI_15m vs. GTP:PMA_IAA_15m	****	<0.0001
IMP:PMA_DPI_15m vs. Guanosine:PMA_15m	ns	>0.9999
IMP:PMA_DPI_15m vs. Guanosine:PMA_2DG_15m	ns	>0.9999
IMP:PMA_DPI_15m vs. Guanosine:PMA_6AN_15m	ns	>0.9999
IMP:PMA_DPI_15m vs. Guanosine:PMA_DPI_15m	ns	>0.9999
IMP:PMA_DPI_15m vs. Guanosine:PMA_AA_15m	ns	>0.9999
IMP:PMA_DPI_15m vs. Guanosine:PMA_IAA_15m	ns	0.8825
IMP:PMA_DPI_15m vs. Guanine:PMA_15m	ns	0.9335
IMP:PMA_DPI_15m vs. Guanine:PMA_2DG_15m	ns	>0.9999
IMP:PMA_DPI_15m vs. Guanine:PMA_6AN_15m	ns	>0.9999
IMP:PMA_DPI_15m vs. Guanine:PMA_DPI_15m	ns	0.9766
IMP:PMA_DPI_15m vs. Guanine:PMA_AA_15m	ns	>0.9999
IMP:PMA_DPI_15m vs. Guanine:PMA_IAA_15m	**	0.0038
IMP:PMA_DPI_15m vs. Aspartic acid:PMA_15m	ns	0.9896
IMP:PMA_DPI_15m vs. Aspartic acid:PMA_2DG_15m	ns	>0.9999
IMP:PMA_DPI_15m vs. Aspartic acid:PMA_6AN_15m	ns	>0.9999
IMP:PMA_DPI_15m vs. Aspartic acid:PMA_DPI_15m	ns	0.9986
IMP:PMA_DPI_15m vs. Aspartic acid:PMA_AA_15m	ns	>0.9999
IMP:PMA_DPI_15m vs. Aspartic acid:PMA_IAA_15m	*	0.0113
IMP:PMA_DPI_15m vs. AMP:PMA_15m	ns	>0.9999
IMP:PMA_DPI_15m vs. AMP:PMA_2DG_15m	ns	>0.9999
IMP:PMA_DPI_15m vs. AMP:PMA_6AN_15m	ns	>0.9999
IMP:PMA_DPI_15m vs. AMP:PMA_DPI_15m	ns	>0.9999
IMP:PMA_DPI_15m vs. AMP:PMA_AA_15m	ns	>0.9999
IMP:PMA_DPI_15m vs. AMP:PMA_IAA_15m	ns	0.5706
IMP:PMA_DPI_15m vs. ADP:PMA_15m	ns	>0.9999
IMP:PMA_DPI_15m vs. ADP:PMA_2DG_15m	ns	>0.9999
IMP:PMA_DPI_15m vs. ADP:PMA_6AN_15m	ns	>0.9999
IMP:PMA_DPI_15m vs. ADP:PMA_DPI_15m	ns	>0.9999
IMP:PMA_DPI_15m vs. ADP:PMA_AA_15m	ns	>0.9999
IMP:PMA_DPI_15m vs. ADP:PMA_IAA_15m	ns	0.2888
IMP:PMA_DPI_15m vs. ATP:PMA_15m	ns	0.1134
IMP:PMA_DPI_15m vs. ATP:PMA_2DG_15m	ns	0.9885
IMP:PMA_DPI_15m vs. ATP:PMA_6AN_15m	ns	>0.9999
IMP:PMA_DPI_15m vs. ATP:PMA_DPI_15m	ns	0.1118
IMP:PMA_DPI_15m vs. ATP:PMA_AA_15m	ns	>0.9999
IMP:PMA_DPI_15m vs. ATP:PMA_IAA_15m	****	<0.0001
IMP:PMA_DPI_15m vs. Xanthine:PMA_15m	ns	0.9077
IMP:PMA_DPI_15m vs. Xanthine:PMA_2DG_15m	ns	>0.9999
IMP:PMA_DPI_15m vs. Xanthine:PMA_6AN_15m	ns	>0.9999
IMP:PMA_DPI_15m vs. Xanthine:PMA_DPI_15m	ns	0.9639
IMP:PMA_DPI_15m vs. Xanthine:PMA_AA_15m	ns	>0.9999
IMP:PMA_DPI_15m vs. CMP:PMA_15m	ns	>0.9999
IMP:PMA_DPI_15m vs. CMP:PMA_2DG_15m	ns	>0.9999
IMP:PMA_DPI_15m vs. CMP:PMA_6AN_15m	ns	>0.9999
IMP:PMA_DPI_15m vs. CMP:PMA_DPI_15m	ns	>0.9999

IMP:PMA_DPI_15m vs. CMP:PMA_AA_15m	ns	>0.9999
IMP:PMA_DPI_15m vs. CMP:PMA_IAA_15m	ns	0.6812
IMP:PMA_DPI_15m vs. CDP:PMA_15m	ns	>0.9999
IMP:PMA_DPI_15m vs. CDP:PMA_2DG_15m	ns	>0.9999
IMP:PMA_DPI_15m vs. CDP:PMA_6AN_15m	ns	>0.9999
IMP:PMA_DPI_15m vs. CDP:PMA_DPI_15m	ns	>0.9999
IMP:PMA_DPI_15m vs. CDP:PMA_AA_15m	ns	>0.9999
IMP:PMA_DPI_15m vs. CDP:PMA_IAA_15m	ns	0.9743
IMP:PMA_DPI_15m vs. CTP:PMA_15m	ns	0.5151
IMP:PMA_DPI_15m vs. CTP:PMA_2DG_15m	ns	>0.9999
IMP:PMA_DPI_15m vs. CTP:PMA_6AN_15m	ns	>0.9999
IMP:PMA_DPI_15m vs. CTP:PMA_DPI_15m	ns	0.5892
IMP:PMA_DPI_15m vs. CTP:PMA_AA_15m	ns	>0.9999
IMP:PMA_DPI_15m vs. CTP:PMA_IAA_15m	***	0.0003
IMP:PMA_DPI_15m vs. Uridine:PMA_15m	ns	>0.9999
IMP:PMA_DPI_15m vs. Uridine:PMA_2DG_15m	ns	>0.9999
IMP:PMA_DPI_15m vs. Uridine:PMA_6AN_15m	ns	>0.9999
IMP:PMA_DPI_15m vs. Uridine:PMA_DPI_15m	ns	>0.9999
IMP:PMA_DPI_15m vs. Uridine:PMA_AA_15m	ns	>0.9999
IMP:PMA_DPI_15m vs. Uridine:PMA_IAA_15m	ns	0.8309
IMP:PMA_DPI_15m vs. UMP:PMA_15m	ns	>0.9999
IMP:PMA_DPI_15m vs. UMP:PMA_2DG_15m	ns	>0.9999
IMP:PMA_DPI_15m vs. UMP:PMA_6AN_15m	ns	>0.9999
IMP:PMA_DPI_15m vs. UMP:PMA_DPI_15m	ns	>0.9999
IMP:PMA_DPI_15m vs. UMP:PMA_AA_15m	ns	>0.9999
IMP:PMA_DPI_15m vs. UMP:PMA_IAA_15m	ns	0.9993
IMP:PMA_DPI_15m vs. UDP:PMA_15m	ns	>0.9999
IMP:PMA_DPI_15m vs. UDP:PMA_2DG_15m	ns	>0.9999
IMP:PMA_DPI_15m vs. UDP:PMA_6AN_15m	ns	>0.9999
IMP:PMA_DPI_15m vs. UDP:PMA_DPI_15m	ns	>0.9999
IMP:PMA_DPI_15m vs. UDP:PMA_AA_15m	ns	>0.9999
IMP:PMA_DPI_15m vs. UDP:PMA_IAA_15m	ns	0.9992
IMP:PMA_DPI_15m vs. UTP:PMA_15m	ns	0.3553
IMP:PMA_DPI_15m vs. UTP:PMA_2DG_15m	ns	>0.9999
IMP:PMA_DPI_15m vs. UTP:PMA_6AN_15m	ns	>0.9999
IMP:PMA_DPI_15m vs. UTP:PMA_DPI_15m	ns	0.4001
IMP:PMA_DPI_15m vs. UTP:PMA_AA_15m	ns	>0.9999
IMP:PMA_DPI_15m vs. UTP:PMA_IAA_15m	***	0.0001
IMP:PMA_AA_15m vs. IMP:PMA_IAA_15m	****	<0.0001
IMP:PMA_AA_15m vs. GMP:PMA_15m	ns	>0.9999
IMP:PMA_AA_15m vs. GMP:PMA_2DG_15m	ns	>0.9999
IMP:PMA_AA_15m vs. GMP:PMA_6AN_15m	ns	>0.9999
IMP:PMA_AA_15m vs. GMP:PMA_DPI_15m	ns	>0.9999
IMP:PMA_AA_15m vs. GMP:PMA_AA_15m	ns	>0.9999
IMP:PMA_AA_15m vs. GMP:PMA_IAA_15m	ns	0.4946
IMP:PMA_AA_15m vs. GDP:PMA_15m	ns	>0.9999
IMP:PMA_AA_15m vs. GDP:PMA_2DG_15m	ns	>0.9999

IMP:PMA_AA_15m vs. GDP:PMA_6AN_15m	ns	>0.9999
IMP:PMA_AA_15m vs. GDP:PMA_DPI_15m	ns	>0.9999
IMP:PMA_AA_15m vs. GDP:PMA_AA_15m	ns	>0.9999
IMP:PMA_AA_15m vs. GDP:PMA_IAA_15m	ns	0.4338
IMP:PMA_AA_15m vs. GTP:PMA_15m	**	0.0011
IMP:PMA_AA_15m vs. GTP:PMA_2DG_15m	ns	0.2255
IMP:PMA_AA_15m vs. GTP:PMA_6AN_15m	ns	0.5703
IMP:PMA_AA_15m vs. GTP:PMA_DPI_15m	**	0.0081
IMP:PMA_AA_15m vs. GTP:PMA_AA_15m	ns	0.3178
IMP:PMA_AA_15m vs. GTP:PMA_IAA_15m	****	<0.0001
IMP:PMA_AA_15m vs. Guanosine:PMA_15m	ns	0.9985
IMP:PMA_AA_15m vs. Guanosine:PMA_2DG_15m	ns	>0.9999
IMP:PMA_AA_15m vs. Guanosine:PMA_6AN_15m	ns	>0.9999
IMP:PMA_AA_15m vs. Guanosine:PMA_DPI_15m	ns	>0.9999
IMP:PMA_AA_15m vs. Guanosine:PMA_AA_15m	ns	>0.9999
IMP:PMA_AA_15m vs. Guanosine:PMA_IAA_15m	*	0.0237
IMP:PMA_AA_15m vs. Guanine:PMA_15m	*	0.0351
IMP:PMA_AA_15m vs. Guanine:PMA_2DG_15m	ns	0.8909
IMP:PMA_AA_15m vs. Guanine:PMA_6AN_15m	ns	0.9949
IMP:PMA_AA_15m vs. Guanine:PMA_DPI_15m	ns	0.1644
IMP:PMA_AA_15m vs. Guanine:PMA_AA_15m	ns	0.9766
IMP:PMA_AA_15m vs. Guanine:PMA_IAA_15m	****	<0.0001
IMP:PMA_AA_15m vs. Aspartic acid:PMA_15m	ns	0.0869
IMP:PMA_AA_15m vs. Aspartic acid:PMA_2DG_15m	ns	0.9776
IMP:PMA_AA_15m vs. Aspartic acid:PMA_6AN_15m	ns	0.9998
IMP:PMA_AA_15m vs. Aspartic acid:PMA_DPI_15m	ns	0.3253
IMP:PMA_AA_15m vs. Aspartic acid:PMA_AA_15m	ns	0.9986
IMP:PMA_AA_15m vs. Aspartic acid:PMA_IAA_15m	****	<0.0001
IMP:PMA_AA_15m vs. AMP:PMA_15m	ns	0.9512
IMP:PMA_AA_15m vs. AMP:PMA_2DG_15m	ns	>0.9999
IMP:PMA_AA_15m vs. AMP:PMA_6AN_15m	ns	>0.9999
IMP:PMA_AA_15m vs. AMP:PMA_DPI_15m	ns	0.9994
IMP:PMA_AA_15m vs. AMP:PMA_AA_15m	ns	>0.9999
IMP:PMA_AA_15m vs. AMP:PMA_IAA_15m	**	0.0048
IMP:PMA_AA_15m vs. ADP:PMA_15m	ns	0.7662
IMP:PMA_AA_15m vs. ADP:PMA_2DG_15m	ns	>0.9999
IMP:PMA_AA_15m vs. ADP:PMA_6AN_15m	ns	>0.9999
IMP:PMA_AA_15m vs. ADP:PMA_DPI_15m	ns	0.9805
IMP:PMA_AA_15m vs. ADP:PMA_AA_15m	ns	>0.9999
IMP:PMA_AA_15m vs. ADP:PMA_IAA_15m	**	0.0011
IMP:PMA_AA_15m vs. ATP:PMA_15m	***	0.0002
IMP:PMA_AA_15m vs. ATP:PMA_2DG_15m	ns	0.0837
IMP:PMA_AA_15m vs. ATP:PMA_6AN_15m	ns	0.2901
IMP:PMA_AA_15m vs. ATP:PMA_DPI_15m	**	0.0019
IMP:PMA_AA_15m vs. ATP:PMA_AA_15m	ns	0.1118
IMP:PMA_AA_15m vs. ATP:PMA_IAA_15m	****	<0.0001
IMP:PMA_AA_15m vs. Xanthine:PMA_15m	*	0.0347

IMP:PMA_AA_15m vs. Xanthine:PMA_2DG_15m	ns	0.8584
IMP:PMA_AA_15m vs. Xanthine:PMA_6AN_15m	ns	0.9898
IMP:PMA_AA_15m vs. Xanthine:PMA_DPI_15m	ns	0.1548
IMP:PMA_AA_15m vs. Xanthine:PMA_AA_15m	ns	0.9639
IMP:PMA_AA_15m vs. CMP:PMA_15m	ns	0.979
IMP:PMA_AA_15m vs. CMP:PMA_2DG_15m	ns	>0.9999
IMP:PMA_AA_15m vs. CMP:PMA_6AN_15m	ns	>0.9999
IMP:PMA_AA_15m vs. CMP:PMA_DPI_15m	ns	0.9999
IMP:PMA_AA_15m vs. CMP:PMA_AA_15m	ns	>0.9999
IMP:PMA_AA_15m vs. CMP:PMA_IAA_15m	**	0.008
IMP:PMA_AA_15m vs. CDP:PMA_15m	ns	>0.9999
IMP:PMA_AA_15m vs. CDP:PMA_2DG_15m	ns	>0.9999
IMP:PMA_AA_15m vs. CDP:PMA_6AN_15m	ns	>0.9999
IMP:PMA_AA_15m vs. CDP:PMA_DPI_15m	ns	>0.9999
IMP:PMA_AA_15m vs. CDP:PMA_AA_15m	ns	>0.9999
IMP:PMA_AA_15m vs. CDP:PMA_IAA_15m	ns	0.0605
IMP:PMA_AA_15m vs. CTP:PMA_15m	**	0.0036
IMP:PMA_AA_15m vs. CTP:PMA_2DG_15m	ns	0.4305
IMP:PMA_AA_15m vs. CTP:PMA_6AN_15m	ns	0.8056
IMP:PMA_AA_15m vs. CTP:PMA_DPI_15m	*	0.0242
IMP:PMA_AA_15m vs. CTP:PMA_AA_15m	ns	0.5892
IMP:PMA_AA_15m vs. CTP:PMA_IAA_15m	****	<0.0001
IMP:PMA_AA_15m vs. Uridine:PMA_15m	ns	0.9962
IMP:PMA_AA_15m vs. Uridine:PMA_2DG_15m	ns	>0.9999
IMP:PMA_AA_15m vs. Uridine:PMA_6AN_15m	ns	>0.9999
IMP:PMA_AA_15m vs. Uridine:PMA_DPI_15m	ns	>0.9999
IMP:PMA_AA_15m vs. Uridine:PMA_AA_15m	ns	>0.9999
IMP:PMA_AA_15m vs. Uridine:PMA_IAA_15m	*	0.0171
IMP:PMA_AA_15m vs. UMP:PMA_15m	ns	>0.9999
IMP:PMA_AA_15m vs. UMP:PMA_2DG_15m	ns	>0.9999
IMP:PMA_AA_15m vs. UMP:PMA_6AN_15m	ns	>0.9999
IMP:PMA_AA_15m vs. UMP:PMA_DPI_15m	ns	>0.9999
IMP:PMA_AA_15m vs. UMP:PMA_AA_15m	ns	>0.9999
IMP:PMA_AA_15m vs. UMP:PMA_IAA_15m	ns	0.1913
IMP:PMA_AA_15m vs. UDP:PMA_15m	ns	>0.9999
IMP:PMA_AA_15m vs. UDP:PMA_2DG_15m	ns	>0.9999
IMP:PMA_AA_15m vs. UDP:PMA_6AN_15m	ns	>0.9999
IMP:PMA_AA_15m vs. UDP:PMA_DPI_15m	ns	>0.9999
IMP:PMA_AA_15m vs. UDP:PMA_AA_15m	ns	>0.9999
IMP:PMA_AA_15m vs. UDP:PMA_IAA_15m	ns	0.1816
IMP:PMA_AA_15m vs. UTP:PMA_15m	**	0.0016
IMP:PMA_AA_15m vs. UTP:PMA_2DG_15m	ns	0.2844
IMP:PMA_AA_15m vs. UTP:PMA_6AN_15m	ns	0.6531
IMP:PMA_AA_15m vs. UTP:PMA_DPI_15m	*	0.0117
IMP:PMA_AA_15m vs. UTP:PMA_AA_15m	ns	0.4001
IMP:PMA_AA_15m vs. UTP:PMA_IAA_15m	****	<0.0001
IMP:PMA_IAA_15m vs. GMP:PMA_15m	ns	0.8011

IMP:PMA_IAA_15m vs. GMP:PMA_2DG_15m	*	0.0209
IMP:PMA_IAA_15m vs. GMP:PMA_6AN_15m	**	0.0036
IMP:PMA_IAA_15m vs. GMP:PMA_DPI_15m	ns	0.3957
IMP:PMA_IAA_15m vs. GMP:PMA_AA_15m	**	0.002
IMP:PMA_IAA_15m vs. GMP:PMA_IAA_15m	ns	>0.9999
IMP:PMA_IAA_15m vs. GDP:PMA_15m	ns	0.848
IMP:PMA_IAA_15m vs. GDP:PMA_2DG_15m	*	0.0272
IMP:PMA_IAA_15m vs. GDP:PMA_6AN_15m	**	0.0048
IMP:PMA_IAA_15m vs. GDP:PMA_DPI_15m	ns	0.4546
IMP:PMA_IAA_15m vs. GDP:PMA_AA_15m	**	0.0028
IMP:PMA_IAA_15m vs. GDP:PMA_IAA_15m	ns	>0.9999
IMP:PMA_IAA_15m vs. GTP:PMA_15m	ns	>0.9999
IMP:PMA_IAA_15m vs. GTP:PMA_2DG_15m	ns	>0.9999
IMP:PMA_IAA_15m vs. GTP:PMA_6AN_15m	ns	>0.9999
IMP:PMA_IAA_15m vs. GTP:PMA_DPI_15m	ns	>0.9999
IMP:PMA_IAA_15m vs. GTP:PMA_AA_15m	ns	>0.9999
IMP:PMA_IAA_15m vs. GTP:PMA_IAA_15m	ns	0.3178
IMP:PMA_IAA_15m vs. Guanosine:PMA_15m	ns	>0.9999
IMP:PMA_IAA_15m vs. Guanosine:PMA_2DG_15m	ns	0.4656
IMP:PMA_IAA_15m vs. Guanosine:PMA_6AN_15m	ns	0.1646
IMP:PMA_IAA_15m vs. Guanosine:PMA_DPI_15m	ns	0.9946
IMP:PMA_IAA_15m vs. Guanosine:PMA_AA_15m	ns	0.111
IMP:PMA_IAA_15m vs. Guanosine:PMA_IAA_15m	ns	>0.9999
IMP:PMA_IAA_15m vs. Guanine:PMA_15m	ns	>0.9999
IMP:PMA_IAA_15m vs. Guanine:PMA_2DG_15m	ns	>0.9999
IMP:PMA_IAA_15m vs. Guanine:PMA_6AN_15m	ns	>0.9999
IMP:PMA_IAA_15m vs. Guanine:PMA_DPI_15m	ns	>0.9999
IMP:PMA_IAA_15m vs. Guanine:PMA_AA_15m	ns	>0.9999
IMP:PMA_IAA_15m vs. Guanine:PMA_IAA_15m	ns	0.9766
IMP:PMA_IAA_15m vs. Aspartic acid:PMA_15m	ns	>0.9999
IMP:PMA_IAA_15m vs. Aspartic acid:PMA_2DG_15m	ns	>0.9999
IMP:PMA_IAA_15m vs. Aspartic acid:PMA_6AN_15m	ns	>0.9999
IMP:PMA_IAA_15m vs. Aspartic acid:PMA_DPI_15m	ns	>0.9999
IMP:PMA_IAA_15m vs. Aspartic acid:PMA_AA_15m	ns	0.9994
IMP:PMA_IAA_15m vs. Aspartic acid:PMA_IAA_15m	ns	0.9986
IMP:PMA_IAA_15m vs. AMP:PMA_15m	ns	>0.9999
IMP:PMA_IAA_15m vs. AMP:PMA_2DG_15m	ns	0.8094
IMP:PMA_IAA_15m vs. AMP:PMA_6AN_15m	ns	0.4364
IMP:PMA_IAA_15m vs. AMP:PMA_DPI_15m	ns	>0.9999
IMP:PMA_IAA_15m vs. AMP:PMA_AA_15m	ns	0.3292
IMP:PMA_IAA_15m vs. AMP:PMA_IAA_15m	ns	>0.9999
IMP:PMA_IAA_15m vs. ADP:PMA_15m	ns	>0.9999
IMP:PMA_IAA_15m vs. ADP:PMA_2DG_15m	ns	0.9662
IMP:PMA_IAA_15m vs. ADP:PMA_6AN_15m	ns	0.7358
IMP:PMA_IAA_15m vs. ADP:PMA_DPI_15m	ns	>0.9999
IMP:PMA_IAA_15m vs. ADP:PMA_AA_15m	ns	0.6211
IMP:PMA_IAA_15m vs. ADP:PMA_IAA_15m	ns	>0.9999

IMP:PMA_IAA_15m vs. ATP:PMA_15m	ns	>0.9999
IMP:PMA_IAA_15m vs. ATP:PMA_2DG_15m	ns	>0.9999
IMP:PMA_IAA_15m vs. ATP:PMA_6AN_15m	ns	>0.9999
IMP:PMA_IAA_15m vs. ATP:PMA_DPI_15m	ns	>0.9999
IMP:PMA_IAA_15m vs. ATP:PMA_AA_15m	ns	>0.9999
IMP:PMA_IAA_15m vs. ATP:PMA_IAA_15m	ns	0.1118
IMP:PMA_IAA_15m vs. Xanthine:PMA_15m	ns	>0.9999
IMP:PMA_IAA_15m vs. Xanthine:PMA_2DG_15m	ns	>0.9999
IMP:PMA_IAA_15m vs. Xanthine:PMA_6AN_15m	ns	>0.9999
IMP:PMA_IAA_15m vs. Xanthine:PMA_DPI_15m	ns	>0.9999
IMP:PMA_IAA_15m vs. Xanthine:PMA_AA_15m	ns	>0.9999
IMP:PMA_IAA_15m vs. CMP:PMA_15m	ns	>0.9999
IMP:PMA_IAA_15m vs. CMP:PMA_2DG_15m	ns	0.7131
IMP:PMA_IAA_15m vs. CMP:PMA_6AN_15m	ns	0.3359
IMP:PMA_IAA_15m vs. CMP:PMA_DPI_15m	ns	0.9998
IMP:PMA_IAA_15m vs. CMP:PMA_AA_15m	ns	0.244
IMP:PMA_IAA_15m vs. CMP:PMA_IAA_15m	ns	>0.9999
IMP:PMA_IAA_15m vs. CDP:PMA_15m	ns	0.9995
IMP:PMA_IAA_15m vs. CDP:PMA_2DG_15m	ns	0.2621
IMP:PMA_IAA_15m vs. CDP:PMA_6AN_15m	ns	0.0734
IMP:PMA_IAA_15m vs. CDP:PMA_DPI_15m	ns	0.9573
IMP:PMA_IAA_15m vs. CDP:PMA_AA_15m	*	0.0467
IMP:PMA_IAA_15m vs. CDP:PMA_IAA_15m	ns	>0.9999
IMP:PMA_IAA_15m vs. CTP:PMA_15m	ns	>0.9999
IMP:PMA_IAA_15m vs. CTP:PMA_2DG_15m	ns	>0.9999
IMP:PMA_IAA_15m vs. CTP:PMA_6AN_15m	ns	>0.9999
IMP:PMA_IAA_15m vs. CTP:PMA_DPI_15m	ns	>0.9999
IMP:PMA_IAA_15m vs. CTP:PMA_AA_15m	ns	>0.9999
IMP:PMA_IAA_15m vs. CTP:PMA_IAA_15m	ns	0.5892
IMP:PMA_IAA_15m vs. Uridine:PMA_15m	ns	>0.9999
IMP:PMA_IAA_15m vs. Uridine:PMA_2DG_15m	ns	0.5422
IMP:PMA_IAA_15m vs. Uridine:PMA_6AN_15m	ns	0.2086
IMP:PMA_IAA_15m vs. Uridine:PMA_DPI_15m	ns	0.9978
IMP:PMA_IAA_15m vs. Uridine:PMA_AA_15m	ns	0.1437
IMP:PMA_IAA_15m vs. Uridine:PMA_IAA_15m	ns	>0.9999
IMP:PMA_IAA_15m vs. UMP:PMA_15m	ns	0.9785
IMP:PMA_IAA_15m vs. UMP:PMA_2DG_15m	ns	0.09
IMP:PMA_IAA_15m vs. UMP:PMA_6AN_15m	*	0.0193
IMP:PMA_IAA_15m vs. UMP:PMA_DPI_15m	ns	0.7574
IMP:PMA_IAA_15m vs. UMP:PMA_AA_15m	*	0.0115
IMP:PMA_IAA_15m vs. UMP:PMA_IAA_15m	ns	>0.9999
IMP:PMA_IAA_15m vs. UDP:PMA_15m	ns	0.9813
IMP:PMA_IAA_15m vs. UDP:PMA_2DG_15m	ns	0.0956
IMP:PMA_IAA_15m vs. UDP:PMA_6AN_15m	*	0.0208
IMP:PMA_IAA_15m vs. UDP:PMA_DPI_15m	ns	0.7721
IMP:PMA_IAA_15m vs. UDP:PMA_AA_15m	*	0.0124
IMP:PMA_IAA_15m vs. UDP:PMA_IAA_15m	ns	>0.9999

IMP:PMA_IAA_15m vs. UTP:PMA_15m	ns	>0.9999
IMP:PMA_IAA_15m vs. UTP:PMA_2DG_15m	ns	>0.9999
IMP:PMA_IAA_15m vs. UTP:PMA_6AN_15m	ns	>0.9999
IMP:PMA_IAA_15m vs. UTP:PMA_DPI_15m	ns	>0.9999
IMP:PMA_IAA_15m vs. UTP:PMA_AA_15m	ns	>0.9999
IMP:PMA_IAA_15m vs. UTP:PMA_IAA_15m	ns	0.4001
GMP:PMA_15m vs. GMP:PMA_2DG_15m	ns	0.7475
GMP:PMA_15m vs. GMP:PMA_6AN_15m	ns	0.1037
GMP:PMA_15m vs. GMP:PMA_DPI_15m	ns	>0.9999
GMP:PMA_15m vs. GMP:PMA_AA_15m	*	0.0409
GMP:PMA_15m vs. GMP:PMA_IAA_15m	*	0.0236
GMP:PMA_15m vs. GDP:PMA_15m	ns	>0.9999
GMP:PMA_15m vs. GDP:PMA_2DG_15m	ns	>0.9999
GMP:PMA_15m vs. GDP:PMA_6AN_15m	ns	>0.9999
GMP:PMA_15m vs. GDP:PMA_DPI_15m	ns	>0.9999
GMP:PMA_15m vs. GDP:PMA_AA_15m	ns	>0.9999
GMP:PMA_15m vs. GDP:PMA_IAA_15m	ns	0.9993
GMP:PMA_15m vs. GTP:PMA_15m	*	0.0156
GMP:PMA_15m vs. GTP:PMA_2DG_15m	ns	0.9868
GMP:PMA_15m vs. GTP:PMA_6AN_15m	ns	>0.9999
GMP:PMA_15m vs. GTP:PMA_DPI_15m	ns	0.3775
GMP:PMA_15m vs. GTP:PMA_AA_15m	ns	>0.9999
GMP:PMA_15m vs. GTP:PMA_IAA_15m	****	<0.0001
GMP:PMA_15m vs. Guanosine:PMA_15m	ns	>0.9999
GMP:PMA_15m vs. Guanosine:PMA_2DG_15m	ns	>0.9999
GMP:PMA_15m vs. Guanosine:PMA_6AN_15m	ns	>0.9999
GMP:PMA_15m vs. Guanosine:PMA_DPI_15m	ns	>0.9999
GMP:PMA_15m vs. Guanosine:PMA_AA_15m	ns	>0.9999
GMP:PMA_15m vs. Guanosine:PMA_IAA_15m	ns	0.6151
GMP:PMA_15m vs. Guanine:PMA_15m	ns	0.341
GMP:PMA_15m vs. Guanine:PMA_2DG_15m	ns	>0.9999
GMP:PMA_15m vs. Guanine:PMA_6AN_15m	ns	>0.9999
GMP:PMA_15m vs. Guanine:PMA_DPI_15m	ns	0.9684
GMP:PMA_15m vs. Guanine:PMA_AA_15m	ns	>0.9999
GMP:PMA_15m vs. Guanine:PMA_IAA_15m	***	0.0008
GMP:PMA_15m vs. Aspartic acid:PMA_15m	ns	0.6013
GMP:PMA_15m vs. Aspartic acid:PMA_2DG_15m	ns	>0.9999
GMP:PMA_15m vs. Aspartic acid:PMA_6AN_15m	ns	>0.9999
GMP:PMA_15m vs. Aspartic acid:PMA_DPI_15m	ns	0.9967
GMP:PMA_15m vs. Aspartic acid:PMA_AA_15m	ns	>0.9999
GMP:PMA_15m vs. Aspartic acid:PMA_IAA_15m	**	0.0026
GMP:PMA_15m vs. AMP:PMA_15m	ns	>0.9999
GMP:PMA_15m vs. AMP:PMA_2DG_15m	ns	>0.9999
GMP:PMA_15m vs. AMP:PMA_6AN_15m	ns	>0.9999
GMP:PMA_15m vs. AMP:PMA_DPI_15m	ns	>0.9999
GMP:PMA_15m vs. AMP:PMA_AA_15m	ns	>0.9999
GMP:PMA_15m vs. AMP:PMA_IAA_15m	ns	0.2794

GMP:PMA_15m vs. ADP:PMA_15m	ns	0.9999
GMP:PMA_15m vs. ADP:PMA_2DG_15m	ns	>0.9999
GMP:PMA_15m vs. ADP:PMA_6AN_15m	ns	>0.9999
GMP:PMA_15m vs. ADP:PMA_DPI_15m	ns	>0.9999
GMP:PMA_15m vs. ADP:PMA_AA_15m	ns	>0.9999
GMP:PMA_15m vs. ADP:PMA_IAA_15m	ns	0.1087
GMP:PMA_15m vs. ATP:PMA_15m	**	0.0032
GMP:PMA_15m vs. ATP:PMA_2DG_15m	ns	0.8882
GMP:PMA_15m vs. ATP:PMA_6AN_15m	ns	0.9946
GMP:PMA_15m vs. ATP:PMA_DPI_15m	ns	0.162
GMP:PMA_15m vs. ATP:PMA_AA_15m	ns	0.9987
GMP:PMA_15m vs. ATP:PMA_IAA_15m	****	<0.0001
GMP:PMA_15m vs. Xanthine:PMA_15m	ns	0.3289
GMP:PMA_15m vs. Xanthine:PMA_2DG_15m	ns	>0.9999
GMP:PMA_15m vs. Xanthine:PMA_6AN_15m	ns	>0.9999
GMP:PMA_15m vs. Xanthine:PMA_DPI_15m	ns	0.9514
GMP:PMA_15m vs. Xanthine:PMA_AA_15m	ns	>0.9999
GMP:PMA_15m vs. CMP:PMA_15m	ns	>0.9999
GMP:PMA_15m vs. CMP:PMA_2DG_15m	ns	>0.9999
GMP:PMA_15m vs. CMP:PMA_6AN_15m	ns	>0.9999
GMP:PMA_15m vs. CMP:PMA_DPI_15m	ns	>0.9999
GMP:PMA_15m vs. CMP:PMA_AA_15m	ns	>0.9999
GMP:PMA_15m vs. CMP:PMA_IAA_15m	ns	0.3713
GMP:PMA_15m vs. CDP:PMA_15m	ns	>0.9999
GMP:PMA_15m vs. CDP:PMA_2DG_15m	ns	>0.9999
GMP:PMA_15m vs. CDP:PMA_6AN_15m	ns	>0.9999
GMP:PMA_15m vs. CDP:PMA_DPI_15m	ns	>0.9999
GMP:PMA_15m vs. CDP:PMA_AA_15m	ns	>0.9999
GMP:PMA_15m vs. CDP:PMA_IAA_15m	ns	0.8273
GMP:PMA_15m vs. CTP:PMA_15m	ns	0.0507
GMP:PMA_15m vs. CTP:PMA_2DG_15m	ns	0.9993
GMP:PMA_15m vs. CTP:PMA_6AN_15m	ns	>0.9999
GMP:PMA_15m vs. CTP:PMA_DPI_15m	ns	0.6236
GMP:PMA_15m vs. CTP:PMA_AA_15m	ns	>0.9999
GMP:PMA_15m vs. CTP:PMA_IAA_15m	****	<0.0001
GMP:PMA_15m vs. Uridine:PMA_15m	ns	>0.9999
GMP:PMA_15m vs. Uridine:PMA_2DG_15m	ns	>0.9999
GMP:PMA_15m vs. Uridine:PMA_6AN_15m	ns	>0.9999
GMP:PMA_15m vs. Uridine:PMA_DPI_15m	ns	>0.9999
GMP:PMA_15m vs. Uridine:PMA_AA_15m	ns	>0.9999
GMP:PMA_15m vs. Uridine:PMA_IAA_15m	ns	0.5375
GMP:PMA_15m vs. UMP:PMA_15m	ns	>0.9999
GMP:PMA_15m vs. UMP:PMA_2DG_15m	ns	>0.9999
GMP:PMA_15m vs. UMP:PMA_6AN_15m	ns	>0.9999
GMP:PMA_15m vs. UMP:PMA_DPI_15m	ns	>0.9999
GMP:PMA_15m vs. UMP:PMA_AA_15m	ns	>0.9999
GMP:PMA_15m vs. UMP:PMA_IAA_15m	ns	0.9778

GMP:PMA_15m vs. UDP:PMA_15m	ns	>0.9999
GMP:PMA_15m vs. UDP:PMA_2DG_15m	ns	>0.9999
GMP:PMA_15m vs. UDP:PMA_6AN_15m	ns	>0.9999
GMP:PMA_15m vs. UDP:PMA_DPI_15m	ns	>0.9999
GMP:PMA_15m vs. UDP:PMA_AA_15m	ns	>0.9999
GMP:PMA_15m vs. UDP:PMA_IAA_15m	ns	0.9746
GMP:PMA_15m vs. UTP:PMA_15m	*	0.0233
GMP:PMA_15m vs. UTP:PMA_2DG_15m	ns	0.9942
GMP:PMA_15m vs. UTP:PMA_6AN_15m	ns	>0.9999
GMP:PMA_15m vs. UTP:PMA_DPI_15m	ns	0.455
GMP:PMA_15m vs. UTP:PMA_AA_15m	ns	>0.9999
GMP:PMA_15m vs. UTP:PMA_IAA_15m	****	<0.0001
GMP:PMA_2DG_15m vs. GMP:PMA_6AN_15m	ns	>0.9999
GMP:PMA_2DG_15m vs. GMP:PMA_DPI_15m	ns	>0.9999
GMP:PMA_2DG_15m vs. GMP:PMA_AA_15m	ns	>0.9999
GMP:PMA_2DG_15m vs. GMP:PMA_IAA_15m	****	<0.0001
GMP:PMA_2DG_15m vs. GDP:PMA_15m	ns	>0.9999
GMP:PMA_2DG_15m vs. GDP:PMA_2DG_15m	ns	>0.9999
GMP:PMA_2DG_15m vs. GDP:PMA_6AN_15m	ns	>0.9999
GMP:PMA_2DG_15m vs. GDP:PMA_DPI_15m	ns	>0.9999
GMP:PMA_2DG_15m vs. GDP:PMA_AA_15m	ns	>0.9999
GMP:PMA_2DG_15m vs. GDP:PMA_IAA_15m	ns	0.2418
GMP:PMA_2DG_15m vs. GTP:PMA_15m	***	0.0003
GMP:PMA_2DG_15m vs. GTP:PMA_2DG_15m	*	0.0156
GMP:PMA_2DG_15m vs. GTP:PMA_6AN_15m	ns	0.3483
GMP:PMA_2DG_15m vs. GTP:PMA_DPI_15m	**	0.0027
GMP:PMA_2DG_15m vs. GTP:PMA_AA_15m	ns	0.4586
GMP:PMA_2DG_15m vs. GTP:PMA_IAA_15m	****	<0.0001
GMP:PMA_2DG_15m vs. Guanosine:PMA_15m	ns	0.9822
GMP:PMA_2DG_15m vs. Guanosine:PMA_2DG_15m	ns	>0.9999
GMP:PMA_2DG_15m vs. Guanosine:PMA_6AN_15m	ns	>0.9999
GMP:PMA_2DG_15m vs. Guanosine:PMA_DPI_15m	ns	>0.9999
GMP:PMA_2DG_15m vs. Guanosine:PMA_AA_15m	ns	>0.9999
GMP:PMA_2DG_15m vs. Guanosine:PMA_IAA_15m	**	0.0087
GMP:PMA_2DG_15m vs. Guanine:PMA_15m	*	0.0132
GMP:PMA_2DG_15m vs. Guanine:PMA_2DG_15m	ns	0.341
GMP:PMA_2DG_15m vs. Guanine:PMA_6AN_15m	ns	0.9596
GMP:PMA_2DG_15m vs. Guanine:PMA_DPI_15m	ns	0.0743
GMP:PMA_2DG_15m vs. Guanine:PMA_AA_15m	ns	0.9845
GMP:PMA_2DG_15m vs. Guanine:PMA_IAA_15m	****	<0.0001
GMP:PMA_2DG_15m vs. Aspartic acid:PMA_15m	*	0.036
GMP:PMA_2DG_15m vs. Aspartic acid:PMA_2DG_15m	ns	0.6013
GMP:PMA_2DG_15m vs. Aspartic acid:PMA_6AN_15m	ns	0.9952
GMP:PMA_2DG_15m vs. Aspartic acid:PMA_DPI_15m	ns	0.1676
GMP:PMA_2DG_15m vs. Aspartic acid:PMA_AA_15m	ns	0.9989
GMP:PMA_2DG_15m vs. Aspartic acid:PMA_IAA_15m	****	<0.0001
GMP:PMA_2DG_15m vs. AMP:PMA_15m	ns	0.8263

GMP:PMA_2DG_15m vs. AMP:PMA_2DG_15m	ns	>0.9999
GMP:PMA_2DG_15m vs. AMP:PMA_6AN_15m	ns	>0.9999
GMP:PMA_2DG_15m vs. AMP:PMA_DPI_15m	ns	0.9901
GMP:PMA_2DG_15m vs. AMP:PMA_AA_15m	ns	>0.9999
GMP:PMA_2DG_15m vs. AMP:PMA_IAA_15m	**	0.0016
GMP:PMA_2DG_15m vs. ADP:PMA_15m	ns	0.5425
GMP:PMA_2DG_15m vs. ADP:PMA_2DG_15m	ns	0.9999
GMP:PMA_2DG_15m vs. ADP:PMA_6AN_15m	ns	>0.9999
GMP:PMA_2DG_15m vs. ADP:PMA_DPI_15m	ns	0.9034
GMP:PMA_2DG_15m vs. ADP:PMA_AA_15m	ns	>0.9999
GMP:PMA_2DG_15m vs. ADP:PMA_IAA_15m	***	0.0003
GMP:PMA_2DG_15m vs. ATP:PMA_15m	****	<0.0001
GMP:PMA_2DG_15m vs. ATP:PMA_2DG_15m	**	0.0032
GMP:PMA_2DG_15m vs. ATP:PMA_6AN_15m	ns	0.1456
GMP:PMA_2DG_15m vs. ATP:PMA_DPI_15m	***	0.0006
GMP:PMA_2DG_15m vs. ATP:PMA_AA_15m	ns	0.2114
GMP:PMA_2DG_15m vs. ATP:PMA_IAA_15m	****	<0.0001
GMP:PMA_2DG_15m vs. Xanthine:PMA_15m	*	0.0136
GMP:PMA_2DG_15m vs. Xanthine:PMA_2DG_15m	ns	0.3289
GMP:PMA_2DG_15m vs. Xanthine:PMA_6AN_15m	ns	0.9399
GMP:PMA_2DG_15m vs. Xanthine:PMA_DPI_15m	ns	0.0716
GMP:PMA_2DG_15m vs. Xanthine:PMA_AA_15m	ns	0.9737
GMP:PMA_2DG_15m vs. CMP:PMA_15m	ns	0.8988
GMP:PMA_2DG_15m vs. CMP:PMA_2DG_15m	ns	>0.9999
GMP:PMA_2DG_15m vs. CMP:PMA_6AN_15m	ns	>0.9999
GMP:PMA_2DG_15m vs. CMP:PMA_DPI_15m	ns	0.997
GMP:PMA_2DG_15m vs. CMP:PMA_AA_15m	ns	>0.9999
GMP:PMA_2DG_15m vs. CMP:PMA_IAA_15m	**	0.0027
GMP:PMA_2DG_15m vs. CDP:PMA_15m	ns	0.9986
GMP:PMA_2DG_15m vs. CDP:PMA_2DG_15m	ns	>0.9999
GMP:PMA_2DG_15m vs. CDP:PMA_6AN_15m	ns	>0.9999
GMP:PMA_2DG_15m vs. CDP:PMA_DPI_15m	ns	>0.9999
GMP:PMA_2DG_15m vs. CDP:PMA_AA_15m	ns	>0.9999
GMP:PMA_2DG_15m vs. CDP:PMA_IAA_15m	*	0.0241
GMP:PMA_2DG_15m vs. CTP:PMA_15m	**	0.0012
GMP:PMA_2DG_15m vs. CTP:PMA_2DG_15m	ns	0.0507
GMP:PMA_2DG_15m vs. CTP:PMA_6AN_15m	ns	0.5903
GMP:PMA_2DG_15m vs. CTP:PMA_DPI_15m	**	0.0088
GMP:PMA_2DG_15m vs. CTP:PMA_AA_15m	ns	0.7078
GMP:PMA_2DG_15m vs. CTP:PMA_IAA_15m	****	<0.0001
GMP:PMA_2DG_15m vs. Uridine:PMA_15m	ns	0.9668
GMP:PMA_2DG_15m vs. Uridine:PMA_2DG_15m	ns	>0.9999
GMP:PMA_2DG_15m vs. Uridine:PMA_6AN_15m	ns	>0.9999
GMP:PMA_2DG_15m vs. Uridine:PMA_DPI_15m	ns	0.9997
GMP:PMA_2DG_15m vs. Uridine:PMA_AA_15m	ns	>0.9999
GMP:PMA_2DG_15m vs. Uridine:PMA_IAA_15m	**	0.0061
GMP:PMA_2DG_15m vs. UMP:PMA_15m	ns	>0.9999

GMP:PMA_2DG_15m vs. UMP:PMA_2DG_15m	ns	>0.9999
GMP:PMA_2DG_15m vs. UMP:PMA_6AN_15m	ns	>0.9999
GMP:PMA_2DG_15m vs. UMP:PMA_DPI_15m	ns	>0.9999
GMP:PMA_2DG_15m vs. UMP:PMA_AA_15m	ns	>0.9999
GMP:PMA_2DG_15m vs. UMP:PMA_IAA_15m	ns	0.0889
GMP:PMA_2DG_15m vs. UDP:PMA_15m	ns	>0.9999
GMP:PMA_2DG_15m vs. UDP:PMA_2DG_15m	ns	>0.9999
GMP:PMA_2DG_15m vs. UDP:PMA_6AN_15m	ns	>0.9999
GMP:PMA_2DG_15m vs. UDP:PMA_DPI_15m	ns	>0.9999
GMP:PMA_2DG_15m vs. UDP:PMA_AA_15m	ns	>0.9999
GMP:PMA_2DG_15m vs. UDP:PMA_IAA_15m	ns	0.0836
GMP:PMA_2DG_15m vs. UTP:PMA_15m	***	0.0005
GMP:PMA_2DG_15m vs. UTP:PMA_2DG_15m	*	0.0233
GMP:PMA_2DG_15m vs. UTP:PMA_6AN_15m	ns	0.4232
GMP:PMA_2DG_15m vs. UTP:PMA_DPI_15m	**	0.0041
GMP:PMA_2DG_15m vs. UTP:PMA_AA_15m	ns	0.5407
GMP:PMA_2DG_15m vs. UTP:PMA_IAA_15m	****	<0.0001
GMP:PMA_6AN_15m vs. GMP:PMA_DPI_15m	ns	0.8059
GMP:PMA_6AN_15m vs. GMP:PMA_AA_15m	ns	>0.9999
GMP:PMA_6AN_15m vs. GMP:PMA_IAA_15m	****	<0.0001
GMP:PMA_6AN_15m vs. GDP:PMA_15m	ns	>0.9999
GMP:PMA_6AN_15m vs. GDP:PMA_2DG_15m	ns	>0.9999
GMP:PMA_6AN_15m vs. GDP:PMA_6AN_15m	ns	>0.9999
GMP:PMA_6AN_15m vs. GDP:PMA_DPI_15m	ns	>0.9999
GMP:PMA_6AN_15m vs. GDP:PMA_AA_15m	ns	>0.9999
GMP:PMA_6AN_15m vs. GDP:PMA_IAA_15m	ns	0.066
GMP:PMA_6AN_15m vs. GTP:PMA_15m	****	<0.0001
GMP:PMA_6AN_15m vs. GTP:PMA_2DG_15m	*	0.0238
GMP:PMA_6AN_15m vs. GTP:PMA_6AN_15m	*	0.0156
GMP:PMA_6AN_15m vs. GTP:PMA_DPI_15m	***	0.0004
GMP:PMA_6AN_15m vs. GTP:PMA_AA_15m	ns	0.1602
GMP:PMA_6AN_15m vs. GTP:PMA_IAA_15m	****	<0.0001
GMP:PMA_6AN_15m vs. Guanosine:PMA_15m	ns	0.8028
GMP:PMA_6AN_15m vs. Guanosine:PMA_2DG_15m	ns	>0.9999
GMP:PMA_6AN_15m vs. Guanosine:PMA_6AN_15m	ns	>0.9999
GMP:PMA_6AN_15m vs. Guanosine:PMA_DPI_15m	ns	0.9868
GMP:PMA_6AN_15m vs. Guanosine:PMA_AA_15m	ns	>0.9999
GMP:PMA_6AN_15m vs. Guanosine:PMA_IAA_15m	**	0.0014
GMP:PMA_6AN_15m vs. Guanine:PMA_15m	**	0.0022
GMP:PMA_6AN_15m vs. Guanine:PMA_2DG_15m	ns	0.3338
GMP:PMA_6AN_15m vs. Guanine:PMA_6AN_15m	ns	0.341
GMP:PMA_6AN_15m vs. Guanine:PMA_DPI_15m	*	0.0153
GMP:PMA_6AN_15m vs. Guanine:PMA_AA_15m	ns	0.8152
GMP:PMA_6AN_15m vs. Guanine:PMA_IAA_15m	****	<0.0001
GMP:PMA_6AN_15m vs. Aspartic acid:PMA_15m	**	0.0066
GMP:PMA_6AN_15m vs. Aspartic acid:PMA_2DG_15m	ns	0.5591
GMP:PMA_6AN_15m vs. Aspartic acid:PMA_6AN_15m	ns	0.6013

GMP:PMA_6AN_15m vs. Aspartic acid:PMA_DPI_15m	*	0.0411
GMP:PMA_6AN_15m vs. Aspartic acid:PMA_AA_15m	ns	0.9483
GMP:PMA_6AN_15m vs. Aspartic acid:PMA_IAA_15m	****	<0.0001
GMP:PMA_6AN_15m vs. AMP:PMA_15m	ns	0.4562
GMP:PMA_6AN_15m vs. AMP:PMA_2DG_15m	ns	>0.9999
GMP:PMA_6AN_15m vs. AMP:PMA_6AN_15m	ns	>0.9999
GMP:PMA_6AN_15m vs. AMP:PMA_DPI_15m	ns	0.8502
GMP:PMA_6AN_15m vs. AMP:PMA_AA_15m	ns	>0.9999
GMP:PMA_6AN_15m vs. AMP:PMA_IAA_15m	***	0.0002
GMP:PMA_6AN_15m vs. ADP:PMA_15m	ns	0.2079
GMP:PMA_6AN_15m vs. ADP:PMA_2DG_15m	ns	0.9986
GMP:PMA_6AN_15m vs. ADP:PMA_6AN_15m	ns	0.9999
GMP:PMA_6AN_15m vs. ADP:PMA_DPI_15m	ns	0.5759
GMP:PMA_6AN_15m vs. ADP:PMA_AA_15m	ns	>0.9999
GMP:PMA_6AN_15m vs. ADP:PMA_IAA_15m	****	<0.0001
GMP:PMA_6AN_15m vs. ATP:PMA_15m	****	<0.0001
GMP:PMA_6AN_15m vs. ATP:PMA_2DG_15m	**	0.0063
GMP:PMA_6AN_15m vs. ATP:PMA_6AN_15m	**	0.0032
GMP:PMA_6AN_15m vs. ATP:PMA_DPI_15m	****	<0.0001
GMP:PMA_6AN_15m vs. ATP:PMA_AA_15m	ns	0.0552
GMP:PMA_6AN_15m vs. ATP:PMA_IAA_15m	****	<0.0001
GMP:PMA_6AN_15m vs. Xanthine:PMA_15m	**	0.0024
GMP:PMA_6AN_15m vs. Xanthine:PMA_2DG_15m	ns	0.3104
GMP:PMA_6AN_15m vs. Xanthine:PMA_6AN_15m	ns	0.3289
GMP:PMA_6AN_15m vs. Xanthine:PMA_DPI_15m	*	0.0156
GMP:PMA_6AN_15m vs. Xanthine:PMA_AA_15m	ns	0.7762
GMP:PMA_6AN_15m vs. CMP:PMA_15m	ns	0.5674
GMP:PMA_6AN_15m vs. CMP:PMA_2DG_15m	ns	>0.9999
GMP:PMA_6AN_15m vs. CMP:PMA_6AN_15m	ns	>0.9999
GMP:PMA_6AN_15m vs. CMP:PMA_DPI_15m	ns	0.9159
GMP:PMA_6AN_15m vs. CMP:PMA_AA_15m	ns	>0.9999
GMP:PMA_6AN_15m vs. CMP:PMA_IAA_15m	***	0.0004
GMP:PMA_6AN_15m vs. CDP:PMA_15m	ns	0.942
GMP:PMA_6AN_15m vs. CDP:PMA_2DG_15m	ns	>0.9999
GMP:PMA_6AN_15m vs. CDP:PMA_6AN_15m	ns	>0.9999
GMP:PMA_6AN_15m vs. CDP:PMA_DPI_15m	ns	0.9991
GMP:PMA_6AN_15m vs. CDP:PMA_AA_15m	ns	>0.9999
GMP:PMA_6AN_15m vs. CDP:PMA_IAA_15m	**	0.0042
GMP:PMA_6AN_15m vs. CTP:PMA_15m	***	0.0002
GMP:PMA_6AN_15m vs. CTP:PMA_2DG_15m	ns	0.0646
GMP:PMA_6AN_15m vs. CTP:PMA_6AN_15m	ns	0.0507
GMP:PMA_6AN_15m vs. CTP:PMA_DPI_15m	**	0.0014
GMP:PMA_6AN_15m vs. CTP:PMA_AA_15m	ns	0.33
GMP:PMA_6AN_15m vs. CTP:PMA_IAA_15m	****	<0.0001
GMP:PMA_6AN_15m vs. Uridine:PMA_15m	ns	0.7367
GMP:PMA_6AN_15m vs. Uridine:PMA_2DG_15m	ns	>0.9999
GMP:PMA_6AN_15m vs. Uridine:PMA_6AN_15m	ns	>0.9999

GMP:PMA_6AN_15m vs. Uridine:PMA_DPI_15m	ns	0.9743
GMP:PMA_6AN_15m vs. Uridine:PMA_AA_15m	ns	>0.9999
GMP:PMA_6AN_15m vs. Uridine:PMA_IAA_15m	***	0.0009
GMP:PMA_6AN_15m vs. UMP:PMA_15m	ns	0.9972
GMP:PMA_6AN_15m vs. UMP:PMA_2DG_15m	ns	>0.9999
GMP:PMA_6AN_15m vs. UMP:PMA_6AN_15m	ns	>0.9999
GMP:PMA_6AN_15m vs. UMP:PMA_DPI_15m	ns	>0.9999
GMP:PMA_6AN_15m vs. UMP:PMA_AA_15m	ns	>0.9999
GMP:PMA_6AN_15m vs. UMP:PMA_IAA_15m	*	0.019
GMP:PMA_6AN_15m vs. UDP:PMA_15m	ns	0.9965
GMP:PMA_6AN_15m vs. UDP:PMA_2DG_15m	ns	>0.9999
GMP:PMA_6AN_15m vs. UDP:PMA_6AN_15m	ns	>0.9999
GMP:PMA_6AN_15m vs. UDP:PMA_DPI_15m	ns	>0.9999
GMP:PMA_6AN_15m vs. UDP:PMA_AA_15m	ns	>0.9999
GMP:PMA_6AN_15m vs. UDP:PMA_IAA_15m	*	0.0177
GMP:PMA_6AN_15m vs. UTP:PMA_15m	****	<0.0001
GMP:PMA_6AN_15m vs. UTP:PMA_2DG_15m	*	0.0334
GMP:PMA_6AN_15m vs. UTP:PMA_6AN_15m	*	0.0233
GMP:PMA_6AN_15m vs. UTP:PMA_DPI_15m	***	0.0006
GMP:PMA_6AN_15m vs. UTP:PMA_AA_15m	ns	0.2068
GMP:PMA_6AN_15m vs. UTP:PMA_IAA_15m	****	<0.0001
GMP:PMA_DPI_15m vs. GMP:PMA_AA_15m	ns	0.5731
GMP:PMA_DPI_15m vs. GMP:PMA_IAA_15m	***	0.0004
GMP:PMA_DPI_15m vs. GDP:PMA_15m	ns	>0.9999
GMP:PMA_DPI_15m vs. GDP:PMA_2DG_15m	ns	>0.9999
GMP:PMA_DPI_15m vs. GDP:PMA_6AN_15m	ns	>0.9999
GMP:PMA_DPI_15m vs. GDP:PMA_DPI_15m	ns	>0.9999
GMP:PMA_DPI_15m vs. GDP:PMA_AA_15m	ns	>0.9999
GMP:PMA_DPI_15m vs. GDP:PMA_IAA_15m	ns	0.948
GMP:PMA_DPI_15m vs. GTP:PMA_15m	*	0.0207
GMP:PMA_DPI_15m vs. GTP:PMA_2DG_15m	ns	0.8026
GMP:PMA_DPI_15m vs. GTP:PMA_6AN_15m	ns	0.9822
GMP:PMA_DPI_15m vs. GTP:PMA_DPI_15m	*	0.0156
GMP:PMA_DPI_15m vs. GTP:PMA_AA_15m	ns	0.9944
GMP:PMA_DPI_15m vs. GTP:PMA_IAA_15m	****	<0.0001
GMP:PMA_DPI_15m vs. Guanosine:PMA_15m	ns	>0.9999
GMP:PMA_DPI_15m vs. Guanosine:PMA_2DG_15m	ns	>0.9999
GMP:PMA_DPI_15m vs. Guanosine:PMA_6AN_15m	ns	>0.9999
GMP:PMA_DPI_15m vs. Guanosine:PMA_DPI_15m	ns	>0.9999
GMP:PMA_DPI_15m vs. Guanosine:PMA_AA_15m	ns	>0.9999
GMP:PMA_DPI_15m vs. Guanosine:PMA_IAA_15m	ns	0.2341
GMP:PMA_DPI_15m vs. Guanine:PMA_15m	ns	0.3065
GMP:PMA_DPI_15m vs. Guanine:PMA_2DG_15m	ns	0.9998
GMP:PMA_DPI_15m vs. Guanine:PMA_6AN_15m	ns	>0.9999
GMP:PMA_DPI_15m vs. Guanine:PMA_DPI_15m	ns	0.341
GMP:PMA_DPI_15m vs. Guanine:PMA_AA_15m	ns	>0.9999
GMP:PMA_DPI_15m vs. Guanine:PMA_IAA_15m	****	<0.0001

GMP:PMA_DPI_15m vs. Aspartic acid:PMA_15m	ns	0.5257
GMP:PMA_DPI_15m vs. Aspartic acid:PMA_2DG_15m	ns	>0.9999
GMP:PMA_DPI_15m vs. Aspartic acid:PMA_6AN_15m	ns	>0.9999
GMP:PMA_DPI_15m vs. Aspartic acid:PMA_DPI_15m	ns	0.6013
GMP:PMA_DPI_15m vs. Aspartic acid:PMA_AA_15m	ns	>0.9999
GMP:PMA_DPI_15m vs. Aspartic acid:PMA_IAA_15m	***	0.0003
GMP:PMA_DPI_15m vs. AMP:PMA_15m	ns	>0.9999
GMP:PMA_DPI_15m vs. AMP:PMA_2DG_15m	ns	>0.9999
GMP:PMA_DPI_15m vs. AMP:PMA_6AN_15m	ns	>0.9999
GMP:PMA_DPI_15m vs. AMP:PMA_DPI_15m	ns	>0.9999
GMP:PMA_DPI_15m vs. AMP:PMA_AA_15m	ns	>0.9999
GMP:PMA_DPI_15m vs. AMP:PMA_IAA_15m	ns	0.0708
GMP:PMA_DPI_15m vs. ADP:PMA_15m	ns	0.9979
GMP:PMA_DPI_15m vs. ADP:PMA_2DG_15m	ns	>0.9999
GMP:PMA_DPI_15m vs. ADP:PMA_6AN_15m	ns	>0.9999
GMP:PMA_DPI_15m vs. ADP:PMA_DPI_15m	ns	0.9999
GMP:PMA_DPI_15m vs. ADP:PMA_AA_15m	ns	>0.9999
GMP:PMA_DPI_15m vs. ADP:PMA_IAA_15m	*	0.0211
GMP:PMA_DPI_15m vs. ATP:PMA_15m	**	0.0054
GMP:PMA_DPI_15m vs. ATP:PMA_2DG_15m	ns	0.515
GMP:PMA_DPI_15m vs. ATP:PMA_6AN_15m	ns	0.8678
GMP:PMA_DPI_15m vs. ATP:PMA_DPI_15m	**	0.0032
GMP:PMA_DPI_15m vs. ATP:PMA_AA_15m	ns	0.9315
GMP:PMA_DPI_15m vs. ATP:PMA_IAA_15m	****	<0.0001
GMP:PMA_DPI_15m vs. Xanthine:PMA_15m	ns	0.2852
GMP:PMA_DPI_15m vs. Xanthine:PMA_2DG_15m	ns	0.9994
GMP:PMA_DPI_15m vs. Xanthine:PMA_6AN_15m	ns	>0.9999
GMP:PMA_DPI_15m vs. Xanthine:PMA_DPI_15m	ns	0.3289
GMP:PMA_DPI_15m vs. Xanthine:PMA_AA_15m	ns	>0.9999
GMP:PMA_DPI_15m vs. CMP:PMA_15m	ns	>0.9999
GMP:PMA_DPI_15m vs. CMP:PMA_2DG_15m	ns	>0.9999
GMP:PMA_DPI_15m vs. CMP:PMA_6AN_15m	ns	>0.9999
GMP:PMA_DPI_15m vs. CMP:PMA_DPI_15m	ns	>0.9999
GMP:PMA_DPI_15m vs. CMP:PMA_AA_15m	ns	>0.9999
GMP:PMA_DPI_15m vs. CMP:PMA_IAA_15m	ns	0.1055
GMP:PMA_DPI_15m vs. CDP:PMA_15m	ns	>0.9999
GMP:PMA_DPI_15m vs. CDP:PMA_2DG_15m	ns	>0.9999
GMP:PMA_DPI_15m vs. CDP:PMA_6AN_15m	ns	>0.9999
GMP:PMA_DPI_15m vs. CDP:PMA_DPI_15m	ns	>0.9999
GMP:PMA_DPI_15m vs. CDP:PMA_AA_15m	ns	>0.9999
GMP:PMA_DPI_15m vs. CDP:PMA_IAA_15m	ns	0.4271
GMP:PMA_DPI_15m vs. CTP:PMA_15m	ns	0.057
GMP:PMA_DPI_15m vs. CTP:PMA_2DG_15m	ns	0.9476
GMP:PMA_DPI_15m vs. CTP:PMA_6AN_15m	ns	0.9989
GMP:PMA_DPI_15m vs. CTP:PMA_DPI_15m	ns	0.0507
GMP:PMA_DPI_15m vs. CTP:PMA_AA_15m	ns	0.9998
GMP:PMA_DPI_15m vs. CTP:PMA_IAA_15m	****	<0.0001

GMP:PMA_DPI_15m vs. Uridine:PMA_15m	ns	>0.9999
GMP:PMA_DPI_15m vs. Uridine:PMA_2DG_15m	ns	>0.9999
GMP:PMA_DPI_15m vs. Uridine:PMA_6AN_15m	ns	>0.9999
GMP:PMA_DPI_15m vs. Uridine:PMA_DPI_15m	ns	>0.9999
GMP:PMA_DPI_15m vs. Uridine:PMA_AA_15m	ns	>0.9999
GMP:PMA_DPI_15m vs. Uridine:PMA_IAA_15m	ns	0.1861
GMP:PMA_DPI_15m vs. UMP:PMA_15m	ns	>0.9999
GMP:PMA_DPI_15m vs. UMP:PMA_2DG_15m	ns	>0.9999
GMP:PMA_DPI_15m vs. UMP:PMA_6AN_15m	ns	>0.9999
GMP:PMA_DPI_15m vs. UMP:PMA_DPI_15m	ns	>0.9999
GMP:PMA_DPI_15m vs. UMP:PMA_AA_15m	ns	>0.9999
GMP:PMA_DPI_15m vs. UMP:PMA_IAA_15m	ns	0.7543
GMP:PMA_DPI_15m vs. UDP:PMA_15m	ns	>0.9999
GMP:PMA_DPI_15m vs. UDP:PMA_2DG_15m	ns	>0.9999
GMP:PMA_DPI_15m vs. UDP:PMA_6AN_15m	ns	>0.9999
GMP:PMA_DPI_15m vs. UDP:PMA_DPI_15m	ns	>0.9999
GMP:PMA_DPI_15m vs. UDP:PMA_AA_15m	ns	>0.9999
GMP:PMA_DPI_15m vs. UDP:PMA_IAA_15m	ns	0.7392
GMP:PMA_DPI_15m vs. UTP:PMA_15m	*	0.0292
GMP:PMA_DPI_15m vs. UTP:PMA_2DG_15m	ns	0.863
GMP:PMA_DPI_15m vs. UTP:PMA_6AN_15m	ns	0.9918
GMP:PMA_DPI_15m vs. UTP:PMA_DPI_15m	*	0.0233
GMP:PMA_DPI_15m vs. UTP:PMA_AA_15m	ns	0.9978
GMP:PMA_DPI_15m vs. UTP:PMA_IAA_15m	****	<0.0001
GMP:PMA_AA_15m vs. GMP:PMA_IAA_15m	****	<0.0001
GMP:PMA_AA_15m vs. GDP:PMA_15m	ns	0.9998
GMP:PMA_AA_15m vs. GDP:PMA_2DG_15m	ns	>0.9999
GMP:PMA_AA_15m vs. GDP:PMA_6AN_15m	ns	>0.9999
GMP:PMA_AA_15m vs. GDP:PMA_DPI_15m	ns	>0.9999
GMP:PMA_AA_15m vs. GDP:PMA_AA_15m	ns	>0.9999
GMP:PMA_AA_15m vs. GDP:PMA_IAA_15m	*	0.0417
GMP:PMA_AA_15m vs. GTP:PMA_15m	****	<0.0001
GMP:PMA_AA_15m vs. GTP:PMA_2DG_15m	*	0.0143
GMP:PMA_AA_15m vs. GTP:PMA_6AN_15m	ns	0.07
GMP:PMA_AA_15m vs. GTP:PMA_DPI_15m	***	0.0002
GMP:PMA_AA_15m vs. GTP:PMA_AA_15m	*	0.0156
GMP:PMA_AA_15m vs. GTP:PMA_IAA_15m	****	<0.0001
GMP:PMA_AA_15m vs. Guanosine:PMA_15m	ns	0.6972
GMP:PMA_AA_15m vs. Guanosine:PMA_2DG_15m	ns	>0.9999
GMP:PMA_AA_15m vs. Guanosine:PMA_6AN_15m	ns	>0.9999
GMP:PMA_AA_15m vs. Guanosine:PMA_DPI_15m	ns	0.9644
GMP:PMA_AA_15m vs. Guanosine:PMA_AA_15m	ns	>0.9999
GMP:PMA_AA_15m vs. Guanosine:PMA_IAA_15m	***	0.0008
GMP:PMA_AA_15m vs. Guanine:PMA_15m	**	0.0012
GMP:PMA_AA_15m vs. Guanine:PMA_2DG_15m	ns	0.242
GMP:PMA_AA_15m vs. Guanine:PMA_6AN_15m	ns	0.5949
GMP:PMA_AA_15m vs. Guanine:PMA_DPI_15m	**	0.009

GMP:PMA_AA_15m vs. Guanine:PMA_AA_15m	ns	0.341
GMP:PMA_AA_15m vs. Guanine:PMA_IAA_15m	****	<0.0001
GMP:PMA_AA_15m vs. Aspartic acid:PMA_15m	**	0.0038
GMP:PMA_AA_15m vs. Aspartic acid:PMA_2DG_15m	ns	0.4405
GMP:PMA_AA_15m vs. Aspartic acid:PMA_6AN_15m	ns	0.8139
GMP:PMA_AA_15m vs. Aspartic acid:PMA_DPI_15m	*	0.0253
GMP:PMA_AA_15m vs. Aspartic acid:PMA_AA_15m	ns	0.6013
GMP:PMA_AA_15m vs. Aspartic acid:PMA_IAA_15m	****	<0.0001
GMP:PMA_AA_15m vs. AMP:PMA_15m	ns	0.3462
GMP:PMA_AA_15m vs. AMP:PMA_2DG_15m	ns	>0.9999
GMP:PMA_AA_15m vs. AMP:PMA_6AN_15m	ns	>0.9999
GMP:PMA_AA_15m vs. AMP:PMA_DPI_15m	ns	0.7555
GMP:PMA_AA_15m vs. AMP:PMA_AA_15m	ns	>0.9999
GMP:PMA_AA_15m vs. AMP:PMA_IAA_15m	***	0.0001
GMP:PMA_AA_15m vs. ADP:PMA_15m	ns	0.1429
GMP:PMA_AA_15m vs. ADP:PMA_2DG_15m	ns	0.9943
GMP:PMA_AA_15m vs. ADP:PMA_6AN_15m	ns	>0.9999
GMP:PMA_AA_15m vs. ADP:PMA_DPI_15m	ns	0.4566
GMP:PMA_AA_15m vs. ADP:PMA_AA_15m	ns	0.9999
GMP:PMA_AA_15m vs. ADP:PMA_IAA_15m	****	<0.0001
GMP:PMA_AA_15m vs. ATP:PMA_15m	****	<0.0001
GMP:PMA_AA_15m vs. ATP:PMA_2DG_15m	**	0.0036
GMP:PMA_AA_15m vs. ATP:PMA_6AN_15m	*	0.021
GMP:PMA_AA_15m vs. ATP:PMA_DPI_15m	****	<0.0001
GMP:PMA_AA_15m vs. ATP:PMA_AA_15m	**	0.0032
GMP:PMA_AA_15m vs. ATP:PMA_IAA_15m	****	<0.0001
GMP:PMA_AA_15m vs. Xanthine:PMA_15m	**	0.0014
GMP:PMA_AA_15m vs. Xanthine:PMA_2DG_15m	ns	0.2259
GMP:PMA_AA_15m vs. Xanthine:PMA_6AN_15m	ns	0.5557
GMP:PMA_AA_15m vs. Xanthine:PMA_DPI_15m	**	0.0094
GMP:PMA_AA_15m vs. Xanthine:PMA_AA_15m	ns	0.3289
GMP:PMA_AA_15m vs. CMP:PMA_15m	ns	0.4484
GMP:PMA_AA_15m vs. CMP:PMA_2DG_15m	ns	>0.9999
GMP:PMA_AA_15m vs. CMP:PMA_6AN_15m	ns	>0.9999
GMP:PMA_AA_15m vs. CMP:PMA_DPI_15m	ns	0.8446
GMP:PMA_AA_15m vs. CMP:PMA_AA_15m	ns	>0.9999
GMP:PMA_AA_15m vs. CMP:PMA_IAA_15m	***	0.0002
GMP:PMA_AA_15m vs. CDP:PMA_15m	ns	0.8842
GMP:PMA_AA_15m vs. CDP:PMA_2DG_15m	ns	>0.9999
GMP:PMA_AA_15m vs. CDP:PMA_6AN_15m	ns	>0.9999
GMP:PMA_AA_15m vs. CDP:PMA_DPI_15m	ns	0.996
GMP:PMA_AA_15m vs. CDP:PMA_AA_15m	ns	>0.9999
GMP:PMA_AA_15m vs. CDP:PMA_IAA_15m	**	0.0024
GMP:PMA_AA_15m vs. CTP:PMA_15m	****	<0.0001
GMP:PMA_AA_15m vs. CTP:PMA_2DG_15m	*	0.0407
GMP:PMA_AA_15m vs. CTP:PMA_6AN_15m	ns	0.1665
GMP:PMA_AA_15m vs. CTP:PMA_DPI_15m	***	0.0008

GMP:PMA_AA_15m vs. CTP:PMA_AA_15m	ns	0.0507
GMP:PMA_AA_15m vs. CTP:PMA_IAA_15m	****	<0.0001
GMP:PMA_AA_15m vs. Uridine:PMA_15m	ns	0.6217
GMP:PMA_AA_15m vs. Uridine:PMA_2DG_15m	ns	>0.9999
GMP:PMA_AA_15m vs. Uridine:PMA_6AN_15m	ns	>0.9999
GMP:PMA_AA_15m vs. Uridine:PMA_DPI_15m	ns	0.9394
GMP:PMA_AA_15m vs. Uridine:PMA_AA_15m	ns	>0.9999
GMP:PMA_AA_15m vs. Uridine:PMA_IAA_15m	***	0.0005
GMP:PMA_AA_15m vs. UMP:PMA_15m	ns	0.9897
GMP:PMA_AA_15m vs. UMP:PMA_2DG_15m	ns	>0.9999
GMP:PMA_AA_15m vs. UMP:PMA_6AN_15m	ns	>0.9999
GMP:PMA_AA_15m vs. UMP:PMA_DPI_15m	ns	>0.9999
GMP:PMA_AA_15m vs. UMP:PMA_AA_15m	ns	>0.9999
GMP:PMA_AA_15m vs. UMP:PMA_IAA_15m	*	0.0113
GMP:PMA_AA_15m vs. UDP:PMA_15m	ns	0.9879
GMP:PMA_AA_15m vs. UDP:PMA_2DG_15m	ns	>0.9999
GMP:PMA_AA_15m vs. UDP:PMA_6AN_15m	ns	>0.9999
GMP:PMA_AA_15m vs. UDP:PMA_DPI_15m	ns	>0.9999
GMP:PMA_AA_15m vs. UDP:PMA_AA_15m	ns	>0.9999
GMP:PMA_AA_15m vs. UDP:PMA_IAA_15m	*	0.0105
GMP:PMA_AA_15m vs. UTP:PMA_15m	****	<0.0001
GMP:PMA_AA_15m vs. UTP:PMA_2DG_15m	*	0.0204
GMP:PMA_AA_15m vs. UTP:PMA_6AN_15m	ns	0.0945
GMP:PMA_AA_15m vs. UTP:PMA_DPI_15m	***	0.0003
GMP:PMA_AA_15m vs. UTP:PMA_AA_15m	*	0.0233
GMP:PMA_AA_15m vs. UTP:PMA_IAA_15m	****	<0.0001
GMP:PMA_IAA_15m vs. GDP:PMA_15m	ns	>0.9999
GMP:PMA_IAA_15m vs. GDP:PMA_2DG_15m	ns	0.3379
GMP:PMA_IAA_15m vs. GDP:PMA_6AN_15m	ns	0.1036
GMP:PMA_IAA_15m vs. GDP:PMA_DPI_15m	ns	0.9797
GMP:PMA_IAA_15m vs. GDP:PMA_AA_15m	ns	0.0674
GMP:PMA_IAA_15m vs. GDP:PMA_IAA_15m	ns	>0.9999
GMP:PMA_IAA_15m vs. GTP:PMA_15m	ns	>0.9999
GMP:PMA_IAA_15m vs. GTP:PMA_2DG_15m	ns	>0.9999
GMP:PMA_IAA_15m vs. GTP:PMA_6AN_15m	ns	>0.9999
GMP:PMA_IAA_15m vs. GTP:PMA_DPI_15m	ns	>0.9999
GMP:PMA_IAA_15m vs. GTP:PMA_AA_15m	ns	>0.9999
GMP:PMA_IAA_15m vs. GTP:PMA_IAA_15m	*	0.0156
GMP:PMA_IAA_15m vs. Guanosine:PMA_15m	ns	>0.9999
GMP:PMA_IAA_15m vs. Guanosine:PMA_2DG_15m	ns	0.9816
GMP:PMA_IAA_15m vs. Guanosine:PMA_6AN_15m	ns	0.8006
GMP:PMA_IAA_15m vs. Guanosine:PMA_DPI_15m	ns	>0.9999
GMP:PMA_IAA_15m vs. Guanosine:PMA_AA_15m	ns	0.6951
GMP:PMA_IAA_15m vs. Guanosine:PMA_IAA_15m	ns	>0.9999
GMP:PMA_IAA_15m vs. Guanine:PMA_15m	ns	>0.9999
GMP:PMA_IAA_15m vs. Guanine:PMA_2DG_15m	ns	>0.9999
GMP:PMA_IAA_15m vs. Guanine:PMA_6AN_15m	ns	>0.9999

GMP:PMA_IAA_15m vs. Guanine:PMA_DPI_15m	ns	>0.9999
GMP:PMA_IAA_15m vs. Guanine:PMA_AA_15m	ns	>0.9999
GMP:PMA_IAA_15m vs. Guanine:PMA_IAA_15m	ns	0.341
GMP:PMA_IAA_15m vs. Aspartic acid:PMA_15m	ns	>0.9999
GMP:PMA_IAA_15m vs. Aspartic acid:PMA_2DG_15m	ns	>0.9999
GMP:PMA_IAA_15m vs. Aspartic acid:PMA_6AN_15m	ns	>0.9999
GMP:PMA_IAA_15m vs. Aspartic acid:PMA_DPI_15m	ns	>0.9999
GMP:PMA_IAA_15m vs. Aspartic acid:PMA_AA_15m	ns	>0.9999
GMP:PMA_IAA_15m vs. Aspartic acid:PMA_IAA_15m	ns	0.6013
GMP:PMA_IAA_15m vs. AMP:PMA_15m	ns	>0.9999
GMP:PMA_IAA_15m vs. AMP:PMA_2DG_15m	ns	0.9998
GMP:PMA_IAA_15m vs. AMP:PMA_6AN_15m	ns	0.9762
GMP:PMA_IAA_15m vs. AMP:PMA_DPI_15m	ns	>0.9999
GMP:PMA_IAA_15m vs. AMP:PMA_AA_15m	ns	0.9432
GMP:PMA_IAA_15m vs. AMP:PMA_IAA_15m	ns	>0.9999
GMP:PMA_IAA_15m vs. ADP:PMA_15m	ns	>0.9999
GMP:PMA_IAA_15m vs. ADP:PMA_2DG_15m	ns	>0.9999
GMP:PMA_IAA_15m vs. ADP:PMA_6AN_15m	ns	0.9991
GMP:PMA_IAA_15m vs. ADP:PMA_DPI_15m	ns	>0.9999
GMP:PMA_IAA_15m vs. ADP:PMA_AA_15m	ns	0.9961
GMP:PMA_IAA_15m vs. ADP:PMA_IAA_15m	ns	0.9999
GMP:PMA_IAA_15m vs. ATP:PMA_15m	ns	0.9997
GMP:PMA_IAA_15m vs. ATP:PMA_2DG_15m	ns	>0.9999
GMP:PMA_IAA_15m vs. ATP:PMA_6AN_15m	ns	>0.9999
GMP:PMA_IAA_15m vs. ATP:PMA_DPI_15m	ns	>0.9999
GMP:PMA_IAA_15m vs. ATP:PMA_AA_15m	ns	>0.9999
GMP:PMA_IAA_15m vs. ATP:PMA_IAA_15m	**	0.0032
GMP:PMA_IAA_15m vs. Xanthine:PMA_15m	ns	>0.9999
GMP:PMA_IAA_15m vs. Xanthine:PMA_2DG_15m	ns	>0.9999
GMP:PMA_IAA_15m vs. Xanthine:PMA_6AN_15m	ns	>0.9999
GMP:PMA_IAA_15m vs. Xanthine:PMA_DPI_15m	ns	>0.9999
GMP:PMA_IAA_15m vs. Xanthine:PMA_AA_15m	ns	>0.9999
GMP:PMA_IAA_15m vs. CMP:PMA_15m	ns	>0.9999
GMP:PMA_IAA_15m vs. CMP:PMA_2DG_15m	ns	0.9988
GMP:PMA_IAA_15m vs. CMP:PMA_6AN_15m	ns	0.9462
GMP:PMA_IAA_15m vs. CMP:PMA_DPI_15m	ns	>0.9999
GMP:PMA_IAA_15m vs. CMP:PMA_AA_15m	ns	0.891
GMP:PMA_IAA_15m vs. CMP:PMA_IAA_15m	ns	>0.9999
GMP:PMA_IAA_15m vs. CDP:PMA_15m	ns	>0.9999
GMP:PMA_IAA_15m vs. CDP:PMA_2DG_15m	ns	0.905
GMP:PMA_IAA_15m vs. CDP:PMA_6AN_15m	ns	0.5805
GMP:PMA_IAA_15m vs. CDP:PMA_DPI_15m	ns	>0.9999
GMP:PMA_IAA_15m vs. CDP:PMA_AA_15m	ns	0.4614
GMP:PMA_IAA_15m vs. CDP:PMA_IAA_15m	ns	>0.9999
GMP:PMA_IAA_15m vs. CTP:PMA_15m	ns	>0.9999
GMP:PMA_IAA_15m vs. CTP:PMA_2DG_15m	ns	>0.9999
GMP:PMA_IAA_15m vs. CTP:PMA_6AN_15m	ns	>0.9999

GMP:PMA_IAA_15m vs. CTP:PMA_DPI_15m	ns	>0.9999
GMP:PMA_IAA_15m vs. CTP:PMA_AA_15m	ns	>0.9999
GMP:PMA_IAA_15m vs. CTP:PMA_IAA_15m	ns	0.0507
GMP:PMA_IAA_15m vs. Uridine:PMA_15m	ns	>0.9999
GMP:PMA_IAA_15m vs. Uridine:PMA_2DG_15m	ns	0.9909
GMP:PMA_IAA_15m vs. Uridine:PMA_6AN_15m	ns	0.8575
GMP:PMA_IAA_15m vs. Uridine:PMA_DPI_15m	ns	>0.9999
GMP:PMA_IAA_15m vs. Uridine:PMA_AA_15m	ns	0.7653
GMP:PMA_IAA_15m vs. Uridine:PMA_IAA_15m	ns	>0.9999
GMP:PMA_IAA_15m vs. UMP:PMA_15m	ns	>0.9999
GMP:PMA_IAA_15m vs. UMP:PMA_2DG_15m	ns	0.6368
GMP:PMA_IAA_15m vs. UMP:PMA_6AN_15m	ns	0.2731
GMP:PMA_IAA_15m vs. UMP:PMA_DPI_15m	ns	0.9994
GMP:PMA_IAA_15m vs. UMP:PMA_AA_15m	ns	0.1934
GMP:PMA_IAA_15m vs. UMP:PMA_IAA_15m	ns	>0.9999
GMP:PMA_IAA_15m vs. UDP:PMA_15m	ns	>0.9999
GMP:PMA_IAA_15m vs. UDP:PMA_2DG_15m	ns	0.6535
GMP:PMA_IAA_15m vs. UDP:PMA_6AN_15m	ns	0.2859
GMP:PMA_IAA_15m vs. UDP:PMA_DPI_15m	ns	0.9995
GMP:PMA_IAA_15m vs. UDP:PMA_AA_15m	ns	0.2036
GMP:PMA_IAA_15m vs. UDP:PMA_IAA_15m	ns	>0.9999
GMP:PMA_IAA_15m vs. UTP:PMA_15m	ns	>0.9999
GMP:PMA_IAA_15m vs. UTP:PMA_2DG_15m	ns	>0.9999
GMP:PMA_IAA_15m vs. UTP:PMA_6AN_15m	ns	>0.9999
GMP:PMA_IAA_15m vs. UTP:PMA_DPI_15m	ns	>0.9999
GMP:PMA_IAA_15m vs. UTP:PMA_AA_15m	ns	>0.9999
GMP:PMA_IAA_15m vs. UTP:PMA_IAA_15m	*	0.0233
GDP:PMA_15m vs. GDP:PMA_2DG_15m	ns	0.7475
GDP:PMA_15m vs. GDP:PMA_6AN_15m	ns	0.1037
GDP:PMA_15m vs. GDP:PMA_DPI_15m	ns	>0.9999
GDP:PMA_15m vs. GDP:PMA_AA_15m	*	0.0409
GDP:PMA_15m vs. GDP:PMA_IAA_15m	*	0.0236
GDP:PMA_15m vs. GTP:PMA_15m	*	0.0212
GDP:PMA_15m vs. GTP:PMA_2DG_15m	ns	0.9928
GDP:PMA_15m vs. GTP:PMA_6AN_15m	ns	>0.9999
GDP:PMA_15m vs. GTP:PMA_DPI_15m	ns	0.4356
GDP:PMA_15m vs. GTP:PMA_AA_15m	ns	>0.9999
GDP:PMA_15m vs. GTP:PMA_IAA_15m	****	<0.0001
GDP:PMA_15m vs. Guanosine:PMA_15m	ns	>0.9999
GDP:PMA_15m vs. Guanosine:PMA_2DG_15m	ns	>0.9999
GDP:PMA_15m vs. Guanosine:PMA_6AN_15m	ns	>0.9999
GDP:PMA_15m vs. Guanosine:PMA_DPI_15m	ns	>0.9999
GDP:PMA_15m vs. Guanosine:PMA_AA_15m	ns	>0.9999
GDP:PMA_15m vs. Guanosine:PMA_IAA_15m	ns	0.6767
GDP:PMA_15m vs. Guanine:PMA_15m	ns	0.4046
GDP:PMA_15m vs. Guanine:PMA_2DG_15m	ns	>0.9999
GDP:PMA_15m vs. Guanine:PMA_6AN_15m	ns	>0.9999

GDP:PMA_15m vs. Guanine:PMA_DPI_15m	ns	0.9809
GDP:PMA_15m vs. Guanine:PMA_AA_15m	ns	>0.9999
GDP:PMA_15m vs. Guanine:PMA_IAA_15m	**	0.0011
GDP:PMA_15m vs. Aspartic acid:PMA_15m	ns	0.6721
GDP:PMA_15m vs. Aspartic acid:PMA_2DG_15m	ns	>0.9999
GDP:PMA_15m vs. Aspartic acid:PMA_6AN_15m	ns	>0.9999
GDP:PMA_15m vs. Aspartic acid:PMA_DPI_15m	ns	0.9985
GDP:PMA_15m vs. Aspartic acid:PMA_AA_15m	ns	>0.9999
GDP:PMA_15m vs. Aspartic acid:PMA_IAA_15m	**	0.0035
GDP:PMA_15m vs. AMP:PMA_15m	ns	>0.9999
GDP:PMA_15m vs. AMP:PMA_2DG_15m	ns	>0.9999
GDP:PMA_15m vs. AMP:PMA_6AN_15m	ns	>0.9999
GDP:PMA_15m vs. AMP:PMA_DPI_15m	ns	>0.9999
GDP:PMA_15m vs. AMP:PMA_AA_15m	ns	>0.9999
GDP:PMA_15m vs. AMP:PMA_IAA_15m	ns	0.3292
GDP:PMA_15m vs. ADP:PMA_15m	ns	>0.9999
GDP:PMA_15m vs. ADP:PMA_2DG_15m	ns	>0.9999
GDP:PMA_15m vs. ADP:PMA_6AN_15m	ns	>0.9999
GDP:PMA_15m vs. ADP:PMA_DPI_15m	ns	>0.9999
GDP:PMA_15m vs. ADP:PMA_AA_15m	ns	>0.9999
GDP:PMA_15m vs. ADP:PMA_IAA_15m	ns	0.1342
GDP:PMA_15m vs. ATP:PMA_15m	**	0.0045
GDP:PMA_15m vs. ATP:PMA_2DG_15m	ns	0.9209
GDP:PMA_15m vs. ATP:PMA_6AN_15m	ns	0.9974
GDP:PMA_15m vs. ATP:PMA_DPI_15m	ns	0.1968
GDP:PMA_15m vs. ATP:PMA_AA_15m	ns	0.9995
GDP:PMA_15m vs. ATP:PMA_IAA_15m	****	<0.0001
GDP:PMA_15m vs. Xanthine:PMA_15m	ns	0.3882
GDP:PMA_15m vs. Xanthine:PMA_2DG_15m	ns	>0.9999
GDP:PMA_15m vs. Xanthine:PMA_6AN_15m	ns	>0.9999
GDP:PMA_15m vs. Xanthine:PMA_DPI_15m	ns	0.9685
GDP:PMA_15m vs. Xanthine:PMA_AA_15m	ns	>0.9999
GDP:PMA_15m vs. CMP:PMA_15m	ns	>0.9999
GDP:PMA_15m vs. CMP:PMA_2DG_15m	ns	>0.9999
GDP:PMA_15m vs. CMP:PMA_6AN_15m	ns	>0.9999
GDP:PMA_15m vs. CMP:PMA_DPI_15m	ns	>0.9999
GDP:PMA_15m vs. CMP:PMA_AA_15m	ns	>0.9999
GDP:PMA_15m vs. CMP:PMA_IAA_15m	ns	0.4288
GDP:PMA_15m vs. CDP:PMA_15m	ns	>0.9999
GDP:PMA_15m vs. CDP:PMA_2DG_15m	ns	>0.9999
GDP:PMA_15m vs. CDP:PMA_6AN_15m	ns	>0.9999
GDP:PMA_15m vs. CDP:PMA_DPI_15m	ns	>0.9999
GDP:PMA_15m vs. CDP:PMA_AA_15m	ns	>0.9999
GDP:PMA_15m vs. CDP:PMA_IAA_15m	ns	0.8705
GDP:PMA_15m vs. CTP:PMA_15m	ns	0.0664
GDP:PMA_15m vs. CTP:PMA_2DG_15m	ns	0.9997
GDP:PMA_15m vs. CTP:PMA_6AN_15m	ns	>0.9999

GDP:PMA_15m vs. CTP:PMA_DPI_15m	ns	0.6851
GDP:PMA_15m vs. CTP:PMA_AA_15m	ns	>0.9999
GDP:PMA_15m vs. CTP:PMA_IAA_15m	****	<0.0001
GDP:PMA_15m vs. Uridine:PMA_15m	ns	>0.9999
GDP:PMA_15m vs. Uridine:PMA_2DG_15m	ns	>0.9999
GDP:PMA_15m vs. Uridine:PMA_6AN_15m	ns	>0.9999
GDP:PMA_15m vs. Uridine:PMA_DPI_15m	ns	>0.9999
GDP:PMA_15m vs. Uridine:PMA_AA_15m	ns	>0.9999
GDP:PMA_15m vs. Uridine:PMA_IAA_15m	ns	0.6004
GDP:PMA_15m vs. UMP:PMA_15m	ns	>0.9999
GDP:PMA_15m vs. UMP:PMA_2DG_15m	ns	>0.9999
GDP:PMA_15m vs. UMP:PMA_6AN_15m	ns	>0.9999
GDP:PMA_15m vs. UMP:PMA_DPI_15m	ns	>0.9999
GDP:PMA_15m vs. UMP:PMA_AA_15m	ns	>0.9999
GDP:PMA_15m vs. UMP:PMA_IAA_15m	ns	0.9872
GDP:PMA_15m vs. UDP:PMA_15m	ns	>0.9999
GDP:PMA_15m vs. UDP:PMA_2DG_15m	ns	>0.9999
GDP:PMA_15m vs. UDP:PMA_6AN_15m	ns	>0.9999
GDP:PMA_15m vs. UDP:PMA_DPI_15m	ns	>0.9999
GDP:PMA_15m vs. UDP:PMA_AA_15m	ns	>0.9999
GDP:PMA_15m vs. UDP:PMA_IAA_15m	ns	0.985
GDP:PMA_15m vs. UTP:PMA_15m	*	0.0313
GDP:PMA_15m vs. UTP:PMA_2DG_15m	ns	0.9971
GDP:PMA_15m vs. UTP:PMA_6AN_15m	ns	>0.9999
GDP:PMA_15m vs. UTP:PMA_DPI_15m	ns	0.5168
GDP:PMA_15m vs. UTP:PMA_AA_15m	ns	>0.9999
GDP:PMA_15m vs. UTP:PMA_IAA_15m	****	<0.0001
GDP:PMA_2DG_15m vs. GDP:PMA_6AN_15m	ns	>0.9999
GDP:PMA_2DG_15m vs. GDP:PMA_DPI_15m	ns	>0.9999
GDP:PMA_2DG_15m vs. GDP:PMA_AA_15m	ns	>0.9999
GDP:PMA_2DG_15m vs. GDP:PMA_IAA_15m	****	<0.0001
GDP:PMA_2DG_15m vs. GTP:PMA_15m	***	0.0005
GDP:PMA_2DG_15m vs. GTP:PMA_2DG_15m	*	0.0212
GDP:PMA_2DG_15m vs. GTP:PMA_6AN_15m	ns	0.4044
GDP:PMA_2DG_15m vs. GTP:PMA_DPI_15m	**	0.0037
GDP:PMA_2DG_15m vs. GTP:PMA_AA_15m	ns	0.5205
GDP:PMA_2DG_15m vs. GTP:PMA_IAA_15m	****	<0.0001
GDP:PMA_2DG_15m vs. Guanosine:PMA_15m	ns	0.99
GDP:PMA_2DG_15m vs. Guanosine:PMA_2DG_15m	ns	>0.9999
GDP:PMA_2DG_15m vs. Guanosine:PMA_6AN_15m	ns	>0.9999
GDP:PMA_2DG_15m vs. Guanosine:PMA_DPI_15m	ns	>0.9999
GDP:PMA_2DG_15m vs. Guanosine:PMA_AA_15m	ns	>0.9999
GDP:PMA_2DG_15m vs. Guanosine:PMA_IAA_15m	*	0.0115
GDP:PMA_2DG_15m vs. Guanine:PMA_15m	*	0.0174
GDP:PMA_2DG_15m vs. Guanine:PMA_2DG_15m	ns	0.4046
GDP:PMA_2DG_15m vs. Guanine:PMA_6AN_15m	ns	0.9749
GDP:PMA_2DG_15m vs. Guanine:PMA_DPI_15m	ns	0.0931

GDP:PMA_2DG_15m vs. Guanine:PMA_AA_15m	ns	0.9914
GDP:PMA_2DG_15m vs. Guanine:PMA_IAA_15m	****	<0.0001
GDP:PMA_2DG_15m vs. Aspartic acid:PMA_15m	*	0.0461
GDP:PMA_2DG_15m vs. Aspartic acid:PMA_2DG_15m	ns	0.6721
GDP:PMA_2DG_15m vs. Aspartic acid:PMA_6AN_15m	ns	0.9977
GDP:PMA_2DG_15m vs. Aspartic acid:PMA_DPI_15m	ns	0.2032
GDP:PMA_2DG_15m vs. Aspartic acid:PMA_AA_15m	ns	0.9995
GDP:PMA_2DG_15m vs. Aspartic acid:PMA_IAA_15m	****	<0.0001
GDP:PMA_2DG_15m vs. AMP:PMA_15m	ns	0.8698
GDP:PMA_2DG_15m vs. AMP:PMA_2DG_15m	ns	>0.9999
GDP:PMA_2DG_15m vs. AMP:PMA_6AN_15m	ns	>0.9999
GDP:PMA_2DG_15m vs. AMP:PMA_DPI_15m	ns	0.9948
GDP:PMA_2DG_15m vs. AMP:PMA_AA_15m	ns	>0.9999
GDP:PMA_2DG_15m vs. AMP:PMA_IAA_15m	**	0.0021
GDP:PMA_2DG_15m vs. ADP:PMA_15m	ns	0.6056
GDP:PMA_2DG_15m vs. ADP:PMA_2DG_15m	ns	>0.9999
GDP:PMA_2DG_15m vs. ADP:PMA_6AN_15m	ns	>0.9999
GDP:PMA_2DG_15m vs. ADP:PMA_DPI_15m	ns	0.933
GDP:PMA_2DG_15m vs. ADP:PMA_AA_15m	ns	>0.9999
GDP:PMA_2DG_15m vs. ADP:PMA_IAA_15m	***	0.0005
GDP:PMA_2DG_15m vs. ATP:PMA_15m	****	<0.0001
GDP:PMA_2DG_15m vs. ATP:PMA_2DG_15m	**	0.0045
GDP:PMA_2DG_15m vs. ATP:PMA_6AN_15m	ns	0.1777
GDP:PMA_2DG_15m vs. ATP:PMA_DPI_15m	***	0.0008
GDP:PMA_2DG_15m vs. ATP:PMA_AA_15m	ns	0.2534
GDP:PMA_2DG_15m vs. ATP:PMA_IAA_15m	****	<0.0001
GDP:PMA_2DG_15m vs. Xanthine:PMA_15m	*	0.0177
GDP:PMA_2DG_15m vs. Xanthine:PMA_2DG_15m	ns	0.3882
GDP:PMA_2DG_15m vs. Xanthine:PMA_6AN_15m	ns	0.9601
GDP:PMA_2DG_15m vs. Xanthine:PMA_DPI_15m	ns	0.0891
GDP:PMA_2DG_15m vs. Xanthine:PMA_AA_15m	ns	0.9841
GDP:PMA_2DG_15m vs. CMP:PMA_15m	ns	0.9294
GDP:PMA_2DG_15m vs. CMP:PMA_2DG_15m	ns	>0.9999
GDP:PMA_2DG_15m vs. CMP:PMA_6AN_15m	ns	>0.9999
GDP:PMA_2DG_15m vs. CMP:PMA_DPI_15m	ns	0.9986
GDP:PMA_2DG_15m vs. CMP:PMA_AA_15m	ns	>0.9999
GDP:PMA_2DG_15m vs. CMP:PMA_IAA_15m	**	0.0037
GDP:PMA_2DG_15m vs. CDP:PMA_15m	ns	0.9994
GDP:PMA_2DG_15m vs. CDP:PMA_2DG_15m	ns	>0.9999
GDP:PMA_2DG_15m vs. CDP:PMA_6AN_15m	ns	>0.9999
GDP:PMA_2DG_15m vs. CDP:PMA_DPI_15m	ns	>0.9999
GDP:PMA_2DG_15m vs. CDP:PMA_AA_15m	ns	>0.9999
GDP:PMA_2DG_15m vs. CDP:PMA_IAA_15m	*	0.0312
GDP:PMA_2DG_15m vs. CTP:PMA_15m	**	0.0016
GDP:PMA_2DG_15m vs. CTP:PMA_2DG_15m	ns	0.0664
GDP:PMA_2DG_15m vs. CTP:PMA_6AN_15m	ns	0.6528
GDP:PMA_2DG_15m vs. CTP:PMA_DPI_15m	*	0.0117

GDP:PMA_2DG_15m vs. CTP:PMA_AA_15m	ns	0.7645
GDP:PMA_2DG_15m vs. CTP:PMA_IAA_15m	****	<0.0001
GDP:PMA_2DG_15m vs. Uridine:PMA_15m	ns	0.9798
GDP:PMA_2DG_15m vs. Uridine:PMA_2DG_15m	ns	>0.9999
GDP:PMA_2DG_15m vs. Uridine:PMA_6AN_15m	ns	>0.9999
GDP:PMA_2DG_15m vs. Uridine:PMA_DPI_15m	ns	>0.9999
GDP:PMA_2DG_15m vs. Uridine:PMA_AA_15m	ns	>0.9999
GDP:PMA_2DG_15m vs. Uridine:PMA_IAA_15m	**	0.0081
GDP:PMA_2DG_15m vs. UMP:PMA_15m	ns	>0.9999
GDP:PMA_2DG_15m vs. UMP:PMA_2DG_15m	ns	>0.9999
GDP:PMA_2DG_15m vs. UMP:PMA_6AN_15m	ns	>0.9999
GDP:PMA_2DG_15m vs. UMP:PMA_DPI_15m	ns	>0.9999
GDP:PMA_2DG_15m vs. UMP:PMA_AA_15m	ns	>0.9999
GDP:PMA_2DG_15m vs. UMP:PMA_IAA_15m	ns	0.1106
GDP:PMA_2DG_15m vs. UDP:PMA_15m	ns	>0.9999
GDP:PMA_2DG_15m vs. UDP:PMA_2DG_15m	ns	>0.9999
GDP:PMA_2DG_15m vs. UDP:PMA_6AN_15m	ns	>0.9999
GDP:PMA_2DG_15m vs. UDP:PMA_DPI_15m	ns	>0.9999
GDP:PMA_2DG_15m vs. UDP:PMA_AA_15m	ns	>0.9999
GDP:PMA_2DG_15m vs. UDP:PMA_IAA_15m	ns	0.1043
GDP:PMA_2DG_15m vs. UTP:PMA_15m	***	0.0007
GDP:PMA_2DG_15m vs. UTP:PMA_2DG_15m	*	0.0313
GDP:PMA_2DG_15m vs. UTP:PMA_6AN_15m	ns	0.4838
GDP:PMA_2DG_15m vs. UTP:PMA_DPI_15m	**	0.0054
GDP:PMA_2DG_15m vs. UTP:PMA_AA_15m	ns	0.6039
GDP:PMA_2DG_15m vs. UTP:PMA_IAA_15m	****	<0.0001
GDP:PMA_6AN_15m vs. GDP:PMA_DPI_15m	ns	0.8059
GDP:PMA_6AN_15m vs. GDP:PMA_AA_15m	ns	>0.9999
GDP:PMA_6AN_15m vs. GDP:PMA_IAA_15m	****	<0.0001
GDP:PMA_6AN_15m vs. GTP:PMA_15m	****	<0.0001
GDP:PMA_6AN_15m vs. GTP:PMA_2DG_15m	*	0.0308
GDP:PMA_6AN_15m vs. GTP:PMA_6AN_15m	*	0.0212
GDP:PMA_6AN_15m vs. GTP:PMA_DPI_15m	***	0.0005
GDP:PMA_6AN_15m vs. GTP:PMA_AA_15m	ns	0.1947
GDP:PMA_6AN_15m vs. GTP:PMA_IAA_15m	****	<0.0001
GDP:PMA_6AN_15m vs. Guanosine:PMA_15m	ns	0.8496
GDP:PMA_6AN_15m vs. Guanosine:PMA_2DG_15m	ns	>0.9999
GDP:PMA_6AN_15m vs. Guanosine:PMA_6AN_15m	ns	>0.9999
GDP:PMA_6AN_15m vs. Guanosine:PMA_DPI_15m	ns	0.9928
GDP:PMA_6AN_15m vs. Guanosine:PMA_AA_15m	ns	>0.9999
GDP:PMA_6AN_15m vs. Guanosine:PMA_IAA_15m	**	0.0019
GDP:PMA_6AN_15m vs. Guanine:PMA_15m	**	0.0029
GDP:PMA_6AN_15m vs. Guanine:PMA_2DG_15m	ns	0.3887
GDP:PMA_6AN_15m vs. Guanine:PMA_6AN_15m	ns	0.4046
GDP:PMA_6AN_15m vs. Guanine:PMA_DPI_15m	*	0.02
GDP:PMA_6AN_15m vs. Guanine:PMA_AA_15m	ns	0.8603
GDP:PMA_6AN_15m vs. Guanine:PMA_IAA_15m	****	<0.0001

GDP:PMA_6AN_15m vs. Aspartic acid:PMA_15m	**	0.0088
GDP:PMA_6AN_15m vs. Aspartic acid:PMA_2DG_15m	ns	0.6221
GDP:PMA_6AN_15m vs. Aspartic acid:PMA_6AN_15m	ns	0.6721
GDP:PMA_6AN_15m vs. Aspartic acid:PMA_DPI_15m	ns	0.0524
GDP:PMA_6AN_15m vs. Aspartic acid:PMA_AA_15m	ns	0.9669
GDP:PMA_6AN_15m vs. Aspartic acid:PMA_IAA_15m	****	<0.0001
GDP:PMA_6AN_15m vs. AMP:PMA_15m	ns	0.5181
GDP:PMA_6AN_15m vs. AMP:PMA_2DG_15m	ns	>0.9999
GDP:PMA_6AN_15m vs. AMP:PMA_6AN_15m	ns	>0.9999
GDP:PMA_6AN_15m vs. AMP:PMA_DPI_15m	ns	0.8899
GDP:PMA_6AN_15m vs. AMP:PMA_AA_15m	ns	>0.9999
GDP:PMA_6AN_15m vs. AMP:PMA_IAA_15m	***	0.0003
GDP:PMA_6AN_15m vs. ADP:PMA_15m	ns	0.2494
GDP:PMA_6AN_15m vs. ADP:PMA_2DG_15m	ns	0.9994
GDP:PMA_6AN_15m vs. ADP:PMA_6AN_15m	ns	>0.9999
GDP:PMA_6AN_15m vs. ADP:PMA_DPI_15m	ns	0.6387
GDP:PMA_6AN_15m vs. ADP:PMA_AA_15m	ns	>0.9999
GDP:PMA_6AN_15m vs. ADP:PMA_IAA_15m	****	<0.0001
GDP:PMA_6AN_15m vs. ATP:PMA_15m	****	<0.0001
GDP:PMA_6AN_15m vs. ATP:PMA_2DG_15m	**	0.0084
GDP:PMA_6AN_15m vs. ATP:PMA_6AN_15m	**	0.0045
GDP:PMA_6AN_15m vs. ATP:PMA_DPI_15m	***	0.0001
GDP:PMA_6AN_15m vs. ATP:PMA_AA_15m	ns	0.0698
GDP:PMA_6AN_15m vs. ATP:PMA_IAA_15m	****	<0.0001
GDP:PMA_6AN_15m vs. Xanthine:PMA_15m	**	0.0032
GDP:PMA_6AN_15m vs. Xanthine:PMA_2DG_15m	ns	0.3612
GDP:PMA_6AN_15m vs. Xanthine:PMA_6AN_15m	ns	0.3882
GDP:PMA_6AN_15m vs. Xanthine:PMA_DPI_15m	*	0.0202
GDP:PMA_6AN_15m vs. Xanthine:PMA_AA_15m	ns	0.8245
GDP:PMA_6AN_15m vs. CMP:PMA_15m	ns	0.6303
GDP:PMA_6AN_15m vs. CMP:PMA_2DG_15m	ns	>0.9999
GDP:PMA_6AN_15m vs. CMP:PMA_6AN_15m	ns	>0.9999
GDP:PMA_6AN_15m vs. CMP:PMA_DPI_15m	ns	0.9427
GDP:PMA_6AN_15m vs. CMP:PMA_AA_15m	ns	>0.9999
GDP:PMA_6AN_15m vs. CMP:PMA_IAA_15m	***	0.0005
GDP:PMA_6AN_15m vs. CDP:PMA_15m	ns	0.9623
GDP:PMA_6AN_15m vs. CDP:PMA_2DG_15m	ns	>0.9999
GDP:PMA_6AN_15m vs. CDP:PMA_6AN_15m	ns	>0.9999
GDP:PMA_6AN_15m vs. CDP:PMA_DPI_15m	ns	0.9996
GDP:PMA_6AN_15m vs. CDP:PMA_AA_15m	ns	>0.9999
GDP:PMA_6AN_15m vs. CDP:PMA_IAA_15m	**	0.0057
GDP:PMA_6AN_15m vs. CTP:PMA_15m	***	0.0002
GDP:PMA_6AN_15m vs. CTP:PMA_2DG_15m	ns	0.0814
GDP:PMA_6AN_15m vs. CTP:PMA_6AN_15m	ns	0.0664
GDP:PMA_6AN_15m vs. CTP:PMA_DPI_15m	**	0.0019
GDP:PMA_6AN_15m vs. CTP:PMA_AA_15m	ns	0.3846
GDP:PMA_6AN_15m vs. CTP:PMA_IAA_15m	****	<0.0001

GDP:PMA_6AN_15m vs. Uridine:PMA_15m	ns	0.7909
GDP:PMA_6AN_15m vs. Uridine:PMA_2DG_15m	ns	>0.9999
GDP:PMA_6AN_15m vs. Uridine:PMA_6AN_15m	ns	>0.9999
GDP:PMA_6AN_15m vs. Uridine:PMA_DPI_15m	ns	0.9849
GDP:PMA_6AN_15m vs. Uridine:PMA_AA_15m	ns	>0.9999
GDP:PMA_6AN_15m vs. Uridine:PMA_IAA_15m	**	0.0013
GDP:PMA_6AN_15m vs. UMP:PMA_15m	ns	0.9987
GDP:PMA_6AN_15m vs. UMP:PMA_2DG_15m	ns	>0.9999
GDP:PMA_6AN_15m vs. UMP:PMA_6AN_15m	ns	>0.9999
GDP:PMA_6AN_15m vs. UMP:PMA_DPI_15m	ns	>0.9999
GDP:PMA_6AN_15m vs. UMP:PMA_AA_15m	ns	>0.9999
GDP:PMA_6AN_15m vs. UMP:PMA_IAA_15m	*	0.0248
GDP:PMA_6AN_15m vs. UDP:PMA_15m	ns	0.9984
GDP:PMA_6AN_15m vs. UDP:PMA_2DG_15m	ns	>0.9999
GDP:PMA_6AN_15m vs. UDP:PMA_6AN_15m	ns	>0.9999
GDP:PMA_6AN_15m vs. UDP:PMA_DPI_15m	ns	>0.9999
GDP:PMA_6AN_15m vs. UDP:PMA_AA_15m	ns	>0.9999
GDP:PMA_6AN_15m vs. UDP:PMA_IAA_15m	*	0.0231
GDP:PMA_6AN_15m vs. UTP:PMA_15m	****	<0.0001
GDP:PMA_6AN_15m vs. UTP:PMA_2DG_15m	*	0.0429
GDP:PMA_6AN_15m vs. UTP:PMA_6AN_15m	*	0.0313
GDP:PMA_6AN_15m vs. UTP:PMA_DPI_15m	***	0.0008
GDP:PMA_6AN_15m vs. UTP:PMA_AA_15m	ns	0.2482
GDP:PMA_6AN_15m vs. UTP:PMA_IAA_15m	****	<0.0001
GDP:PMA_DPI_15m vs. GDP:PMA_AA_15m	ns	0.5731
GDP:PMA_DPI_15m vs. GDP:PMA_IAA_15m	***	0.0004
GDP:PMA_DPI_15m vs. GTP:PMA_15m	*	0.0269
GDP:PMA_DPI_15m vs. GTP:PMA_2DG_15m	ns	0.8494
GDP:PMA_DPI_15m vs. GTP:PMA_6AN_15m	ns	0.99
GDP:PMA_DPI_15m vs. GTP:PMA_DPI_15m	*	0.0212
GDP:PMA_DPI_15m vs. GTP:PMA_AA_15m	ns	0.9972
GDP:PMA_DPI_15m vs. GTP:PMA_IAA_15m	****	<0.0001
GDP:PMA_DPI_15m vs. Guanosine:PMA_15m	ns	>0.9999
GDP:PMA_DPI_15m vs. Guanosine:PMA_2DG_15m	ns	>0.9999
GDP:PMA_DPI_15m vs. Guanosine:PMA_6AN_15m	ns	>0.9999
GDP:PMA_DPI_15m vs. Guanosine:PMA_DPI_15m	ns	>0.9999
GDP:PMA_DPI_15m vs. Guanosine:PMA_AA_15m	ns	>0.9999
GDP:PMA_DPI_15m vs. Guanosine:PMA_IAA_15m	ns	0.2787
GDP:PMA_DPI_15m vs. Guanine:PMA_15m	ns	0.3591
GDP:PMA_DPI_15m vs. Guanine:PMA_2DG_15m	ns	>0.9999
GDP:PMA_DPI_15m vs. Guanine:PMA_6AN_15m	ns	>0.9999
GDP:PMA_DPI_15m vs. Guanine:PMA_DPI_15m	ns	0.4046
GDP:PMA_DPI_15m vs. Guanine:PMA_AA_15m	ns	>0.9999
GDP:PMA_DPI_15m vs. Guanine:PMA_IAA_15m	***	0.0001
GDP:PMA_DPI_15m vs. Aspartic acid:PMA_15m	ns	0.5888
GDP:PMA_DPI_15m vs. Aspartic acid:PMA_2DG_15m	ns	>0.9999
GDP:PMA_DPI_15m vs. Aspartic acid:PMA_6AN_15m	ns	>0.9999

GDP:PMA_DPI_15m vs. Aspartic acid:PMA_DPI_15m	ns	0.6721
GDP:PMA_DPI_15m vs. Aspartic acid:PMA_AA_15m	ns	>0.9999
GDP:PMA_DPI_15m vs. Aspartic acid:PMA_IAA_15m	***	0.0004
GDP:PMA_DPI_15m vs. AMP:PMA_15m	ns	>0.9999
GDP:PMA_DPI_15m vs. AMP:PMA_2DG_15m	ns	>0.9999
GDP:PMA_DPI_15m vs. AMP:PMA_6AN_15m	ns	>0.9999
GDP:PMA_DPI_15m vs. AMP:PMA_DPI_15m	ns	>0.9999
GDP:PMA_DPI_15m vs. AMP:PMA_AA_15m	ns	>0.9999
GDP:PMA_DPI_15m vs. AMP:PMA_IAA_15m	ns	0.0889
GDP:PMA_DPI_15m vs. ADP:PMA_15m	ns	0.9991
GDP:PMA_DPI_15m vs. ADP:PMA_2DG_15m	ns	>0.9999
GDP:PMA_DPI_15m vs. ADP:PMA_6AN_15m	ns	>0.9999
GDP:PMA_DPI_15m vs. ADP:PMA_DPI_15m	ns	>0.9999
GDP:PMA_DPI_15m vs. ADP:PMA_AA_15m	ns	>0.9999
GDP:PMA_DPI_15m vs. ADP:PMA_IAA_15m	*	0.0274
GDP:PMA_DPI_15m vs. ATP:PMA_15m	**	0.0072
GDP:PMA_DPI_15m vs. ATP:PMA_2DG_15m	ns	0.578
GDP:PMA_DPI_15m vs. ATP:PMA_6AN_15m	ns	0.9045
GDP:PMA_DPI_15m vs. ATP:PMA_DPI_15m	**	0.0045
GDP:PMA_DPI_15m vs. ATP:PMA_AA_15m	ns	0.9545
GDP:PMA_DPI_15m vs. ATP:PMA_IAA_15m	****	<0.0001
GDP:PMA_DPI_15m vs. Xanthine:PMA_15m	ns	0.3337
GDP:PMA_DPI_15m vs. Xanthine:PMA_2DG_15m	ns	0.9998
GDP:PMA_DPI_15m vs. Xanthine:PMA_6AN_15m	ns	>0.9999
GDP:PMA_DPI_15m vs. Xanthine:PMA_DPI_15m	ns	0.3882
GDP:PMA_DPI_15m vs. Xanthine:PMA_AA_15m	ns	>0.9999
GDP:PMA_DPI_15m vs. CMP:PMA_15m	ns	>0.9999
GDP:PMA_DPI_15m vs. CMP:PMA_2DG_15m	ns	>0.9999
GDP:PMA_DPI_15m vs. CMP:PMA_6AN_15m	ns	>0.9999
GDP:PMA_DPI_15m vs. CMP:PMA_DPI_15m	ns	>0.9999
GDP:PMA_DPI_15m vs. CMP:PMA_AA_15m	ns	>0.9999
GDP:PMA_DPI_15m vs. CMP:PMA_IAA_15m	ns	0.1305
GDP:PMA_DPI_15m vs. CDP:PMA_15m	ns	>0.9999
GDP:PMA_DPI_15m vs. CDP:PMA_2DG_15m	ns	>0.9999
GDP:PMA_DPI_15m vs. CDP:PMA_6AN_15m	ns	>0.9999
GDP:PMA_DPI_15m vs. CDP:PMA_DPI_15m	ns	>0.9999
GDP:PMA_DPI_15m vs. CDP:PMA_AA_15m	ns	>0.9999
GDP:PMA_DPI_15m vs. CDP:PMA_IAA_15m	ns	0.4876
GDP:PMA_DPI_15m vs. CTP:PMA_15m	ns	0.0721
GDP:PMA_DPI_15m vs. CTP:PMA_2DG_15m	ns	0.9664
GDP:PMA_DPI_15m vs. CTP:PMA_6AN_15m	ns	0.9995
GDP:PMA_DPI_15m vs. CTP:PMA_DPI_15m	ns	0.0664
GDP:PMA_DPI_15m vs. CTP:PMA_AA_15m	ns	>0.9999
GDP:PMA_DPI_15m vs. CTP:PMA_IAA_15m	****	<0.0001
GDP:PMA_DPI_15m vs. Uridine:PMA_15m	ns	>0.9999
GDP:PMA_DPI_15m vs. Uridine:PMA_2DG_15m	ns	>0.9999
GDP:PMA_DPI_15m vs. Uridine:PMA_6AN_15m	ns	>0.9999

GDP:PMA_DPI_15m vs. Uridine:PMA_DPI_15m	ns	>0.9999
GDP:PMA_DPI_15m vs. Uridine:PMA_AA_15m	ns	>0.9999
GDP:PMA_DPI_15m vs. Uridine:PMA_IAA_15m	ns	0.2244
GDP:PMA_DPI_15m vs. UMP:PMA_15m	ns	>0.9999
GDP:PMA_DPI_15m vs. UMP:PMA_2DG_15m	ns	>0.9999
GDP:PMA_DPI_15m vs. UMP:PMA_6AN_15m	ns	>0.9999
GDP:PMA_DPI_15m vs. UMP:PMA_DPI_15m	ns	>0.9999
GDP:PMA_DPI_15m vs. UMP:PMA_AA_15m	ns	>0.9999
GDP:PMA_DPI_15m vs. UMP:PMA_IAA_15m	ns	0.8066
GDP:PMA_DPI_15m vs. UDP:PMA_15m	ns	>0.9999
GDP:PMA_DPI_15m vs. UDP:PMA_2DG_15m	ns	>0.9999
GDP:PMA_DPI_15m vs. UDP:PMA_6AN_15m	ns	>0.9999
GDP:PMA_DPI_15m vs. UDP:PMA_DPI_15m	ns	>0.9999
GDP:PMA_DPI_15m vs. UDP:PMA_AA_15m	ns	>0.9999
GDP:PMA_DPI_15m vs. UDP:PMA_IAA_15m	ns	0.793
GDP:PMA_DPI_15m vs. UTP:PMA_15m	*	0.0376
GDP:PMA_DPI_15m vs. UTP:PMA_2DG_15m	ns	0.9005
GDP:PMA_DPI_15m vs. UTP:PMA_6AN_15m	ns	0.9958
GDP:PMA_DPI_15m vs. UTP:PMA_DPI_15m	*	0.0313
GDP:PMA_DPI_15m vs. UTP:PMA_AA_15m	ns	0.9991
GDP:PMA_DPI_15m vs. UTP:PMA_IAA_15m	****	<0.0001
GDP:PMA_AA_15m vs. GDP:PMA_IAA_15m	****	<0.0001
GDP:PMA_AA_15m vs. GTP:PMA_15m	****	<0.0001
GDP:PMA_AA_15m vs. GTP:PMA_2DG_15m	*	0.0187
GDP:PMA_AA_15m vs. GTP:PMA_6AN_15m	ns	0.088
GDP:PMA_AA_15m vs. GTP:PMA_DPI_15m	***	0.0003
GDP:PMA_AA_15m vs. GTP:PMA_AA_15m	*	0.0212
GDP:PMA_AA_15m vs. GTP:PMA_IAA_15m	****	<0.0001
GDP:PMA_AA_15m vs. Guanosine:PMA_15m	ns	0.7547
GDP:PMA_AA_15m vs. Guanosine:PMA_2DG_15m	ns	>0.9999
GDP:PMA_AA_15m vs. Guanosine:PMA_6AN_15m	ns	>0.9999
GDP:PMA_AA_15m vs. Guanosine:PMA_DPI_15m	ns	0.9782
GDP:PMA_AA_15m vs. Guanosine:PMA_AA_15m	ns	>0.9999
GDP:PMA_AA_15m vs. Guanosine:PMA_IAA_15m	**	0.001
GDP:PMA_AA_15m vs. Guanine:PMA_15m	**	0.0016
GDP:PMA_AA_15m vs. Guanine:PMA_2DG_15m	ns	0.2877
GDP:PMA_AA_15m vs. Guanine:PMA_6AN_15m	ns	0.6573
GDP:PMA_AA_15m vs. Guanine:PMA_DPI_15m	*	0.0119
GDP:PMA_AA_15m vs. Guanine:PMA_AA_15m	ns	0.4046
GDP:PMA_AA_15m vs. Guanine:PMA_IAA_15m	****	<0.0001
GDP:PMA_AA_15m vs. Aspartic acid:PMA_15m	**	0.0051
GDP:PMA_AA_15m vs. Aspartic acid:PMA_2DG_15m	ns	0.5018
GDP:PMA_AA_15m vs. Aspartic acid:PMA_6AN_15m	ns	0.8592
GDP:PMA_AA_15m vs. Aspartic acid:PMA_DPI_15m	*	0.0327
GDP:PMA_AA_15m vs. Aspartic acid:PMA_AA_15m	ns	0.6721
GDP:PMA_AA_15m vs. Aspartic acid:PMA_IAA_15m	****	<0.0001
GDP:PMA_AA_15m vs. AMP:PMA_15m	ns	0.4021

GDP:PMA_AA_15m vs. AMP:PMA_2DG_15m	ns	>0.9999
GDP:PMA_AA_15m vs. AMP:PMA_6AN_15m	ns	>0.9999
GDP:PMA_AA_15m vs. AMP:PMA_DPI_15m	ns	0.8079
GDP:PMA_AA_15m vs. AMP:PMA_AA_15m	ns	>0.9999
GDP:PMA_AA_15m vs. AMP:PMA_IAA_15m	***	0.0002
GDP:PMA_AA_15m vs. ADP:PMA_15m	ns	0.1746
GDP:PMA_AA_15m vs. ADP:PMA_2DG_15m	ns	0.9972
GDP:PMA_AA_15m vs. ADP:PMA_6AN_15m	ns	>0.9999
GDP:PMA_AA_15m vs. ADP:PMA_DPI_15m	ns	0.5185
GDP:PMA_AA_15m vs. ADP:PMA_AA_15m	ns	>0.9999
GDP:PMA_AA_15m vs. ADP:PMA_IAA_15m	****	<0.0001
GDP:PMA_AA_15m vs. ATP:PMA_15m	****	<0.0001
GDP:PMA_AA_15m vs. ATP:PMA_2DG_15m	**	0.0048
GDP:PMA_AA_15m vs. ATP:PMA_6AN_15m	*	0.0273
GDP:PMA_AA_15m vs. ATP:PMA_DPI_15m	****	<0.0001
GDP:PMA_AA_15m vs. ATP:PMA_AA_15m	**	0.0045
GDP:PMA_AA_15m vs. ATP:PMA_IAA_15m	****	<0.0001
GDP:PMA_AA_15m vs. Xanthine:PMA_15m	**	0.0018
GDP:PMA_AA_15m vs. Xanthine:PMA_2DG_15m	ns	0.2679
GDP:PMA_AA_15m vs. Xanthine:PMA_6AN_15m	ns	0.6164
GDP:PMA_AA_15m vs. Xanthine:PMA_DPI_15m	*	0.0123
GDP:PMA_AA_15m vs. Xanthine:PMA_AA_15m	ns	0.3882
GDP:PMA_AA_15m vs. CMP:PMA_15m	ns	0.51
GDP:PMA_AA_15m vs. CMP:PMA_2DG_15m	ns	>0.9999
GDP:PMA_AA_15m vs. CMP:PMA_6AN_15m	ns	>0.9999
GDP:PMA_AA_15m vs. CMP:PMA_DPI_15m	ns	0.8852
GDP:PMA_AA_15m vs. CMP:PMA_AA_15m	ns	>0.9999
GDP:PMA_AA_15m vs. CMP:PMA_IAA_15m	***	0.0003
GDP:PMA_AA_15m vs. CDP:PMA_15m	ns	0.9177
GDP:PMA_AA_15m vs. CDP:PMA_2DG_15m	ns	>0.9999
GDP:PMA_AA_15m vs. CDP:PMA_6AN_15m	ns	>0.9999
GDP:PMA_AA_15m vs. CDP:PMA_DPI_15m	ns	0.9981
GDP:PMA_AA_15m vs. CDP:PMA_AA_15m	ns	>0.9999
GDP:PMA_AA_15m vs. CDP:PMA_IAA_15m	**	0.0032
GDP:PMA_AA_15m vs. CTP:PMA_15m	***	0.0001
GDP:PMA_AA_15m vs. CTP:PMA_2DG_15m	ns	0.052
GDP:PMA_AA_15m vs. CTP:PMA_6AN_15m	ns	0.202
GDP:PMA_AA_15m vs. CTP:PMA_DPI_15m	**	0.001
GDP:PMA_AA_15m vs. CTP:PMA_AA_15m	ns	0.0664
GDP:PMA_AA_15m vs. CTP:PMA_IAA_15m	****	<0.0001
GDP:PMA_AA_15m vs. Uridine:PMA_15m	ns	0.6832
GDP:PMA_AA_15m vs. Uridine:PMA_2DG_15m	ns	>0.9999
GDP:PMA_AA_15m vs. Uridine:PMA_6AN_15m	ns	>0.9999
GDP:PMA_AA_15m vs. Uridine:PMA_DPI_15m	ns	0.9604
GDP:PMA_AA_15m vs. Uridine:PMA_AA_15m	ns	>0.9999
GDP:PMA_AA_15m vs. Uridine:PMA_IAA_15m	***	0.0007
GDP:PMA_AA_15m vs. UMP:PMA_15m	ns	0.9946

GDP:PMA_AA_15m vs. UMP:PMA_2DG_15m	ns	>0.9999
GDP:PMA_AA_15m vs. UMP:PMA_6AN_15m	ns	>0.9999
GDP:PMA_AA_15m vs. UMP:PMA_DPI_15m	ns	>0.9999
GDP:PMA_AA_15m vs. UMP:PMA_AA_15m	ns	>0.9999
GDP:PMA_AA_15m vs. UMP:PMA_IAA_15m	*	0.0149
GDP:PMA_AA_15m vs. UDP:PMA_15m	ns	0.9935
GDP:PMA_AA_15m vs. UDP:PMA_2DG_15m	ns	>0.9999
GDP:PMA_AA_15m vs. UDP:PMA_6AN_15m	ns	>0.9999
GDP:PMA_AA_15m vs. UDP:PMA_DPI_15m	ns	>0.9999
GDP:PMA_AA_15m vs. UDP:PMA_AA_15m	ns	>0.9999
GDP:PMA_AA_15m vs. UDP:PMA_IAA_15m	*	0.0139
GDP:PMA_AA_15m vs. UTP:PMA_15m	****	<0.0001
GDP:PMA_AA_15m vs. UTP:PMA_2DG_15m	*	0.0265
GDP:PMA_AA_15m vs. UTP:PMA_6AN_15m	ns	0.1175
GDP:PMA_AA_15m vs. UTP:PMA_DPI_15m	***	0.0004
GDP:PMA_AA_15m vs. UTP:PMA_AA_15m	*	0.0313
GDP:PMA_AA_15m vs. UTP:PMA_IAA_15m	****	<0.0001
GDP:PMA_IAA_15m vs. GTP:PMA_15m	ns	>0.9999
GDP:PMA_IAA_15m vs. GTP:PMA_2DG_15m	ns	>0.9999
GDP:PMA_IAA_15m vs. GTP:PMA_6AN_15m	ns	>0.9999
GDP:PMA_IAA_15m vs. GTP:PMA_DPI_15m	ns	>0.9999
GDP:PMA_IAA_15m vs. GTP:PMA_AA_15m	ns	>0.9999
GDP:PMA_IAA_15m vs. GTP:PMA_IAA_15m	*	0.0212
GDP:PMA_IAA_15m vs. Guanosine:PMA_15m	ns	>0.9999
GDP:PMA_IAA_15m vs. Guanosine:PMA_2DG_15m	ns	0.9694
GDP:PMA_IAA_15m vs. Guanosine:PMA_6AN_15m	ns	0.7476
GDP:PMA_IAA_15m vs. Guanosine:PMA_DPI_15m	ns	>0.9999
GDP:PMA_IAA_15m vs. Guanosine:PMA_AA_15m	ns	0.6342
GDP:PMA_IAA_15m vs. Guanosine:PMA_IAA_15m	ns	>0.9999
GDP:PMA_IAA_15m vs. Guanine:PMA_15m	ns	>0.9999
GDP:PMA_IAA_15m vs. Guanine:PMA_2DG_15m	ns	>0.9999
GDP:PMA_IAA_15m vs. Guanine:PMA_6AN_15m	ns	>0.9999
GDP:PMA_IAA_15m vs. Guanine:PMA_DPI_15m	ns	>0.9999
GDP:PMA_IAA_15m vs. Guanine:PMA_AA_15m	ns	>0.9999
GDP:PMA_IAA_15m vs. Guanine:PMA_IAA_15m	ns	0.4046
GDP:PMA_IAA_15m vs. Aspartic acid:PMA_15m	ns	>0.9999
GDP:PMA_IAA_15m vs. Aspartic acid:PMA_2DG_15m	ns	>0.9999
GDP:PMA_IAA_15m vs. Aspartic acid:PMA_6AN_15m	ns	>0.9999
GDP:PMA_IAA_15m vs. Aspartic acid:PMA_DPI_15m	ns	>0.9999
GDP:PMA_IAA_15m vs. Aspartic acid:PMA_AA_15m	ns	>0.9999
GDP:PMA_IAA_15m vs. Aspartic acid:PMA_IAA_15m	ns	0.6721
GDP:PMA_IAA_15m vs. AMP:PMA_15m	ns	>0.9999
GDP:PMA_IAA_15m vs. AMP:PMA_2DG_15m	ns	0.9994
GDP:PMA_IAA_15m vs. AMP:PMA_6AN_15m	ns	0.9616
GDP:PMA_IAA_15m vs. AMP:PMA_DPI_15m	ns	>0.9999
GDP:PMA_IAA_15m vs. AMP:PMA_AA_15m	ns	0.9167
GDP:PMA_IAA_15m vs. AMP:PMA_IAA_15m	ns	>0.9999

GDP:PMA_IAA_15m vs. ADP:PMA_15m	ns	>0.9999
GDP:PMA_IAA_15m vs. ADP:PMA_2DG_15m	ns	>0.9999
GDP:PMA_IAA_15m vs. ADP:PMA_6AN_15m	ns	0.998
GDP:PMA_IAA_15m vs. ADP:PMA_DPI_15m	ns	>0.9999
GDP:PMA_IAA_15m vs. ADP:PMA_AA_15m	ns	0.9923
GDP:PMA_IAA_15m vs. ADP:PMA_IAA_15m	ns	>0.9999
GDP:PMA_IAA_15m vs. ATP:PMA_15m	ns	0.9999
GDP:PMA_IAA_15m vs. ATP:PMA_2DG_15m	ns	>0.9999
GDP:PMA_IAA_15m vs. ATP:PMA_6AN_15m	ns	>0.9999
GDP:PMA_IAA_15m vs. ATP:PMA_DPI_15m	ns	>0.9999
GDP:PMA_IAA_15m vs. ATP:PMA_AA_15m	ns	>0.9999
GDP:PMA_IAA_15m vs. ATP:PMA_IAA_15m	**	0.0045
GDP:PMA_IAA_15m vs. Xanthine:PMA_15m	ns	>0.9999
GDP:PMA_IAA_15m vs. Xanthine:PMA_2DG_15m	ns	>0.9999
GDP:PMA_IAA_15m vs. Xanthine:PMA_6AN_15m	ns	>0.9999
GDP:PMA_IAA_15m vs. Xanthine:PMA_DPI_15m	ns	>0.9999
GDP:PMA_IAA_15m vs. Xanthine:PMA_AA_15m	ns	>0.9999
GDP:PMA_IAA_15m vs. CMP:PMA_15m	ns	>0.9999
GDP:PMA_IAA_15m vs. CMP:PMA_2DG_15m	ns	0.9973
GDP:PMA_IAA_15m vs. CMP:PMA_6AN_15m	ns	0.9205
GDP:PMA_IAA_15m vs. CMP:PMA_DPI_15m	ns	>0.9999
GDP:PMA_IAA_15m vs. CMP:PMA_AA_15m	ns	0.8516
GDP:PMA_IAA_15m vs. CMP:PMA_IAA_15m	ns	>0.9999
GDP:PMA_IAA_15m vs. CDP:PMA_15m	ns	>0.9999
GDP:PMA_IAA_15m vs. CDP:PMA_2DG_15m	ns	0.8686
GDP:PMA_IAA_15m vs. CDP:PMA_6AN_15m	ns	0.5177
GDP:PMA_IAA_15m vs. CDP:PMA_DPI_15m	ns	>0.9999
GDP:PMA_IAA_15m vs. CDP:PMA_AA_15m	ns	0.4021
GDP:PMA_IAA_15m vs. CDP:PMA_IAA_15m	ns	>0.9999
GDP:PMA_IAA_15m vs. CTP:PMA_15m	ns	>0.9999
GDP:PMA_IAA_15m vs. CTP:PMA_2DG_15m	ns	>0.9999
GDP:PMA_IAA_15m vs. CTP:PMA_6AN_15m	ns	>0.9999
GDP:PMA_IAA_15m vs. CTP:PMA_DPI_15m	ns	>0.9999
GDP:PMA_IAA_15m vs. CTP:PMA_AA_15m	ns	>0.9999
GDP:PMA_IAA_15m vs. CTP:PMA_IAA_15m	ns	0.0664
GDP:PMA_IAA_15m vs. Uridine:PMA_15m	ns	>0.9999
GDP:PMA_IAA_15m vs. Uridine:PMA_2DG_15m	ns	0.9838
GDP:PMA_IAA_15m vs. Uridine:PMA_6AN_15m	ns	0.8121
GDP:PMA_IAA_15m vs. Uridine:PMA_DPI_15m	ns	>0.9999
GDP:PMA_IAA_15m vs. Uridine:PMA_AA_15m	ns	0.7089
GDP:PMA_IAA_15m vs. Uridine:PMA_IAA_15m	ns	>0.9999
GDP:PMA_IAA_15m vs. UMP:PMA_15m	ns	>0.9999
GDP:PMA_IAA_15m vs. UMP:PMA_2DG_15m	ns	0.5742
GDP:PMA_IAA_15m vs. UMP:PMA_6AN_15m	ns	0.229
GDP:PMA_IAA_15m vs. UMP:PMA_DPI_15m	ns	0.9985
GDP:PMA_IAA_15m vs. UMP:PMA_AA_15m	ns	0.1592
GDP:PMA_IAA_15m vs. UMP:PMA_IAA_15m	ns	>0.9999

GDP:PMA_IAA_15m vs. UDP:PMA_15m	ns	>0.9999
GDP:PMA_IAA_15m vs. UDP:PMA_2DG_15m	ns	0.5913
GDP:PMA_IAA_15m vs. UDP:PMA_6AN_15m	ns	0.2405
GDP:PMA_IAA_15m vs. UDP:PMA_DPI_15m	ns	0.9988
GDP:PMA_IAA_15m vs. UDP:PMA_AA_15m	ns	0.168
GDP:PMA_IAA_15m vs. UDP:PMA_IAA_15m	ns	>0.9999
GDP:PMA_IAA_15m vs. UTP:PMA_15m	ns	>0.9999
GDP:PMA_IAA_15m vs. UTP:PMA_2DG_15m	ns	>0.9999
GDP:PMA_IAA_15m vs. UTP:PMA_6AN_15m	ns	>0.9999
GDP:PMA_IAA_15m vs. UTP:PMA_DPI_15m	ns	>0.9999
GDP:PMA_IAA_15m vs. UTP:PMA_AA_15m	ns	>0.9999
GDP:PMA_IAA_15m vs. UTP:PMA_IAA_15m	*	0.0313
GTP:PMA_15m vs. GTP:PMA_2DG_15m	ns	0.7475
GTP:PMA_15m vs. GTP:PMA_6AN_15m	ns	0.1037
GTP:PMA_15m vs. GTP:PMA_DPI_15m	ns	>0.9999
GTP:PMA_15m vs. GTP:PMA_AA_15m	*	0.0409
GTP:PMA_15m vs. GTP:PMA_IAA_15m	*	0.0236
GTP:PMA_15m vs. Guanosine:PMA_15m	ns	0.5324
GTP:PMA_15m vs. Guanosine:PMA_2DG_15m	*	0.0282
GTP:PMA_15m vs. Guanosine:PMA_6AN_15m	**	0.005
GTP:PMA_15m vs. Guanosine:PMA_DPI_15m	ns	0.4662
GTP:PMA_15m vs. Guanosine:PMA_AA_15m	**	0.0029
GTP:PMA_15m vs. Guanosine:PMA_IAA_15m	ns	>0.9999
GTP:PMA_15m vs. Guanine:PMA_15m	ns	>0.9999
GTP:PMA_15m vs. Guanine:PMA_2DG_15m	ns	0.9971
GTP:PMA_15m vs. Guanine:PMA_6AN_15m	ns	0.9172
GTP:PMA_15m vs. Guanine:PMA_DPI_15m	ns	>0.9999
GTP:PMA_15m vs. Guanine:PMA_AA_15m	ns	0.8464
GTP:PMA_15m vs. Guanine:PMA_IAA_15m	ns	>0.9999
GTP:PMA_15m vs. Aspartic acid:PMA_15m	ns	>0.9999
GTP:PMA_15m vs. Aspartic acid:PMA_2DG_15m	ns	0.9709
GTP:PMA_15m vs. Aspartic acid:PMA_6AN_15m	ns	0.7522
GTP:PMA_15m vs. Aspartic acid:PMA_DPI_15m	ns	>0.9999
GTP:PMA_15m vs. Aspartic acid:PMA_AA_15m	ns	0.6389
GTP:PMA_15m vs. Aspartic acid:PMA_IAA_15m	ns	>0.9999
GTP:PMA_15m vs. AMP:PMA_15m	ns	0.8908
GTP:PMA_15m vs. AMP:PMA_2DG_15m	ns	0.1115
GTP:PMA_15m vs. AMP:PMA_6AN_15m	*	0.0249
GTP:PMA_15m vs. AMP:PMA_DPI_15m	ns	0.8109
GTP:PMA_15m vs. AMP:PMA_AA_15m	*	0.0149
GTP:PMA_15m vs. AMP:PMA_IAA_15m	ns	>0.9999
GTP:PMA_15m vs. ADP:PMA_15m	ns	0.9919
GTP:PMA_15m vs. ADP:PMA_2DG_15m	ns	0.2859
GTP:PMA_15m vs. ADP:PMA_6AN_15m	ns	0.082
GTP:PMA_15m vs. ADP:PMA_DPI_15m	ns	0.9669
GTP:PMA_15m vs. ADP:PMA_AA_15m	ns	0.0525
GTP:PMA_15m vs. ADP:PMA_IAA_15m	ns	>0.9999

GTP:PMA_15m vs. ATP:PMA_15m	ns	>0.9999
GTP:PMA_15m vs. ATP:PMA_2DG_15m	ns	>0.9999
GTP:PMA_15m vs. ATP:PMA_6AN_15m	ns	>0.9999
GTP:PMA_15m vs. ATP:PMA_DPI_15m	ns	>0.9999
GTP:PMA_15m vs. ATP:PMA_AA_15m	ns	>0.9999
GTP:PMA_15m vs. ATP:PMA_IAA_15m	ns	0.9881
GTP:PMA_15m vs. Xanthine:PMA_15m	ns	>0.9999
GTP:PMA_15m vs. Xanthine:PMA_2DG_15m	ns	0.9999
GTP:PMA_15m vs. Xanthine:PMA_6AN_15m	ns	0.986
GTP:PMA_15m vs. Xanthine:PMA_DPI_15m	ns	>0.9999
GTP:PMA_15m vs. Xanthine:PMA_AA_15m	ns	0.9639
GTP:PMA_15m vs. CMP:PMA_15m	ns	0.8031
GTP:PMA_15m vs. CMP:PMA_2DG_15m	ns	0.075
GTP:PMA_15m vs. CMP:PMA_6AN_15m	*	0.0155
GTP:PMA_15m vs. CMP:PMA_DPI_15m	ns	0.7145
GTP:PMA_15m vs. CMP:PMA_AA_15m	**	0.0091
GTP:PMA_15m vs. CMP:PMA_IAA_15m	ns	>0.9999
GTP:PMA_15m vs. CDP:PMA_15m	ns	0.2871
GTP:PMA_15m vs. CDP:PMA_2DG_15m	*	0.0102
GTP:PMA_15m vs. CDP:PMA_6AN_15m	**	0.0016
GTP:PMA_15m vs. CDP:PMA_DPI_15m	ns	0.2619
GTP:PMA_15m vs. CDP:PMA_AA_15m	***	0.0009
GTP:PMA_15m vs. CDP:PMA_IAA_15m	ns	>0.9999
GTP:PMA_15m vs. CTP:PMA_15m	ns	>0.9999
GTP:PMA_15m vs. CTP:PMA_2DG_15m	ns	>0.9999
GTP:PMA_15m vs. CTP:PMA_6AN_15m	ns	0.9994
GTP:PMA_15m vs. CTP:PMA_DPI_15m	ns	>0.9999
GTP:PMA_15m vs. CTP:PMA_AA_15m	ns	0.9973
GTP:PMA_15m vs. CTP:PMA_IAA_15m	ns	>0.9999
GTP:PMA_15m vs. Uridine:PMA_15m	ns	0.6211
GTP:PMA_15m vs. Uridine:PMA_2DG_15m	*	0.0385
GTP:PMA_15m vs. Uridine:PMA_6AN_15m	**	0.0072
GTP:PMA_15m vs. Uridine:PMA_DPI_15m	ns	0.5431
GTP:PMA_15m vs. Uridine:PMA_AA_15m	**	0.0041
GTP:PMA_15m vs. Uridine:PMA_IAA_15m	ns	>0.9999
GTP:PMA_15m vs. UMP:PMA_15m	ns	0.0853
GTP:PMA_15m vs. UMP:PMA_2DG_15m	**	0.0021
GTP:PMA_15m vs. UMP:PMA_6AN_15m	***	0.0003
GTP:PMA_15m vs. UMP:PMA_DPI_15m	ns	0.0895
GTP:PMA_15m vs. UMP:PMA_AA_15m	***	0.0002
GTP:PMA_15m vs. UMP:PMA_IAA_15m	ns	>0.9999
GTP:PMA_15m vs. UDP:PMA_15m	ns	0.0914
GTP:PMA_15m vs. UDP:PMA_2DG_15m	**	0.0023
GTP:PMA_15m vs. UDP:PMA_6AN_15m	***	0.0003
GTP:PMA_15m vs. UDP:PMA_DPI_15m	ns	0.0951
GTP:PMA_15m vs. UDP:PMA_AA_15m	***	0.0002
GTP:PMA_15m vs. UDP:PMA_IAA_15m	ns	>0.9999

GTP:PMA_15m vs. UTP:PMA_15m	ns	>0.9999
GTP:PMA_15m vs. UTP:PMA_2DG_15m	ns	>0.9999
GTP:PMA_15m vs. UTP:PMA_6AN_15m	ns	>0.9999
GTP:PMA_15m vs. UTP:PMA_DPI_15m	ns	>0.9999
GTP:PMA_15m vs. UTP:PMA_AA_15m	ns	0.9997
GTP:PMA_15m vs. UTP:PMA_IAA_15m	ns	>0.9999
GTP:PMA_2DG_15m vs. GTP:PMA_6AN_15m	ns	>0.9999
GTP:PMA_2DG_15m vs. GTP:PMA_DPI_15m	ns	>0.9999
GTP:PMA_2DG_15m vs. GTP:PMA_AA_15m	ns	>0.9999
GTP:PMA_2DG_15m vs. GTP:PMA_IAA_15m	****	<0.0001
GTP:PMA_2DG_15m vs. Guanosine:PMA_15m	ns	>0.9999
GTP:PMA_2DG_15m vs. Guanosine:PMA_2DG_15m	ns	0.5324
GTP:PMA_2DG_15m vs. Guanosine:PMA_6AN_15m	ns	0.499
GTP:PMA_2DG_15m vs. Guanosine:PMA_DPI_15m	ns	>0.9999
GTP:PMA_2DG_15m vs. Guanosine:PMA_AA_15m	ns	0.3846
GTP:PMA_2DG_15m vs. Guanosine:PMA_IAA_15m	ns	>0.9999
GTP:PMA_2DG_15m vs. Guanine:PMA_15m	ns	>0.9999
GTP:PMA_2DG_15m vs. Guanine:PMA_2DG_15m	ns	>0.9999
GTP:PMA_2DG_15m vs. Guanine:PMA_6AN_15m	ns	>0.9999
GTP:PMA_2DG_15m vs. Guanine:PMA_DPI_15m	ns	>0.9999
GTP:PMA_2DG_15m vs. Guanine:PMA_AA_15m	ns	>0.9999
GTP:PMA_2DG_15m vs. Guanine:PMA_IAA_15m	ns	0.9323
GTP:PMA_2DG_15m vs. Aspartic acid:PMA_15m	ns	>0.9999
GTP:PMA_2DG_15m vs. Aspartic acid:PMA_2DG_15m	ns	>0.9999
GTP:PMA_2DG_15m vs. Aspartic acid:PMA_6AN_15m	ns	>0.9999
GTP:PMA_2DG_15m vs. Aspartic acid:PMA_DPI_15m	ns	>0.9999
GTP:PMA_2DG_15m vs. Aspartic acid:PMA_AA_15m	ns	>0.9999
GTP:PMA_2DG_15m vs. Aspartic acid:PMA_IAA_15m	ns	0.9893
GTP:PMA_2DG_15m vs. AMP:PMA_15m	ns	>0.9999
GTP:PMA_2DG_15m vs. AMP:PMA_2DG_15m	ns	0.8908
GTP:PMA_2DG_15m vs. AMP:PMA_6AN_15m	ns	0.836
GTP:PMA_2DG_15m vs. AMP:PMA_DPI_15m	ns	>0.9999
GTP:PMA_2DG_15m vs. AMP:PMA_AA_15m	ns	0.7376
GTP:PMA_2DG_15m vs. AMP:PMA_IAA_15m	ns	>0.9999
GTP:PMA_2DG_15m vs. ADP:PMA_15m	ns	>0.9999
GTP:PMA_2DG_15m vs. ADP:PMA_2DG_15m	ns	0.9919
GTP:PMA_2DG_15m vs. ADP:PMA_6AN_15m	ns	0.9744
GTP:PMA_2DG_15m vs. ADP:PMA_DPI_15m	ns	>0.9999
GTP:PMA_2DG_15m vs. ADP:PMA_AA_15m	ns	0.9397
GTP:PMA_2DG_15m vs. ADP:PMA_IAA_15m	ns	>0.9999
GTP:PMA_2DG_15m vs. ATP:PMA_15m	ns	>0.9999
GTP:PMA_2DG_15m vs. ATP:PMA_2DG_15m	ns	>0.9999
GTP:PMA_2DG_15m vs. ATP:PMA_6AN_15m	ns	>0.9999
GTP:PMA_2DG_15m vs. ATP:PMA_DPI_15m	ns	>0.9999
GTP:PMA_2DG_15m vs. ATP:PMA_AA_15m	ns	>0.9999
GTP:PMA_2DG_15m vs. ATP:PMA_IAA_15m	ns	0.1139
GTP:PMA_2DG_15m vs. Xanthine:PMA_15m	ns	>0.9999

GTP:PMA_2DG_15m vs. Xanthine:PMA_2DG_15m	ns	>0.9999
GTP:PMA_2DG_15m vs. Xanthine:PMA_6AN_15m	ns	>0.9999
GTP:PMA_2DG_15m vs. Xanthine:PMA_DPI_15m	ns	>0.9999
GTP:PMA_2DG_15m vs. Xanthine:PMA_AA_15m	ns	>0.9999
GTP:PMA_2DG_15m vs. CMP:PMA_15m	ns	>0.9999
GTP:PMA_2DG_15m vs. CMP:PMA_2DG_15m	ns	0.8031
GTP:PMA_2DG_15m vs. CMP:PMA_6AN_15m	ns	0.7449
GTP:PMA_2DG_15m vs. CMP:PMA_DPI_15m	ns	>0.9999
GTP:PMA_2DG_15m vs. CMP:PMA_AA_15m	ns	0.6307
GTP:PMA_2DG_15m vs. CMP:PMA_IAA_15m	ns	>0.9999
GTP:PMA_2DG_15m vs. CDP:PMA_15m	ns	>0.9999
GTP:PMA_2DG_15m vs. CDP:PMA_2DG_15m	ns	0.2871
GTP:PMA_2DG_15m vs. CDP:PMA_6AN_15m	ns	0.2868
GTP:PMA_2DG_15m vs. CDP:PMA_DPI_15m	ns	0.9996
GTP:PMA_2DG_15m vs. CDP:PMA_AA_15m	ns	0.204
GTP:PMA_2DG_15m vs. CDP:PMA_IAA_15m	ns	>0.9999
GTP:PMA_2DG_15m vs. CTP:PMA_15m	ns	>0.9999
GTP:PMA_2DG_15m vs. CTP:PMA_2DG_15m	ns	>0.9999
GTP:PMA_2DG_15m vs. CTP:PMA_6AN_15m	ns	>0.9999
GTP:PMA_2DG_15m vs. CTP:PMA_DPI_15m	ns	>0.9999
GTP:PMA_2DG_15m vs. CTP:PMA_AA_15m	ns	>0.9999
GTP:PMA_2DG_15m vs. CTP:PMA_IAA_15m	ns	0.5143
GTP:PMA_2DG_15m vs. Uridine:PMA_15m	ns	>0.9999
GTP:PMA_2DG_15m vs. Uridine:PMA_2DG_15m	ns	0.6211
GTP:PMA_2DG_15m vs. Uridine:PMA_6AN_15m	ns	0.5766
GTP:PMA_2DG_15m vs. Uridine:PMA_DPI_15m	ns	>0.9999
GTP:PMA_2DG_15m vs. Uridine:PMA_AA_15m	ns	0.4572
GTP:PMA_2DG_15m vs. Uridine:PMA_IAA_15m	ns	>0.9999
GTP:PMA_2DG_15m vs. UMP:PMA_15m	ns	0.9999
GTP:PMA_2DG_15m vs. UMP:PMA_2DG_15m	ns	0.0853
GTP:PMA_2DG_15m vs. UMP:PMA_6AN_15m	ns	0.1007
GTP:PMA_2DG_15m vs. UMP:PMA_DPI_15m	ns	0.9791
GTP:PMA_2DG_15m vs. UMP:PMA_AA_15m	ns	0.0653
GTP:PMA_2DG_15m vs. UMP:PMA_IAA_15m	ns	>0.9999
GTP:PMA_2DG_15m vs. UDP:PMA_15m	ns	>0.9999
GTP:PMA_2DG_15m vs. UDP:PMA_2DG_15m	ns	0.0914
GTP:PMA_2DG_15m vs. UDP:PMA_6AN_15m	ns	0.1068
GTP:PMA_2DG_15m vs. UDP:PMA_DPI_15m	ns	0.9819
GTP:PMA_2DG_15m vs. UDP:PMA_AA_15m	ns	0.0695
GTP:PMA_2DG_15m vs. UDP:PMA_IAA_15m	ns	>0.9999
GTP:PMA_2DG_15m vs. UTP:PMA_15m	ns	>0.9999
GTP:PMA_2DG_15m vs. UTP:PMA_2DG_15m	ns	>0.9999
GTP:PMA_2DG_15m vs. UTP:PMA_6AN_15m	ns	>0.9999
GTP:PMA_2DG_15m vs. UTP:PMA_DPI_15m	ns	>0.9999
GTP:PMA_2DG_15m vs. UTP:PMA_AA_15m	ns	>0.9999
GTP:PMA_2DG_15m vs. UTP:PMA_IAA_15m	ns	0.3552
GTP:PMA_6AN_15m vs. GTP:PMA_DPI_15m	ns	0.8059

GTP:PMA_6AN_15m vs. GTP:PMA_AA_15m	ns	>0.9999
GTP:PMA_6AN_15m vs. GTP:PMA_IAA_15m	****	<0.0001
GTP:PMA_6AN_15m vs. Guanosine:PMA_15m	ns	>0.9999
GTP:PMA_6AN_15m vs. Guanosine:PMA_2DG_15m	ns	0.991
GTP:PMA_6AN_15m vs. Guanosine:PMA_6AN_15m	ns	0.5324
GTP:PMA_6AN_15m vs. Guanosine:PMA_DPI_15m	ns	>0.9999
GTP:PMA_6AN_15m vs. Guanosine:PMA_AA_15m	ns	0.7645
GTP:PMA_6AN_15m vs. Guanosine:PMA_IAA_15m	ns	>0.9999
GTP:PMA_6AN_15m vs. Guanine:PMA_15m	ns	>0.9999
GTP:PMA_6AN_15m vs. Guanine:PMA_2DG_15m	ns	>0.9999
GTP:PMA_6AN_15m vs. Guanine:PMA_6AN_15m	ns	>0.9999
GTP:PMA_6AN_15m vs. Guanine:PMA_DPI_15m	ns	>0.9999
GTP:PMA_6AN_15m vs. Guanine:PMA_AA_15m	ns	>0.9999
GTP:PMA_6AN_15m vs. Guanine:PMA_IAA_15m	ns	0.6387
GTP:PMA_6AN_15m vs. Aspartic acid:PMA_15m	ns	>0.9999
GTP:PMA_6AN_15m vs. Aspartic acid:PMA_2DG_15m	ns	>0.9999
GTP:PMA_6AN_15m vs. Aspartic acid:PMA_6AN_15m	ns	>0.9999
GTP:PMA_6AN_15m vs. Aspartic acid:PMA_DPI_15m	ns	>0.9999
GTP:PMA_6AN_15m vs. Aspartic acid:PMA_AA_15m	ns	>0.9999
GTP:PMA_6AN_15m vs. Aspartic acid:PMA_IAA_15m	ns	0.8456
GTP:PMA_6AN_15m vs. AMP:PMA_15m	ns	>0.9999
GTP:PMA_6AN_15m vs. AMP:PMA_2DG_15m	ns	>0.9999
GTP:PMA_6AN_15m vs. AMP:PMA_6AN_15m	ns	0.8908
GTP:PMA_6AN_15m vs. AMP:PMA_DPI_15m	ns	>0.9999
GTP:PMA_6AN_15m vs. AMP:PMA_AA_15m	ns	0.967
GTP:PMA_6AN_15m vs. AMP:PMA_IAA_15m	ns	>0.9999
GTP:PMA_6AN_15m vs. ADP:PMA_15m	ns	>0.9999
GTP:PMA_6AN_15m vs. ADP:PMA_2DG_15m	ns	>0.9999
GTP:PMA_6AN_15m vs. ADP:PMA_6AN_15m	ns	0.9919
GTP:PMA_6AN_15m vs. ADP:PMA_DPI_15m	ns	>0.9999
GTP:PMA_6AN_15m vs. ADP:PMA_AA_15m	ns	0.9985
GTP:PMA_6AN_15m vs. ADP:PMA_IAA_15m	ns	>0.9999
GTP:PMA_6AN_15m vs. ATP:PMA_15m	ns	0.9989
GTP:PMA_6AN_15m vs. ATP:PMA_2DG_15m	ns	>0.9999
GTP:PMA_6AN_15m vs. ATP:PMA_6AN_15m	ns	>0.9999
GTP:PMA_6AN_15m vs. ATP:PMA_DPI_15m	ns	>0.9999
GTP:PMA_6AN_15m vs. ATP:PMA_AA_15m	ns	>0.9999
GTP:PMA_6AN_15m vs. ATP:PMA_IAA_15m	*	0.0257
GTP:PMA_6AN_15m vs. Xanthine:PMA_15m	ns	>0.9999
GTP:PMA_6AN_15m vs. Xanthine:PMA_2DG_15m	ns	>0.9999
GTP:PMA_6AN_15m vs. Xanthine:PMA_6AN_15m	ns	>0.9999
GTP:PMA_6AN_15m vs. Xanthine:PMA_DPI_15m	ns	>0.9999
GTP:PMA_6AN_15m vs. Xanthine:PMA_AA_15m	ns	>0.9999
GTP:PMA_6AN_15m vs. CMP:PMA_15m	ns	>0.9999
GTP:PMA_6AN_15m vs. CMP:PMA_2DG_15m	ns	0.9996
GTP:PMA_6AN_15m vs. CMP:PMA_6AN_15m	ns	0.8031
GTP:PMA_6AN_15m vs. CMP:PMA_DPI_15m	ns	>0.9999

GTP:PMA_6AN_15m vs. CMP:PMA_AA_15m	ns	0.9296
GTP:PMA_6AN_15m vs. CMP:PMA_IAA_15m	ns	>0.9999
GTP:PMA_6AN_15m vs. CDP:PMA_15m	ns	>0.9999
GTP:PMA_6AN_15m vs. CDP:PMA_2DG_15m	ns	0.94
GTP:PMA_6AN_15m vs. CDP:PMA_6AN_15m	ns	0.2871
GTP:PMA_6AN_15m vs. CDP:PMA_DPI_15m	ns	>0.9999
GTP:PMA_6AN_15m vs. CDP:PMA_AA_15m	ns	0.5361
GTP:PMA_6AN_15m vs. CDP:PMA_IAA_15m	ns	>0.9999
GTP:PMA_6AN_15m vs. CTP:PMA_15m	ns	>0.9999
GTP:PMA_6AN_15m vs. CTP:PMA_2DG_15m	ns	>0.9999
GTP:PMA_6AN_15m vs. CTP:PMA_6AN_15m	ns	>0.9999
GTP:PMA_6AN_15m vs. CTP:PMA_DPI_15m	ns	>0.9999
GTP:PMA_6AN_15m vs. CTP:PMA_AA_15m	ns	>0.9999
GTP:PMA_6AN_15m vs. CTP:PMA_IAA_15m	ns	0.1919
GTP:PMA_6AN_15m vs. Uridine:PMA_15m	ns	>0.9999
GTP:PMA_6AN_15m vs. Uridine:PMA_2DG_15m	ns	0.996
GTP:PMA_6AN_15m vs. Uridine:PMA_6AN_15m	ns	0.6211
GTP:PMA_6AN_15m vs. Uridine:PMA_DPI_15m	ns	>0.9999
GTP:PMA_6AN_15m vs. Uridine:PMA_AA_15m	ns	0.8271
GTP:PMA_6AN_15m vs. Uridine:PMA_IAA_15m	ns	>0.9999
GTP:PMA_6AN_15m vs. UMP:PMA_15m	ns	>0.9999
GTP:PMA_6AN_15m vs. UMP:PMA_2DG_15m	ns	0.7103
GTP:PMA_6AN_15m vs. UMP:PMA_6AN_15m	ns	0.0853
GTP:PMA_6AN_15m vs. UMP:PMA_DPI_15m	ns	0.9998
GTP:PMA_6AN_15m vs. UMP:PMA_AA_15m	ns	0.2406
GTP:PMA_6AN_15m vs. UMP:PMA_IAA_15m	ns	>0.9999
GTP:PMA_6AN_15m vs. UDP:PMA_15m	ns	>0.9999
GTP:PMA_6AN_15m vs. UDP:PMA_2DG_15m	ns	0.7261
GTP:PMA_6AN_15m vs. UDP:PMA_6AN_15m	ns	0.0914
GTP:PMA_6AN_15m vs. UDP:PMA_DPI_15m	ns	0.9999
GTP:PMA_6AN_15m vs. UDP:PMA_AA_15m	ns	0.2525
GTP:PMA_6AN_15m vs. UDP:PMA_IAA_15m	ns	>0.9999
GTP:PMA_6AN_15m vs. UTP:PMA_15m	ns	>0.9999
GTP:PMA_6AN_15m vs. UTP:PMA_2DG_15m	ns	>0.9999
GTP:PMA_6AN_15m vs. UTP:PMA_6AN_15m	ns	>0.9999
GTP:PMA_6AN_15m vs. UTP:PMA_DPI_15m	ns	>0.9999
GTP:PMA_6AN_15m vs. UTP:PMA_AA_15m	ns	>0.9999
GTP:PMA_6AN_15m vs. UTP:PMA_IAA_15m	ns	0.1111
GTP:PMA_DPI_15m vs. GTP:PMA_AA_15m	ns	0.5731
GTP:PMA_DPI_15m vs. GTP:PMA_IAA_15m	***	0.0004
GTP:PMA_DPI_15m vs. Guanosine:PMA_15m	ns	0.9936
GTP:PMA_DPI_15m vs. Guanosine:PMA_2DG_15m	ns	0.1381
GTP:PMA_DPI_15m vs. Guanosine:PMA_6AN_15m	*	0.0323
GTP:PMA_DPI_15m vs. Guanosine:PMA_DPI_15m	ns	0.5324
GTP:PMA_DPI_15m vs. Guanosine:PMA_AA_15m	*	0.0196
GTP:PMA_DPI_15m vs. Guanosine:PMA_IAA_15m	ns	>0.9999
GTP:PMA_DPI_15m vs. Guanine:PMA_15m	ns	>0.9999

GTP:PMA_DPI_15m vs. Guanine:PMA_2DG_15m	ns	>0.9999
GTP:PMA_DPI_15m vs. Guanine:PMA_6AN_15m	ns	0.9981
GTP:PMA_DPI_15m vs. Guanine:PMA_DPI_15m	ns	>0.9999
GTP:PMA_DPI_15m vs. Guanine:PMA_AA_15m	ns	0.9925
GTP:PMA_DPI_15m vs. Guanine:PMA_IAA_15m	ns	>0.9999
GTP:PMA_DPI_15m vs. Aspartic acid:PMA_15m	ns	>0.9999
GTP:PMA_DPI_15m vs. Aspartic acid:PMA_2DG_15m	ns	0.9998
GTP:PMA_DPI_15m vs. Aspartic acid:PMA_6AN_15m	ns	0.9777
GTP:PMA_DPI_15m vs. Aspartic acid:PMA_DPI_15m	ns	>0.9999
GTP:PMA_DPI_15m vs. Aspartic acid:PMA_AA_15m	ns	0.9459
GTP:PMA_DPI_15m vs. Aspartic acid:PMA_IAA_15m	ns	>0.9999
GTP:PMA_DPI_15m vs. AMP:PMA_15m	ns	>0.9999
GTP:PMA_DPI_15m vs. AMP:PMA_2DG_15m	ns	0.387
GTP:PMA_DPI_15m vs. AMP:PMA_6AN_15m	ns	0.1248
GTP:PMA_DPI_15m vs. AMP:PMA_DPI_15m	ns	0.8908
GTP:PMA_DPI_15m vs. AMP:PMA_AA_15m	ns	0.0822
GTP:PMA_DPI_15m vs. AMP:PMA_IAA_15m	ns	>0.9999
GTP:PMA_DPI_15m vs. ADP:PMA_15m	ns	>0.9999
GTP:PMA_DPI_15m vs. ADP:PMA_2DG_15m	ns	0.6873
GTP:PMA_DPI_15m vs. ADP:PMA_6AN_15m	ns	0.3122
GTP:PMA_DPI_15m vs. ADP:PMA_DPI_15m	ns	0.9919
GTP:PMA_DPI_15m vs. ADP:PMA_AA_15m	ns	0.2243
GTP:PMA_DPI_15m vs. ADP:PMA_IAA_15m	ns	>0.9999
GTP:PMA_DPI_15m vs. ATP:PMA_15m	ns	>0.9999
GTP:PMA_DPI_15m vs. ATP:PMA_2DG_15m	ns	>0.9999
GTP:PMA_DPI_15m vs. ATP:PMA_6AN_15m	ns	>0.9999
GTP:PMA_DPI_15m vs. ATP:PMA_DPI_15m	ns	>0.9999
GTP:PMA_DPI_15m vs. ATP:PMA_AA_15m	ns	>0.9999
GTP:PMA_DPI_15m vs. ATP:PMA_IAA_15m	ns	0.8133
GTP:PMA_DPI_15m vs. Xanthine:PMA_15m	ns	>0.9999
GTP:PMA_DPI_15m vs. Xanthine:PMA_2DG_15m	ns	>0.9999
GTP:PMA_DPI_15m vs. Xanthine:PMA_6AN_15m	ns	>0.9999
GTP:PMA_DPI_15m vs. Xanthine:PMA_DPI_15m	ns	>0.9999
GTP:PMA_DPI_15m vs. Xanthine:PMA_AA_15m	ns	0.9996
GTP:PMA_DPI_15m vs. CMP:PMA_15m	ns	0.9998
GTP:PMA_DPI_15m vs. CMP:PMA_2DG_15m	ns	0.2925
GTP:PMA_DPI_15m vs. CMP:PMA_6AN_15m	ns	0.0846
GTP:PMA_DPI_15m vs. CMP:PMA_DPI_15m	ns	0.8031
GTP:PMA_DPI_15m vs. CMP:PMA_AA_15m	ns	0.0542
GTP:PMA_DPI_15m vs. CMP:PMA_IAA_15m	ns	>0.9999
GTP:PMA_DPI_15m vs. CDP:PMA_15m	ns	0.9518
GTP:PMA_DPI_15m vs. CDP:PMA_2DG_15m	ns	0.0598
GTP:PMA_DPI_15m vs. CDP:PMA_6AN_15m	*	0.0119
GTP:PMA_DPI_15m vs. CDP:PMA_DPI_15m	ns	0.2871
GTP:PMA_DPI_15m vs. CDP:PMA_AA_15m	**	0.0069
GTP:PMA_DPI_15m vs. CDP:PMA_IAA_15m	ns	>0.9999
GTP:PMA_DPI_15m vs. CTP:PMA_15m	ns	>0.9999

GTP:PMA_DPI_15m vs. CTP:PMA_2DG_15m	ns	>0.9999
GTP:PMA_DPI_15m vs. CTP:PMA_6AN_15m	ns	>0.9999
GTP:PMA_DPI_15m vs. CTP:PMA_DPI_15m	ns	>0.9999
GTP:PMA_DPI_15m vs. CTP:PMA_AA_15m	ns	>0.9999
GTP:PMA_DPI_15m vs. CTP:PMA_IAA_15m	ns	0.9969
GTP:PMA_DPI_15m vs. Uridine:PMA_15m	ns	0.9973
GTP:PMA_DPI_15m vs. Uridine:PMA_2DG_15m	ns	0.1769
GTP:PMA_DPI_15m vs. Uridine:PMA_6AN_15m	*	0.044
GTP:PMA_DPI_15m vs. Uridine:PMA_DPI_15m	ns	0.6211
GTP:PMA_DPI_15m vs. Uridine:PMA_AA_15m	*	0.0271
GTP:PMA_DPI_15m vs. Uridine:PMA_IAA_15m	ns	>0.9999
GTP:PMA_DPI_15m vs. UMP:PMA_15m	ns	0.7408
GTP:PMA_DPI_15m vs. UMP:PMA_2DG_15m	*	0.0152
GTP:PMA_DPI_15m vs. UMP:PMA_6AN_15m	**	0.0025
GTP:PMA_DPI_15m vs. UMP:PMA_DPI_15m	ns	0.0853
GTP:PMA_DPI_15m vs. UMP:PMA_AA_15m	**	0.0014
GTP:PMA_DPI_15m vs. UMP:PMA_IAA_15m	ns	>0.9999
GTP:PMA_DPI_15m vs. UDP:PMA_15m	ns	0.756
GTP:PMA_DPI_15m vs. UDP:PMA_2DG_15m	*	0.0163
GTP:PMA_DPI_15m vs. UDP:PMA_6AN_15m	**	0.0027
GTP:PMA_DPI_15m vs. UDP:PMA_DPI_15m	ns	0.0914
GTP:PMA_DPI_15m vs. UDP:PMA_AA_15m	**	0.0015
GTP:PMA_DPI_15m vs. UDP:PMA_IAA_15m	ns	>0.9999
GTP:PMA_DPI_15m vs. UTP:PMA_15m	ns	>0.9999
GTP:PMA_DPI_15m vs. UTP:PMA_2DG_15m	ns	>0.9999
GTP:PMA_DPI_15m vs. UTP:PMA_6AN_15m	ns	>0.9999
GTP:PMA_DPI_15m vs. UTP:PMA_DPI_15m	ns	>0.9999
GTP:PMA_DPI_15m vs. UTP:PMA_AA_15m	ns	>0.9999
GTP:PMA_DPI_15m vs. UTP:PMA_IAA_15m	ns	0.9829
GTP:PMA_AA_15m vs. GTP:PMA_IAA_15m	****	<0.0001
GTP:PMA_AA_15m vs. Guanosine:PMA_15m	ns	>0.9999
GTP:PMA_AA_15m vs. Guanosine:PMA_2DG_15m	ns	0.9976
GTP:PMA_AA_15m vs. Guanosine:PMA_6AN_15m	ns	0.9245
GTP:PMA_AA_15m vs. Guanosine:PMA_DPI_15m	ns	>0.9999
GTP:PMA_AA_15m vs. Guanosine:PMA_AA_15m	ns	0.5324
GTP:PMA_AA_15m vs. Guanosine:PMA_IAA_15m	ns	>0.9999
GTP:PMA_AA_15m vs. Guanine:PMA_15m	ns	>0.9999
GTP:PMA_AA_15m vs. Guanine:PMA_2DG_15m	ns	>0.9999
GTP:PMA_AA_15m vs. Guanine:PMA_6AN_15m	ns	>0.9999
GTP:PMA_AA_15m vs. Guanine:PMA_DPI_15m	ns	>0.9999
GTP:PMA_AA_15m vs. Guanine:PMA_AA_15m	ns	>0.9999
GTP:PMA_AA_15m vs. Guanine:PMA_IAA_15m	ns	0.5189
GTP:PMA_AA_15m vs. Aspartic acid:PMA_15m	ns	>0.9999
GTP:PMA_AA_15m vs. Aspartic acid:PMA_2DG_15m	ns	>0.9999
GTP:PMA_AA_15m vs. Aspartic acid:PMA_6AN_15m	ns	>0.9999
GTP:PMA_AA_15m vs. Aspartic acid:PMA_DPI_15m	ns	>0.9999
GTP:PMA_AA_15m vs. Aspartic acid:PMA_AA_15m	ns	>0.9999

GTP:PMA_AA_15m vs. Aspartic acid:PMA_IAA_15m	ns	0.7501
GTP:PMA_AA_15m vs. AMP:PMA_15m	ns	>0.9999
GTP:PMA_AA_15m vs. AMP:PMA_2DG_15m	ns	>0.9999
GTP:PMA_AA_15m vs. AMP:PMA_6AN_15m	ns	0.9966
GTP:PMA_AA_15m vs. AMP:PMA_DPI_15m	ns	>0.9999
GTP:PMA_AA_15m vs. AMP:PMA_AA_15m	ns	0.8908
GTP:PMA_AA_15m vs. AMP:PMA_IAA_15m	ns	>0.9999
GTP:PMA_AA_15m vs. ADP:PMA_15m	ns	>0.9999
GTP:PMA_AA_15m vs. ADP:PMA_2DG_15m	ns	>0.9999
GTP:PMA_AA_15m vs. ADP:PMA_6AN_15m	ns	>0.9999
GTP:PMA_AA_15m vs. ADP:PMA_DPI_15m	ns	>0.9999
GTP:PMA_AA_15m vs. ADP:PMA_AA_15m	ns	0.9919
GTP:PMA_AA_15m vs. ADP:PMA_IAA_15m	ns	>0.9999
GTP:PMA_AA_15m vs. ATP:PMA_15m	ns	0.9951
GTP:PMA_AA_15m vs. ATP:PMA_2DG_15m	ns	>0.9999
GTP:PMA_AA_15m vs. ATP:PMA_6AN_15m	ns	>0.9999
GTP:PMA_AA_15m vs. ATP:PMA_DPI_15m	ns	>0.9999
GTP:PMA_AA_15m vs. ATP:PMA_AA_15m	ns	>0.9999
GTP:PMA_AA_15m vs. ATP:PMA_IAA_15m	*	0.0155
GTP:PMA_AA_15m vs. Xanthine:PMA_15m	ns	>0.9999
GTP:PMA_AA_15m vs. Xanthine:PMA_2DG_15m	ns	>0.9999
GTP:PMA_AA_15m vs. Xanthine:PMA_6AN_15m	ns	>0.9999
GTP:PMA_AA_15m vs. Xanthine:PMA_DPI_15m	ns	>0.9999
GTP:PMA_AA_15m vs. Xanthine:PMA_AA_15m	ns	>0.9999
GTP:PMA_AA_15m vs. CMP:PMA_15m	ns	>0.9999
GTP:PMA_AA_15m vs. CMP:PMA_2DG_15m	ns	>0.9999
GTP:PMA_AA_15m vs. CMP:PMA_6AN_15m	ns	0.9889
GTP:PMA_AA_15m vs. CMP:PMA_DPI_15m	ns	>0.9999
GTP:PMA_AA_15m vs. CMP:PMA_AA_15m	ns	0.8031
GTP:PMA_AA_15m vs. CMP:PMA_IAA_15m	ns	>0.9999
GTP:PMA_AA_15m vs. CDP:PMA_15m	ns	>0.9999
GTP:PMA_AA_15m vs. CDP:PMA_2DG_15m	ns	0.9746
GTP:PMA_AA_15m vs. CDP:PMA_6AN_15m	ns	0.7674
GTP:PMA_AA_15m vs. CDP:PMA_DPI_15m	ns	>0.9999
GTP:PMA_AA_15m vs. CDP:PMA_AA_15m	ns	0.2871
GTP:PMA_AA_15m vs. CDP:PMA_IAA_15m	ns	>0.9999
GTP:PMA_AA_15m vs. CTP:PMA_15m	ns	>0.9999
GTP:PMA_AA_15m vs. CTP:PMA_2DG_15m	ns	>0.9999
GTP:PMA_AA_15m vs. CTP:PMA_6AN_15m	ns	>0.9999
GTP:PMA_AA_15m vs. CTP:PMA_DPI_15m	ns	>0.9999
GTP:PMA_AA_15m vs. CTP:PMA_AA_15m	ns	>0.9999
GTP:PMA_AA_15m vs. CTP:PMA_IAA_15m	ns	0.1311
GTP:PMA_AA_15m vs. Uridine:PMA_15m	ns	>0.9999
GTP:PMA_AA_15m vs. Uridine:PMA_2DG_15m	ns	0.9991
GTP:PMA_AA_15m vs. Uridine:PMA_6AN_15m	ns	0.9541
GTP:PMA_AA_15m vs. Uridine:PMA_DPI_15m	ns	>0.9999
GTP:PMA_AA_15m vs. Uridine:PMA_AA_15m	ns	0.6211

GTP:PMA_AA_15m vs. Uridine:PMA_IAA_15m	ns	>0.9999
GTP:PMA_AA_15m vs. UMP:PMA_15m	ns	>0.9999
GTP:PMA_AA_15m vs. UMP:PMA_2DG_15m	ns	0.8137
GTP:PMA_AA_15m vs. UMP:PMA_6AN_15m	ns	0.4403
GTP:PMA_AA_15m vs. UMP:PMA_DPI_15m	ns	>0.9999
GTP:PMA_AA_15m vs. UMP:PMA_AA_15m	ns	0.0853
GTP:PMA_AA_15m vs. UMP:PMA_IAA_15m	ns	>0.9999
GTP:PMA_AA_15m vs. UDP:PMA_15m	ns	>0.9999
GTP:PMA_AA_15m vs. UDP:PMA_2DG_15m	ns	0.8266
GTP:PMA_AA_15m vs. UDP:PMA_6AN_15m	ns	0.4567
GTP:PMA_AA_15m vs. UDP:PMA_DPI_15m	ns	>0.9999
GTP:PMA_AA_15m vs. UDP:PMA_AA_15m	ns	0.0914
GTP:PMA_AA_15m vs. UDP:PMA_IAA_15m	ns	>0.9999
GTP:PMA_AA_15m vs. UTP:PMA_15m	ns	>0.9999
GTP:PMA_AA_15m vs. UTP:PMA_2DG_15m	ns	>0.9999
GTP:PMA_AA_15m vs. UTP:PMA_6AN_15m	ns	>0.9999
GTP:PMA_AA_15m vs. UTP:PMA_DPI_15m	ns	>0.9999
GTP:PMA_AA_15m vs. UTP:PMA_AA_15m	ns	>0.9999
GTP:PMA_AA_15m vs. UTP:PMA_IAA_15m	ns	0.0726
GTP:PMA_IAA_15m vs. Guanosine:PMA_15m	**	0.002
GTP:PMA_IAA_15m vs. Guanosine:PMA_2DG_15m	****	<0.0001
GTP:PMA_IAA_15m vs. Guanosine:PMA_6AN_15m	****	<0.0001
GTP:PMA_IAA_15m vs. Guanosine:PMA_DPI_15m	***	0.0002
GTP:PMA_IAA_15m vs. Guanosine:PMA_AA_15m	****	<0.0001
GTP:PMA_IAA_15m vs. Guanosine:PMA_IAA_15m	ns	0.5324
GTP:PMA_IAA_15m vs. Guanine:PMA_15m	ns	0.7811
GTP:PMA_IAA_15m vs. Guanine:PMA_2DG_15m	*	0.0189
GTP:PMA_IAA_15m vs. Guanine:PMA_6AN_15m	**	0.0032
GTP:PMA_IAA_15m vs. Guanine:PMA_DPI_15m	ns	0.3736
GTP:PMA_IAA_15m vs. Guanine:PMA_AA_15m	**	0.0018
GTP:PMA_IAA_15m vs. Guanine:PMA_IAA_15m	ns	>0.9999
GTP:PMA_IAA_15m vs. Aspartic acid:PMA_15m	ns	0.5549
GTP:PMA_IAA_15m vs. Aspartic acid:PMA_2DG_15m	**	0.0066
GTP:PMA_IAA_15m vs. Aspartic acid:PMA_6AN_15m	**	0.001
GTP:PMA_IAA_15m vs. Aspartic acid:PMA_DPI_15m	ns	0.1962
GTP:PMA_IAA_15m vs. Aspartic acid:PMA_AA_15m	***	0.0006
GTP:PMA_IAA_15m vs. Aspartic acid:PMA_IAA_15m	ns	>0.9999
GTP:PMA_IAA_15m vs. AMP:PMA_15m	*	0.0105
GTP:PMA_IAA_15m vs. AMP:PMA_2DG_15m	****	<0.0001
GTP:PMA_IAA_15m vs. AMP:PMA_6AN_15m	****	<0.0001
GTP:PMA_IAA_15m vs. AMP:PMA_DPI_15m	**	0.0014
GTP:PMA_IAA_15m vs. AMP:PMA_AA_15m	****	<0.0001
GTP:PMA_IAA_15m vs. AMP:PMA_IAA_15m	ns	0.8908
GTP:PMA_IAA_15m vs. ADP:PMA_15m	*	0.0383
GTP:PMA_IAA_15m vs. ADP:PMA_2DG_15m	****	<0.0001
GTP:PMA_IAA_15m vs. ADP:PMA_6AN_15m	****	<0.0001
GTP:PMA_IAA_15m vs. ADP:PMA_DPI_15m	**	0.0061

GTP:PMA_IAA_15m vs. ADP:PMA_AA_15m	****	<0.0001
GTP:PMA_IAA_15m vs. ADP:PMA_IAA_15m	ns	0.9919
GTP:PMA_IAA_15m vs. ATP:PMA_15m	ns	>0.9999
GTP:PMA_IAA_15m vs. ATP:PMA_2DG_15m	ns	0.5656
GTP:PMA_IAA_15m vs. ATP:PMA_6AN_15m	ns	0.2234
GTP:PMA_IAA_15m vs. ATP:PMA_DPI_15m	ns	0.9984
GTP:PMA_IAA_15m vs. ATP:PMA_AA_15m	ns	0.1549
GTP:PMA_IAA_15m vs. ATP:PMA_IAA_15m	ns	>0.9999
GTP:PMA_IAA_15m vs. Xanthine:PMA_15m	ns	0.9116
GTP:PMA_IAA_15m vs. Xanthine:PMA_2DG_15m	*	0.0452
GTP:PMA_IAA_15m vs. Xanthine:PMA_6AN_15m	**	0.0089
GTP:PMA_IAA_15m vs. Xanthine:PMA_DPI_15m	ns	0.5669
GTP:PMA_IAA_15m vs. Xanthine:PMA_AA_15m	**	0.0052
GTP:PMA_IAA_15m vs. CMP:PMA_15m	**	0.0064
GTP:PMA_IAA_15m vs. CMP:PMA_2DG_15m	****	<0.0001
GTP:PMA_IAA_15m vs. CMP:PMA_6AN_15m	****	<0.0001
GTP:PMA_IAA_15m vs. CMP:PMA_DPI_15m	***	0.0008
GTP:PMA_IAA_15m vs. CMP:PMA_AA_15m	****	<0.0001
GTP:PMA_IAA_15m vs. CMP:PMA_IAA_15m	ns	0.8031
GTP:PMA_IAA_15m vs. CDP:PMA_15m	***	0.0006
GTP:PMA_IAA_15m vs. CDP:PMA_2DG_15m	****	<0.0001
GTP:PMA_IAA_15m vs. CDP:PMA_6AN_15m	****	<0.0001
GTP:PMA_IAA_15m vs. CDP:PMA_DPI_15m	****	<0.0001
GTP:PMA_IAA_15m vs. CDP:PMA_AA_15m	****	<0.0001
GTP:PMA_IAA_15m vs. CDP:PMA_IAA_15m	ns	0.2871
GTP:PMA_IAA_15m vs. CTP:PMA_15m	ns	0.9928
GTP:PMA_IAA_15m vs. CTP:PMA_2DG_15m	ns	0.1352
GTP:PMA_IAA_15m vs. CTP:PMA_6AN_15m	*	0.0316
GTP:PMA_IAA_15m vs. CTP:PMA_DPI_15m	ns	0.8507
GTP:PMA_IAA_15m vs. CTP:PMA_AA_15m	*	0.0193
GTP:PMA_IAA_15m vs. CTP:PMA_IAA_15m	ns	>0.9999
GTP:PMA_IAA_15m vs. Uridine:PMA_15m	**	0.0028
GTP:PMA_IAA_15m vs. Uridine:PMA_2DG_15m	****	<0.0001
GTP:PMA_IAA_15m vs. Uridine:PMA_6AN_15m	****	<0.0001
GTP:PMA_IAA_15m vs. Uridine:PMA_DPI_15m	***	0.0003
GTP:PMA_IAA_15m vs. Uridine:PMA_AA_15m	****	<0.0001
GTP:PMA_IAA_15m vs. Uridine:PMA_IAA_15m	ns	0.6211
GTP:PMA_IAA_15m vs. UMP:PMA_15m	***	0.0001
GTP:PMA_IAA_15m vs. UMP:PMA_2DG_15m	****	<0.0001
GTP:PMA_IAA_15m vs. UMP:PMA_6AN_15m	****	<0.0001
GTP:PMA_IAA_15m vs. UMP:PMA_DPI_15m	****	<0.0001
GTP:PMA_IAA_15m vs. UMP:PMA_AA_15m	****	<0.0001
GTP:PMA_IAA_15m vs. UMP:PMA_IAA_15m	ns	0.0853
GTP:PMA_IAA_15m vs. UDP:PMA_15m	***	0.0001
GTP:PMA_IAA_15m vs. UDP:PMA_2DG_15m	****	<0.0001
GTP:PMA_IAA_15m vs. UDP:PMA_6AN_15m	****	<0.0001
GTP:PMA_IAA_15m vs. UDP:PMA_DPI_15m	****	<0.0001

GTP:PMA_IAA_15m vs. UDP:PMA_AA_15m	****	<0.0001
GTP:PMA_IAA_15m vs. UDP:PMA_IAA_15m	ns	0.0914
GTP:PMA_IAA_15m vs. UTP:PMA_15m	ns	0.999
GTP:PMA_IAA_15m vs. UTP:PMA_2DG_15m	ns	0.2283
GTP:PMA_IAA_15m vs. UTP:PMA_6AN_15m	ns	0.0612
GTP:PMA_IAA_15m vs. UTP:PMA_DPI_15m	ns	0.9407
GTP:PMA_IAA_15m vs. UTP:PMA_AA_15m	*	0.0385
GTP:PMA_IAA_15m vs. UTP:PMA_IAA_15m	ns	>0.9999
Guanosine:PMA_15m vs. Guanosine:PMA_2DG_15m	ns	0.7475
Guanosine:PMA_15m vs. Guanosine:PMA_6AN_15m	ns	0.1037
Guanosine:PMA_15m vs. Guanosine:PMA_DPI_15m	ns	>0.9999
Guanosine:PMA_15m vs. Guanosine:PMA_AA_15m	*	0.0409
Guanosine:PMA_15m vs. Guanosine:PMA_IAA_15m	*	0.0236
Guanosine:PMA_15m vs. Guanine:PMA_15m	ns	0.9977
Guanosine:PMA_15m vs. Guanine:PMA_2DG_15m	ns	>0.9999
Guanosine:PMA_15m vs. Guanine:PMA_6AN_15m	ns	>0.9999
Guanosine:PMA_15m vs. Guanine:PMA_DPI_15m	ns	>0.9999
Guanosine:PMA_15m vs. Guanine:PMA_AA_15m	ns	>0.9999
Guanosine:PMA_15m vs. Guanine:PMA_IAA_15m	ns	0.0565
Guanosine:PMA_15m vs. Aspartic acid:PMA_15m	ns	>0.9999
Guanosine:PMA_15m vs. Aspartic acid:PMA_2DG_15m	ns	>0.9999
Guanosine:PMA_15m vs. Aspartic acid:PMA_6AN_15m	ns	>0.9999
Guanosine:PMA_15m vs. Aspartic acid:PMA_DPI_15m	ns	>0.9999
Guanosine:PMA_15m vs. Aspartic acid:PMA_AA_15m	ns	>0.9999
Guanosine:PMA_15m vs. Aspartic acid:PMA_IAA_15m	ns	0.1319
Guanosine:PMA_15m vs. AMP:PMA_15m	ns	>0.9999
Guanosine:PMA_15m vs. AMP:PMA_2DG_15m	ns	>0.9999
Guanosine:PMA_15m vs. AMP:PMA_6AN_15m	ns	>0.9999
Guanosine:PMA_15m vs. AMP:PMA_DPI_15m	ns	>0.9999
Guanosine:PMA_15m vs. AMP:PMA_AA_15m	ns	>0.9999
Guanosine:PMA_15m vs. AMP:PMA_IAA_15m	ns	0.9798
Guanosine:PMA_15m vs. ADP:PMA_15m	ns	>0.9999
Guanosine:PMA_15m vs. ADP:PMA_2DG_15m	ns	>0.9999
Guanosine:PMA_15m vs. ADP:PMA_6AN_15m	ns	>0.9999
Guanosine:PMA_15m vs. ADP:PMA_DPI_15m	ns	>0.9999
Guanosine:PMA_15m vs. ADP:PMA_AA_15m	ns	>0.9999
Guanosine:PMA_15m vs. ADP:PMA_IAA_15m	ns	0.857
Guanosine:PMA_15m vs. ATP:PMA_15m	ns	0.2322
Guanosine:PMA_15m vs. ATP:PMA_2DG_15m	ns	>0.9999
Guanosine:PMA_15m vs. ATP:PMA_6AN_15m	ns	>0.9999
Guanosine:PMA_15m vs. ATP:PMA_DPI_15m	ns	0.9261
Guanosine:PMA_15m vs. ATP:PMA_AA_15m	ns	>0.9999
Guanosine:PMA_15m vs. ATP:PMA_IAA_15m	***	0.0004
Guanosine:PMA_15m vs. Xanthine:PMA_15m	ns	0.9951
Guanosine:PMA_15m vs. Xanthine:PMA_2DG_15m	ns	>0.9999
Guanosine:PMA_15m vs. Xanthine:PMA_6AN_15m	ns	>0.9999
Guanosine:PMA_15m vs. Xanthine:PMA_DPI_15m	ns	>0.9999

Guanosine:PMA_15m vs. Xanthine:PMA_AA_15m	ns	>0.9999
Guanosine:PMA_15m vs. CMP:PMA_15m	ns	>0.9999
Guanosine:PMA_15m vs. CMP:PMA_2DG_15m	ns	>0.9999
Guanosine:PMA_15m vs. CMP:PMA_6AN_15m	ns	>0.9999
Guanosine:PMA_15m vs. CMP:PMA_DPI_15m	ns	>0.9999
Guanosine:PMA_15m vs. CMP:PMA_AA_15m	ns	>0.9999
Guanosine:PMA_15m vs. CMP:PMA_IAA_15m	ns	0.9929
Guanosine:PMA_15m vs. CDP:PMA_15m	ns	>0.9999
Guanosine:PMA_15m vs. CDP:PMA_2DG_15m	ns	>0.9999
Guanosine:PMA_15m vs. CDP:PMA_6AN_15m	ns	0.9996
Guanosine:PMA_15m vs. CDP:PMA_DPI_15m	ns	>0.9999
Guanosine:PMA_15m vs. CDP:PMA_AA_15m	ns	0.9977
Guanosine:PMA_15m vs. CDP:PMA_IAA_15m	ns	>0.9999
Guanosine:PMA_15m vs. CTP:PMA_15m	ns	0.8038
Guanosine:PMA_15m vs. CTP:PMA_2DG_15m	ns	>0.9999
Guanosine:PMA_15m vs. CTP:PMA_6AN_15m	ns	>0.9999
Guanosine:PMA_15m vs. CTP:PMA_DPI_15m	ns	0.9998
Guanosine:PMA_15m vs. CTP:PMA_AA_15m	ns	>0.9999
Guanosine:PMA_15m vs. CTP:PMA_IAA_15m	**	0.0064
Guanosine:PMA_15m vs. Uridine:PMA_15m	ns	>0.9999
Guanosine:PMA_15m vs. Uridine:PMA_2DG_15m	ns	>0.9999
Guanosine:PMA_15m vs. Uridine:PMA_6AN_15m	ns	>0.9999
Guanosine:PMA_15m vs. Uridine:PMA_DPI_15m	ns	>0.9999
Guanosine:PMA_15m vs. Uridine:PMA_AA_15m	ns	>0.9999
Guanosine:PMA_15m vs. Uridine:PMA_IAA_15m	ns	0.9991
Guanosine:PMA_15m vs. UMP:PMA_15m	ns	>0.9999
Guanosine:PMA_15m vs. UMP:PMA_2DG_15m	ns	0.9998
Guanosine:PMA_15m vs. UMP:PMA_6AN_15m	ns	0.9791
Guanosine:PMA_15m vs. UMP:PMA_DPI_15m	ns	>0.9999
Guanosine:PMA_15m vs. UMP:PMA_AA_15m	ns	0.9486
Guanosine:PMA_15m vs. UMP:PMA_IAA_15m	ns	>0.9999
Guanosine:PMA_15m vs. UDP:PMA_15m	ns	>0.9999
Guanosine:PMA_15m vs. UDP:PMA_2DG_15m	ns	0.9999
Guanosine:PMA_15m vs. UDP:PMA_6AN_15m	ns	0.9819
Guanosine:PMA_15m vs. UDP:PMA_DPI_15m	ns	>0.9999
Guanosine:PMA_15m vs. UDP:PMA_AA_15m	ns	0.9542
Guanosine:PMA_15m vs. UDP:PMA_IAA_15m	ns	>0.9999
Guanosine:PMA_15m vs. UTP:PMA_15m	ns	0.6273
Guanosine:PMA_15m vs. UTP:PMA_2DG_15m	ns	>0.9999
Guanosine:PMA_15m vs. UTP:PMA_6AN_15m	ns	>0.9999
Guanosine:PMA_15m vs. UTP:PMA_DPI_15m	ns	0.9975
Guanosine:PMA_15m vs. UTP:PMA_AA_15m	ns	>0.9999
Guanosine:PMA_15m vs. UTP:PMA_IAA_15m	**	0.0029
Guanosine:PMA_2DG_15m vs. Guanosine:PMA_6AN_15m	ns	>0.9999
Guanosine:PMA_2DG_15m vs. Guanosine:PMA_DPI_15m	ns	>0.9999
Guanosine:PMA_2DG_15m vs. Guanosine:PMA_AA_15m	ns	>0.9999
Guanosine:PMA_2DG_15m vs. Guanosine:PMA_IAA_15m	****	<0.0001

Guanosine:PMA_2DG_15m vs. Guanine:PMA_15m	ns	0.3691
Guanosine:PMA_2DG_15m vs. Guanine:PMA_2DG_15m	ns	0.9977
Guanosine:PMA_2DG_15m vs. Guanine:PMA_6AN_15m	ns	>0.9999
Guanosine:PMA_2DG_15m vs. Guanine:PMA_DPI_15m	ns	0.7782
Guanosine:PMA_2DG_15m vs. Guanine:PMA_AA_15m	ns	>0.9999
Guanosine:PMA_2DG_15m vs. Guanine:PMA_IAA_15m	***	0.0001
Guanosine:PMA_2DG_15m vs. Aspartic acid:PMA_15m	ns	0.6002
Guanosine:PMA_2DG_15m vs. Aspartic acid:PMA_2DG_15m	ns	>0.9999
Guanosine:PMA_2DG_15m vs. Aspartic acid:PMA_6AN_15m	ns	>0.9999
Guanosine:PMA_2DG_15m vs. Aspartic acid:PMA_DPI_15m	ns	0.9307
Guanosine:PMA_2DG_15m vs. Aspartic acid:PMA_AA_15m	ns	>0.9999
Guanosine:PMA_2DG_15m vs. Aspartic acid:PMA_IAA_15m	***	0.0005
Guanosine:PMA_2DG_15m vs. AMP:PMA_15m	ns	>0.9999
Guanosine:PMA_2DG_15m vs. AMP:PMA_2DG_15m	ns	>0.9999
Guanosine:PMA_2DG_15m vs. AMP:PMA_6AN_15m	ns	>0.9999
Guanosine:PMA_2DG_15m vs. AMP:PMA_DPI_15m	ns	>0.9999
Guanosine:PMA_2DG_15m vs. AMP:PMA_AA_15m	ns	>0.9999
Guanosine:PMA_2DG_15m vs. AMP:PMA_IAA_15m	ns	0.0925
Guanosine:PMA_2DG_15m vs. ADP:PMA_15m	ns	0.9992
Guanosine:PMA_2DG_15m vs. ADP:PMA_2DG_15m	ns	>0.9999
Guanosine:PMA_2DG_15m vs. ADP:PMA_6AN_15m	ns	>0.9999
Guanosine:PMA_2DG_15m vs. ADP:PMA_DPI_15m	ns	>0.9999
Guanosine:PMA_2DG_15m vs. ADP:PMA_AA_15m	ns	>0.9999
Guanosine:PMA_2DG_15m vs. ADP:PMA_IAA_15m	*	0.0287
Guanosine:PMA_2DG_15m vs. ATP:PMA_15m	**	0.0076
Guanosine:PMA_2DG_15m vs. ATP:PMA_2DG_15m	ns	0.2322
Guanosine:PMA_2DG_15m vs. ATP:PMA_6AN_15m	ns	0.9103
Guanosine:PMA_2DG_15m vs. ATP:PMA_DPI_15m	*	0.0462
Guanosine:PMA_2DG_15m vs. ATP:PMA_AA_15m	ns	0.958
Guanosine:PMA_2DG_15m vs. ATP:PMA_IAA_15m	****	<0.0001
Guanosine:PMA_2DG_15m vs. Xanthine:PMA_15m	ns	0.343
Guanosine:PMA_2DG_15m vs. Xanthine:PMA_2DG_15m	ns	0.9951
Guanosine:PMA_2DG_15m vs. Xanthine:PMA_6AN_15m	ns	>0.9999
Guanosine:PMA_2DG_15m vs. Xanthine:PMA_DPI_15m	ns	0.7377
Guanosine:PMA_2DG_15m vs. Xanthine:PMA_AA_15m	ns	>0.9999
Guanosine:PMA_2DG_15m vs. CMP:PMA_15m	ns	>0.9999
Guanosine:PMA_2DG_15m vs. CMP:PMA_2DG_15m	ns	>0.9999
Guanosine:PMA_2DG_15m vs. CMP:PMA_6AN_15m	ns	>0.9999
Guanosine:PMA_2DG_15m vs. CMP:PMA_DPI_15m	ns	>0.9999
Guanosine:PMA_2DG_15m vs. CMP:PMA_AA_15m	ns	>0.9999
Guanosine:PMA_2DG_15m vs. CMP:PMA_IAA_15m	ns	0.1355
Guanosine:PMA_2DG_15m vs. CDP:PMA_15m	ns	>0.9999
Guanosine:PMA_2DG_15m vs. CDP:PMA_2DG_15m	ns	>0.9999
Guanosine:PMA_2DG_15m vs. CDP:PMA_6AN_15m	ns	>0.9999
Guanosine:PMA_2DG_15m vs. CDP:PMA_DPI_15m	ns	>0.9999
Guanosine:PMA_2DG_15m vs. CDP:PMA_AA_15m	ns	>0.9999
Guanosine:PMA_2DG_15m vs. CDP:PMA_IAA_15m	ns	0.4989

Guanosine:PMA_2DG_15m vs. CTP:PMA_15m	ns	0.0752
Guanosine:PMA_2DG_15m vs. CTP:PMA_2DG_15m	ns	0.8038
Guanosine:PMA_2DG_15m vs. CTP:PMA_6AN_15m	ns	0.9996
Guanosine:PMA_2DG_15m vs. CTP:PMA_DPI_15m	ns	0.293
Guanosine:PMA_2DG_15m vs. CTP:PMA_AA_15m	ns	>0.9999
Guanosine:PMA_2DG_15m vs. CTP:PMA_IAA_15m	****	<0.0001
Guanosine:PMA_2DG_15m vs. Uridine:PMA_15m	ns	>0.9999
Guanosine:PMA_2DG_15m vs. Uridine:PMA_2DG_15m	ns	>0.9999
Guanosine:PMA_2DG_15m vs. Uridine:PMA_6AN_15m	ns	>0.9999
Guanosine:PMA_2DG_15m vs. Uridine:PMA_DPI_15m	ns	>0.9999
Guanosine:PMA_2DG_15m vs. Uridine:PMA_AA_15m	ns	>0.9999
Guanosine:PMA_2DG_15m vs. Uridine:PMA_IAA_15m	ns	0.2319
Guanosine:PMA_2DG_15m vs. UMP:PMA_15m	ns	>0.9999
Guanosine:PMA_2DG_15m vs. UMP:PMA_2DG_15m	ns	>0.9999
Guanosine:PMA_2DG_15m vs. UMP:PMA_6AN_15m	ns	>0.9999
Guanosine:PMA_2DG_15m vs. UMP:PMA_DPI_15m	ns	>0.9999
Guanosine:PMA_2DG_15m vs. UMP:PMA_AA_15m	ns	>0.9999
Guanosine:PMA_2DG_15m vs. UMP:PMA_IAA_15m	ns	0.8155
Guanosine:PMA_2DG_15m vs. UDP:PMA_15m	ns	>0.9999
Guanosine:PMA_2DG_15m vs. UDP:PMA_2DG_15m	ns	>0.9999
Guanosine:PMA_2DG_15m vs. UDP:PMA_6AN_15m	ns	>0.9999
Guanosine:PMA_2DG_15m vs. UDP:PMA_DPI_15m	ns	>0.9999
Guanosine:PMA_2DG_15m vs. UDP:PMA_AA_15m	ns	>0.9999
Guanosine:PMA_2DG_15m vs. UDP:PMA_IAA_15m	ns	0.8021
Guanosine:PMA_2DG_15m vs. UTP:PMA_15m	*	0.0394
Guanosine:PMA_2DG_15m vs. UTP:PMA_2DG_15m	ns	0.6273
Guanosine:PMA_2DG_15m vs. UTP:PMA_6AN_15m	ns	0.9963
Guanosine:PMA_2DG_15m vs. UTP:PMA_DPI_15m	ns	0.18
Guanosine:PMA_2DG_15m vs. UTP:PMA_AA_15m	ns	0.9992
Guanosine:PMA_2DG_15m vs. UTP:PMA_IAA_15m	****	<0.0001
Guanosine:PMA_6AN_15m vs. Guanosine:PMA_DPI_15m	ns	0.8059
Guanosine:PMA_6AN_15m vs. Guanosine:PMA_AA_15m	ns	>0.9999
Guanosine:PMA_6AN_15m vs. Guanosine:PMA_IAA_15m	****	<0.0001
Guanosine:PMA_6AN_15m vs. Guanine:PMA_15m	ns	0.1167
Guanosine:PMA_6AN_15m vs. Guanine:PMA_2DG_15m	ns	0.9894
Guanosine:PMA_6AN_15m vs. Guanine:PMA_6AN_15m	ns	0.9977
Guanosine:PMA_6AN_15m vs. Guanine:PMA_DPI_15m	ns	0.3992
Guanosine:PMA_6AN_15m vs. Guanine:PMA_AA_15m	ns	>0.9999
Guanosine:PMA_6AN_15m vs. Guanine:PMA_IAA_15m	****	<0.0001
Guanosine:PMA_6AN_15m vs. Aspartic acid:PMA_15m	ns	0.2456
Guanosine:PMA_6AN_15m vs. Aspartic acid:PMA_2DG_15m	ns	0.9994
Guanosine:PMA_6AN_15m vs. Aspartic acid:PMA_6AN_15m	ns	>0.9999
Guanosine:PMA_6AN_15m vs. Aspartic acid:PMA_DPI_15m	ns	0.6335
Guanosine:PMA_6AN_15m vs. Aspartic acid:PMA_AA_15m	ns	>0.9999
Guanosine:PMA_6AN_15m vs. Aspartic acid:PMA_IAA_15m	****	<0.0001
Guanosine:PMA_6AN_15m vs. AMP:PMA_15m	ns	0.9975
Guanosine:PMA_6AN_15m vs. AMP:PMA_2DG_15m	ns	>0.9999

Guanosine:PMA_6AN_15m vs. AMP:PMA_6AN_15m	ns	>0.9999
Guanosine:PMA_6AN_15m vs. AMP:PMA_DPI_15m	ns	>0.9999
Guanosine:PMA_6AN_15m vs. AMP:PMA_AA_15m	ns	>0.9999
Guanosine:PMA_6AN_15m vs. AMP:PMA_IAA_15m	*	0.02
Guanosine:PMA_6AN_15m vs. ADP:PMA_15m	ns	0.9563
Guanosine:PMA_6AN_15m vs. ADP:PMA_2DG_15m	ns	>0.9999
Guanosine:PMA_6AN_15m vs. ADP:PMA_6AN_15m	ns	>0.9999
Guanosine:PMA_6AN_15m vs. ADP:PMA_DPI_15m	ns	0.9995
Guanosine:PMA_6AN_15m vs. ADP:PMA_AA_15m	ns	>0.9999
Guanosine:PMA_6AN_15m vs. ADP:PMA_IAA_15m	**	0.0051
Guanosine:PMA_6AN_15m vs. ATP:PMA_15m	**	0.0012
Guanosine:PMA_6AN_15m vs. ATP:PMA_2DG_15m	ns	0.2383
Guanosine:PMA_6AN_15m vs. ATP:PMA_6AN_15m	ns	0.2322
Guanosine:PMA_6AN_15m vs. ATP:PMA_DPI_15m	**	0.0088
Guanosine:PMA_6AN_15m vs. ATP:PMA_AA_15m	ns	0.7071
Guanosine:PMA_6AN_15m vs. ATP:PMA_IAA_15m	****	<0.0001
Guanosine:PMA_6AN_15m vs. Xanthine:PMA_15m	ns	0.1109
Guanosine:PMA_6AN_15m vs. Xanthine:PMA_2DG_15m	ns	0.981
Guanosine:PMA_6AN_15m vs. Xanthine:PMA_6AN_15m	ns	0.9951
Guanosine:PMA_6AN_15m vs. Xanthine:PMA_DPI_15m	ns	0.3709
Guanosine:PMA_6AN_15m vs. Xanthine:PMA_AA_15m	ns	>0.9999
Guanosine:PMA_6AN_15m vs. CMP:PMA_15m	ns	0.9994
Guanosine:PMA_6AN_15m vs. CMP:PMA_2DG_15m	ns	>0.9999
Guanosine:PMA_6AN_15m vs. CMP:PMA_6AN_15m	ns	>0.9999
Guanosine:PMA_6AN_15m vs. CMP:PMA_DPI_15m	ns	>0.9999
Guanosine:PMA_6AN_15m vs. CMP:PMA_AA_15m	ns	>0.9999
Guanosine:PMA_6AN_15m vs. CMP:PMA_IAA_15m	*	0.0317
Guanosine:PMA_6AN_15m vs. CDP:PMA_15m	ns	>0.9999
Guanosine:PMA_6AN_15m vs. CDP:PMA_2DG_15m	ns	>0.9999
Guanosine:PMA_6AN_15m vs. CDP:PMA_6AN_15m	ns	>0.9999
Guanosine:PMA_6AN_15m vs. CDP:PMA_DPI_15m	ns	>0.9999
Guanosine:PMA_6AN_15m vs. CDP:PMA_AA_15m	ns	>0.9999
Guanosine:PMA_6AN_15m vs. CDP:PMA_IAA_15m	ns	0.183
Guanosine:PMA_6AN_15m vs. CTP:PMA_15m	*	0.0155
Guanosine:PMA_6AN_15m vs. CTP:PMA_2DG_15m	ns	0.7455
Guanosine:PMA_6AN_15m vs. CTP:PMA_6AN_15m	ns	0.8038
Guanosine:PMA_6AN_15m vs. CTP:PMA_DPI_15m	ns	0.0848
Guanosine:PMA_6AN_15m vs. CTP:PMA_AA_15m	ns	0.9889
Guanosine:PMA_6AN_15m vs. CTP:PMA_IAA_15m	****	<0.0001
Guanosine:PMA_6AN_15m vs. Uridine:PMA_15m	ns	>0.9999
Guanosine:PMA_6AN_15m vs. Uridine:PMA_2DG_15m	ns	>0.9999
Guanosine:PMA_6AN_15m vs. Uridine:PMA_6AN_15m	ns	>0.9999
Guanosine:PMA_6AN_15m vs. Uridine:PMA_DPI_15m	ns	>0.9999
Guanosine:PMA_6AN_15m vs. Uridine:PMA_AA_15m	ns	>0.9999
Guanosine:PMA_6AN_15m vs. Uridine:PMA_IAA_15m	ns	0.0625
Guanosine:PMA_6AN_15m vs. UMP:PMA_15m	ns	>0.9999
Guanosine:PMA_6AN_15m vs. UMP:PMA_2DG_15m	ns	>0.9999

Guanosine:PMA_6AN_15m vs. UMP:PMA_6AN_15m	ns	>0.9999
Guanosine:PMA_6AN_15m vs. UMP:PMA_DPI_15m	ns	>0.9999
Guanosine:PMA_6AN_15m vs. UMP:PMA_AA_15m	ns	>0.9999
Guanosine:PMA_6AN_15m vs. UMP:PMA_IAA_15m	ns	0.4439
Guanosine:PMA_6AN_15m vs. UDP:PMA_15m	ns	>0.9999
Guanosine:PMA_6AN_15m vs. UDP:PMA_2DG_15m	ns	>0.9999
Guanosine:PMA_6AN_15m vs. UDP:PMA_6AN_15m	ns	>0.9999
Guanosine:PMA_6AN_15m vs. UDP:PMA_DPI_15m	ns	>0.9999
Guanosine:PMA_6AN_15m vs. UDP:PMA_AA_15m	ns	>0.9999
Guanosine:PMA_6AN_15m vs. UDP:PMA_IAA_15m	ns	0.4277
Guanosine:PMA_6AN_15m vs. UTP:PMA_15m	**	0.0073
Guanosine:PMA_6AN_15m vs. UTP:PMA_2DG_15m	ns	0.5821
Guanosine:PMA_6AN_15m vs. UTP:PMA_6AN_15m	ns	0.6273
Guanosine:PMA_6AN_15m vs. UTP:PMA_DPI_15m	*	0.0449
Guanosine:PMA_6AN_15m vs. UTP:PMA_AA_15m	ns	0.9558
Guanosine:PMA_6AN_15m vs. UTP:PMA_IAA_15m	****	<0.0001
Guanosine:PMA_DPI_15m vs. Guanosine:PMA_AA_15m	ns	0.5731
Guanosine:PMA_DPI_15m vs. Guanosine:PMA_IAA_15m	***	0.0004
Guanosine:PMA_DPI_15m vs. Guanine:PMA_15m	ns	0.9856
Guanosine:PMA_DPI_15m vs. Guanine:PMA_2DG_15m	ns	>0.9999
Guanosine:PMA_DPI_15m vs. Guanine:PMA_6AN_15m	ns	>0.9999
Guanosine:PMA_DPI_15m vs. Guanine:PMA_DPI_15m	ns	0.9977
Guanosine:PMA_DPI_15m vs. Guanine:PMA_AA_15m	ns	>0.9999
Guanosine:PMA_DPI_15m vs. Guanine:PMA_IAA_15m	**	0.0097
Guanosine:PMA_DPI_15m vs. Aspartic acid:PMA_15m	ns	0.999
Guanosine:PMA_DPI_15m vs. Aspartic acid:PMA_2DG_15m	ns	>0.9999
Guanosine:PMA_DPI_15m vs. Aspartic acid:PMA_6AN_15m	ns	>0.9999
Guanosine:PMA_DPI_15m vs. Aspartic acid:PMA_DPI_15m	ns	>0.9999
Guanosine:PMA_DPI_15m vs. Aspartic acid:PMA_AA_15m	ns	>0.9999
Guanosine:PMA_DPI_15m vs. Aspartic acid:PMA_IAA_15m	*	0.0268
Guanosine:PMA_DPI_15m vs. AMP:PMA_15m	ns	>0.9999
Guanosine:PMA_DPI_15m vs. AMP:PMA_2DG_15m	ns	>0.9999
Guanosine:PMA_DPI_15m vs. AMP:PMA_6AN_15m	ns	>0.9999
Guanosine:PMA_DPI_15m vs. AMP:PMA_DPI_15m	ns	>0.9999
Guanosine:PMA_DPI_15m vs. AMP:PMA_AA_15m	ns	>0.9999
Guanosine:PMA_DPI_15m vs. AMP:PMA_IAA_15m	ns	0.7642
Guanosine:PMA_DPI_15m vs. ADP:PMA_15m	ns	>0.9999
Guanosine:PMA_DPI_15m vs. ADP:PMA_2DG_15m	ns	>0.9999
Guanosine:PMA_DPI_15m vs. ADP:PMA_6AN_15m	ns	>0.9999
Guanosine:PMA_DPI_15m vs. ADP:PMA_DPI_15m	ns	>0.9999
Guanosine:PMA_DPI_15m vs. ADP:PMA_AA_15m	ns	>0.9999
Guanosine:PMA_DPI_15m vs. ADP:PMA_IAA_15m	ns	0.4674
Guanosine:PMA_DPI_15m vs. ATP:PMA_15m	ns	0.2164
Guanosine:PMA_DPI_15m vs. ATP:PMA_2DG_15m	ns	0.9989
Guanosine:PMA_DPI_15m vs. ATP:PMA_6AN_15m	ns	>0.9999
Guanosine:PMA_DPI_15m vs. ATP:PMA_DPI_15m	ns	0.2322
Guanosine:PMA_DPI_15m vs. ATP:PMA_AA_15m	ns	>0.9999

Guanosine:PMA_DPI_15m vs. ATP:PMA_IAA_15m	****	<0.0001
Guanosine:PMA_DPI_15m vs. Xanthine:PMA_15m	ns	0.9752
Guanosine:PMA_DPI_15m vs. Xanthine:PMA_2DG_15m	ns	>0.9999
Guanosine:PMA_DPI_15m vs. Xanthine:PMA_6AN_15m	ns	>0.9999
Guanosine:PMA_DPI_15m vs. Xanthine:PMA_DPI_15m	ns	0.9951
Guanosine:PMA_DPI_15m vs. Xanthine:PMA_AA_15m	ns	>0.9999
Guanosine:PMA_DPI_15m vs. CMP:PMA_15m	ns	>0.9999
Guanosine:PMA_DPI_15m vs. CMP:PMA_2DG_15m	ns	>0.9999
Guanosine:PMA_DPI_15m vs. CMP:PMA_6AN_15m	ns	>0.9999
Guanosine:PMA_DPI_15m vs. CMP:PMA_DPI_15m	ns	>0.9999
Guanosine:PMA_DPI_15m vs. CMP:PMA_AA_15m	ns	>0.9999
Guanosine:PMA_DPI_15m vs. CMP:PMA_IAA_15m	ns	0.8512
Guanosine:PMA_DPI_15m vs. CDP:PMA_15m	ns	>0.9999
Guanosine:PMA_DPI_15m vs. CDP:PMA_2DG_15m	ns	>0.9999
Guanosine:PMA_DPI_15m vs. CDP:PMA_6AN_15m	ns	>0.9999
Guanosine:PMA_DPI_15m vs. CDP:PMA_DPI_15m	ns	>0.9999
Guanosine:PMA_DPI_15m vs. CDP:PMA_AA_15m	ns	>0.9999
Guanosine:PMA_DPI_15m vs. CDP:PMA_IAA_15m	ns	0.9963
Guanosine:PMA_DPI_15m vs. CTP:PMA_15m	ns	0.7152
Guanosine:PMA_DPI_15m vs. CTP:PMA_2DG_15m	ns	>0.9999
Guanosine:PMA_DPI_15m vs. CTP:PMA_6AN_15m	ns	>0.9999
Guanosine:PMA_DPI_15m vs. CTP:PMA_DPI_15m	ns	0.8038
Guanosine:PMA_DPI_15m vs. CTP:PMA_AA_15m	ns	>0.9999
Guanosine:PMA_DPI_15m vs. CTP:PMA_IAA_15m	***	0.0008
Guanosine:PMA_DPI_15m vs. Uridine:PMA_15m	ns	>0.9999
Guanosine:PMA_DPI_15m vs. Uridine:PMA_2DG_15m	ns	>0.9999
Guanosine:PMA_DPI_15m vs. Uridine:PMA_6AN_15m	ns	>0.9999
Guanosine:PMA_DPI_15m vs. Uridine:PMA_DPI_15m	ns	>0.9999
Guanosine:PMA_DPI_15m vs. Uridine:PMA_AA_15m	ns	>0.9999
Guanosine:PMA_DPI_15m vs. Uridine:PMA_IAA_15m	ns	0.9428
Guanosine:PMA_DPI_15m vs. UMP:PMA_15m	ns	>0.9999
Guanosine:PMA_DPI_15m vs. UMP:PMA_2DG_15m	ns	>0.9999
Guanosine:PMA_DPI_15m vs. UMP:PMA_6AN_15m	ns	0.9999
Guanosine:PMA_DPI_15m vs. UMP:PMA_DPI_15m	ns	>0.9999
Guanosine:PMA_DPI_15m vs. UMP:PMA_AA_15m	ns	0.9993
Guanosine:PMA_DPI_15m vs. UMP:PMA_IAA_15m	ns	>0.9999
Guanosine:PMA_DPI_15m vs. UDP:PMA_15m	ns	>0.9999
Guanosine:PMA_DPI_15m vs. UDP:PMA_2DG_15m	ns	>0.9999
Guanosine:PMA_DPI_15m vs. UDP:PMA_6AN_15m	ns	>0.9999
Guanosine:PMA_DPI_15m vs. UDP:PMA_DPI_15m	ns	>0.9999
Guanosine:PMA_DPI_15m vs. UDP:PMA_AA_15m	ns	0.9995
Guanosine:PMA_DPI_15m vs. UDP:PMA_IAA_15m	ns	>0.9999
Guanosine:PMA_DPI_15m vs. UTP:PMA_15m	ns	0.5486
Guanosine:PMA_DPI_15m vs. UTP:PMA_2DG_15m	ns	>0.9999
Guanosine:PMA_DPI_15m vs. UTP:PMA_6AN_15m	ns	>0.9999
Guanosine:PMA_DPI_15m vs. UTP:PMA_DPI_15m	ns	0.6273
Guanosine:PMA_DPI_15m vs. UTP:PMA_AA_15m	ns	>0.9999

Guanosine:PMA_DPI_15m vs. UTP:PMA_IAA_15m	***	0.0004
Guanosine:PMA_AA_15m vs. Guanosine:PMA_IAA_15m	****	<0.0001
Guanosine:PMA_AA_15m vs. Guanine:PMA_15m	ns	0.0764
Guanosine:PMA_AA_15m vs. Guanine:PMA_2DG_15m	ns	0.9702
Guanosine:PMA_AA_15m vs. Guanine:PMA_6AN_15m	ns	0.9996
Guanosine:PMA_AA_15m vs. Guanine:PMA_DPI_15m	ns	0.2966
Guanosine:PMA_AA_15m vs. Guanine:PMA_AA_15m	ns	0.9977
Guanosine:PMA_AA_15m vs. Guanine:PMA_IAA_15m	****	<0.0001
Guanosine:PMA_AA_15m vs. Aspartic acid:PMA_15m	ns	0.1718
Guanosine:PMA_AA_15m vs. Aspartic acid:PMA_2DG_15m	ns	0.997
Guanosine:PMA_AA_15m vs. Aspartic acid:PMA_6AN_15m	ns	>0.9999
Guanosine:PMA_AA_15m vs. Aspartic acid:PMA_DPI_15m	ns	0.5132
Guanosine:PMA_AA_15m vs. Aspartic acid:PMA_AA_15m	ns	>0.9999
Guanosine:PMA_AA_15m vs. Aspartic acid:PMA_IAA_15m	****	<0.0001
Guanosine:PMA_AA_15m vs. AMP:PMA_15m	ns	0.9908
Guanosine:PMA_AA_15m vs. AMP:PMA_2DG_15m	ns	>0.9999
Guanosine:PMA_AA_15m vs. AMP:PMA_6AN_15m	ns	>0.9999
Guanosine:PMA_AA_15m vs. AMP:PMA_DPI_15m	ns	>0.9999
Guanosine:PMA_AA_15m vs. AMP:PMA_AA_15m	ns	>0.9999
Guanosine:PMA_AA_15m vs. AMP:PMA_IAA_15m	*	0.0119
Guanosine:PMA_AA_15m vs. ADP:PMA_15m	ns	0.9075
Guanosine:PMA_AA_15m vs. ADP:PMA_2DG_15m	ns	>0.9999
Guanosine:PMA_AA_15m vs. ADP:PMA_6AN_15m	ns	>0.9999
Guanosine:PMA_AA_15m vs. ADP:PMA_DPI_15m	ns	0.9975
Guanosine:PMA_AA_15m vs. ADP:PMA_AA_15m	ns	>0.9999
Guanosine:PMA_AA_15m vs. ADP:PMA_IAA_15m	**	0.0029
Guanosine:PMA_AA_15m vs. ATP:PMA_15m	***	0.0006
Guanosine:PMA_AA_15m vs. ATP:PMA_2DG_15m	ns	0.1661
Guanosine:PMA_AA_15m vs. ATP:PMA_6AN_15m	ns	0.4698
Guanosine:PMA_AA_15m vs. ATP:PMA_DPI_15m	**	0.0051
Guanosine:PMA_AA_15m vs. ATP:PMA_AA_15m	ns	0.2322
Guanosine:PMA_AA_15m vs. ATP:PMA_IAA_15m	****	<0.0001
Guanosine:PMA_AA_15m vs. Xanthine:PMA_15m	ns	0.0736
Guanosine:PMA_AA_15m vs. Xanthine:PMA_2DG_15m	ns	0.9538
Guanosine:PMA_AA_15m vs. Xanthine:PMA_6AN_15m	ns	0.9989
Guanosine:PMA_AA_15m vs. Xanthine:PMA_DPI_15m	ns	0.2761
Guanosine:PMA_AA_15m vs. Xanthine:PMA_AA_15m	ns	0.9951
Guanosine:PMA_AA_15m vs. CMP:PMA_15m	ns	0.9973
Guanosine:PMA_AA_15m vs. CMP:PMA_2DG_15m	ns	>0.9999
Guanosine:PMA_AA_15m vs. CMP:PMA_6AN_15m	ns	>0.9999
Guanosine:PMA_AA_15m vs. CMP:PMA_DPI_15m	ns	>0.9999
Guanosine:PMA_AA_15m vs. CMP:PMA_AA_15m	ns	>0.9999
Guanosine:PMA_AA_15m vs. CMP:PMA_IAA_15m	*	0.0193
Guanosine:PMA_AA_15m vs. CDP:PMA_15m	ns	>0.9999
Guanosine:PMA_AA_15m vs. CDP:PMA_2DG_15m	ns	>0.9999
Guanosine:PMA_AA_15m vs. CDP:PMA_6AN_15m	ns	>0.9999
Guanosine:PMA_AA_15m vs. CDP:PMA_DPI_15m	ns	>0.9999

Guanosine:PMA_AA_15m vs. CDP:PMA_AA_15m	ns	>0.9999
Guanosine:PMA_AA_15m vs. CDP:PMA_IAA_15m	ns	0.1245
Guanosine:PMA_AA_15m vs. CTP:PMA_15m	**	0.0092
Guanosine:PMA_AA_15m vs. CTP:PMA_2DG_15m	ns	0.6314
Guanosine:PMA_AA_15m vs. CTP:PMA_6AN_15m	ns	0.9299
Guanosine:PMA_AA_15m vs. CTP:PMA_DPI_15m	ns	0.0543
Guanosine:PMA_AA_15m vs. CTP:PMA_AA_15m	ns	0.8038
Guanosine:PMA_AA_15m vs. CTP:PMA_IAA_15m	****	<0.0001
Guanosine:PMA_AA_15m vs. Uridine:PMA_15m	ns	0.9998
Guanosine:PMA_AA_15m vs. Uridine:PMA_2DG_15m	ns	>0.9999
Guanosine:PMA_AA_15m vs. Uridine:PMA_6AN_15m	ns	>0.9999
Guanosine:PMA_AA_15m vs. Uridine:PMA_DPI_15m	ns	>0.9999
Guanosine:PMA_AA_15m vs. Uridine:PMA_AA_15m	ns	>0.9999
Guanosine:PMA_AA_15m vs. Uridine:PMA_IAA_15m	*	0.0394
Guanosine:PMA_AA_15m vs. UMP:PMA_15m	ns	>0.9999
Guanosine:PMA_AA_15m vs. UMP:PMA_2DG_15m	ns	>0.9999
Guanosine:PMA_AA_15m vs. UMP:PMA_6AN_15m	ns	>0.9999
Guanosine:PMA_AA_15m vs. UMP:PMA_DPI_15m	ns	>0.9999
Guanosine:PMA_AA_15m vs. UMP:PMA_AA_15m	ns	>0.9999
Guanosine:PMA_AA_15m vs. UMP:PMA_IAA_15m	ns	0.3357
Guanosine:PMA_AA_15m vs. UDP:PMA_15m	ns	>0.9999
Guanosine:PMA_AA_15m vs. UDP:PMA_2DG_15m	ns	>0.9999
Guanosine:PMA_AA_15m vs. UDP:PMA_6AN_15m	ns	>0.9999
Guanosine:PMA_AA_15m vs. UDP:PMA_DPI_15m	ns	>0.9999
Guanosine:PMA_AA_15m vs. UDP:PMA_AA_15m	ns	>0.9999
Guanosine:PMA_AA_15m vs. UDP:PMA_IAA_15m	ns	0.3216
Guanosine:PMA_AA_15m vs. UTP:PMA_15m	**	0.0042
Guanosine:PMA_AA_15m vs. UTP:PMA_2DG_15m	ns	0.4626
Guanosine:PMA_AA_15m vs. UTP:PMA_6AN_15m	ns	0.8311
Guanosine:PMA_AA_15m vs. UTP:PMA_DPI_15m	*	0.0278
Guanosine:PMA_AA_15m vs. UTP:PMA_AA_15m	ns	0.6273
Guanosine:PMA_AA_15m vs. UTP:PMA_IAA_15m	****	<0.0001
Guanosine:PMA_IAA_15m vs. Guanine:PMA_15m	ns	>0.9999
Guanosine:PMA_IAA_15m vs. Guanine:PMA_2DG_15m	ns	>0.9999
Guanosine:PMA_IAA_15m vs. Guanine:PMA_6AN_15m	ns	>0.9999
Guanosine:PMA_IAA_15m vs. Guanine:PMA_DPI_15m	ns	>0.9999
Guanosine:PMA_IAA_15m vs. Guanine:PMA_AA_15m	ns	0.9996
Guanosine:PMA_IAA_15m vs. Guanine:PMA_IAA_15m	ns	0.9977
Guanosine:PMA_IAA_15m vs. Aspartic acid:PMA_15m	ns	>0.9999
Guanosine:PMA_IAA_15m vs. Aspartic acid:PMA_2DG_15m	ns	>0.9999
Guanosine:PMA_IAA_15m vs. Aspartic acid:PMA_6AN_15m	ns	0.9981
Guanosine:PMA_IAA_15m vs. Aspartic acid:PMA_DPI_15m	ns	>0.9999
Guanosine:PMA_IAA_15m vs. Aspartic acid:PMA_AA_15m	ns	0.9927
Guanosine:PMA_IAA_15m vs. Aspartic acid:PMA_IAA_15m	ns	>0.9999
Guanosine:PMA_IAA_15m vs. AMP:PMA_15m	ns	>0.9999
Guanosine:PMA_IAA_15m vs. AMP:PMA_2DG_15m	ns	0.6255
Guanosine:PMA_IAA_15m vs. AMP:PMA_6AN_15m	ns	0.2647

Guanosine:PMA_IAA_15m vs. AMP:PMA_DPI_15m	ns	0.9993
Guanosine:PMA_IAA_15m vs. AMP:PMA_AA_15m	ns	0.1868
Guanosine:PMA_IAA_15m vs. AMP:PMA_IAA_15m	ns	>0.9999
Guanosine:PMA_IAA_15m vs. ADP:PMA_15m	ns	>0.9999
Guanosine:PMA_IAA_15m vs. ADP:PMA_2DG_15m	ns	0.8814
Guanosine:PMA_IAA_15m vs. ADP:PMA_6AN_15m	ns	0.5384
Guanosine:PMA_IAA_15m vs. ADP:PMA_DPI_15m	ns	>0.9999
Guanosine:PMA_IAA_15m vs. ADP:PMA_AA_15m	ns	0.4214
Guanosine:PMA_IAA_15m vs. ADP:PMA_IAA_15m	ns	>0.9999
Guanosine:PMA_IAA_15m vs. ATP:PMA_15m	ns	>0.9999
Guanosine:PMA_IAA_15m vs. ATP:PMA_2DG_15m	ns	>0.9999
Guanosine:PMA_IAA_15m vs. ATP:PMA_6AN_15m	ns	>0.9999
Guanosine:PMA_IAA_15m vs. ATP:PMA_DPI_15m	ns	>0.9999
Guanosine:PMA_IAA_15m vs. ATP:PMA_AA_15m	ns	>0.9999
Guanosine:PMA_IAA_15m vs. ATP:PMA_IAA_15m	ns	0.2322
Guanosine:PMA_IAA_15m vs. Xanthine:PMA_15m	ns	>0.9999
Guanosine:PMA_IAA_15m vs. Xanthine:PMA_2DG_15m	ns	>0.9999
Guanosine:PMA_IAA_15m vs. Xanthine:PMA_6AN_15m	ns	>0.9999
Guanosine:PMA_IAA_15m vs. Xanthine:PMA_DPI_15m	ns	>0.9999
Guanosine:PMA_IAA_15m vs. Xanthine:PMA_AA_15m	ns	>0.9999
Guanosine:PMA_IAA_15m vs. CMP:PMA_15m	ns	>0.9999
Guanosine:PMA_IAA_15m vs. CMP:PMA_2DG_15m	ns	0.5136
Guanosine:PMA_IAA_15m vs. CMP:PMA_6AN_15m	ns	0.1915
Guanosine:PMA_IAA_15m vs. CMP:PMA_DPI_15m	ns	0.9969
Guanosine:PMA_IAA_15m vs. CMP:PMA_AA_15m	ns	0.1308
Guanosine:PMA_IAA_15m vs. CMP:PMA_IAA_15m	ns	>0.9999
Guanosine:PMA_IAA_15m vs. CDP:PMA_15m	ns	0.9939
Guanosine:PMA_IAA_15m vs. CDP:PMA_2DG_15m	ns	0.1423
Guanosine:PMA_IAA_15m vs. CDP:PMA_6AN_15m	*	0.0337
Guanosine:PMA_IAA_15m vs. CDP:PMA_DPI_15m	ns	0.8612
Guanosine:PMA_IAA_15m vs. CDP:PMA_AA_15m	*	0.0206
Guanosine:PMA_IAA_15m vs. CDP:PMA_IAA_15m	ns	>0.9999
Guanosine:PMA_IAA_15m vs. CTP:PMA_15m	ns	>0.9999
Guanosine:PMA_IAA_15m vs. CTP:PMA_2DG_15m	ns	>0.9999
Guanosine:PMA_IAA_15m vs. CTP:PMA_6AN_15m	ns	>0.9999
Guanosine:PMA_IAA_15m vs. CTP:PMA_DPI_15m	ns	>0.9999
Guanosine:PMA_IAA_15m vs. CTP:PMA_AA_15m	ns	>0.9999
Guanosine:PMA_IAA_15m vs. CTP:PMA_IAA_15m	ns	0.8038
Guanosine:PMA_IAA_15m vs. Uridine:PMA_15m	ns	>0.9999
Guanosine:PMA_IAA_15m vs. Uridine:PMA_2DG_15m	ns	0.3504
Guanosine:PMA_IAA_15m vs. Uridine:PMA_6AN_15m	ns	0.109
Guanosine:PMA_IAA_15m vs. Uridine:PMA_DPI_15m	ns	0.9821
Guanosine:PMA_IAA_15m vs. Uridine:PMA_AA_15m	ns	0.0711
Guanosine:PMA_IAA_15m vs. Uridine:PMA_IAA_15m	ns	>0.9999
Guanosine:PMA_IAA_15m vs. UMP:PMA_15m	ns	0.9129
Guanosine:PMA_IAA_15m vs. UMP:PMA_2DG_15m	*	0.0422
Guanosine:PMA_IAA_15m vs. UMP:PMA_6AN_15m	**	0.008

Guanosine:PMA_IAA_15m vs. UMP:PMA_DPI_15m	ns	0.5628
Guanosine:PMA_IAA_15m vs. UMP:PMA_AA_15m	**	0.0046
Guanosine:PMA_IAA_15m vs. UMP:PMA_IAA_15m	ns	>0.9999
Guanosine:PMA_IAA_15m vs. UDP:PMA_15m	ns	0.921
Guanosine:PMA_IAA_15m vs. UDP:PMA_2DG_15m	*	0.0451
Guanosine:PMA_IAA_15m vs. UDP:PMA_6AN_15m	**	0.0086
Guanosine:PMA_IAA_15m vs. UDP:PMA_DPI_15m	ns	0.5798
Guanosine:PMA_IAA_15m vs. UDP:PMA_AA_15m	**	0.005
Guanosine:PMA_IAA_15m vs. UDP:PMA_IAA_15m	ns	>0.9999
Guanosine:PMA_IAA_15m vs. UTP:PMA_15m	ns	>0.9999
Guanosine:PMA_IAA_15m vs. UTP:PMA_2DG_15m	ns	>0.9999
Guanosine:PMA_IAA_15m vs. UTP:PMA_6AN_15m	ns	>0.9999
Guanosine:PMA_IAA_15m vs. UTP:PMA_DPI_15m	ns	>0.9999
Guanosine:PMA_IAA_15m vs. UTP:PMA_AA_15m	ns	>0.9999
Guanosine:PMA_IAA_15m vs. UTP:PMA_IAA_15m	ns	0.6273
Guanine:PMA_15m vs. Guanine:PMA_2DG_15m	ns	0.7475
Guanine:PMA_15m vs. Guanine:PMA_6AN_15m	ns	0.1037
Guanine:PMA_15m vs. Guanine:PMA_DPI_15m	ns	>0.9999
Guanine:PMA_15m vs. Guanine:PMA_AA_15m	*	0.0409
Guanine:PMA_15m vs. Guanine:PMA_IAA_15m	*	0.0236
Guanine:PMA_15m vs. Aspartic acid:PMA_15m	ns	>0.9999
Guanine:PMA_15m vs. Aspartic acid:PMA_2DG_15m	ns	>0.9999
Guanine:PMA_15m vs. Aspartic acid:PMA_6AN_15m	ns	0.9995
Guanine:PMA_15m vs. Aspartic acid:PMA_DPI_15m	ns	>0.9999
Guanine:PMA_15m vs. Aspartic acid:PMA_AA_15m	ns	0.9977
Guanine:PMA_15m vs. Aspartic acid:PMA_IAA_15m	ns	>0.9999
Guanine:PMA_15m vs. AMP:PMA_15m	ns	>0.9999
Guanine:PMA_15m vs. AMP:PMA_2DG_15m	ns	0.7218
Guanine:PMA_15m vs. AMP:PMA_6AN_15m	ns	0.3427
Guanine:PMA_15m vs. AMP:PMA_DPI_15m	ns	0.9999
Guanine:PMA_15m vs. AMP:PMA_AA_15m	ns	0.2492
Guanine:PMA_15m vs. AMP:PMA_IAA_15m	ns	>0.9999
Guanine:PMA_15m vs. ADP:PMA_15m	ns	>0.9999
Guanine:PMA_15m vs. ADP:PMA_2DG_15m	ns	0.9327
Guanine:PMA_15m vs. ADP:PMA_6AN_15m	ns	0.6382
Guanine:PMA_15m vs. ADP:PMA_DPI_15m	ns	>0.9999
Guanine:PMA_15m vs. ADP:PMA_AA_15m	ns	0.5179
Guanine:PMA_15m vs. ADP:PMA_IAA_15m	ns	>0.9999
Guanine:PMA_15m vs. ATP:PMA_15m	ns	>0.9999
Guanine:PMA_15m vs. ATP:PMA_2DG_15m	ns	>0.9999
Guanine:PMA_15m vs. ATP:PMA_6AN_15m	ns	>0.9999
Guanine:PMA_15m vs. ATP:PMA_DPI_15m	ns	>0.9999
Guanine:PMA_15m vs. ATP:PMA_AA_15m	ns	>0.9999
Guanine:PMA_15m vs. ATP:PMA_IAA_15m	ns	0.49
Guanine:PMA_15m vs. Xanthine:PMA_15m	ns	>0.9999
Guanine:PMA_15m vs. Xanthine:PMA_2DG_15m	ns	>0.9999
Guanine:PMA_15m vs. Xanthine:PMA_6AN_15m	ns	>0.9999

Guanine:PMA_15m vs. Xanthine:PMA_DPI_15m	ns	>0.9999
Guanine:PMA_15m vs. Xanthine:PMA_AA_15m	ns	>0.9999
Guanine:PMA_15m vs. CMP:PMA_15m	ns	>0.9999
Guanine:PMA_15m vs. CMP:PMA_2DG_15m	ns	0.6135
Guanine:PMA_15m vs. CMP:PMA_6AN_15m	ns	0.255
Guanine:PMA_15m vs. CMP:PMA_DPI_15m	ns	0.9992
Guanine:PMA_15m vs. CMP:PMA_AA_15m	ns	0.179
Guanine:PMA_15m vs. CMP:PMA_IAA_15m	ns	>0.9999
Guanine:PMA_15m vs. CDP:PMA_15m	ns	0.9683
Guanine:PMA_15m vs. CDP:PMA_2DG_15m	ns	0.1936
Guanine:PMA_15m vs. CDP:PMA_6AN_15m	*	0.0493
Guanine:PMA_15m vs. CDP:PMA_DPI_15m	ns	0.9184
Guanine:PMA_15m vs. CDP:PMA_AA_15m	*	0.0306
Guanine:PMA_15m vs. CDP:PMA_IAA_15m	ns	>0.9999
Guanine:PMA_15m vs. CTP:PMA_15m	ns	>0.9999
Guanine:PMA_15m vs. CTP:PMA_2DG_15m	ns	>0.9999
Guanine:PMA_15m vs. CTP:PMA_6AN_15m	ns	>0.9999
Guanine:PMA_15m vs. CTP:PMA_DPI_15m	ns	>0.9999
Guanine:PMA_15m vs. CTP:PMA_AA_15m	ns	>0.9999
Guanine:PMA_15m vs. CTP:PMA_IAA_15m	ns	0.9375
Guanine:PMA_15m vs. Uridine:PMA_15m	ns	0.9993
Guanine:PMA_15m vs. Uridine:PMA_2DG_15m	ns	0.4406
Guanine:PMA_15m vs. Uridine:PMA_6AN_15m	ns	0.1508
Guanine:PMA_15m vs. Uridine:PMA_DPI_15m	ns	0.9932
Guanine:PMA_15m vs. Uridine:PMA_AA_15m	ns	0.1008
Guanine:PMA_15m vs. Uridine:PMA_IAA_15m	ns	>0.9999
Guanine:PMA_15m vs. UMP:PMA_15m	ns	0.7504
Guanine:PMA_15m vs. UMP:PMA_2DG_15m	ns	0.0612
Guanine:PMA_15m vs. UMP:PMA_6AN_15m	*	0.0122
Guanine:PMA_15m vs. UMP:PMA_DPI_15m	ns	0.6621
Guanine:PMA_15m vs. UMP:PMA_AA_15m	**	0.0071
Guanine:PMA_15m vs. UMP:PMA_IAA_15m	ns	>0.9999
Guanine:PMA_15m vs. UDP:PMA_15m	ns	0.7674
Guanine:PMA_15m vs. UDP:PMA_2DG_15m	ns	0.0652
Guanine:PMA_15m vs. UDP:PMA_6AN_15m	*	0.0131
Guanine:PMA_15m vs. UDP:PMA_DPI_15m	ns	0.6786
Guanine:PMA_15m vs. UDP:PMA_AA_15m	**	0.0077
Guanine:PMA_15m vs. UDP:PMA_IAA_15m	ns	>0.9999
Guanine:PMA_15m vs. UTP:PMA_15m	ns	>0.9999
Guanine:PMA_15m vs. UTP:PMA_2DG_15m	ns	>0.9999
Guanine:PMA_15m vs. UTP:PMA_6AN_15m	ns	>0.9999
Guanine:PMA_15m vs. UTP:PMA_DPI_15m	ns	>0.9999
Guanine:PMA_15m vs. UTP:PMA_AA_15m	ns	>0.9999
Guanine:PMA_15m vs. UTP:PMA_IAA_15m	ns	0.845
Guanine:PMA_2DG_15m vs. Guanine:PMA_6AN_15m	ns	>0.9999
Guanine:PMA_2DG_15m vs. Guanine:PMA_DPI_15m	ns	>0.9999
Guanine:PMA_2DG_15m vs. Guanine:PMA_AA_15m	ns	>0.9999

Guanine:PMA_2DG_15m vs. Guanine:PMA_IAA_15m	****	<0.0001
Guanine:PMA_2DG_15m vs. Aspartic acid:PMA_15m	ns	>0.9999
Guanine:PMA_2DG_15m vs. Aspartic acid:PMA_2DG_15m	ns	>0.9999
Guanine:PMA_2DG_15m vs. Aspartic acid:PMA_6AN_15m	ns	>0.9999
Guanine:PMA_2DG_15m vs. Aspartic acid:PMA_DPI_15m	ns	>0.9999
Guanine:PMA_2DG_15m vs. Aspartic acid:PMA_AA_15m	ns	>0.9999
Guanine:PMA_2DG_15m vs. Aspartic acid:PMA_IAA_15m	ns	0.5005
Guanine:PMA_2DG_15m vs. AMP:PMA_15m	ns	>0.9999
Guanine:PMA_2DG_15m vs. AMP:PMA_2DG_15m	ns	>0.9999
Guanine:PMA_2DG_15m vs. AMP:PMA_6AN_15m	ns	>0.9999
Guanine:PMA_2DG_15m vs. AMP:PMA_DPI_15m	ns	>0.9999
Guanine:PMA_2DG_15m vs. AMP:PMA_AA_15m	ns	0.9994
Guanine:PMA_2DG_15m vs. AMP:PMA_IAA_15m	ns	>0.9999
Guanine:PMA_2DG_15m vs. ADP:PMA_15m	ns	>0.9999
Guanine:PMA_2DG_15m vs. ADP:PMA_2DG_15m	ns	>0.9999
Guanine:PMA_2DG_15m vs. ADP:PMA_6AN_15m	ns	>0.9999
Guanine:PMA_2DG_15m vs. ADP:PMA_DPI_15m	ns	>0.9999
Guanine:PMA_2DG_15m vs. ADP:PMA_AA_15m	ns	>0.9999
Guanine:PMA_2DG_15m vs. ADP:PMA_IAA_15m	ns	0.997
Guanine:PMA_2DG_15m vs. ATP:PMA_15m	ns	0.9531
Guanine:PMA_2DG_15m vs. ATP:PMA_2DG_15m	ns	>0.9999
Guanine:PMA_2DG_15m vs. ATP:PMA_6AN_15m	ns	>0.9999
Guanine:PMA_2DG_15m vs. ATP:PMA_DPI_15m	ns	0.9994
Guanine:PMA_2DG_15m vs. ATP:PMA_AA_15m	ns	>0.9999
Guanine:PMA_2DG_15m vs. ATP:PMA_IAA_15m	**	0.0049
Guanine:PMA_2DG_15m vs. Xanthine:PMA_15m	ns	>0.9999
Guanine:PMA_2DG_15m vs. Xanthine:PMA_2DG_15m	ns	>0.9999
Guanine:PMA_2DG_15m vs. Xanthine:PMA_6AN_15m	ns	>0.9999
Guanine:PMA_2DG_15m vs. Xanthine:PMA_DPI_15m	ns	>0.9999
Guanine:PMA_2DG_15m vs. Xanthine:PMA_AA_15m	ns	>0.9999
Guanine:PMA_2DG_15m vs. CMP:PMA_15m	ns	>0.9999
Guanine:PMA_2DG_15m vs. CMP:PMA_2DG_15m	ns	>0.9999
Guanine:PMA_2DG_15m vs. CMP:PMA_6AN_15m	ns	0.9995
Guanine:PMA_2DG_15m vs. CMP:PMA_DPI_15m	ns	>0.9999
Guanine:PMA_2DG_15m vs. CMP:PMA_AA_15m	ns	0.9974
Guanine:PMA_2DG_15m vs. CMP:PMA_IAA_15m	ns	>0.9999
Guanine:PMA_2DG_15m vs. CDP:PMA_15m	ns	>0.9999
Guanine:PMA_2DG_15m vs. CDP:PMA_2DG_15m	ns	0.9683
Guanine:PMA_2DG_15m vs. CDP:PMA_6AN_15m	ns	0.9332
Guanine:PMA_2DG_15m vs. CDP:PMA_DPI_15m	ns	>0.9999
Guanine:PMA_2DG_15m vs. CDP:PMA_AA_15m	ns	0.8704
Guanine:PMA_2DG_15m vs. CDP:PMA_IAA_15m	ns	>0.9999
Guanine:PMA_2DG_15m vs. CTP:PMA_15m	ns	>0.9999
Guanine:PMA_2DG_15m vs. CTP:PMA_2DG_15m	ns	>0.9999
Guanine:PMA_2DG_15m vs. CTP:PMA_6AN_15m	ns	>0.9999
Guanine:PMA_2DG_15m vs. CTP:PMA_DPI_15m	ns	>0.9999
Guanine:PMA_2DG_15m vs. CTP:PMA_AA_15m	ns	>0.9999

Guanine:PMA_2DG_15m vs. CTP:PMA_IAA_15m	ns	0.0523
Guanine:PMA_2DG_15m vs. Uridine:PMA_15m	ns	>0.9999
Guanine:PMA_2DG_15m vs. Uridine:PMA_2DG_15m	ns	0.9993
Guanine:PMA_2DG_15m vs. Uridine:PMA_6AN_15m	ns	0.9952
Guanine:PMA_2DG_15m vs. Uridine:PMA_DPI_15m	ns	>0.9999
Guanine:PMA_2DG_15m vs. Uridine:PMA_AA_15m	ns	0.9843
Guanine:PMA_2DG_15m vs. Uridine:PMA_IAA_15m	ns	>0.9999
Guanine:PMA_2DG_15m vs. UMP:PMA_15m	ns	>0.9999
Guanine:PMA_2DG_15m vs. UMP:PMA_2DG_15m	ns	0.7504
Guanine:PMA_2DG_15m vs. UMP:PMA_6AN_15m	ns	0.6942
Guanine:PMA_2DG_15m vs. UMP:PMA_DPI_15m	ns	>0.9999
Guanine:PMA_2DG_15m vs. UMP:PMA_AA_15m	ns	0.5758
Guanine:PMA_2DG_15m vs. UMP:PMA_IAA_15m	ns	>0.9999
Guanine:PMA_2DG_15m vs. UDP:PMA_15m	ns	>0.9999
Guanine:PMA_2DG_15m vs. UDP:PMA_2DG_15m	ns	0.7674
Guanine:PMA_2DG_15m vs. UDP:PMA_6AN_15m	ns	0.7103
Guanine:PMA_2DG_15m vs. UDP:PMA_DPI_15m	ns	>0.9999
Guanine:PMA_2DG_15m vs. UDP:PMA_AA_15m	ns	0.5929
Guanine:PMA_2DG_15m vs. UDP:PMA_IAA_15m	ns	>0.9999
Guanine:PMA_2DG_15m vs. UTP:PMA_15m	ns	0.999
Guanine:PMA_2DG_15m vs. UTP:PMA_2DG_15m	ns	>0.9999
Guanine:PMA_2DG_15m vs. UTP:PMA_6AN_15m	ns	>0.9999
Guanine:PMA_2DG_15m vs. UTP:PMA_DPI_15m	ns	>0.9999
Guanine:PMA_2DG_15m vs. UTP:PMA_AA_15m	ns	>0.9999
Guanine:PMA_2DG_15m vs. UTP:PMA_IAA_15m	*	0.0267
Guanine:PMA_6AN_15m vs. Guanine:PMA_DPI_15m	ns	0.8059
Guanine:PMA_6AN_15m vs. Guanine:PMA_AA_15m	ns	>0.9999
Guanine:PMA_6AN_15m vs. Guanine:PMA_IAA_15m	****	<0.0001
Guanine:PMA_6AN_15m vs. Aspartic acid:PMA_15m	ns	>0.9999
Guanine:PMA_6AN_15m vs. Aspartic acid:PMA_2DG_15m	ns	>0.9999
Guanine:PMA_6AN_15m vs. Aspartic acid:PMA_6AN_15m	ns	>0.9999
Guanine:PMA_6AN_15m vs. Aspartic acid:PMA_DPI_15m	ns	>0.9999
Guanine:PMA_6AN_15m vs. Aspartic acid:PMA_AA_15m	ns	>0.9999
Guanine:PMA_6AN_15m vs. Aspartic acid:PMA_IAA_15m	ns	0.1839
Guanine:PMA_6AN_15m vs. AMP:PMA_15m	ns	>0.9999
Guanine:PMA_6AN_15m vs. AMP:PMA_2DG_15m	ns	>0.9999
Guanine:PMA_6AN_15m vs. AMP:PMA_6AN_15m	ns	>0.9999
Guanine:PMA_6AN_15m vs. AMP:PMA_DPI_15m	ns	>0.9999
Guanine:PMA_6AN_15m vs. AMP:PMA_AA_15m	ns	>0.9999
Guanine:PMA_6AN_15m vs. AMP:PMA_IAA_15m	ns	0.9923
Guanine:PMA_6AN_15m vs. ADP:PMA_15m	ns	>0.9999
Guanine:PMA_6AN_15m vs. ADP:PMA_2DG_15m	ns	>0.9999
Guanine:PMA_6AN_15m vs. ADP:PMA_6AN_15m	ns	>0.9999
Guanine:PMA_6AN_15m vs. ADP:PMA_DPI_15m	ns	>0.9999
Guanine:PMA_6AN_15m vs. ADP:PMA_AA_15m	ns	>0.9999
Guanine:PMA_6AN_15m vs. ADP:PMA_IAA_15m	ns	0.9168
Guanine:PMA_6AN_15m vs. ATP:PMA_15m	ns	0.6922

Guanine:PMA_6AN_15m vs. ATP:PMA_2DG_15m	ns	>0.9999
Guanine:PMA_6AN_15m vs. ATP:PMA_6AN_15m	ns	>0.9999
Guanine:PMA_6AN_15m vs. ATP:PMA_DPI_15m	ns	0.963
Guanine:PMA_6AN_15m vs. ATP:PMA_AA_15m	ns	>0.9999
Guanine:PMA_6AN_15m vs. ATP:PMA_IAA_15m	***	0.0007
Guanine:PMA_6AN_15m vs. Xanthine:PMA_15m	ns	>0.9999
Guanine:PMA_6AN_15m vs. Xanthine:PMA_2DG_15m	ns	>0.9999
Guanine:PMA_6AN_15m vs. Xanthine:PMA_6AN_15m	ns	>0.9999
Guanine:PMA_6AN_15m vs. Xanthine:PMA_DPI_15m	ns	>0.9999
Guanine:PMA_6AN_15m vs. Xanthine:PMA_AA_15m	ns	>0.9999
Guanine:PMA_6AN_15m vs. CMP:PMA_15m	ns	>0.9999
Guanine:PMA_6AN_15m vs. CMP:PMA_2DG_15m	ns	>0.9999
Guanine:PMA_6AN_15m vs. CMP:PMA_6AN_15m	ns	>0.9999
Guanine:PMA_6AN_15m vs. CMP:PMA_DPI_15m	ns	>0.9999
Guanine:PMA_6AN_15m vs. CMP:PMA_AA_15m	ns	>0.9999
Guanine:PMA_6AN_15m vs. CMP:PMA_IAA_15m	ns	0.9978
Guanine:PMA_6AN_15m vs. CDP:PMA_15m	ns	>0.9999
Guanine:PMA_6AN_15m vs. CDP:PMA_2DG_15m	ns	>0.9999
Guanine:PMA_6AN_15m vs. CDP:PMA_6AN_15m	ns	0.9683
Guanine:PMA_6AN_15m vs. CDP:PMA_DPI_15m	ns	>0.9999
Guanine:PMA_6AN_15m vs. CDP:PMA_AA_15m	ns	0.9927
Guanine:PMA_6AN_15m vs. CDP:PMA_IAA_15m	ns	>0.9999
Guanine:PMA_6AN_15m vs. CTP:PMA_15m	ns	0.9872
Guanine:PMA_6AN_15m vs. CTP:PMA_2DG_15m	ns	>0.9999
Guanine:PMA_6AN_15m vs. CTP:PMA_6AN_15m	ns	>0.9999
Guanine:PMA_6AN_15m vs. CTP:PMA_DPI_15m	ns	>0.9999
Guanine:PMA_6AN_15m vs. CTP:PMA_AA_15m	ns	>0.9999
Guanine:PMA_6AN_15m vs. CTP:PMA_IAA_15m	*	0.0102
Guanine:PMA_6AN_15m vs. Uridine:PMA_15m	ns	>0.9999
Guanine:PMA_6AN_15m vs. Uridine:PMA_2DG_15m	ns	>0.9999
Guanine:PMA_6AN_15m vs. Uridine:PMA_6AN_15m	ns	0.9993
Guanine:PMA_6AN_15m vs. Uridine:PMA_DPI_15m	ns	>0.9999
Guanine:PMA_6AN_15m vs. Uridine:PMA_AA_15m	ns	0.9999
Guanine:PMA_6AN_15m vs. Uridine:PMA_IAA_15m	ns	0.9998
Guanine:PMA_6AN_15m vs. UMP:PMA_15m	ns	>0.9999
Guanine:PMA_6AN_15m vs. UMP:PMA_2DG_15m	ns	0.9991
Guanine:PMA_6AN_15m vs. UMP:PMA_6AN_15m	ns	0.7504
Guanine:PMA_6AN_15m vs. UMP:PMA_DPI_15m	ns	>0.9999
Guanine:PMA_6AN_15m vs. UMP:PMA_AA_15m	ns	0.9033
Guanine:PMA_6AN_15m vs. UMP:PMA_IAA_15m	ns	>0.9999
Guanine:PMA_6AN_15m vs. UDP:PMA_15m	ns	>0.9999
Guanine:PMA_6AN_15m vs. UDP:PMA_2DG_15m	ns	0.9993
Guanine:PMA_6AN_15m vs. UDP:PMA_6AN_15m	ns	0.7674
Guanine:PMA_6AN_15m vs. UDP:PMA_DPI_15m	ns	>0.9999
Guanine:PMA_6AN_15m vs. UDP:PMA_AA_15m	ns	0.912
Guanine:PMA_6AN_15m vs. UDP:PMA_IAA_15m	ns	>0.9999
Guanine:PMA_6AN_15m vs. UTP:PMA_15m	ns	0.9507

Guanine:PMA_6AN_15m vs. UTP:PMA_2DG_15m	ns	>0.9999
Guanine:PMA_6AN_15m vs. UTP:PMA_6AN_15m	ns	>0.9999
Guanine:PMA_6AN_15m vs. UTP:PMA_DPI_15m	ns	0.9994
Guanine:PMA_6AN_15m vs. UTP:PMA_AA_15m	ns	>0.9999
Guanine:PMA_6AN_15m vs. UTP:PMA_IAA_15m	**	0.0047
Guanine:PMA_DPI_15m vs. Guanine:PMA_AA_15m	ns	0.5731
Guanine:PMA_DPI_15m vs. Guanine:PMA_IAA_15m	***	0.0004
Guanine:PMA_DPI_15m vs. Aspartic acid:PMA_15m	ns	>0.9999
Guanine:PMA_DPI_15m vs. Aspartic acid:PMA_2DG_15m	ns	>0.9999
Guanine:PMA_DPI_15m vs. Aspartic acid:PMA_6AN_15m	ns	>0.9999
Guanine:PMA_DPI_15m vs. Aspartic acid:PMA_DPI_15m	ns	>0.9999
Guanine:PMA_DPI_15m vs. Aspartic acid:PMA_AA_15m	ns	>0.9999
Guanine:PMA_DPI_15m vs. Aspartic acid:PMA_IAA_15m	ns	0.9964
Guanine:PMA_DPI_15m vs. AMP:PMA_15m	ns	>0.9999
Guanine:PMA_DPI_15m vs. AMP:PMA_2DG_15m	ns	0.9708
Guanine:PMA_DPI_15m vs. AMP:PMA_6AN_15m	ns	0.7519
Guanine:PMA_DPI_15m vs. AMP:PMA_DPI_15m	ns	>0.9999
Guanine:PMA_DPI_15m vs. AMP:PMA_AA_15m	ns	0.6386
Guanine:PMA_DPI_15m vs. AMP:PMA_IAA_15m	ns	>0.9999
Guanine:PMA_DPI_15m vs. ADP:PMA_15m	ns	>0.9999
Guanine:PMA_DPI_15m vs. ADP:PMA_2DG_15m	ns	0.9988
Guanine:PMA_DPI_15m vs. ADP:PMA_6AN_15m	ns	0.9456
Guanine:PMA_DPI_15m vs. ADP:PMA_DPI_15m	ns	>0.9999
Guanine:PMA_DPI_15m vs. ADP:PMA_AA_15m	ns	0.8898
Guanine:PMA_DPI_15m vs. ADP:PMA_IAA_15m	ns	>0.9999
Guanine:PMA_DPI_15m vs. ATP:PMA_15m	ns	>0.9999
Guanine:PMA_DPI_15m vs. ATP:PMA_2DG_15m	ns	>0.9999
Guanine:PMA_DPI_15m vs. ATP:PMA_6AN_15m	ns	>0.9999
Guanine:PMA_DPI_15m vs. ATP:PMA_DPI_15m	ns	>0.9999
Guanine:PMA_DPI_15m vs. ATP:PMA_AA_15m	ns	>0.9999
Guanine:PMA_DPI_15m vs. ATP:PMA_IAA_15m	ns	0.1603
Guanine:PMA_DPI_15m vs. Xanthine:PMA_15m	ns	>0.9999
Guanine:PMA_DPI_15m vs. Xanthine:PMA_2DG_15m	ns	>0.9999
Guanine:PMA_DPI_15m vs. Xanthine:PMA_6AN_15m	ns	>0.9999
Guanine:PMA_DPI_15m vs. Xanthine:PMA_DPI_15m	ns	>0.9999
Guanine:PMA_DPI_15m vs. Xanthine:PMA_AA_15m	ns	>0.9999
Guanine:PMA_DPI_15m vs. CMP:PMA_15m	ns	>0.9999
Guanine:PMA_DPI_15m vs. CMP:PMA_2DG_15m	ns	0.9362
Guanine:PMA_DPI_15m vs. CMP:PMA_6AN_15m	ns	0.6466
Guanine:PMA_DPI_15m vs. CMP:PMA_DPI_15m	ns	>0.9999
Guanine:PMA_DPI_15m vs. CMP:PMA_AA_15m	ns	0.5264
Guanine:PMA_DPI_15m vs. CMP:PMA_IAA_15m	ns	>0.9999
Guanine:PMA_DPI_15m vs. CDP:PMA_15m	ns	>0.9999
Guanine:PMA_DPI_15m vs. CDP:PMA_2DG_15m	ns	0.5522
Guanine:PMA_DPI_15m vs. CDP:PMA_6AN_15m	ns	0.2139
Guanine:PMA_DPI_15m vs. CDP:PMA_DPI_15m	ns	0.9683
Guanine:PMA_DPI_15m vs. CDP:PMA_AA_15m	ns	0.1475

Guanine:PMA_DPI_15m vs. CDP:PMA_IAA_15m	ns	>0.9999
Guanine:PMA_DPI_15m vs. CTP:PMA_15m	ns	>0.9999
Guanine:PMA_DPI_15m vs. CTP:PMA_2DG_15m	ns	>0.9999
Guanine:PMA_DPI_15m vs. CTP:PMA_6AN_15m	ns	>0.9999
Guanine:PMA_DPI_15m vs. CTP:PMA_DPI_15m	ns	>0.9999
Guanine:PMA_DPI_15m vs. CTP:PMA_AA_15m	ns	>0.9999
Guanine:PMA_DPI_15m vs. CTP:PMA_IAA_15m	ns	0.6184
Guanine:PMA_DPI_15m vs. Uridine:PMA_15m	ns	>0.9999
Guanine:PMA_DPI_15m vs. Uridine:PMA_2DG_15m	ns	0.8388
Guanine:PMA_DPI_15m vs. Uridine:PMA_6AN_15m	ns	0.4728
Guanine:PMA_DPI_15m vs. Uridine:PMA_DPI_15m	ns	0.9993
Guanine:PMA_DPI_15m vs. Uridine:PMA_AA_15m	ns	0.361
Guanine:PMA_DPI_15m vs. Uridine:PMA_IAA_15m	ns	>0.9999
Guanine:PMA_DPI_15m vs. UMP:PMA_15m	ns	0.9995
Guanine:PMA_DPI_15m vs. UMP:PMA_2DG_15m	ns	0.2518
Guanine:PMA_DPI_15m vs. UMP:PMA_6AN_15m	ns	0.0692
Guanine:PMA_DPI_15m vs. UMP:PMA_DPI_15m	ns	0.7504
Guanine:PMA_DPI_15m vs. UMP:PMA_AA_15m	*	0.0438
Guanine:PMA_DPI_15m vs. UMP:PMA_IAA_15m	ns	>0.9999
Guanine:PMA_DPI_15m vs. UDP:PMA_15m	ns	0.9996
Guanine:PMA_DPI_15m vs. UDP:PMA_2DG_15m	ns	0.264
Guanine:PMA_DPI_15m vs. UDP:PMA_6AN_15m	ns	0.0737
Guanine:PMA_DPI_15m vs. UDP:PMA_DPI_15m	ns	0.7674
Guanine:PMA_DPI_15m vs. UDP:PMA_AA_15m	*	0.0468
Guanine:PMA_DPI_15m vs. UDP:PMA_IAA_15m	ns	>0.9999
Guanine:PMA_DPI_15m vs. UTP:PMA_15m	ns	>0.9999
Guanine:PMA_DPI_15m vs. UTP:PMA_2DG_15m	ns	>0.9999
Guanine:PMA_DPI_15m vs. UTP:PMA_6AN_15m	ns	>0.9999
Guanine:PMA_DPI_15m vs. UTP:PMA_DPI_15m	ns	>0.9999
Guanine:PMA_DPI_15m vs. UTP:PMA_AA_15m	ns	>0.9999
Guanine:PMA_DPI_15m vs. UTP:PMA_IAA_15m	ns	0.4505
Guanine:PMA_AA_15m vs. Guanine:PMA_IAA_15m	****	<0.0001
Guanine:PMA_AA_15m vs. Aspartic acid:PMA_15m	ns	>0.9999
Guanine:PMA_AA_15m vs. Aspartic acid:PMA_2DG_15m	ns	>0.9999
Guanine:PMA_AA_15m vs. Aspartic acid:PMA_6AN_15m	ns	>0.9999
Guanine:PMA_AA_15m vs. Aspartic acid:PMA_DPI_15m	ns	>0.9999
Guanine:PMA_AA_15m vs. Aspartic acid:PMA_AA_15m	ns	>0.9999
Guanine:PMA_AA_15m vs. Aspartic acid:PMA_IAA_15m	ns	0.1252
Guanine:PMA_AA_15m vs. AMP:PMA_15m	ns	>0.9999
Guanine:PMA_AA_15m vs. AMP:PMA_2DG_15m	ns	>0.9999
Guanine:PMA_AA_15m vs. AMP:PMA_6AN_15m	ns	>0.9999
Guanine:PMA_AA_15m vs. AMP:PMA_DPI_15m	ns	>0.9999
Guanine:PMA_AA_15m vs. AMP:PMA_AA_15m	ns	>0.9999
Guanine:PMA_AA_15m vs. AMP:PMA_IAA_15m	ns	0.977
Guanine:PMA_AA_15m vs. ADP:PMA_15m	ns	>0.9999
Guanine:PMA_AA_15m vs. ADP:PMA_2DG_15m	ns	>0.9999
Guanine:PMA_AA_15m vs. ADP:PMA_6AN_15m	ns	>0.9999

Guanine:PMA_AA_15m vs. ADP:PMA_DPI_15m	ns	>0.9999
Guanine:PMA_AA_15m vs. ADP:PMA_AA_15m	ns	>0.9999
Guanine:PMA_AA_15m vs. ADP:PMA_IAA_15m	ns	0.8462
Guanine:PMA_AA_15m vs. ATP:PMA_15m	ns	0.5736
Guanine:PMA_AA_15m vs. ATP:PMA_2DG_15m	ns	>0.9999
Guanine:PMA_AA_15m vs. ATP:PMA_6AN_15m	ns	>0.9999
Guanine:PMA_AA_15m vs. ATP:PMA_DPI_15m	ns	0.9189
Guanine:PMA_AA_15m vs. ATP:PMA_AA_15m	ns	>0.9999
Guanine:PMA_AA_15m vs. ATP:PMA_IAA_15m	***	0.0004
Guanine:PMA_AA_15m vs. Xanthine:PMA_15m	ns	0.9998
Guanine:PMA_AA_15m vs. Xanthine:PMA_2DG_15m	ns	>0.9999
Guanine:PMA_AA_15m vs. Xanthine:PMA_6AN_15m	ns	>0.9999
Guanine:PMA_AA_15m vs. Xanthine:PMA_DPI_15m	ns	>0.9999
Guanine:PMA_AA_15m vs. Xanthine:PMA_AA_15m	ns	>0.9999
Guanine:PMA_AA_15m vs. CMP:PMA_15m	ns	>0.9999
Guanine:PMA_AA_15m vs. CMP:PMA_2DG_15m	ns	>0.9999
Guanine:PMA_AA_15m vs. CMP:PMA_6AN_15m	ns	>0.9999
Guanine:PMA_AA_15m vs. CMP:PMA_DPI_15m	ns	>0.9999
Guanine:PMA_AA_15m vs. CMP:PMA_AA_15m	ns	>0.9999
Guanine:PMA_AA_15m vs. CMP:PMA_IAA_15m	ns	0.9916
Guanine:PMA_AA_15m vs. CDP:PMA_15m	ns	>0.9999
Guanine:PMA_AA_15m vs. CDP:PMA_2DG_15m	ns	>0.9999
Guanine:PMA_AA_15m vs. CDP:PMA_6AN_15m	ns	0.9997
Guanine:PMA_AA_15m vs. CDP:PMA_DPI_15m	ns	>0.9999
Guanine:PMA_AA_15m vs. CDP:PMA_AA_15m	ns	0.9683
Guanine:PMA_AA_15m vs. CDP:PMA_IAA_15m	ns	>0.9999
Guanine:PMA_AA_15m vs. CTP:PMA_15m	ns	0.9653
Guanine:PMA_AA_15m vs. CTP:PMA_2DG_15m	ns	>0.9999
Guanine:PMA_AA_15m vs. CTP:PMA_6AN_15m	ns	>0.9999
Guanine:PMA_AA_15m vs. CTP:PMA_DPI_15m	ns	0.9997
Guanine:PMA_AA_15m vs. CTP:PMA_AA_15m	ns	>0.9999
Guanine:PMA_AA_15m vs. CTP:PMA_IAA_15m	**	0.006
Guanine:PMA_AA_15m vs. Uridine:PMA_15m	ns	>0.9999
Guanine:PMA_AA_15m vs. Uridine:PMA_2DG_15m	ns	>0.9999
Guanine:PMA_AA_15m vs. Uridine:PMA_6AN_15m	ns	>0.9999
Guanine:PMA_AA_15m vs. Uridine:PMA_DPI_15m	ns	>0.9999
Guanine:PMA_AA_15m vs. Uridine:PMA_AA_15m	ns	0.9993
Guanine:PMA_AA_15m vs. Uridine:PMA_IAA_15m	ns	0.9989
Guanine:PMA_AA_15m vs. UMP:PMA_15m	ns	>0.9999
Guanine:PMA_AA_15m vs. UMP:PMA_2DG_15m	ns	0.9999
Guanine:PMA_AA_15m vs. UMP:PMA_6AN_15m	ns	0.9817
Guanine:PMA_AA_15m vs. UMP:PMA_DPI_15m	ns	>0.9999
Guanine:PMA_AA_15m vs. UMP:PMA_AA_15m	ns	0.7504
Guanine:PMA_AA_15m vs. UMP:PMA_IAA_15m	ns	>0.9999
Guanine:PMA_AA_15m vs. UDP:PMA_15m	ns	>0.9999
Guanine:PMA_AA_15m vs. UDP:PMA_2DG_15m	ns	0.9999
Guanine:PMA_AA_15m vs. UDP:PMA_6AN_15m	ns	0.9842

Guanine:PMA_AA_15m vs. UDP:PMA_DPI_15m	ns	>0.9999
Guanine:PMA_AA_15m vs. UDP:PMA_AA_15m	ns	0.7674
Guanine:PMA_AA_15m vs. UDP:PMA_IAA_15m	ns	>0.9999
Guanine:PMA_AA_15m vs. UTP:PMA_15m	ns	0.8982
Guanine:PMA_AA_15m vs. UTP:PMA_2DG_15m	ns	>0.9999
Guanine:PMA_AA_15m vs. UTP:PMA_6AN_15m	ns	>0.9999
Guanine:PMA_AA_15m vs. UTP:PMA_DPI_15m	ns	0.997
Guanine:PMA_AA_15m vs. UTP:PMA_AA_15m	ns	>0.9999
Guanine:PMA_AA_15m vs. UTP:PMA_IAA_15m	**	0.0027
Guanine:PMA_IAA_15m vs. Aspartic acid:PMA_15m	ns	0.9938
Guanine:PMA_IAA_15m vs. Aspartic acid:PMA_2DG_15m	ns	0.1415
Guanine:PMA_IAA_15m vs. Aspartic acid:PMA_6AN_15m	*	0.0335
Guanine:PMA_IAA_15m vs. Aspartic acid:PMA_DPI_15m	ns	0.8601
Guanine:PMA_IAA_15m vs. Aspartic acid:PMA_AA_15m	*	0.0204
Guanine:PMA_IAA_15m vs. Aspartic acid:PMA_IAA_15m	ns	>0.9999
Guanine:PMA_IAA_15m vs. AMP:PMA_15m	ns	0.196
Guanine:PMA_IAA_15m vs. AMP:PMA_2DG_15m	***	0.0009
Guanine:PMA_IAA_15m vs. AMP:PMA_6AN_15m	***	0.0001
Guanine:PMA_IAA_15m vs. AMP:PMA_DPI_15m	*	0.0442
Guanine:PMA_IAA_15m vs. AMP:PMA_AA_15m	****	<0.0001
Guanine:PMA_IAA_15m vs. AMP:PMA_IAA_15m	ns	>0.9999
Guanine:PMA_IAA_15m vs. ADP:PMA_15m	ns	0.4363
Guanine:PMA_IAA_15m vs. ADP:PMA_2DG_15m	**	0.0038
Guanine:PMA_IAA_15m vs. ADP:PMA_6AN_15m	***	0.0006
Guanine:PMA_IAA_15m vs. ADP:PMA_DPI_15m	ns	0.134
Guanine:PMA_IAA_15m vs. ADP:PMA_AA_15m	***	0.0003
Guanine:PMA_IAA_15m vs. ADP:PMA_IAA_15m	ns	>0.9999
Guanine:PMA_IAA_15m vs. ATP:PMA_15m	ns	>0.9999
Guanine:PMA_IAA_15m vs. ATP:PMA_2DG_15m	ns	0.9945
Guanine:PMA_IAA_15m vs. ATP:PMA_6AN_15m	ns	0.8874
Guanine:PMA_IAA_15m vs. ATP:PMA_DPI_15m	ns	>0.9999
Guanine:PMA_IAA_15m vs. ATP:PMA_AA_15m	ns	0.8048
Guanine:PMA_IAA_15m vs. ATP:PMA_IAA_15m	ns	>0.9999
Guanine:PMA_IAA_15m vs. Xanthine:PMA_15m	ns	>0.9999
Guanine:PMA_IAA_15m vs. Xanthine:PMA_2DG_15m	ns	0.4653
Guanine:PMA_IAA_15m vs. Xanthine:PMA_6AN_15m	ns	0.1677
Guanine:PMA_IAA_15m vs. Xanthine:PMA_DPI_15m	ns	0.9938
Guanine:PMA_IAA_15m vs. Xanthine:PMA_AA_15m	ns	0.1139
Guanine:PMA_IAA_15m vs. CMP:PMA_15m	ns	0.1378
Guanine:PMA_IAA_15m vs. CMP:PMA_2DG_15m	***	0.0005
Guanine:PMA_IAA_15m vs. CMP:PMA_6AN_15m	****	<0.0001
Guanine:PMA_IAA_15m vs. CMP:PMA_DPI_15m	*	0.0283
Guanine:PMA_IAA_15m vs. CMP:PMA_AA_15m	****	<0.0001
Guanine:PMA_IAA_15m vs. CMP:PMA_IAA_15m	ns	>0.9999
Guanine:PMA_IAA_15m vs. CDP:PMA_15m	*	0.022
Guanine:PMA_IAA_15m vs. CDP:PMA_2DG_15m	****	<0.0001
Guanine:PMA_IAA_15m vs. CDP:PMA_6AN_15m	****	<0.0001

Guanine:PMA_IAA_15m vs. CDP:PMA_DPI_15m	**	0.0033
Guanine:PMA_IAA_15m vs. CDP:PMA_AA_15m	****	<0.0001
Guanine:PMA_IAA_15m vs. CDP:PMA_IAA_15m	ns	0.9683
Guanine:PMA_IAA_15m vs. CTP:PMA_15m	ns	>0.9999
Guanine:PMA_IAA_15m vs. CTP:PMA_2DG_15m	ns	0.7704
Guanine:PMA_IAA_15m vs. CTP:PMA_6AN_15m	ns	0.3921
Guanine:PMA_IAA_15m vs. CTP:PMA_DPI_15m	ns	>0.9999
Guanine:PMA_IAA_15m vs. CTP:PMA_AA_15m	ns	0.2909
Guanine:PMA_IAA_15m vs. CTP:PMA_IAA_15m	ns	>0.9999
Guanine:PMA_IAA_15m vs. Uridine:PMA_15m	ns	0.0754
Guanine:PMA_IAA_15m vs. Uridine:PMA_2DG_15m	***	0.0002
Guanine:PMA_IAA_15m vs. Uridine:PMA_6AN_15m	****	<0.0001
Guanine:PMA_IAA_15m vs. Uridine:PMA_DPI_15m	*	0.0136
Guanine:PMA_IAA_15m vs. Uridine:PMA_AA_15m	****	<0.0001
Guanine:PMA_IAA_15m vs. Uridine:PMA_IAA_15m	ns	0.9993
Guanine:PMA_IAA_15m vs. UMP:PMA_15m	**	0.005
Guanine:PMA_IAA_15m vs. UMP:PMA_2DG_15m	****	<0.0001
Guanine:PMA_IAA_15m vs. UMP:PMA_6AN_15m	****	<0.0001
Guanine:PMA_IAA_15m vs. UMP:PMA_DPI_15m	***	0.0006
Guanine:PMA_IAA_15m vs. UMP:PMA_AA_15m	****	<0.0001
Guanine:PMA_IAA_15m vs. UMP:PMA_IAA_15m	ns	0.7504
Guanine:PMA_IAA_15m vs. UDP:PMA_15m	**	0.0054
Guanine:PMA_IAA_15m vs. UDP:PMA_2DG_15m	****	<0.0001
Guanine:PMA_IAA_15m vs. UDP:PMA_6AN_15m	****	<0.0001
Guanine:PMA_IAA_15m vs. UDP:PMA_DPI_15m	***	0.0007
Guanine:PMA_IAA_15m vs. UDP:PMA_AA_15m	****	<0.0001
Guanine:PMA_IAA_15m vs. UDP:PMA_IAA_15m	ns	0.7674
Guanine:PMA_IAA_15m vs. UTP:PMA_15m	ns	>0.9999
Guanine:PMA_IAA_15m vs. UTP:PMA_2DG_15m	ns	0.8916
Guanine:PMA_IAA_15m vs. UTP:PMA_6AN_15m	ns	0.5559
Guanine:PMA_IAA_15m vs. UTP:PMA_DPI_15m	ns	>0.9999
Guanine:PMA_IAA_15m vs. UTP:PMA_AA_15m	ns	0.4379
Guanine:PMA_IAA_15m vs. UTP:PMA_IAA_15m	ns	>0.9999
Aspartic acid:PMA_15m vs. Aspartic acid:PMA_2DG_15m	ns	0.7475
Aspartic acid:PMA_15m vs. Aspartic acid:PMA_6AN_15m	ns	0.1037
Aspartic acid:PMA_15m vs. Aspartic acid:PMA_DPI_15m	ns	>0.9999
Aspartic acid:PMA_15m vs. Aspartic acid:PMA_AA_15m	*	0.0409
Aspartic acid:PMA_15m vs. Aspartic acid:PMA_IAA_15m	*	0.0236
Aspartic acid:PMA_15m vs. AMP:PMA_15m	ns	>0.9999
Aspartic acid:PMA_15m vs. AMP:PMA_2DG_15m	ns	0.9
Aspartic acid:PMA_15m vs. AMP:PMA_6AN_15m	ns	0.5697
Aspartic acid:PMA_15m vs. AMP:PMA_DPI_15m	ns	>0.9999
Aspartic acid:PMA_15m vs. AMP:PMA_AA_15m	ns	0.4506
Aspartic acid:PMA_15m vs. AMP:PMA_IAA_15m	ns	>0.9999
Aspartic acid:PMA_15m vs. ADP:PMA_15m	ns	>0.9999
Aspartic acid:PMA_15m vs. ADP:PMA_2DG_15m	ns	0.9895
Aspartic acid:PMA_15m vs. ADP:PMA_6AN_15m	ns	0.8459

Aspartic acid:PMA_15m vs. ADP:PMA_DPI_15m	ns	>0.9999
Aspartic acid:PMA_15m vs. ADP:PMA_AA_15m	ns	0.75
Aspartic acid:PMA_15m vs. ADP:PMA_IAA_15m	ns	>0.9999
Aspartic acid:PMA_15m vs. ATP:PMA_15m	ns	>0.9999
Aspartic acid:PMA_15m vs. ATP:PMA_2DG_15m	ns	>0.9999
Aspartic acid:PMA_15m vs. ATP:PMA_6AN_15m	ns	>0.9999
Aspartic acid:PMA_15m vs. ATP:PMA_DPI_15m	ns	>0.9999
Aspartic acid:PMA_15m vs. ATP:PMA_AA_15m	ns	>0.9999
Aspartic acid:PMA_15m vs. ATP:PMA_IAA_15m	ns	0.2793
Aspartic acid:PMA_15m vs. Xanthine:PMA_15m	ns	>0.9999
Aspartic acid:PMA_15m vs. Xanthine:PMA_2DG_15m	ns	>0.9999
Aspartic acid:PMA_15m vs. Xanthine:PMA_6AN_15m	ns	>0.9999
Aspartic acid:PMA_15m vs. Xanthine:PMA_DPI_15m	ns	>0.9999
Aspartic acid:PMA_15m vs. Xanthine:PMA_AA_15m	ns	>0.9999
Aspartic acid:PMA_15m vs. CMP:PMA_15m	ns	>0.9999
Aspartic acid:PMA_15m vs. CMP:PMA_2DG_15m	ns	0.828
Aspartic acid:PMA_15m vs. CMP:PMA_6AN_15m	ns	0.4584
Aspartic acid:PMA_15m vs. CMP:PMA_DPI_15m	ns	>0.9999
Aspartic acid:PMA_15m vs. CMP:PMA_AA_15m	ns	0.3482
Aspartic acid:PMA_15m vs. CMP:PMA_IAA_15m	ns	>0.9999
Aspartic acid:PMA_15m vs. CDP:PMA_15m	ns	0.9978
Aspartic acid:PMA_15m vs. CDP:PMA_2DG_15m	ns	0.3706
Aspartic acid:PMA_15m vs. CDP:PMA_6AN_15m	ns	0.1174
Aspartic acid:PMA_15m vs. CDP:PMA_DPI_15m	ns	0.9858
Aspartic acid:PMA_15m vs. CDP:PMA_AA_15m	ns	0.0769
Aspartic acid:PMA_15m vs. CDP:PMA_IAA_15m	ns	>0.9999
Aspartic acid:PMA_15m vs. CTP:PMA_15m	ns	>0.9999
Aspartic acid:PMA_15m vs. CTP:PMA_2DG_15m	ns	>0.9999
Aspartic acid:PMA_15m vs. CTP:PMA_6AN_15m	ns	>0.9999
Aspartic acid:PMA_15m vs. CTP:PMA_DPI_15m	ns	>0.9999
Aspartic acid:PMA_15m vs. CTP:PMA_AA_15m	ns	>0.9999
Aspartic acid:PMA_15m vs. CTP:PMA_IAA_15m	ns	0.7926
Aspartic acid:PMA_15m vs. Uridine:PMA_15m	ns	>0.9999
Aspartic acid:PMA_15m vs. Uridine:PMA_2DG_15m	ns	0.6768
Aspartic acid:PMA_15m vs. Uridine:PMA_6AN_15m	ns	0.3034
Aspartic acid:PMA_15m vs. Uridine:PMA_DPI_15m	ns	0.9997
Aspartic acid:PMA_15m vs. Uridine:PMA_AA_15m	ns	0.2173
Aspartic acid:PMA_15m vs. Uridine:PMA_IAA_15m	ns	>0.9999
Aspartic acid:PMA_15m vs. UMP:PMA_15m	ns	0.9313
Aspartic acid:PMA_15m vs. UMP:PMA_2DG_15m	ns	0.1417
Aspartic acid:PMA_15m vs. UMP:PMA_6AN_15m	*	0.0333
Aspartic acid:PMA_15m vs. UMP:PMA_DPI_15m	ns	0.8624
Aspartic acid:PMA_15m vs. UMP:PMA_AA_15m	*	0.0203
Aspartic acid:PMA_15m vs. UMP:PMA_IAA_15m	ns	>0.9999
Aspartic acid:PMA_15m vs. UDP:PMA_15m	ns	0.9391
Aspartic acid:PMA_15m vs. UDP:PMA_2DG_15m	ns	0.1498
Aspartic acid:PMA_15m vs. UDP:PMA_6AN_15m	*	0.0357

Aspartic acid:PMA_15m vs. UDP:PMA_DPI_15m	ns	0.8733
Aspartic acid:PMA_15m vs. UDP:PMA_AA_15m	*	0.0218
Aspartic acid:PMA_15m vs. UDP:PMA_IAA_15m	ns	>0.9999
Aspartic acid:PMA_15m vs. UTP:PMA_15m	ns	>0.9999
Aspartic acid:PMA_15m vs. UTP:PMA_2DG_15m	ns	>0.9999
Aspartic acid:PMA_15m vs. UTP:PMA_6AN_15m	ns	>0.9999
Aspartic acid:PMA_15m vs. UTP:PMA_DPI_15m	ns	>0.9999
Aspartic acid:PMA_15m vs. UTP:PMA_AA_15m	ns	>0.9999
Aspartic acid:PMA_15m vs. UTP:PMA_IAA_15m	ns	0.6377
Aspartic acid:PMA_2DG_15m vs. Aspartic acid:PMA_6AN_15m	ns	>0.9999
Aspartic acid:PMA_2DG_15m vs. Aspartic acid:PMA_DPI_15m	ns	>0.9999
Aspartic acid:PMA_2DG_15m vs. Aspartic acid:PMA_AA_15m	ns	>0.9999
Aspartic acid:PMA_2DG_15m vs. Aspartic acid:PMA_IAA_15m	****	<0.0001
Aspartic acid:PMA_2DG_15m vs. AMP:PMA_15m	ns	>0.9999
Aspartic acid:PMA_2DG_15m vs. AMP:PMA_2DG_15m	ns	>0.9999
Aspartic acid:PMA_2DG_15m vs. AMP:PMA_6AN_15m	ns	>0.9999
Aspartic acid:PMA_2DG_15m vs. AMP:PMA_DPI_15m	ns	>0.9999
Aspartic acid:PMA_2DG_15m vs. AMP:PMA_AA_15m	ns	>0.9999
Aspartic acid:PMA_2DG_15m vs. AMP:PMA_IAA_15m	ns	0.9987
Aspartic acid:PMA_2DG_15m vs. ADP:PMA_15m	ns	>0.9999
Aspartic acid:PMA_2DG_15m vs. ADP:PMA_2DG_15m	ns	>0.9999
Aspartic acid:PMA_2DG_15m vs. ADP:PMA_6AN_15m	ns	>0.9999
Aspartic acid:PMA_2DG_15m vs. ADP:PMA_DPI_15m	ns	>0.9999
Aspartic acid:PMA_2DG_15m vs. ADP:PMA_AA_15m	ns	>0.9999
Aspartic acid:PMA_2DG_15m vs. ADP:PMA_IAA_15m	ns	0.9706
Aspartic acid:PMA_2DG_15m vs. ATP:PMA_15m	ns	0.8261
Aspartic acid:PMA_2DG_15m vs. ATP:PMA_2DG_15m	ns	>0.9999
Aspartic acid:PMA_2DG_15m vs. ATP:PMA_6AN_15m	ns	>0.9999
Aspartic acid:PMA_2DG_15m vs. ATP:PMA_DPI_15m	ns	0.9901
Aspartic acid:PMA_2DG_15m vs. ATP:PMA_AA_15m	ns	>0.9999
Aspartic acid:PMA_2DG_15m vs. ATP:PMA_IAA_15m	**	0.0016
Aspartic acid:PMA_2DG_15m vs. Xanthine:PMA_15m	ns	>0.9999
Aspartic acid:PMA_2DG_15m vs. Xanthine:PMA_2DG_15m	ns	>0.9999
Aspartic acid:PMA_2DG_15m vs. Xanthine:PMA_6AN_15m	ns	>0.9999
Aspartic acid:PMA_2DG_15m vs. Xanthine:PMA_DPI_15m	ns	>0.9999
Aspartic acid:PMA_2DG_15m vs. Xanthine:PMA_AA_15m	ns	>0.9999
Aspartic acid:PMA_2DG_15m vs. CMP:PMA_15m	ns	>0.9999
Aspartic acid:PMA_2DG_15m vs. CMP:PMA_2DG_15m	ns	>0.9999
Aspartic acid:PMA_2DG_15m vs. CMP:PMA_6AN_15m	ns	>0.9999
Aspartic acid:PMA_2DG_15m vs. CMP:PMA_DPI_15m	ns	>0.9999
Aspartic acid:PMA_2DG_15m vs. CMP:PMA_AA_15m	ns	>0.9999
Aspartic acid:PMA_2DG_15m vs. CMP:PMA_IAA_15m	ns	0.9998
Aspartic acid:PMA_2DG_15m vs. CDP:PMA_15m	ns	>0.9999
Aspartic acid:PMA_2DG_15m vs. CDP:PMA_2DG_15m	ns	0.9978
Aspartic acid:PMA_2DG_15m vs. CDP:PMA_6AN_15m	ns	0.9896
Aspartic acid:PMA_2DG_15m vs. CDP:PMA_DPI_15m	ns	>0.9999
Aspartic acid:PMA_2DG_15m vs. CDP:PMA_AA_15m	ns	0.9706

Aspartic acid:PMA_2DG_15m vs. CDP:PMA_IAA_15m	ns	>0.9999
Aspartic acid:PMA_2DG_15m vs. CTP:PMA_15m	ns	0.9975
Aspartic acid:PMA_2DG_15m vs. CTP:PMA_2DG_15m	ns	>0.9999
Aspartic acid:PMA_2DG_15m vs. CTP:PMA_6AN_15m	ns	>0.9999
Aspartic acid:PMA_2DG_15m vs. CTP:PMA_DPI_15m	ns	>0.9999
Aspartic acid:PMA_2DG_15m vs. CTP:PMA_AA_15m	ns	>0.9999
Aspartic acid:PMA_2DG_15m vs. CTP:PMA_IAA_15m	*	0.02
Aspartic acid:PMA_2DG_15m vs. Uridine:PMA_15m	ns	>0.9999
Aspartic acid:PMA_2DG_15m vs. Uridine:PMA_2DG_15m	ns	>0.9999
Aspartic acid:PMA_2DG_15m vs. Uridine:PMA_6AN_15m	ns	0.9998
Aspartic acid:PMA_2DG_15m vs. Uridine:PMA_DPI_15m	ns	>0.9999
Aspartic acid:PMA_2DG_15m vs. Uridine:PMA_AA_15m	ns	0.9989
Aspartic acid:PMA_2DG_15m vs. Uridine:PMA_IAA_15m	ns	>0.9999
Aspartic acid:PMA_2DG_15m vs. UMP:PMA_15m	ns	>0.9999
Aspartic acid:PMA_2DG_15m vs. UMP:PMA_2DG_15m	ns	0.9313
Aspartic acid:PMA_2DG_15m vs. UMP:PMA_6AN_15m	ns	0.8833
Aspartic acid:PMA_2DG_15m vs. UMP:PMA_DPI_15m	ns	>0.9999
Aspartic acid:PMA_2DG_15m vs. UMP:PMA_AA_15m	ns	0.7989
Aspartic acid:PMA_2DG_15m vs. UMP:PMA_IAA_15m	ns	>0.9999
Aspartic acid:PMA_2DG_15m vs. UDP:PMA_15m	ns	>0.9999
Aspartic acid:PMA_2DG_15m vs. UDP:PMA_2DG_15m	ns	0.9391
Aspartic acid:PMA_2DG_15m vs. UDP:PMA_6AN_15m	ns	0.8932
Aspartic acid:PMA_2DG_15m vs. UDP:PMA_DPI_15m	ns	>0.9999
Aspartic acid:PMA_2DG_15m vs. UDP:PMA_AA_15m	ns	0.8123
Aspartic acid:PMA_2DG_15m vs. UDP:PMA_IAA_15m	ns	>0.9999
Aspartic acid:PMA_2DG_15m vs. UTP:PMA_15m	ns	0.9855
Aspartic acid:PMA_2DG_15m vs. UTP:PMA_2DG_15m	ns	>0.9999
Aspartic acid:PMA_2DG_15m vs. UTP:PMA_6AN_15m	ns	>0.9999
Aspartic acid:PMA_2DG_15m vs. UTP:PMA_DPI_15m	ns	>0.9999
Aspartic acid:PMA_2DG_15m vs. UTP:PMA_AA_15m	ns	>0.9999
Aspartic acid:PMA_2DG_15m vs. UTP:PMA_IAA_15m	**	0.0096
Aspartic acid:PMA_6AN_15m vs. Aspartic acid:PMA_DPI_15m	ns	0.8059
Aspartic acid:PMA_6AN_15m vs. Aspartic acid:PMA_AA_15m	ns	>0.9999
Aspartic acid:PMA_6AN_15m vs. Aspartic acid:PMA_IAA_15m	****	<0.0001
Aspartic acid:PMA_6AN_15m vs. AMP:PMA_15m	ns	>0.9999
Aspartic acid:PMA_6AN_15m vs. AMP:PMA_2DG_15m	ns	>0.9999
Aspartic acid:PMA_6AN_15m vs. AMP:PMA_6AN_15m	ns	>0.9999
Aspartic acid:PMA_6AN_15m vs. AMP:PMA_DPI_15m	ns	>0.9999
Aspartic acid:PMA_6AN_15m vs. AMP:PMA_AA_15m	ns	>0.9999
Aspartic acid:PMA_6AN_15m vs. AMP:PMA_IAA_15m	ns	0.9453
Aspartic acid:PMA_6AN_15m vs. ADP:PMA_15m	ns	>0.9999
Aspartic acid:PMA_6AN_15m vs. ADP:PMA_2DG_15m	ns	>0.9999
Aspartic acid:PMA_6AN_15m vs. ADP:PMA_6AN_15m	ns	>0.9999
Aspartic acid:PMA_6AN_15m vs. ADP:PMA_DPI_15m	ns	>0.9999
Aspartic acid:PMA_6AN_15m vs. ADP:PMA_AA_15m	ns	>0.9999
Aspartic acid:PMA_6AN_15m vs. ADP:PMA_IAA_15m	ns	0.7523
Aspartic acid:PMA_6AN_15m vs. ATP:PMA_15m	ns	0.456

Aspartic acid:PMA_6AN_15m vs. ATP:PMA_2DG_15m	ns	>0.9999
Aspartic acid:PMA_6AN_15m vs. ATP:PMA_6AN_15m	ns	>0.9999
Aspartic acid:PMA_6AN_15m vs. ATP:PMA_DPI_15m	ns	0.8501
Aspartic acid:PMA_6AN_15m vs. ATP:PMA_AA_15m	ns	>0.9999
Aspartic acid:PMA_6AN_15m vs. ATP:PMA_IAA_15m	***	0.0002
Aspartic acid:PMA_6AN_15m vs. Xanthine:PMA_15m	ns	0.9987
Aspartic acid:PMA_6AN_15m vs. Xanthine:PMA_2DG_15m	ns	>0.9999
Aspartic acid:PMA_6AN_15m vs. Xanthine:PMA_6AN_15m	ns	>0.9999
Aspartic acid:PMA_6AN_15m vs. Xanthine:PMA_DPI_15m	ns	>0.9999
Aspartic acid:PMA_6AN_15m vs. Xanthine:PMA_AA_15m	ns	>0.9999
Aspartic acid:PMA_6AN_15m vs. CMP:PMA_15m	ns	>0.9999
Aspartic acid:PMA_6AN_15m vs. CMP:PMA_2DG_15m	ns	>0.9999
Aspartic acid:PMA_6AN_15m vs. CMP:PMA_6AN_15m	ns	>0.9999
Aspartic acid:PMA_6AN_15m vs. CMP:PMA_DPI_15m	ns	>0.9999
Aspartic acid:PMA_6AN_15m vs. CMP:PMA_AA_15m	ns	>0.9999
Aspartic acid:PMA_6AN_15m vs. CMP:PMA_IAA_15m	ns	0.9758
Aspartic acid:PMA_6AN_15m vs. CDP:PMA_15m	ns	>0.9999
Aspartic acid:PMA_6AN_15m vs. CDP:PMA_2DG_15m	ns	>0.9999
Aspartic acid:PMA_6AN_15m vs. CDP:PMA_6AN_15m	ns	0.9978
Aspartic acid:PMA_6AN_15m vs. CDP:PMA_DPI_15m	ns	>0.9999
Aspartic acid:PMA_6AN_15m vs. CDP:PMA_AA_15m	ns	0.9996
Aspartic acid:PMA_6AN_15m vs. CDP:PMA_IAA_15m	ns	>0.9999
Aspartic acid:PMA_6AN_15m vs. CTP:PMA_15m	ns	0.9236
Aspartic acid:PMA_6AN_15m vs. CTP:PMA_2DG_15m	ns	>0.9999
Aspartic acid:PMA_6AN_15m vs. CTP:PMA_6AN_15m	ns	>0.9999
Aspartic acid:PMA_6AN_15m vs. CTP:PMA_DPI_15m	ns	0.9984
Aspartic acid:PMA_6AN_15m vs. CTP:PMA_AA_15m	ns	>0.9999
Aspartic acid:PMA_6AN_15m vs. CTP:PMA_IAA_15m	**	0.0034
Aspartic acid:PMA_6AN_15m vs. Uridine:PMA_15m	ns	>0.9999
Aspartic acid:PMA_6AN_15m vs. Uridine:PMA_2DG_15m	ns	>0.9999
Aspartic acid:PMA_6AN_15m vs. Uridine:PMA_6AN_15m	ns	>0.9999
Aspartic acid:PMA_6AN_15m vs. Uridine:PMA_DPI_15m	ns	>0.9999
Aspartic acid:PMA_6AN_15m vs. Uridine:PMA_AA_15m	ns	>0.9999
Aspartic acid:PMA_6AN_15m vs. Uridine:PMA_IAA_15m	ns	0.9953
Aspartic acid:PMA_6AN_15m vs. UMP:PMA_15m	ns	>0.9999
Aspartic acid:PMA_6AN_15m vs. UMP:PMA_2DG_15m	ns	>0.9999
Aspartic acid:PMA_6AN_15m vs. UMP:PMA_6AN_15m	ns	0.9313
Aspartic acid:PMA_6AN_15m vs. UMP:PMA_DPI_15m	ns	>0.9999
Aspartic acid:PMA_6AN_15m vs. UMP:PMA_AA_15m	ns	0.9814
Aspartic acid:PMA_6AN_15m vs. UMP:PMA_IAA_15m	ns	>0.9999
Aspartic acid:PMA_6AN_15m vs. UDP:PMA_15m	ns	>0.9999
Aspartic acid:PMA_6AN_15m vs. UDP:PMA_2DG_15m	ns	>0.9999
Aspartic acid:PMA_6AN_15m vs. UDP:PMA_6AN_15m	ns	0.9391
Aspartic acid:PMA_6AN_15m vs. UDP:PMA_DPI_15m	ns	>0.9999
Aspartic acid:PMA_6AN_15m vs. UDP:PMA_AA_15m	ns	0.984
Aspartic acid:PMA_6AN_15m vs. UDP:PMA_IAA_15m	ns	>0.9999
Aspartic acid:PMA_6AN_15m vs. UTP:PMA_15m	ns	0.8206

Aspartic acid:PMA_6AN_15m vs. UTP:PMA_2DG_15m	ns	>0.9999
Aspartic acid:PMA_6AN_15m vs. UTP:PMA_6AN_15m	ns	>0.9999
Aspartic acid:PMA_6AN_15m vs. UTP:PMA_DPI_15m	ns	0.9893
Aspartic acid:PMA_6AN_15m vs. UTP:PMA_AA_15m	ns	>0.9999
Aspartic acid:PMA_6AN_15m vs. UTP:PMA_IAA_15m	**	0.0015
Aspartic acid:PMA_DPI_15m vs. Aspartic acid:PMA_AA_15m	ns	0.5731
Aspartic acid:PMA_DPI_15m vs. Aspartic acid:PMA_IAA_15m	***	0.0004
Aspartic acid:PMA_DPI_15m vs. AMP:PMA_15m	ns	>0.9999
Aspartic acid:PMA_DPI_15m vs. AMP:PMA_2DG_15m	ns	0.9971
Aspartic acid:PMA_DPI_15m vs. AMP:PMA_6AN_15m	ns	0.917
Aspartic acid:PMA_DPI_15m vs. AMP:PMA_DPI_15m	ns	>0.9999
Aspartic acid:PMA_DPI_15m vs. AMP:PMA_AA_15m	ns	0.8462
Aspartic acid:PMA_DPI_15m vs. AMP:PMA_IAA_15m	ns	>0.9999
Aspartic acid:PMA_DPI_15m vs. ADP:PMA_15m	ns	>0.9999
Aspartic acid:PMA_DPI_15m vs. ADP:PMA_2DG_15m	ns	>0.9999
Aspartic acid:PMA_DPI_15m vs. ADP:PMA_6AN_15m	ns	0.9924
Aspartic acid:PMA_DPI_15m vs. ADP:PMA_DPI_15m	ns	>0.9999
Aspartic acid:PMA_DPI_15m vs. ADP:PMA_AA_15m	ns	0.9772
Aspartic acid:PMA_DPI_15m vs. ADP:PMA_IAA_15m	ns	>0.9999
Aspartic acid:PMA_DPI_15m vs. ATP:PMA_15m	ns	>0.9999
Aspartic acid:PMA_DPI_15m vs. ATP:PMA_2DG_15m	ns	>0.9999
Aspartic acid:PMA_DPI_15m vs. ATP:PMA_6AN_15m	ns	>0.9999
Aspartic acid:PMA_DPI_15m vs. ATP:PMA_DPI_15m	ns	>0.9999
Aspartic acid:PMA_DPI_15m vs. ATP:PMA_AA_15m	ns	>0.9999
Aspartic acid:PMA_DPI_15m vs. ATP:PMA_IAA_15m	ns	0.0708
Aspartic acid:PMA_DPI_15m vs. Xanthine:PMA_15m	ns	>0.9999
Aspartic acid:PMA_DPI_15m vs. Xanthine:PMA_2DG_15m	ns	>0.9999
Aspartic acid:PMA_DPI_15m vs. Xanthine:PMA_6AN_15m	ns	>0.9999
Aspartic acid:PMA_DPI_15m vs. Xanthine:PMA_DPI_15m	ns	>0.9999
Aspartic acid:PMA_DPI_15m vs. Xanthine:PMA_AA_15m	ns	>0.9999
Aspartic acid:PMA_DPI_15m vs. CMP:PMA_15m	ns	>0.9999
Aspartic acid:PMA_DPI_15m vs. CMP:PMA_2DG_15m	ns	0.9903
Aspartic acid:PMA_DPI_15m vs. CMP:PMA_6AN_15m	ns	0.8518
Aspartic acid:PMA_DPI_15m vs. CMP:PMA_DPI_15m	ns	>0.9999
Aspartic acid:PMA_DPI_15m vs. CMP:PMA_AA_15m	ns	0.7575
Aspartic acid:PMA_DPI_15m vs. CMP:PMA_IAA_15m	ns	>0.9999
Aspartic acid:PMA_DPI_15m vs. CDP:PMA_15m	ns	>0.9999
Aspartic acid:PMA_DPI_15m vs. CDP:PMA_2DG_15m	ns	0.7796
Aspartic acid:PMA_DPI_15m vs. CDP:PMA_6AN_15m	ns	0.4007
Aspartic acid:PMA_DPI_15m vs. CDP:PMA_DPI_15m	ns	0.9978
Aspartic acid:PMA_DPI_15m vs. CDP:PMA_AA_15m	ns	0.2979
Aspartic acid:PMA_DPI_15m vs. CDP:PMA_IAA_15m	ns	>0.9999
Aspartic acid:PMA_DPI_15m vs. CTP:PMA_15m	ns	>0.9999
Aspartic acid:PMA_DPI_15m vs. CTP:PMA_2DG_15m	ns	>0.9999
Aspartic acid:PMA_DPI_15m vs. CTP:PMA_6AN_15m	ns	>0.9999
Aspartic acid:PMA_DPI_15m vs. CTP:PMA_DPI_15m	ns	>0.9999
Aspartic acid:PMA_DPI_15m vs. CTP:PMA_AA_15m	ns	>0.9999

Aspartic acid:PMA_DPI_15m vs. CTP:PMA_IAA_15m	ns	0.3861
Aspartic acid:PMA_DPI_15m vs. Uridine:PMA_15m	ns	>0.9999
Aspartic acid:PMA_DPI_15m vs. Uridine:PMA_2DG_15m	ns	0.9584
Aspartic acid:PMA_DPI_15m vs. Uridine:PMA_6AN_15m	ns	0.7084
Aspartic acid:PMA_DPI_15m vs. Uridine:PMA_DPI_15m	ns	>0.9999
Aspartic acid:PMA_DPI_15m vs. Uridine:PMA_AA_15m	ns	0.591
Aspartic acid:PMA_DPI_15m vs. Uridine:PMA_IAA_15m	ns	>0.9999
Aspartic acid:PMA_DPI_15m vs. UMP:PMA_15m	ns	>0.9999
Aspartic acid:PMA_DPI_15m vs. UMP:PMA_2DG_15m	ns	0.4541
Aspartic acid:PMA_DPI_15m vs. UMP:PMA_6AN_15m	ns	0.1578
Aspartic acid:PMA_DPI_15m vs. UMP:PMA_DPI_15m	ns	0.9313
Aspartic acid:PMA_DPI_15m vs. UMP:PMA_AA_15m	ns	0.1058
Aspartic acid:PMA_DPI_15m vs. UMP:PMA_IAA_15m	ns	>0.9999
Aspartic acid:PMA_DPI_15m vs. UDP:PMA_15m	ns	>0.9999
Aspartic acid:PMA_DPI_15m vs. UDP:PMA_2DG_15m	ns	0.4706
Aspartic acid:PMA_DPI_15m vs. UDP:PMA_6AN_15m	ns	0.1665
Aspartic acid:PMA_DPI_15m vs. UDP:PMA_DPI_15m	ns	0.9391
Aspartic acid:PMA_DPI_15m vs. UDP:PMA_AA_15m	ns	0.1122
Aspartic acid:PMA_DPI_15m vs. UDP:PMA_IAA_15m	ns	>0.9999
Aspartic acid:PMA_DPI_15m vs. UTP:PMA_15m	ns	>0.9999
Aspartic acid:PMA_DPI_15m vs. UTP:PMA_2DG_15m	ns	>0.9999
Aspartic acid:PMA_DPI_15m vs. UTP:PMA_6AN_15m	ns	>0.9999
Aspartic acid:PMA_DPI_15m vs. UTP:PMA_DPI_15m	ns	>0.9999
Aspartic acid:PMA_DPI_15m vs. UTP:PMA_AA_15m	ns	>0.9999
Aspartic acid:PMA_DPI_15m vs. UTP:PMA_IAA_15m	ns	0.2497
Aspartic acid:PMA_AA_15m vs. Aspartic acid:PMA_IAA_15m	****	<0.0001
Aspartic acid:PMA_AA_15m vs. AMP:PMA_15m	ns	>0.9999
Aspartic acid:PMA_AA_15m vs. AMP:PMA_2DG_15m	ns	>0.9999
Aspartic acid:PMA_AA_15m vs. AMP:PMA_6AN_15m	ns	>0.9999
Aspartic acid:PMA_AA_15m vs. AMP:PMA_DPI_15m	ns	>0.9999
Aspartic acid:PMA_AA_15m vs. AMP:PMA_AA_15m	ns	>0.9999
Aspartic acid:PMA_AA_15m vs. AMP:PMA_IAA_15m	ns	0.8897
Aspartic acid:PMA_AA_15m vs. ADP:PMA_15m	ns	>0.9999
Aspartic acid:PMA_AA_15m vs. ADP:PMA_2DG_15m	ns	>0.9999
Aspartic acid:PMA_AA_15m vs. ADP:PMA_6AN_15m	ns	>0.9999
Aspartic acid:PMA_AA_15m vs. ADP:PMA_DPI_15m	ns	>0.9999
Aspartic acid:PMA_AA_15m vs. ADP:PMA_AA_15m	ns	>0.9999
Aspartic acid:PMA_AA_15m vs. ADP:PMA_IAA_15m	ns	0.6394
Aspartic acid:PMA_AA_15m vs. ATP:PMA_15m	ns	0.3461
Aspartic acid:PMA_AA_15m vs. ATP:PMA_2DG_15m	ns	>0.9999
Aspartic acid:PMA_AA_15m vs. ATP:PMA_6AN_15m	ns	>0.9999
Aspartic acid:PMA_AA_15m vs. ATP:PMA_DPI_15m	ns	0.7554
Aspartic acid:PMA_AA_15m vs. ATP:PMA_AA_15m	ns	>0.9999
Aspartic acid:PMA_AA_15m vs. ATP:PMA_IAA_15m	***	0.0001
Aspartic acid:PMA_AA_15m vs. Xanthine:PMA_15m	ns	0.9948
Aspartic acid:PMA_AA_15m vs. Xanthine:PMA_2DG_15m	ns	>0.9999
Aspartic acid:PMA_AA_15m vs. Xanthine:PMA_6AN_15m	ns	>0.9999

Aspartic acid:PMA_AA_15m vs. Xanthine:PMA_DPI_15m	ns	>0.9999
Aspartic acid:PMA_AA_15m vs. Xanthine:PMA_AA_15m	ns	>0.9999
Aspartic acid:PMA_AA_15m vs. CMP:PMA_15m	ns	>0.9999
Aspartic acid:PMA_AA_15m vs. CMP:PMA_2DG_15m	ns	>0.9999
Aspartic acid:PMA_AA_15m vs. CMP:PMA_6AN_15m	ns	>0.9999
Aspartic acid:PMA_AA_15m vs. CMP:PMA_DPI_15m	ns	>0.9999
Aspartic acid:PMA_AA_15m vs. CMP:PMA_AA_15m	ns	>0.9999
Aspartic acid:PMA_AA_15m vs. CMP:PMA_IAA_15m	ns	0.9424
Aspartic acid:PMA_AA_15m vs. CDP:PMA_15m	ns	>0.9999
Aspartic acid:PMA_AA_15m vs. CDP:PMA_2DG_15m	ns	>0.9999
Aspartic acid:PMA_AA_15m vs. CDP:PMA_6AN_15m	ns	>0.9999
Aspartic acid:PMA_AA_15m vs. CDP:PMA_DPI_15m	ns	>0.9999
Aspartic acid:PMA_AA_15m vs. CDP:PMA_AA_15m	ns	0.9978
Aspartic acid:PMA_AA_15m vs. CDP:PMA_IAA_15m	ns	0.9996
Aspartic acid:PMA_AA_15m vs. CTP:PMA_15m	ns	0.856
Aspartic acid:PMA_AA_15m vs. CTP:PMA_2DG_15m	ns	>0.9999
Aspartic acid:PMA_AA_15m vs. CTP:PMA_6AN_15m	ns	>0.9999
Aspartic acid:PMA_AA_15m vs. CTP:PMA_DPI_15m	ns	0.9935
Aspartic acid:PMA_AA_15m vs. CTP:PMA_AA_15m	ns	>0.9999
Aspartic acid:PMA_AA_15m vs. CTP:PMA_IAA_15m	**	0.0019
Aspartic acid:PMA_AA_15m vs. Uridine:PMA_15m	ns	>0.9999
Aspartic acid:PMA_AA_15m vs. Uridine:PMA_2DG_15m	ns	>0.9999
Aspartic acid:PMA_AA_15m vs. Uridine:PMA_6AN_15m	ns	>0.9999
Aspartic acid:PMA_AA_15m vs. Uridine:PMA_DPI_15m	ns	>0.9999
Aspartic acid:PMA_AA_15m vs. Uridine:PMA_AA_15m	ns	>0.9999
Aspartic acid:PMA_AA_15m vs. Uridine:PMA_IAA_15m	ns	0.9847
Aspartic acid:PMA_AA_15m vs. UMP:PMA_15m	ns	>0.9999
Aspartic acid:PMA_AA_15m vs. UMP:PMA_2DG_15m	ns	>0.9999
Aspartic acid:PMA_AA_15m vs. UMP:PMA_6AN_15m	ns	0.9986
Aspartic acid:PMA_AA_15m vs. UMP:PMA_DPI_15m	ns	>0.9999
Aspartic acid:PMA_AA_15m vs. UMP:PMA_AA_15m	ns	0.9313
Aspartic acid:PMA_AA_15m vs. UMP:PMA_IAA_15m	ns	>0.9999
Aspartic acid:PMA_AA_15m vs. UDP:PMA_15m	ns	>0.9999
Aspartic acid:PMA_AA_15m vs. UDP:PMA_2DG_15m	ns	>0.9999
Aspartic acid:PMA_AA_15m vs. UDP:PMA_6AN_15m	ns	0.9989
Aspartic acid:PMA_AA_15m vs. UDP:PMA_DPI_15m	ns	>0.9999
Aspartic acid:PMA_AA_15m vs. UDP:PMA_AA_15m	ns	0.9391
Aspartic acid:PMA_AA_15m vs. UDP:PMA_IAA_15m	ns	>0.9999
Aspartic acid:PMA_AA_15m vs. UTP:PMA_15m	ns	0.7187
Aspartic acid:PMA_AA_15m vs. UTP:PMA_2DG_15m	ns	>0.9999
Aspartic acid:PMA_AA_15m vs. UTP:PMA_6AN_15m	ns	>0.9999
Aspartic acid:PMA_AA_15m vs. UTP:PMA_DPI_15m	ns	0.97
Aspartic acid:PMA_AA_15m vs. UTP:PMA_AA_15m	ns	>0.9999
Aspartic acid:PMA_AA_15m vs. UTP:PMA_IAA_15m	***	0.0008
Aspartic acid:PMA_IAA_15m vs. AMP:PMA_15m	ns	0.3733
Aspartic acid:PMA_IAA_15m vs. AMP:PMA_2DG_15m	**	0.0027
Aspartic acid:PMA_IAA_15m vs. AMP:PMA_6AN_15m	***	0.0004

Aspartic acid:PMA_IAA_15m vs. AMP:PMA_DPI_15m	ns	0.1064
Aspartic acid:PMA_IAA_15m vs. AMP:PMA_AA_15m	***	0.0002
Aspartic acid:PMA_IAA_15m vs. AMP:PMA_IAA_15m	ns	>0.9999
Aspartic acid:PMA_IAA_15m vs. ADP:PMA_15m	ns	0.6716
Aspartic acid:PMA_IAA_15m vs. ADP:PMA_2DG_15m	*	0.0112
Aspartic acid:PMA_IAA_15m vs. ADP:PMA_6AN_15m	**	0.0018
Aspartic acid:PMA_IAA_15m vs. ADP:PMA_DPI_15m	ns	0.2747
Aspartic acid:PMA_IAA_15m vs. ADP:PMA_AA_15m	**	0.001
Aspartic acid:PMA_IAA_15m vs. ADP:PMA_IAA_15m	ns	>0.9999
Aspartic acid:PMA_IAA_15m vs. ATP:PMA_15m	ns	>0.9999
Aspartic acid:PMA_IAA_15m vs. ATP:PMA_2DG_15m	ns	0.9998
Aspartic acid:PMA_IAA_15m vs. ATP:PMA_6AN_15m	ns	0.9763
Aspartic acid:PMA_IAA_15m vs. ATP:PMA_DPI_15m	ns	>0.9999
Aspartic acid:PMA_IAA_15m vs. ATP:PMA_AA_15m	ns	0.9433
Aspartic acid:PMA_IAA_15m vs. ATP:PMA_IAA_15m	ns	>0.9999
Aspartic acid:PMA_IAA_15m vs. Xanthine:PMA_15m	ns	>0.9999
Aspartic acid:PMA_IAA_15m vs. Xanthine:PMA_2DG_15m	ns	0.6969
Aspartic acid:PMA_IAA_15m vs. Xanthine:PMA_6AN_15m	ns	0.3267
Aspartic acid:PMA_IAA_15m vs. Xanthine:PMA_DPI_15m	ns	0.9997
Aspartic acid:PMA_IAA_15m vs. Xanthine:PMA_AA_15m	ns	0.2377
Aspartic acid:PMA_IAA_15m vs. CMP:PMA_15m	ns	0.2811
Aspartic acid:PMA_IAA_15m vs. CMP:PMA_2DG_15m	**	0.0016
Aspartic acid:PMA_IAA_15m vs. CMP:PMA_6AN_15m	***	0.0002
Aspartic acid:PMA_IAA_15m vs. CMP:PMA_DPI_15m	ns	0.0714
Aspartic acid:PMA_IAA_15m vs. CMP:PMA_AA_15m	***	0.0001
Aspartic acid:PMA_IAA_15m vs. CMP:PMA_IAA_15m	ns	>0.9999
Aspartic acid:PMA_IAA_15m vs. CDP:PMA_15m	ns	0.0569
Aspartic acid:PMA_IAA_15m vs. CDP:PMA_2DG_15m	***	0.0001
Aspartic acid:PMA_IAA_15m vs. CDP:PMA_6AN_15m	****	<0.0001
Aspartic acid:PMA_IAA_15m vs. CDP:PMA_DPI_15m	**	0.0097
Aspartic acid:PMA_IAA_15m vs. CDP:PMA_AA_15m	****	<0.0001
Aspartic acid:PMA_IAA_15m vs. CDP:PMA_IAA_15m	ns	0.9978
Aspartic acid:PMA_IAA_15m vs. CTP:PMA_15m	ns	>0.9999
Aspartic acid:PMA_IAA_15m vs. CTP:PMA_2DG_15m	ns	0.9264
Aspartic acid:PMA_IAA_15m vs. CTP:PMA_6AN_15m	ns	0.6249
Aspartic acid:PMA_IAA_15m vs. CTP:PMA_DPI_15m	ns	>0.9999
Aspartic acid:PMA_IAA_15m vs. CTP:PMA_AA_15m	ns	0.505
Aspartic acid:PMA_IAA_15m vs. CTP:PMA_IAA_15m	ns	>0.9999
Aspartic acid:PMA_IAA_15m vs. Uridine:PMA_15m	ns	0.1693
Aspartic acid:PMA_IAA_15m vs. Uridine:PMA_2DG_15m	***	0.0007
Aspartic acid:PMA_IAA_15m vs. Uridine:PMA_6AN_15m	****	<0.0001
Aspartic acid:PMA_IAA_15m vs. Uridine:PMA_DPI_15m	*	0.0367
Aspartic acid:PMA_IAA_15m vs. Uridine:PMA_AA_15m	****	<0.0001
Aspartic acid:PMA_IAA_15m vs. Uridine:PMA_IAA_15m	ns	>0.9999
Aspartic acid:PMA_IAA_15m vs. UMP:PMA_15m	*	0.0144
Aspartic acid:PMA_IAA_15m vs. UMP:PMA_2DG_15m	****	<0.0001
Aspartic acid:PMA_IAA_15m vs. UMP:PMA_6AN_15m	****	<0.0001

Aspartic acid:PMA_IAA_15m vs. UMP:PMA_DPI_15m	**	0.002
Aspartic acid:PMA_IAA_15m vs. UMP:PMA_AA_15m	****	<0.0001
Aspartic acid:PMA_IAA_15m vs. UMP:PMA_IAA_15m	ns	0.9313
Aspartic acid:PMA_IAA_15m vs. UDP:PMA_15m	*	0.0155
Aspartic acid:PMA_IAA_15m vs. UDP:PMA_2DG_15m	****	<0.0001
Aspartic acid:PMA_IAA_15m vs. UDP:PMA_6AN_15m	****	<0.0001
Aspartic acid:PMA_IAA_15m vs. UDP:PMA_DPI_15m	**	0.0022
Aspartic acid:PMA_IAA_15m vs. UDP:PMA_AA_15m	****	<0.0001
Aspartic acid:PMA_IAA_15m vs. UDP:PMA_IAA_15m	ns	0.9391
Aspartic acid:PMA_IAA_15m vs. UTP:PMA_15m	ns	>0.9999
Aspartic acid:PMA_IAA_15m vs. UTP:PMA_2DG_15m	ns	0.9777
Aspartic acid:PMA_IAA_15m vs. UTP:PMA_6AN_15m	ns	0.7819
Aspartic acid:PMA_IAA_15m vs. UTP:PMA_DPI_15m	ns	>0.9999
Aspartic acid:PMA_IAA_15m vs. UTP:PMA_AA_15m	ns	0.6732
Aspartic acid:PMA_IAA_15m vs. UTP:PMA_IAA_15m	ns	>0.9999
AMP:PMA_15m vs. AMP:PMA_2DG_15m	ns	0.7475
AMP:PMA_15m vs. AMP:PMA_6AN_15m	ns	0.1037
AMP:PMA_15m vs. AMP:PMA_DPI_15m	ns	>0.9999
AMP:PMA_15m vs. AMP:PMA_AA_15m	*	0.0409
AMP:PMA_15m vs. AMP:PMA_IAA_15m	*	0.0236
AMP:PMA_15m vs. ADP:PMA_15m	ns	>0.9999
AMP:PMA_15m vs. ADP:PMA_2DG_15m	ns	>0.9999
AMP:PMA_15m vs. ADP:PMA_6AN_15m	ns	>0.9999
AMP:PMA_15m vs. ADP:PMA_DPI_15m	ns	>0.9999
AMP:PMA_15m vs. ADP:PMA_AA_15m	ns	>0.9999
AMP:PMA_15m vs. ADP:PMA_IAA_15m	ns	0.9878
AMP:PMA_15m vs. ATP:PMA_15m	ns	0.6011
AMP:PMA_15m vs. ATP:PMA_2DG_15m	ns	>0.9999
AMP:PMA_15m vs. ATP:PMA_6AN_15m	ns	>0.9999
AMP:PMA_15m vs. ATP:PMA_DPI_15m	ns	0.9967
AMP:PMA_15m vs. ATP:PMA_AA_15m	ns	>0.9999
AMP:PMA_15m vs. ATP:PMA_IAA_15m	**	0.0026
AMP:PMA_15m vs. Xanthine:PMA_15m	ns	>0.9999
AMP:PMA_15m vs. Xanthine:PMA_2DG_15m	ns	>0.9999
AMP:PMA_15m vs. Xanthine:PMA_6AN_15m	ns	>0.9999
AMP:PMA_15m vs. Xanthine:PMA_DPI_15m	ns	>0.9999
AMP:PMA_15m vs. Xanthine:PMA_AA_15m	ns	>0.9999
AMP:PMA_15m vs. CMP:PMA_15m	ns	>0.9999
AMP:PMA_15m vs. CMP:PMA_2DG_15m	ns	>0.9999
AMP:PMA_15m vs. CMP:PMA_6AN_15m	ns	>0.9999
AMP:PMA_15m vs. CMP:PMA_DPI_15m	ns	>0.9999
AMP:PMA_15m vs. CMP:PMA_AA_15m	ns	0.9996
AMP:PMA_15m vs. CMP:PMA_IAA_15m	ns	>0.9999
AMP:PMA_15m vs. CDP:PMA_15m	ns	>0.9999
AMP:PMA_15m vs. CDP:PMA_2DG_15m	ns	0.9997
AMP:PMA_15m vs. CDP:PMA_6AN_15m	ns	0.9741
AMP:PMA_15m vs. CDP:PMA_DPI_15m	ns	>0.9999

AMP:PMA_15m vs. CDP:PMA_AA_15m	ns	0.939
AMP:PMA_15m vs. CDP:PMA_IAA_15m	ns	>0.9999
AMP:PMA_15m vs. CTP:PMA_15m	ns	0.9853
AMP:PMA_15m vs. CTP:PMA_2DG_15m	ns	>0.9999
AMP:PMA_15m vs. CTP:PMA_6AN_15m	ns	>0.9999
AMP:PMA_15m vs. CTP:PMA_DPI_15m	ns	>0.9999
AMP:PMA_15m vs. CTP:PMA_AA_15m	ns	>0.9999
AMP:PMA_15m vs. CTP:PMA_IAA_15m	*	0.0308
AMP:PMA_15m vs. Uridine:PMA_15m	ns	>0.9999
AMP:PMA_15m vs. Uridine:PMA_2DG_15m	ns	>0.9999
AMP:PMA_15m vs. Uridine:PMA_6AN_15m	ns	0.9991
AMP:PMA_15m vs. Uridine:PMA_DPI_15m	ns	>0.9999
AMP:PMA_15m vs. Uridine:PMA_AA_15m	ns	0.9959
AMP:PMA_15m vs. Uridine:PMA_IAA_15m	ns	>0.9999
AMP:PMA_15m vs. UMP:PMA_15m	ns	>0.9999
AMP:PMA_15m vs. UMP:PMA_2DG_15m	ns	0.9839
AMP:PMA_15m vs. UMP:PMA_6AN_15m	ns	0.8118
AMP:PMA_15m vs. UMP:PMA_DPI_15m	ns	>0.9999
AMP:PMA_15m vs. UMP:PMA_AA_15m	ns	0.708
AMP:PMA_15m vs. UMP:PMA_IAA_15m	ns	>0.9999
AMP:PMA_15m vs. UDP:PMA_15m	ns	>0.9999
AMP:PMA_15m vs. UDP:PMA_2DG_15m	ns	0.9862
AMP:PMA_15m vs. UDP:PMA_6AN_15m	ns	0.8248
AMP:PMA_15m vs. UDP:PMA_DPI_15m	ns	>0.9999
AMP:PMA_15m vs. UDP:PMA_AA_15m	ns	0.7238
AMP:PMA_15m vs. UDP:PMA_IAA_15m	ns	>0.9999
AMP:PMA_15m vs. UTP:PMA_15m	ns	0.9366
AMP:PMA_15m vs. UTP:PMA_2DG_15m	ns	>0.9999
AMP:PMA_15m vs. UTP:PMA_6AN_15m	ns	>0.9999
AMP:PMA_15m vs. UTP:PMA_DPI_15m	ns	>0.9999
AMP:PMA_15m vs. UTP:PMA_AA_15m	ns	>0.9999
AMP:PMA_15m vs. UTP:PMA_IAA_15m	*	0.0152
AMP:PMA_2DG_15m vs. AMP:PMA_6AN_15m	ns	>0.9999
AMP:PMA_2DG_15m vs. AMP:PMA_DPI_15m	ns	>0.9999
AMP:PMA_2DG_15m vs. AMP:PMA_AA_15m	ns	>0.9999
AMP:PMA_2DG_15m vs. AMP:PMA_IAA_15m	****	<0.0001
AMP:PMA_2DG_15m vs. ADP:PMA_15m	ns	>0.9999
AMP:PMA_2DG_15m vs. ADP:PMA_2DG_15m	ns	>0.9999
AMP:PMA_2DG_15m vs. ADP:PMA_6AN_15m	ns	>0.9999
AMP:PMA_2DG_15m vs. ADP:PMA_DPI_15m	ns	>0.9999
AMP:PMA_2DG_15m vs. ADP:PMA_AA_15m	ns	>0.9999
AMP:PMA_2DG_15m vs. ADP:PMA_IAA_15m	ns	0.1127
AMP:PMA_2DG_15m vs. ATP:PMA_15m	*	0.0359
AMP:PMA_2DG_15m vs. ATP:PMA_2DG_15m	ns	0.6011
AMP:PMA_2DG_15m vs. ATP:PMA_6AN_15m	ns	0.9952
AMP:PMA_2DG_15m vs. ATP:PMA_DPI_15m	ns	0.1675
AMP:PMA_2DG_15m vs. ATP:PMA_AA_15m	ns	0.9989

AMP:PMA_2DG_15m vs. ATP:PMA_IAA_15m	****	<0.0001
AMP:PMA_2DG_15m vs. Xanthine:PMA_15m	ns	0.6804
AMP:PMA_2DG_15m vs. Xanthine:PMA_2DG_15m	ns	>0.9999
AMP:PMA_2DG_15m vs. Xanthine:PMA_6AN_15m	ns	>0.9999
AMP:PMA_2DG_15m vs. Xanthine:PMA_DPI_15m	ns	0.9546
AMP:PMA_2DG_15m vs. Xanthine:PMA_AA_15m	ns	>0.9999
AMP:PMA_2DG_15m vs. CMP:PMA_15m	ns	>0.9999
AMP:PMA_2DG_15m vs. CMP:PMA_2DG_15m	ns	>0.9999
AMP:PMA_2DG_15m vs. CMP:PMA_6AN_15m	ns	>0.9999
AMP:PMA_2DG_15m vs. CMP:PMA_DPI_15m	ns	>0.9999
AMP:PMA_2DG_15m vs. CMP:PMA_AA_15m	ns	>0.9999
AMP:PMA_2DG_15m vs. CMP:PMA_IAA_15m	ns	0.3807
AMP:PMA_2DG_15m vs. CDP:PMA_15m	ns	>0.9999
AMP:PMA_2DG_15m vs. CDP:PMA_2DG_15m	ns	>0.9999
AMP:PMA_2DG_15m vs. CDP:PMA_6AN_15m	ns	>0.9999
AMP:PMA_2DG_15m vs. CDP:PMA_DPI_15m	ns	>0.9999
AMP:PMA_2DG_15m vs. CDP:PMA_AA_15m	ns	>0.9999
AMP:PMA_2DG_15m vs. CDP:PMA_IAA_15m	ns	0.835
AMP:PMA_2DG_15m vs. CTP:PMA_15m	ns	0.2461
AMP:PMA_2DG_15m vs. CTP:PMA_2DG_15m	ns	0.9853
AMP:PMA_2DG_15m vs. CTP:PMA_6AN_15m	ns	>0.9999
AMP:PMA_2DG_15m vs. CTP:PMA_DPI_15m	ns	0.6341
AMP:PMA_2DG_15m vs. CTP:PMA_AA_15m	ns	>0.9999
AMP:PMA_2DG_15m vs. CTP:PMA_IAA_15m	****	<0.0001
AMP:PMA_2DG_15m vs. Uridine:PMA_15m	ns	>0.9999
AMP:PMA_2DG_15m vs. Uridine:PMA_2DG_15m	ns	>0.9999
AMP:PMA_2DG_15m vs. Uridine:PMA_6AN_15m	ns	>0.9999
AMP:PMA_2DG_15m vs. Uridine:PMA_DPI_15m	ns	>0.9999
AMP:PMA_2DG_15m vs. Uridine:PMA_AA_15m	ns	>0.9999
AMP:PMA_2DG_15m vs. Uridine:PMA_IAA_15m	ns	0.548
AMP:PMA_2DG_15m vs. UMP:PMA_15m	ns	>0.9999
AMP:PMA_2DG_15m vs. UMP:PMA_2DG_15m	ns	>0.9999
AMP:PMA_2DG_15m vs. UMP:PMA_6AN_15m	ns	>0.9999
AMP:PMA_2DG_15m vs. UMP:PMA_DPI_15m	ns	>0.9999
AMP:PMA_2DG_15m vs. UMP:PMA_AA_15m	ns	>0.9999
AMP:PMA_2DG_15m vs. UMP:PMA_IAA_15m	ns	0.9797
AMP:PMA_2DG_15m vs. UDP:PMA_15m	ns	>0.9999
AMP:PMA_2DG_15m vs. UDP:PMA_2DG_15m	ns	>0.9999
AMP:PMA_2DG_15m vs. UDP:PMA_6AN_15m	ns	>0.9999
AMP:PMA_2DG_15m vs. UDP:PMA_DPI_15m	ns	>0.9999
AMP:PMA_2DG_15m vs. UDP:PMA_AA_15m	ns	>0.9999
AMP:PMA_2DG_15m vs. UDP:PMA_IAA_15m	ns	0.9766
AMP:PMA_2DG_15m vs. UTP:PMA_15m	ns	0.1471
AMP:PMA_2DG_15m vs. UTP:PMA_2DG_15m	ns	0.9366
AMP:PMA_2DG_15m vs. UTP:PMA_6AN_15m	ns	>0.9999
AMP:PMA_2DG_15m vs. UTP:PMA_DPI_15m	ns	0.4651
AMP:PMA_2DG_15m vs. UTP:PMA_AA_15m	ns	>0.9999

AMP:PMA_2DG_15m vs. UTP:PMA_IAA_15m	****	<0.0001
AMP:PMA_6AN_15m vs. AMP:PMA_DPI_15m	ns	0.8059
AMP:PMA_6AN_15m vs. AMP:PMA_AA_15m	ns	>0.9999
AMP:PMA_6AN_15m vs. AMP:PMA_IAA_15m	****	<0.0001
AMP:PMA_6AN_15m vs. ADP:PMA_15m	ns	0.9988
AMP:PMA_6AN_15m vs. ADP:PMA_2DG_15m	ns	>0.9999
AMP:PMA_6AN_15m vs. ADP:PMA_6AN_15m	ns	>0.9999
AMP:PMA_6AN_15m vs. ADP:PMA_DPI_15m	ns	>0.9999
AMP:PMA_6AN_15m vs. ADP:PMA_AA_15m	ns	>0.9999
AMP:PMA_6AN_15m vs. ADP:PMA_IAA_15m	*	0.0253
AMP:PMA_6AN_15m vs. ATP:PMA_15m	**	0.0066
AMP:PMA_6AN_15m vs. ATP:PMA_2DG_15m	ns	0.5589
AMP:PMA_6AN_15m vs. ATP:PMA_6AN_15m	ns	0.6011
AMP:PMA_6AN_15m vs. ATP:PMA_DPI_15m	*	0.041
AMP:PMA_6AN_15m vs. ATP:PMA_AA_15m	ns	0.9483
AMP:PMA_6AN_15m vs. ATP:PMA_IAA_15m	****	<0.0001
AMP:PMA_6AN_15m vs. Xanthine:PMA_15m	ns	0.3186
AMP:PMA_6AN_15m vs. Xanthine:PMA_2DG_15m	ns	0.9997
AMP:PMA_6AN_15m vs. Xanthine:PMA_6AN_15m	ns	>0.9999
AMP:PMA_6AN_15m vs. Xanthine:PMA_DPI_15m	ns	0.7108
AMP:PMA_6AN_15m vs. Xanthine:PMA_AA_15m	ns	>0.9999
AMP:PMA_6AN_15m vs. CMP:PMA_15m	ns	>0.9999
AMP:PMA_6AN_15m vs. CMP:PMA_2DG_15m	ns	>0.9999
AMP:PMA_6AN_15m vs. CMP:PMA_6AN_15m	ns	>0.9999
AMP:PMA_6AN_15m vs. CMP:PMA_DPI_15m	ns	>0.9999
AMP:PMA_6AN_15m vs. CMP:PMA_AA_15m	ns	>0.9999
AMP:PMA_6AN_15m vs. CMP:PMA_IAA_15m	ns	0.1225
AMP:PMA_6AN_15m vs. CDP:PMA_15m	ns	>0.9999
AMP:PMA_6AN_15m vs. CDP:PMA_2DG_15m	ns	>0.9999
AMP:PMA_6AN_15m vs. CDP:PMA_6AN_15m	ns	>0.9999
AMP:PMA_6AN_15m vs. CDP:PMA_DPI_15m	ns	>0.9999
AMP:PMA_6AN_15m vs. CDP:PMA_AA_15m	ns	>0.9999
AMP:PMA_6AN_15m vs. CDP:PMA_IAA_15m	ns	0.4691
AMP:PMA_6AN_15m vs. CTP:PMA_15m	ns	0.0672
AMP:PMA_6AN_15m vs. CTP:PMA_2DG_15m	ns	0.9613
AMP:PMA_6AN_15m vs. CTP:PMA_6AN_15m	ns	0.9853
AMP:PMA_6AN_15m vs. CTP:PMA_DPI_15m	ns	0.27
AMP:PMA_6AN_15m vs. CTP:PMA_AA_15m	ns	>0.9999
AMP:PMA_6AN_15m vs. CTP:PMA_IAA_15m	****	<0.0001
AMP:PMA_6AN_15m vs. Uridine:PMA_15m	ns	>0.9999
AMP:PMA_6AN_15m vs. Uridine:PMA_2DG_15m	ns	>0.9999
AMP:PMA_6AN_15m vs. Uridine:PMA_6AN_15m	ns	>0.9999
AMP:PMA_6AN_15m vs. Uridine:PMA_DPI_15m	ns	>0.9999
AMP:PMA_6AN_15m vs. Uridine:PMA_AA_15m	ns	>0.9999
AMP:PMA_6AN_15m vs. Uridine:PMA_IAA_15m	ns	0.2123
AMP:PMA_6AN_15m vs. UMP:PMA_15m	ns	>0.9999
AMP:PMA_6AN_15m vs. UMP:PMA_2DG_15m	ns	>0.9999

AMP:PMA_6AN_15m vs. UMP:PMA_6AN_15m	ns	>0.9999
AMP:PMA_6AN_15m vs. UMP:PMA_DPI_15m	ns	>0.9999
AMP:PMA_6AN_15m vs. UMP:PMA_AA_15m	ns	>0.9999
AMP:PMA_6AN_15m vs. UMP:PMA_IAA_15m	ns	0.7914
AMP:PMA_6AN_15m vs. UDP:PMA_15m	ns	>0.9999
AMP:PMA_6AN_15m vs. UDP:PMA_2DG_15m	ns	>0.9999
AMP:PMA_6AN_15m vs. UDP:PMA_6AN_15m	ns	>0.9999
AMP:PMA_6AN_15m vs. UDP:PMA_DPI_15m	ns	>0.9999
AMP:PMA_6AN_15m vs. UDP:PMA_AA_15m	ns	>0.9999
AMP:PMA_6AN_15m vs. UDP:PMA_IAA_15m	ns	0.7773
AMP:PMA_6AN_15m vs. UTP:PMA_15m	*	0.0349
AMP:PMA_6AN_15m vs. UTP:PMA_2DG_15m	ns	0.89
AMP:PMA_6AN_15m vs. UTP:PMA_6AN_15m	ns	0.9366
AMP:PMA_6AN_15m vs. UTP:PMA_DPI_15m	ns	0.1636
AMP:PMA_6AN_15m vs. UTP:PMA_AA_15m	ns	0.9988
AMP:PMA_6AN_15m vs. UTP:PMA_IAA_15m	****	<0.0001
AMP:PMA_DPI_15m vs. AMP:PMA_AA_15m	ns	0.5731
AMP:PMA_DPI_15m vs. AMP:PMA_IAA_15m	***	0.0004
AMP:PMA_DPI_15m vs. ADP:PMA_15m	ns	>0.9999
AMP:PMA_DPI_15m vs. ADP:PMA_2DG_15m	ns	>0.9999
AMP:PMA_DPI_15m vs. ADP:PMA_6AN_15m	ns	>0.9999
AMP:PMA_DPI_15m vs. ADP:PMA_DPI_15m	ns	>0.9999
AMP:PMA_DPI_15m vs. ADP:PMA_AA_15m	ns	>0.9999
AMP:PMA_DPI_15m vs. ADP:PMA_IAA_15m	ns	0.8108
AMP:PMA_DPI_15m vs. ATP:PMA_15m	ns	0.5255
AMP:PMA_DPI_15m vs. ATP:PMA_2DG_15m	ns	>0.9999
AMP:PMA_DPI_15m vs. ATP:PMA_6AN_15m	ns	>0.9999
AMP:PMA_DPI_15m vs. ATP:PMA_DPI_15m	ns	0.6011
AMP:PMA_DPI_15m vs. ATP:PMA_AA_15m	ns	>0.9999
AMP:PMA_DPI_15m vs. ATP:PMA_IAA_15m	***	0.0003
AMP:PMA_DPI_15m vs. Xanthine:PMA_15m	ns	0.9995
AMP:PMA_DPI_15m vs. Xanthine:PMA_2DG_15m	ns	>0.9999
AMP:PMA_DPI_15m vs. Xanthine:PMA_6AN_15m	ns	>0.9999
AMP:PMA_DPI_15m vs. Xanthine:PMA_DPI_15m	ns	>0.9999
AMP:PMA_DPI_15m vs. Xanthine:PMA_AA_15m	ns	>0.9999
AMP:PMA_DPI_15m vs. CMP:PMA_15m	ns	>0.9999
AMP:PMA_DPI_15m vs. CMP:PMA_2DG_15m	ns	>0.9999
AMP:PMA_DPI_15m vs. CMP:PMA_6AN_15m	ns	>0.9999
AMP:PMA_DPI_15m vs. CMP:PMA_DPI_15m	ns	>0.9999
AMP:PMA_DPI_15m vs. CMP:PMA_AA_15m	ns	>0.9999
AMP:PMA_DPI_15m vs. CMP:PMA_IAA_15m	ns	0.9868
AMP:PMA_DPI_15m vs. CDP:PMA_15m	ns	>0.9999
AMP:PMA_DPI_15m vs. CDP:PMA_2DG_15m	ns	>0.9999
AMP:PMA_DPI_15m vs. CDP:PMA_6AN_15m	ns	0.9998
AMP:PMA_DPI_15m vs. CDP:PMA_DPI_15m	ns	>0.9999
AMP:PMA_DPI_15m vs. CDP:PMA_AA_15m	ns	0.999
AMP:PMA_DPI_15m vs. CDP:PMA_IAA_15m	ns	>0.9999

AMP:PMA_DPI_15m vs. CTP:PMA_15m	ns	0.9512
AMP:PMA_DPI_15m vs. CTP:PMA_2DG_15m	ns	>0.9999
AMP:PMA_DPI_15m vs. CTP:PMA_6AN_15m	ns	>0.9999
AMP:PMA_DPI_15m vs. CTP:PMA_DPI_15m	ns	0.9853
AMP:PMA_DPI_15m vs. CTP:PMA_AA_15m	ns	>0.9999
AMP:PMA_DPI_15m vs. CTP:PMA_IAA_15m	**	0.0048
AMP:PMA_DPI_15m vs. Uridine:PMA_15m	ns	>0.9999
AMP:PMA_DPI_15m vs. Uridine:PMA_2DG_15m	ns	>0.9999
AMP:PMA_DPI_15m vs. Uridine:PMA_6AN_15m	ns	>0.9999
AMP:PMA_DPI_15m vs. Uridine:PMA_DPI_15m	ns	>0.9999
AMP:PMA_DPI_15m vs. Uridine:PMA_AA_15m	ns	>0.9999
AMP:PMA_DPI_15m vs. Uridine:PMA_IAA_15m	ns	0.998
AMP:PMA_DPI_15m vs. UMP:PMA_15m	ns	>0.9999
AMP:PMA_DPI_15m vs. UMP:PMA_2DG_15m	ns	>0.9999
AMP:PMA_DPI_15m vs. UMP:PMA_6AN_15m	ns	0.9881
AMP:PMA_DPI_15m vs. UMP:PMA_DPI_15m	ns	>0.9999
AMP:PMA_DPI_15m vs. UMP:PMA_AA_15m	ns	0.9673
AMP:PMA_DPI_15m vs. UMP:PMA_IAA_15m	ns	>0.9999
AMP:PMA_DPI_15m vs. UDP:PMA_15m	ns	>0.9999
AMP:PMA_DPI_15m vs. UDP:PMA_2DG_15m	ns	>0.9999
AMP:PMA_DPI_15m vs. UDP:PMA_6AN_15m	ns	0.9899
AMP:PMA_DPI_15m vs. UDP:PMA_DPI_15m	ns	>0.9999
AMP:PMA_DPI_15m vs. UDP:PMA_AA_15m	ns	0.9713
AMP:PMA_DPI_15m vs. UDP:PMA_IAA_15m	ns	>0.9999
AMP:PMA_DPI_15m vs. UTP:PMA_15m	ns	0.8698
AMP:PMA_DPI_15m vs. UTP:PMA_2DG_15m	ns	>0.9999
AMP:PMA_DPI_15m vs. UTP:PMA_6AN_15m	ns	>0.9999
AMP:PMA_DPI_15m vs. UTP:PMA_DPI_15m	ns	0.9366
AMP:PMA_DPI_15m vs. UTP:PMA_AA_15m	ns	>0.9999
AMP:PMA_DPI_15m vs. UTP:PMA_IAA_15m	**	0.0021
AMP:PMA_AA_15m vs. AMP:PMA_IAA_15m	****	<0.0001
AMP:PMA_AA_15m vs. ADP:PMA_15m	ns	0.9949
AMP:PMA_AA_15m vs. ADP:PMA_2DG_15m	ns	>0.9999
AMP:PMA_AA_15m vs. ADP:PMA_6AN_15m	ns	>0.9999
AMP:PMA_AA_15m vs. ADP:PMA_DPI_15m	ns	>0.9999
AMP:PMA_AA_15m vs. ADP:PMA_AA_15m	ns	>0.9999
AMP:PMA_AA_15m vs. ADP:PMA_IAA_15m	*	0.0153
AMP:PMA_AA_15m vs. ATP:PMA_15m	**	0.0038
AMP:PMA_AA_15m vs. ATP:PMA_2DG_15m	ns	0.4404
AMP:PMA_AA_15m vs. ATP:PMA_6AN_15m	ns	0.8137
AMP:PMA_AA_15m vs. ATP:PMA_DPI_15m	*	0.0252
AMP:PMA_AA_15m vs. ATP:PMA_AA_15m	ns	0.6011
AMP:PMA_AA_15m vs. ATP:PMA_IAA_15m	****	<0.0001
AMP:PMA_AA_15m vs. Xanthine:PMA_15m	ns	0.2326
AMP:PMA_AA_15m vs. Xanthine:PMA_2DG_15m	ns	0.9984
AMP:PMA_AA_15m vs. Xanthine:PMA_6AN_15m	ns	>0.9999
AMP:PMA_AA_15m vs. Xanthine:PMA_DPI_15m	ns	0.5981

AMP:PMA_AA_15m vs. Xanthine:PMA_AA_15m	ns	>0.9999
AMP:PMA_AA_15m vs. CMP:PMA_15m	ns	>0.9999
AMP:PMA_AA_15m vs. CMP:PMA_2DG_15m	ns	>0.9999
AMP:PMA_AA_15m vs. CMP:PMA_6AN_15m	ns	>0.9999
AMP:PMA_AA_15m vs. CMP:PMA_DPI_15m	ns	>0.9999
AMP:PMA_AA_15m vs. CMP:PMA_AA_15m	ns	>0.9999
AMP:PMA_AA_15m vs. CMP:PMA_IAA_15m	ns	0.0807
AMP:PMA_AA_15m vs. CDP:PMA_15m	ns	>0.9999
AMP:PMA_AA_15m vs. CDP:PMA_2DG_15m	ns	>0.9999
AMP:PMA_AA_15m vs. CDP:PMA_6AN_15m	ns	>0.9999
AMP:PMA_AA_15m vs. CDP:PMA_DPI_15m	ns	>0.9999
AMP:PMA_AA_15m vs. CDP:PMA_AA_15m	ns	>0.9999
AMP:PMA_AA_15m vs. CDP:PMA_IAA_15m	ns	0.358
AMP:PMA_AA_15m vs. CTP:PMA_15m	*	0.0424
AMP:PMA_AA_15m vs. CTP:PMA_2DG_15m	ns	0.916
AMP:PMA_AA_15m vs. CTP:PMA_6AN_15m	ns	0.997
AMP:PMA_AA_15m vs. CTP:PMA_DPI_15m	ns	0.1907
AMP:PMA_AA_15m vs. CTP:PMA_AA_15m	ns	0.9853
AMP:PMA_AA_15m vs. CTP:PMA_IAA_15m	****	<0.0001
AMP:PMA_AA_15m vs. Uridine:PMA_15m	ns	>0.9999
AMP:PMA_AA_15m vs. Uridine:PMA_2DG_15m	ns	>0.9999
AMP:PMA_AA_15m vs. Uridine:PMA_6AN_15m	ns	>0.9999
AMP:PMA_AA_15m vs. Uridine:PMA_DPI_15m	ns	>0.9999
AMP:PMA_AA_15m vs. Uridine:PMA_AA_15m	ns	>0.9999
AMP:PMA_AA_15m vs. Uridine:PMA_IAA_15m	ns	0.1464
AMP:PMA_AA_15m vs. UMP:PMA_15m	ns	>0.9999
AMP:PMA_AA_15m vs. UMP:PMA_2DG_15m	ns	>0.9999
AMP:PMA_AA_15m vs. UMP:PMA_6AN_15m	ns	>0.9999
AMP:PMA_AA_15m vs. UMP:PMA_DPI_15m	ns	>0.9999
AMP:PMA_AA_15m vs. UMP:PMA_AA_15m	ns	>0.9999
AMP:PMA_AA_15m vs. UMP:PMA_IAA_15m	ns	0.6842
AMP:PMA_AA_15m vs. UDP:PMA_15m	ns	>0.9999
AMP:PMA_AA_15m vs. UDP:PMA_2DG_15m	ns	>0.9999
AMP:PMA_AA_15m vs. UDP:PMA_6AN_15m	ns	>0.9999
AMP:PMA_AA_15m vs. UDP:PMA_DPI_15m	ns	>0.9999
AMP:PMA_AA_15m vs. UDP:PMA_AA_15m	ns	>0.9999
AMP:PMA_AA_15m vs. UDP:PMA_IAA_15m	ns	0.6678
AMP:PMA_AA_15m vs. UTP:PMA_15m	*	0.0213
AMP:PMA_AA_15m vs. UTP:PMA_2DG_15m	ns	0.808
AMP:PMA_AA_15m vs. UTP:PMA_6AN_15m	ns	0.9832
AMP:PMA_AA_15m vs. UTP:PMA_DPI_15m	ns	0.1101
AMP:PMA_AA_15m vs. UTP:PMA_AA_15m	ns	0.9366
AMP:PMA_AA_15m vs. UTP:PMA_IAA_15m	****	<0.0001
AMP:PMA_IAA_15m vs. ADP:PMA_15m	ns	>0.9999
AMP:PMA_IAA_15m vs. ADP:PMA_2DG_15m	ns	0.5688
AMP:PMA_IAA_15m vs. ADP:PMA_6AN_15m	ns	0.2255
AMP:PMA_IAA_15m vs. ADP:PMA_DPI_15m	ns	0.9984

AMP:PMA_IAA_15m vs. ADP:PMA_AA_15m	ns	0.1565
AMP:PMA_IAA_15m vs. ADP:PMA_IAA_15m	ns	>0.9999
AMP:PMA_IAA_15m vs. ATP:PMA_15m	ns	>0.9999
AMP:PMA_IAA_15m vs. ATP:PMA_2DG_15m	ns	>0.9999
AMP:PMA_IAA_15m vs. ATP:PMA_6AN_15m	ns	>0.9999
AMP:PMA_IAA_15m vs. ATP:PMA_DPI_15m	ns	>0.9999
AMP:PMA_IAA_15m vs. ATP:PMA_AA_15m	ns	>0.9999
AMP:PMA_IAA_15m vs. ATP:PMA_IAA_15m	ns	0.6011
AMP:PMA_IAA_15m vs. Xanthine:PMA_15m	ns	>0.9999
AMP:PMA_IAA_15m vs. Xanthine:PMA_2DG_15m	ns	>0.9999
AMP:PMA_IAA_15m vs. Xanthine:PMA_6AN_15m	ns	0.9992
AMP:PMA_IAA_15m vs. Xanthine:PMA_DPI_15m	ns	>0.9999
AMP:PMA_IAA_15m vs. Xanthine:PMA_AA_15m	ns	0.9963
AMP:PMA_IAA_15m vs. CMP:PMA_15m	ns	0.9986
AMP:PMA_IAA_15m vs. CMP:PMA_2DG_15m	ns	0.2097
AMP:PMA_IAA_15m vs. CMP:PMA_6AN_15m	ns	0.0549
AMP:PMA_IAA_15m vs. CMP:PMA_DPI_15m	ns	0.929
AMP:PMA_IAA_15m vs. CMP:PMA_AA_15m	*	0.0343
AMP:PMA_IAA_15m vs. CMP:PMA_IAA_15m	ns	>0.9999
AMP:PMA_IAA_15m vs. CDP:PMA_15m	ns	0.8994
AMP:PMA_IAA_15m vs. CDP:PMA_2DG_15m	*	0.038
AMP:PMA_IAA_15m vs. CDP:PMA_6AN_15m	**	0.0071
AMP:PMA_IAA_15m vs. CDP:PMA_DPI_15m	ns	0.5366
AMP:PMA_IAA_15m vs. CDP:PMA_AA_15m	**	0.0041
AMP:PMA_IAA_15m vs. CDP:PMA_IAA_15m	ns	>0.9999
AMP:PMA_IAA_15m vs. CTP:PMA_15m	ns	>0.9999
AMP:PMA_IAA_15m vs. CTP:PMA_2DG_15m	ns	>0.9999
AMP:PMA_IAA_15m vs. CTP:PMA_6AN_15m	ns	>0.9999
AMP:PMA_IAA_15m vs. CTP:PMA_DPI_15m	ns	>0.9999
AMP:PMA_IAA_15m vs. CTP:PMA_AA_15m	ns	>0.9999
AMP:PMA_IAA_15m vs. CTP:PMA_IAA_15m	ns	0.9853
AMP:PMA_IAA_15m vs. Uridine:PMA_15m	ns	0.99
AMP:PMA_IAA_15m vs. Uridine:PMA_2DG_15m	ns	0.1209
AMP:PMA_IAA_15m vs. Uridine:PMA_6AN_15m	*	0.0276
AMP:PMA_IAA_15m vs. Uridine:PMA_DPI_15m	ns	0.8266
AMP:PMA_IAA_15m vs. Uridine:PMA_AA_15m	*	0.0167
AMP:PMA_IAA_15m vs. Uridine:PMA_IAA_15m	ns	>0.9999
AMP:PMA_IAA_15m vs. UMP:PMA_15m	ns	0.6264
AMP:PMA_IAA_15m vs. UMP:PMA_2DG_15m	**	0.0091
AMP:PMA_IAA_15m vs. UMP:PMA_6AN_15m	**	0.0014
AMP:PMA_IAA_15m vs. UMP:PMA_DPI_15m	ns	0.2418
AMP:PMA_IAA_15m vs. UMP:PMA_AA_15m	***	0.0008
AMP:PMA_IAA_15m vs. UMP:PMA_IAA_15m	ns	>0.9999
AMP:PMA_IAA_15m vs. UDP:PMA_15m	ns	0.6432
AMP:PMA_IAA_15m vs. UDP:PMA_2DG_15m	**	0.0099
AMP:PMA_IAA_15m vs. UDP:PMA_6AN_15m	**	0.0016
AMP:PMA_IAA_15m vs. UDP:PMA_DPI_15m	ns	0.2537

AMP:PMA_IAA_15m vs. UDP:PMA_AA_15m	***	0.0009
AMP:PMA_IAA_15m vs. UDP:PMA_IAA_15m	ns	>0.9999
AMP:PMA_IAA_15m vs. UTP:PMA_15m	ns	>0.9999
AMP:PMA_IAA_15m vs. UTP:PMA_2DG_15m	ns	>0.9999
AMP:PMA_IAA_15m vs. UTP:PMA_6AN_15m	ns	>0.9999
AMP:PMA_IAA_15m vs. UTP:PMA_DPI_15m	ns	>0.9999
AMP:PMA_IAA_15m vs. UTP:PMA_AA_15m	ns	>0.9999
AMP:PMA_IAA_15m vs. UTP:PMA_IAA_15m	ns	0.9366
ADP:PMA_15m vs. ADP:PMA_2DG_15m	ns	0.7475
ADP:PMA_15m vs. ADP:PMA_6AN_15m	ns	0.1037
ADP:PMA_15m vs. ADP:PMA_DPI_15m	ns	>0.9999
ADP:PMA_15m vs. ADP:PMA_AA_15m	*	0.0409
ADP:PMA_15m vs. ADP:PMA_IAA_15m	*	0.0236
ADP:PMA_15m vs. ATP:PMA_15m	ns	0.8929
ADP:PMA_15m vs. ATP:PMA_2DG_15m	ns	>0.9999
ADP:PMA_15m vs. ATP:PMA_6AN_15m	ns	>0.9999
ADP:PMA_15m vs. ATP:PMA_DPI_15m	ns	>0.9999
ADP:PMA_15m vs. ATP:PMA_AA_15m	ns	>0.9999
ADP:PMA_15m vs. ATP:PMA_IAA_15m	*	0.0107
ADP:PMA_15m vs. Xanthine:PMA_15m	ns	>0.9999
ADP:PMA_15m vs. Xanthine:PMA_2DG_15m	ns	>0.9999
ADP:PMA_15m vs. Xanthine:PMA_6AN_15m	ns	>0.9999
ADP:PMA_15m vs. Xanthine:PMA_DPI_15m	ns	>0.9999
ADP:PMA_15m vs. Xanthine:PMA_AA_15m	ns	>0.9999
ADP:PMA_15m vs. CMP:PMA_15m	ns	>0.9999
ADP:PMA_15m vs. CMP:PMA_2DG_15m	ns	>0.9999
ADP:PMA_15m vs. CMP:PMA_6AN_15m	ns	0.9953
ADP:PMA_15m vs. CMP:PMA_DPI_15m	ns	>0.9999
ADP:PMA_15m vs. CMP:PMA_AA_15m	ns	0.9846
ADP:PMA_15m vs. CMP:PMA_IAA_15m	ns	>0.9999
ADP:PMA_15m vs. CDP:PMA_15m	ns	>0.9999
ADP:PMA_15m vs. CDP:PMA_2DG_15m	ns	0.9878
ADP:PMA_15m vs. CDP:PMA_6AN_15m	ns	0.8348
ADP:PMA_15m vs. CDP:PMA_DPI_15m	ns	>0.9999
ADP:PMA_15m vs. CDP:PMA_AA_15m	ns	0.7362
ADP:PMA_15m vs. CDP:PMA_IAA_15m	ns	>0.9999
ADP:PMA_15m vs. CTP:PMA_15m	ns	0.9998
ADP:PMA_15m vs. CTP:PMA_2DG_15m	ns	>0.9999
ADP:PMA_15m vs. CTP:PMA_6AN_15m	ns	>0.9999
ADP:PMA_15m vs. CTP:PMA_DPI_15m	ns	>0.9999
ADP:PMA_15m vs. CTP:PMA_AA_15m	ns	>0.9999
ADP:PMA_15m vs. CTP:PMA_IAA_15m	ns	0.0983
ADP:PMA_15m vs. Uridine:PMA_15m	ns	>0.9999
ADP:PMA_15m vs. Uridine:PMA_2DG_15m	ns	0.9998
ADP:PMA_15m vs. Uridine:PMA_6AN_15m	ns	0.9756
ADP:PMA_15m vs. Uridine:PMA_DPI_15m	ns	>0.9999
ADP:PMA_15m vs. Uridine:PMA_AA_15m	ns	0.9419

ADP:PMA_15m vs. Uridine:PMA_IAA_15m	ns	>0.9999
ADP:PMA_15m vs. UMP:PMA_15m	ns	>0.9999
ADP:PMA_15m vs. UMP:PMA_2DG_15m	ns	0.8731
ADP:PMA_15m vs. UMP:PMA_6AN_15m	ns	0.5234
ADP:PMA_15m vs. UMP:PMA_DPI_15m	ns	>0.9999
ADP:PMA_15m vs. UMP:PMA_AA_15m	ns	0.4071
ADP:PMA_15m vs. UMP:PMA_IAA_15m	ns	>0.9999
ADP:PMA_15m vs. UDP:PMA_15m	ns	>0.9999
ADP:PMA_15m vs. UDP:PMA_2DG_15m	ns	0.8836
ADP:PMA_15m vs. UDP:PMA_6AN_15m	ns	0.5405
ADP:PMA_15m vs. UDP:PMA_DPI_15m	ns	>0.9999
ADP:PMA_15m vs. UDP:PMA_AA_15m	ns	0.423
ADP:PMA_15m vs. UDP:PMA_IAA_15m	ns	>0.9999
ADP:PMA_15m vs. UTP:PMA_15m	ns	0.9971
ADP:PMA_15m vs. UTP:PMA_2DG_15m	ns	>0.9999
ADP:PMA_15m vs. UTP:PMA_6AN_15m	ns	>0.9999
ADP:PMA_15m vs. UTP:PMA_DPI_15m	ns	>0.9999
ADP:PMA_15m vs. UTP:PMA_AA_15m	ns	>0.9999
ADP:PMA_15m vs. UTP:PMA_IAA_15m	ns	0.0529
ADP:PMA_2DG_15m vs. ADP:PMA_6AN_15m	ns	>0.9999
ADP:PMA_2DG_15m vs. ADP:PMA_DPI_15m	ns	>0.9999
ADP:PMA_2DG_15m vs. ADP:PMA_AA_15m	ns	>0.9999
ADP:PMA_2DG_15m vs. ADP:PMA_IAA_15m	****	<0.0001
ADP:PMA_2DG_15m vs. ATP:PMA_15m	ns	0.1127
ADP:PMA_2DG_15m vs. ATP:PMA_2DG_15m	ns	0.8929
ADP:PMA_2DG_15m vs. ATP:PMA_6AN_15m	ns	>0.9999
ADP:PMA_2DG_15m vs. ATP:PMA_DPI_15m	ns	0.3899
ADP:PMA_2DG_15m vs. ATP:PMA_AA_15m	ns	>0.9999
ADP:PMA_2DG_15m vs. ATP:PMA_IAA_15m	****	<0.0001
ADP:PMA_2DG_15m vs. Xanthine:PMA_15m	ns	0.9068
ADP:PMA_2DG_15m vs. Xanthine:PMA_2DG_15m	ns	>0.9999
ADP:PMA_2DG_15m vs. Xanthine:PMA_6AN_15m	ns	>0.9999
ADP:PMA_2DG_15m vs. Xanthine:PMA_DPI_15m	ns	0.997
ADP:PMA_2DG_15m vs. Xanthine:PMA_AA_15m	ns	>0.9999
ADP:PMA_2DG_15m vs. CMP:PMA_15m	ns	>0.9999
ADP:PMA_2DG_15m vs. CMP:PMA_2DG_15m	ns	>0.9999
ADP:PMA_2DG_15m vs. CMP:PMA_6AN_15m	ns	>0.9999
ADP:PMA_2DG_15m vs. CMP:PMA_DPI_15m	ns	>0.9999
ADP:PMA_2DG_15m vs. CMP:PMA_AA_15m	ns	>0.9999
ADP:PMA_2DG_15m vs. CMP:PMA_IAA_15m	ns	0.6795
ADP:PMA_2DG_15m vs. CDP:PMA_15m	ns	>0.9999
ADP:PMA_2DG_15m vs. CDP:PMA_2DG_15m	ns	>0.9999
ADP:PMA_2DG_15m vs. CDP:PMA_6AN_15m	ns	>0.9999
ADP:PMA_2DG_15m vs. CDP:PMA_DPI_15m	ns	>0.9999
ADP:PMA_2DG_15m vs. CDP:PMA_AA_15m	ns	>0.9999
ADP:PMA_2DG_15m vs. CDP:PMA_IAA_15m	ns	0.9739
ADP:PMA_2DG_15m vs. CTP:PMA_15m	ns	0.5133

ADP:PMA_2DG_15m vs. CTP:PMA_2DG_15m	ns	0.9998
ADP:PMA_2DG_15m vs. CTP:PMA_6AN_15m	ns	>0.9999
ADP:PMA_2DG_15m vs. CTP:PMA_DPI_15m	ns	0.8872
ADP:PMA_2DG_15m vs. CTP:PMA_AA_15m	ns	>0.9999
ADP:PMA_2DG_15m vs. CTP:PMA_IAA_15m	***	0.0003
ADP:PMA_2DG_15m vs. Uridine:PMA_15m	ns	>0.9999
ADP:PMA_2DG_15m vs. Uridine:PMA_2DG_15m	ns	>0.9999
ADP:PMA_2DG_15m vs. Uridine:PMA_6AN_15m	ns	>0.9999
ADP:PMA_2DG_15m vs. Uridine:PMA_DPI_15m	ns	>0.9999
ADP:PMA_2DG_15m vs. Uridine:PMA_AA_15m	ns	>0.9999
ADP:PMA_2DG_15m vs. Uridine:PMA_IAA_15m	ns	0.8296
ADP:PMA_2DG_15m vs. UMP:PMA_15m	ns	>0.9999
ADP:PMA_2DG_15m vs. UMP:PMA_2DG_15m	ns	>0.9999
ADP:PMA_2DG_15m vs. UMP:PMA_6AN_15m	ns	>0.9999
ADP:PMA_2DG_15m vs. UMP:PMA_DPI_15m	ns	>0.9999
ADP:PMA_2DG_15m vs. UMP:PMA_AA_15m	ns	>0.9999
ADP:PMA_2DG_15m vs. UMP:PMA_IAA_15m	ns	0.9993
ADP:PMA_2DG_15m vs. UDP:PMA_15m	ns	>0.9999
ADP:PMA_2DG_15m vs. UDP:PMA_2DG_15m	ns	>0.9999
ADP:PMA_2DG_15m vs. UDP:PMA_6AN_15m	ns	>0.9999
ADP:PMA_2DG_15m vs. UDP:PMA_DPI_15m	ns	>0.9999
ADP:PMA_2DG_15m vs. UDP:PMA_AA_15m	ns	>0.9999
ADP:PMA_2DG_15m vs. UDP:PMA_IAA_15m	ns	0.9991
ADP:PMA_2DG_15m vs. UTP:PMA_15m	ns	0.3538
ADP:PMA_2DG_15m vs. UTP:PMA_2DG_15m	ns	0.9971
ADP:PMA_2DG_15m vs. UTP:PMA_6AN_15m	ns	>0.9999
ADP:PMA_2DG_15m vs. UTP:PMA_DPI_15m	ns	0.7632
ADP:PMA_2DG_15m vs. UTP:PMA_AA_15m	ns	>0.9999
ADP:PMA_2DG_15m vs. UTP:PMA_IAA_15m	***	0.0001
ADP:PMA_6AN_15m vs. ADP:PMA_DPI_15m	ns	0.8059
ADP:PMA_6AN_15m vs. ADP:PMA_AA_15m	ns	>0.9999
ADP:PMA_6AN_15m vs. ADP:PMA_IAA_15m	****	<0.0001
ADP:PMA_6AN_15m vs. ATP:PMA_15m	*	0.0252
ADP:PMA_6AN_15m vs. ATP:PMA_2DG_15m	ns	0.8383
ADP:PMA_6AN_15m vs. ATP:PMA_6AN_15m	ns	0.8929
ADP:PMA_6AN_15m vs. ATP:PMA_DPI_15m	ns	0.1262
ADP:PMA_6AN_15m vs. ATP:PMA_AA_15m	ns	0.9967
ADP:PMA_6AN_15m vs. ATP:PMA_IAA_15m	****	<0.0001
ADP:PMA_6AN_15m vs. Xanthine:PMA_15m	ns	0.5977
ADP:PMA_6AN_15m vs. Xanthine:PMA_2DG_15m	ns	>0.9999
ADP:PMA_6AN_15m vs. Xanthine:PMA_6AN_15m	ns	>0.9999
ADP:PMA_6AN_15m vs. Xanthine:PMA_DPI_15m	ns	0.9224
ADP:PMA_6AN_15m vs. Xanthine:PMA_AA_15m	ns	>0.9999
ADP:PMA_6AN_15m vs. CMP:PMA_15m	ns	>0.9999
ADP:PMA_6AN_15m vs. CMP:PMA_2DG_15m	ns	>0.9999
ADP:PMA_6AN_15m vs. CMP:PMA_6AN_15m	ns	>0.9999
ADP:PMA_6AN_15m vs. CMP:PMA_DPI_15m	ns	>0.9999

ADP:PMA_6AN_15m vs. CMP:PMA_AA_15m	ns	>0.9999
ADP:PMA_6AN_15m vs. CMP:PMA_IAA_15m	ns	0.3068
ADP:PMA_6AN_15m vs. CDP:PMA_15m	ns	>0.9999
ADP:PMA_6AN_15m vs. CDP:PMA_2DG_15m	ns	>0.9999
ADP:PMA_6AN_15m vs. CDP:PMA_6AN_15m	ns	>0.9999
ADP:PMA_6AN_15m vs. CDP:PMA_DPI_15m	ns	>0.9999
ADP:PMA_6AN_15m vs. CDP:PMA_AA_15m	ns	>0.9999
ADP:PMA_6AN_15m vs. CDP:PMA_IAA_15m	ns	0.7657
ADP:PMA_6AN_15m vs. CTP:PMA_15m	ns	0.1905
ADP:PMA_6AN_15m vs. CTP:PMA_2DG_15m	ns	0.998
ADP:PMA_6AN_15m vs. CTP:PMA_6AN_15m	ns	0.9998
ADP:PMA_6AN_15m vs. CTP:PMA_DPI_15m	ns	0.5467
ADP:PMA_6AN_15m vs. CTP:PMA_AA_15m	ns	>0.9999
ADP:PMA_6AN_15m vs. CTP:PMA_IAA_15m	****	<0.0001
ADP:PMA_6AN_15m vs. Uridine:PMA_15m	ns	>0.9999
ADP:PMA_6AN_15m vs. Uridine:PMA_2DG_15m	ns	>0.9999
ADP:PMA_6AN_15m vs. Uridine:PMA_6AN_15m	ns	>0.9999
ADP:PMA_6AN_15m vs. Uridine:PMA_DPI_15m	ns	>0.9999
ADP:PMA_6AN_15m vs. Uridine:PMA_AA_15m	ns	>0.9999
ADP:PMA_6AN_15m vs. Uridine:PMA_IAA_15m	ns	0.4619
ADP:PMA_6AN_15m vs. UMP:PMA_15m	ns	>0.9999
ADP:PMA_6AN_15m vs. UMP:PMA_2DG_15m	ns	>0.9999
ADP:PMA_6AN_15m vs. UMP:PMA_6AN_15m	ns	>0.9999
ADP:PMA_6AN_15m vs. UMP:PMA_DPI_15m	ns	>0.9999
ADP:PMA_6AN_15m vs. UMP:PMA_AA_15m	ns	>0.9999
ADP:PMA_6AN_15m vs. UMP:PMA_IAA_15m	ns	0.9601
ADP:PMA_6AN_15m vs. UDP:PMA_15m	ns	>0.9999
ADP:PMA_6AN_15m vs. UDP:PMA_2DG_15m	ns	>0.9999
ADP:PMA_6AN_15m vs. UDP:PMA_6AN_15m	ns	>0.9999
ADP:PMA_6AN_15m vs. UDP:PMA_DPI_15m	ns	>0.9999
ADP:PMA_6AN_15m vs. UDP:PMA_AA_15m	ns	>0.9999
ADP:PMA_6AN_15m vs. UDP:PMA_IAA_15m	ns	0.955
ADP:PMA_6AN_15m vs. UTP:PMA_15m	ns	0.1099
ADP:PMA_6AN_15m vs. UTP:PMA_2DG_15m	ns	0.9875
ADP:PMA_6AN_15m vs. UTP:PMA_6AN_15m	ns	0.9971
ADP:PMA_6AN_15m vs. UTP:PMA_DPI_15m	ns	0.3832
ADP:PMA_6AN_15m vs. UTP:PMA_AA_15m	ns	>0.9999
ADP:PMA_6AN_15m vs. UTP:PMA_IAA_15m	****	<0.0001
ADP:PMA_DPI_15m vs. ADP:PMA_AA_15m	ns	0.5731
ADP:PMA_DPI_15m vs. ADP:PMA_IAA_15m	***	0.0004
ADP:PMA_DPI_15m vs. ATP:PMA_15m	ns	0.8134
ADP:PMA_DPI_15m vs. ATP:PMA_2DG_15m	ns	>0.9999
ADP:PMA_DPI_15m vs. ATP:PMA_6AN_15m	ns	>0.9999
ADP:PMA_DPI_15m vs. ATP:PMA_DPI_15m	ns	0.8929
ADP:PMA_DPI_15m vs. ATP:PMA_AA_15m	ns	>0.9999
ADP:PMA_DPI_15m vs. ATP:PMA_IAA_15m	**	0.0015
ADP:PMA_DPI_15m vs. Xanthine:PMA_15m	ns	>0.9999

ADP:PMA_DPI_15m vs. Xanthine:PMA_2DG_15m	ns	>0.9999
ADP:PMA_DPI_15m vs. Xanthine:PMA_6AN_15m	ns	>0.9999
ADP:PMA_DPI_15m vs. Xanthine:PMA_DPI_15m	ns	>0.9999
ADP:PMA_DPI_15m vs. Xanthine:PMA_AA_15m	ns	>0.9999
ADP:PMA_DPI_15m vs. CMP:PMA_15m	ns	>0.9999
ADP:PMA_DPI_15m vs. CMP:PMA_2DG_15m	ns	>0.9999
ADP:PMA_DPI_15m vs. CMP:PMA_6AN_15m	ns	>0.9999
ADP:PMA_DPI_15m vs. CMP:PMA_DPI_15m	ns	>0.9999
ADP:PMA_DPI_15m vs. CMP:PMA_AA_15m	ns	>0.9999
ADP:PMA_DPI_15m vs. CMP:PMA_IAA_15m	ns	0.9997
ADP:PMA_DPI_15m vs. CDP:PMA_15m	ns	>0.9999
ADP:PMA_DPI_15m vs. CDP:PMA_2DG_15m	ns	>0.9999
ADP:PMA_DPI_15m vs. CDP:PMA_6AN_15m	ns	0.9911
ADP:PMA_DPI_15m vs. CDP:PMA_DPI_15m	ns	>0.9999
ADP:PMA_DPI_15m vs. CDP:PMA_AA_15m	ns	0.9742
ADP:PMA_DPI_15m vs. CDP:PMA_IAA_15m	ns	>0.9999
ADP:PMA_DPI_15m vs. CTP:PMA_15m	ns	0.997
ADP:PMA_DPI_15m vs. CTP:PMA_2DG_15m	ns	>0.9999
ADP:PMA_DPI_15m vs. CTP:PMA_6AN_15m	ns	>0.9999
ADP:PMA_DPI_15m vs. CTP:PMA_DPI_15m	ns	0.9998
ADP:PMA_DPI_15m vs. CTP:PMA_AA_15m	ns	>0.9999
ADP:PMA_DPI_15m vs. CTP:PMA_IAA_15m	*	0.0187
ADP:PMA_DPI_15m vs. Uridine:PMA_15m	ns	>0.9999
ADP:PMA_DPI_15m vs. Uridine:PMA_2DG_15m	ns	>0.9999
ADP:PMA_DPI_15m vs. Uridine:PMA_6AN_15m	ns	0.9999
ADP:PMA_DPI_15m vs. Uridine:PMA_DPI_15m	ns	>0.9999
ADP:PMA_DPI_15m vs. Uridine:PMA_AA_15m	ns	0.9991
ADP:PMA_DPI_15m vs. Uridine:PMA_IAA_15m	ns	>0.9999
ADP:PMA_DPI_15m vs. UMP:PMA_15m	ns	>0.9999
ADP:PMA_DPI_15m vs. UMP:PMA_2DG_15m	ns	0.9951
ADP:PMA_DPI_15m vs. UMP:PMA_6AN_15m	ns	0.893
ADP:PMA_DPI_15m vs. UMP:PMA_DPI_15m	ns	>0.9999
ADP:PMA_DPI_15m vs. UMP:PMA_AA_15m	ns	0.8121
ADP:PMA_DPI_15m vs. UMP:PMA_IAA_15m	ns	>0.9999
ADP:PMA_DPI_15m vs. UDP:PMA_15m	ns	>0.9999
ADP:PMA_DPI_15m vs. UDP:PMA_2DG_15m	ns	0.9959
ADP:PMA_DPI_15m vs. UDP:PMA_6AN_15m	ns	0.9023
ADP:PMA_DPI_15m vs. UDP:PMA_DPI_15m	ns	>0.9999
ADP:PMA_DPI_15m vs. UDP:PMA_AA_15m	ns	0.8251
ADP:PMA_DPI_15m vs. UDP:PMA_IAA_15m	ns	>0.9999
ADP:PMA_DPI_15m vs. UTP:PMA_15m	ns	0.9831
ADP:PMA_DPI_15m vs. UTP:PMA_2DG_15m	ns	>0.9999
ADP:PMA_DPI_15m vs. UTP:PMA_6AN_15m	ns	>0.9999
ADP:PMA_DPI_15m vs. UTP:PMA_DPI_15m	ns	0.9971
ADP:PMA_DPI_15m vs. UTP:PMA_AA_15m	ns	>0.9999
ADP:PMA_DPI_15m vs. UTP:PMA_IAA_15m	**	0.0089
ADP:PMA_AA_15m vs. ADP:PMA_IAA_15m	****	<0.0001

ADP:PMA_AA_15m vs. ATP:PMA_15m	*	0.0152
ADP:PMA_AA_15m vs. ATP:PMA_2DG_15m	ns	0.7405
ADP:PMA_AA_15m vs. ATP:PMA_6AN_15m	ns	0.9678
ADP:PMA_AA_15m vs. ATP:PMA_DPI_15m	ns	0.0831
ADP:PMA_AA_15m vs. ATP:PMA_AA_15m	ns	0.8929
ADP:PMA_AA_15m vs. ATP:PMA_IAA_15m	****	<0.0001
ADP:PMA_AA_15m vs. Xanthine:PMA_15m	ns	0.4822
ADP:PMA_AA_15m vs. Xanthine:PMA_2DG_15m	ns	>0.9999
ADP:PMA_AA_15m vs. Xanthine:PMA_6AN_15m	ns	>0.9999
ADP:PMA_AA_15m vs. Xanthine:PMA_DPI_15m	ns	0.8572
ADP:PMA_AA_15m vs. Xanthine:PMA_AA_15m	ns	>0.9999
ADP:PMA_AA_15m vs. CMP:PMA_15m	ns	>0.9999
ADP:PMA_AA_15m vs. CMP:PMA_2DG_15m	ns	>0.9999
ADP:PMA_AA_15m vs. CMP:PMA_6AN_15m	ns	>0.9999
ADP:PMA_AA_15m vs. CMP:PMA_DPI_15m	ns	>0.9999
ADP:PMA_AA_15m vs. CMP:PMA_AA_15m	ns	>0.9999
ADP:PMA_AA_15m vs. CMP:PMA_IAA_15m	ns	0.2203
ADP:PMA_AA_15m vs. CDP:PMA_15m	ns	>0.9999
ADP:PMA_AA_15m vs. CDP:PMA_2DG_15m	ns	>0.9999
ADP:PMA_AA_15m vs. CDP:PMA_6AN_15m	ns	>0.9999
ADP:PMA_AA_15m vs. CDP:PMA_DPI_15m	ns	>0.9999
ADP:PMA_AA_15m vs. CDP:PMA_AA_15m	ns	>0.9999
ADP:PMA_AA_15m vs. CDP:PMA_IAA_15m	ns	0.6546
ADP:PMA_AA_15m vs. CTP:PMA_15m	ns	0.1299
ADP:PMA_AA_15m vs. CTP:PMA_2DG_15m	ns	0.9923
ADP:PMA_AA_15m vs. CTP:PMA_6AN_15m	ns	>0.9999
ADP:PMA_AA_15m vs. CTP:PMA_DPI_15m	ns	0.4288
ADP:PMA_AA_15m vs. CTP:PMA_AA_15m	ns	0.9998
ADP:PMA_AA_15m vs. CTP:PMA_IAA_15m	****	<0.0001
ADP:PMA_AA_15m vs. Uridine:PMA_15m	ns	>0.9999
ADP:PMA_AA_15m vs. Uridine:PMA_2DG_15m	ns	>0.9999
ADP:PMA_AA_15m vs. Uridine:PMA_6AN_15m	ns	>0.9999
ADP:PMA_AA_15m vs. Uridine:PMA_DPI_15m	ns	>0.9999
ADP:PMA_AA_15m vs. Uridine:PMA_AA_15m	ns	>0.9999
ADP:PMA_AA_15m vs. Uridine:PMA_IAA_15m	ns	0.3516
ADP:PMA_AA_15m vs. UMP:PMA_15m	ns	>0.9999
ADP:PMA_AA_15m vs. UMP:PMA_2DG_15m	ns	>0.9999
ADP:PMA_AA_15m vs. UMP:PMA_6AN_15m	ns	>0.9999
ADP:PMA_AA_15m vs. UMP:PMA_DPI_15m	ns	>0.9999
ADP:PMA_AA_15m vs. UMP:PMA_AA_15m	ns	>0.9999
ADP:PMA_AA_15m vs. UMP:PMA_IAA_15m	ns	0.9141
ADP:PMA_AA_15m vs. UDP:PMA_15m	ns	>0.9999
ADP:PMA_AA_15m vs. UDP:PMA_2DG_15m	ns	>0.9999
ADP:PMA_AA_15m vs. UDP:PMA_6AN_15m	ns	>0.9999
ADP:PMA_AA_15m vs. UDP:PMA_DPI_15m	ns	>0.9999
ADP:PMA_AA_15m vs. UDP:PMA_AA_15m	ns	>0.9999
ADP:PMA_AA_15m vs. UDP:PMA_IAA_15m	ns	0.9056

ADP:PMA_AA_15m vs. UTP:PMA_15m	ns	0.0717
ADP:PMA_AA_15m vs. UTP:PMA_2DG_15m	ns	0.966
ADP:PMA_AA_15m vs. UTP:PMA_6AN_15m	ns	0.9995
ADP:PMA_AA_15m vs. UTP:PMA_DPI_15m	ns	0.2831
ADP:PMA_AA_15m vs. UTP:PMA_AA_15m	ns	0.9971
ADP:PMA_AA_15m vs. UTP:PMA_IAA_15m	****	<0.0001
ADP:PMA_IAA_15m vs. ATP:PMA_15m	ns	>0.9999
ADP:PMA_IAA_15m vs. ATP:PMA_2DG_15m	ns	>0.9999
ADP:PMA_IAA_15m vs. ATP:PMA_6AN_15m	ns	>0.9999
ADP:PMA_IAA_15m vs. ATP:PMA_DPI_15m	ns	>0.9999
ADP:PMA_IAA_15m vs. ATP:PMA_AA_15m	ns	>0.9999
ADP:PMA_IAA_15m vs. ATP:PMA_IAA_15m	ns	0.8929
ADP:PMA_IAA_15m vs. Xanthine:PMA_15m	ns	>0.9999
ADP:PMA_IAA_15m vs. Xanthine:PMA_2DG_15m	ns	0.9998
ADP:PMA_IAA_15m vs. Xanthine:PMA_6AN_15m	ns	0.9778
ADP:PMA_IAA_15m vs. Xanthine:PMA_DPI_15m	ns	>0.9999
ADP:PMA_IAA_15m vs. Xanthine:PMA_AA_15m	ns	0.9469
ADP:PMA_IAA_15m vs. CMP:PMA_15m	ns	0.9687
ADP:PMA_IAA_15m vs. CMP:PMA_2DG_15m	ns	0.0759
ADP:PMA_IAA_15m vs. CMP:PMA_6AN_15m	*	0.0158
ADP:PMA_IAA_15m vs. CMP:PMA_DPI_15m	ns	0.7147
ADP:PMA_IAA_15m vs. CMP:PMA_AA_15m	**	0.0093
ADP:PMA_IAA_15m vs. CMP:PMA_IAA_15m	ns	>0.9999
ADP:PMA_IAA_15m vs. CDP:PMA_15m	ns	0.6566
ADP:PMA_IAA_15m vs. CDP:PMA_2DG_15m	*	0.0105
ADP:PMA_IAA_15m vs. CDP:PMA_6AN_15m	**	0.0017
ADP:PMA_IAA_15m vs. CDP:PMA_DPI_15m	ns	0.2634
ADP:PMA_IAA_15m vs. CDP:PMA_AA_15m	***	0.0009
ADP:PMA_IAA_15m vs. CDP:PMA_IAA_15m	ns	>0.9999
ADP:PMA_IAA_15m vs. CTP:PMA_15m	ns	>0.9999
ADP:PMA_IAA_15m vs. CTP:PMA_2DG_15m	ns	>0.9999
ADP:PMA_IAA_15m vs. CTP:PMA_6AN_15m	ns	0.9994
ADP:PMA_IAA_15m vs. CTP:PMA_DPI_15m	ns	>0.9999
ADP:PMA_IAA_15m vs. CTP:PMA_AA_15m	ns	0.9972
ADP:PMA_IAA_15m vs. CTP:PMA_IAA_15m	ns	0.9998
ADP:PMA_IAA_15m vs. Uridine:PMA_15m	ns	0.9034
ADP:PMA_IAA_15m vs. Uridine:PMA_2DG_15m	*	0.0392
ADP:PMA_IAA_15m vs. Uridine:PMA_6AN_15m	**	0.0073
ADP:PMA_IAA_15m vs. Uridine:PMA_DPI_15m	ns	0.544
ADP:PMA_IAA_15m vs. Uridine:PMA_AA_15m	**	0.0042
ADP:PMA_IAA_15m vs. Uridine:PMA_IAA_15m	ns	>0.9999
ADP:PMA_IAA_15m vs. UMP:PMA_15m	ns	0.3336
ADP:PMA_IAA_15m vs. UMP:PMA_2DG_15m	**	0.0022
ADP:PMA_IAA_15m vs. UMP:PMA_6AN_15m	***	0.0003
ADP:PMA_IAA_15m vs. UMP:PMA_DPI_15m	ns	0.0906
ADP:PMA_IAA_15m vs. UMP:PMA_AA_15m	***	0.0002
ADP:PMA_IAA_15m vs. UMP:PMA_IAA_15m	ns	>0.9999

ADP:PMA_IAA_15m vs. UDP:PMA_15m	ns	0.348
ADP:PMA_IAA_15m vs. UDP:PMA_2DG_15m	**	0.0024
ADP:PMA_IAA_15m vs. UDP:PMA_6AN_15m	***	0.0003
ADP:PMA_IAA_15m vs. UDP:PMA_DPI_15m	ns	0.0962
ADP:PMA_IAA_15m vs. UDP:PMA_AA_15m	***	0.0002
ADP:PMA_IAA_15m vs. UDP:PMA_IAA_15m	ns	>0.9999
ADP:PMA_IAA_15m vs. UTP:PMA_15m	ns	>0.9999
ADP:PMA_IAA_15m vs. UTP:PMA_2DG_15m	ns	>0.9999
ADP:PMA_IAA_15m vs. UTP:PMA_6AN_15m	ns	>0.9999
ADP:PMA_IAA_15m vs. UTP:PMA_DPI_15m	ns	>0.9999
ADP:PMA_IAA_15m vs. UTP:PMA_AA_15m	ns	0.9997
ADP:PMA_IAA_15m vs. UTP:PMA_IAA_15m	ns	0.9971
ATP:PMA_15m vs. ATP:PMA_2DG_15m	ns	0.7475
ATP:PMA_15m vs. ATP:PMA_6AN_15m	ns	0.1037
ATP:PMA_15m vs. ATP:PMA_DPI_15m	ns	>0.9999
ATP:PMA_15m vs. ATP:PMA_AA_15m	*	0.0409
ATP:PMA_15m vs. ATP:PMA_IAA_15m	*	0.0236
ATP:PMA_15m vs. Xanthine:PMA_15m	ns	>0.9999
ATP:PMA_15m vs. Xanthine:PMA_2DG_15m	ns	0.994
ATP:PMA_15m vs. Xanthine:PMA_6AN_15m	ns	0.8913
ATP:PMA_15m vs. Xanthine:PMA_DPI_15m	ns	>0.9999
ATP:PMA_15m vs. Xanthine:PMA_AA_15m	ns	0.8134
ATP:PMA_15m vs. CMP:PMA_15m	ns	0.4736
ATP:PMA_15m vs. CMP:PMA_2DG_15m	*	0.0227
ATP:PMA_15m vs. CMP:PMA_6AN_15m	**	0.0039
ATP:PMA_15m vs. CMP:PMA_DPI_15m	ns	0.4165
ATP:PMA_15m vs. CMP:PMA_AA_15m	**	0.0022
ATP:PMA_15m vs. CMP:PMA_IAA_15m	ns	>0.9999
ATP:PMA_15m vs. CDP:PMA_15m	ns	0.0978
ATP:PMA_15m vs. CDP:PMA_2DG_15m	**	0.0025
ATP:PMA_15m vs. CDP:PMA_6AN_15m	***	0.0004
ATP:PMA_15m vs. CDP:PMA_DPI_15m	ns	0.1009
ATP:PMA_15m vs. CDP:PMA_AA_15m	***	0.0002
ATP:PMA_15m vs. CDP:PMA_IAA_15m	ns	>0.9999
ATP:PMA_15m vs. CTP:PMA_15m	ns	>0.9999
ATP:PMA_15m vs. CTP:PMA_2DG_15m	ns	0.9999
ATP:PMA_15m vs. CTP:PMA_6AN_15m	ns	0.9821
ATP:PMA_15m vs. CTP:PMA_DPI_15m	ns	>0.9999
ATP:PMA_15m vs. CTP:PMA_AA_15m	ns	0.9546
ATP:PMA_15m vs. CTP:PMA_IAA_15m	ns	>0.9999
ATP:PMA_15m vs. Uridine:PMA_15m	ns	0.2964
ATP:PMA_15m vs. Uridine:PMA_2DG_15m	*	0.0107
ATP:PMA_15m vs. Uridine:PMA_6AN_15m	**	0.0017
ATP:PMA_15m vs. Uridine:PMA_DPI_15m	ns	0.2697
ATP:PMA_15m vs. Uridine:PMA_AA_15m	***	0.0009
ATP:PMA_15m vs. Uridine:PMA_IAA_15m	ns	>0.9999
ATP:PMA_15m vs. UMP:PMA_15m	*	0.0221

ATP:PMA_15m vs. UMP:PMA_2DG_15m	***	0.0005
ATP:PMA_15m vs. UMP:PMA_6AN_15m	****	<0.0001
ATP:PMA_15m vs. UMP:PMA_DPI_15m	*	0.0279
ATP:PMA_15m vs. UMP:PMA_AA_15m	****	<0.0001
ATP:PMA_15m vs. UMP:PMA_IAA_15m	ns	>0.9999
ATP:PMA_15m vs. UDP:PMA_15m	*	0.0239
ATP:PMA_15m vs. UDP:PMA_2DG_15m	***	0.0005
ATP:PMA_15m vs. UDP:PMA_6AN_15m	****	<0.0001
ATP:PMA_15m vs. UDP:PMA_DPI_15m	*	0.0299
ATP:PMA_15m vs. UDP:PMA_AA_15m	****	<0.0001
ATP:PMA_15m vs. UDP:PMA_IAA_15m	ns	>0.9999
ATP:PMA_15m vs. UTP:PMA_15m	ns	>0.9999
ATP:PMA_15m vs. UTP:PMA_2DG_15m	ns	>0.9999
ATP:PMA_15m vs. UTP:PMA_6AN_15m	ns	0.9968
ATP:PMA_15m vs. UTP:PMA_DPI_15m	ns	>0.9999
ATP:PMA_15m vs. UTP:PMA_AA_15m	ns	0.9885
ATP:PMA_15m vs. UTP:PMA_IAA_15m	ns	>0.9999
ATP:PMA_2DG_15m vs. ATP:PMA_6AN_15m	ns	>0.9999
ATP:PMA_2DG_15m vs. ATP:PMA_DPI_15m	ns	>0.9999
ATP:PMA_2DG_15m vs. ATP:PMA_AA_15m	ns	>0.9999
ATP:PMA_2DG_15m vs. ATP:PMA_IAA_15m	****	<0.0001
ATP:PMA_2DG_15m vs. Xanthine:PMA_15m	ns	>0.9999
ATP:PMA_2DG_15m vs. Xanthine:PMA_2DG_15m	ns	>0.9999
ATP:PMA_2DG_15m vs. Xanthine:PMA_6AN_15m	ns	>0.9999
ATP:PMA_2DG_15m vs. Xanthine:PMA_DPI_15m	ns	>0.9999
ATP:PMA_2DG_15m vs. Xanthine:PMA_AA_15m	ns	>0.9999
ATP:PMA_2DG_15m vs. CMP:PMA_15m	ns	>0.9999
ATP:PMA_2DG_15m vs. CMP:PMA_2DG_15m	ns	0.4736
ATP:PMA_2DG_15m vs. CMP:PMA_6AN_15m	ns	0.4481
ATP:PMA_2DG_15m vs. CMP:PMA_DPI_15m	ns	>0.9999
ATP:PMA_2DG_15m vs. CMP:PMA_AA_15m	ns	0.339
ATP:PMA_2DG_15m vs. CMP:PMA_IAA_15m	ns	>0.9999
ATP:PMA_2DG_15m vs. CDP:PMA_15m	ns	>0.9999
ATP:PMA_2DG_15m vs. CDP:PMA_2DG_15m	ns	0.0978
ATP:PMA_2DG_15m vs. CDP:PMA_6AN_15m	ns	0.1132
ATP:PMA_2DG_15m vs. CDP:PMA_DPI_15m	ns	0.9843
ATP:PMA_2DG_15m vs. CDP:PMA_AA_15m	ns	0.0739
ATP:PMA_2DG_15m vs. CDP:PMA_IAA_15m	ns	>0.9999
ATP:PMA_2DG_15m vs. CTP:PMA_15m	ns	>0.9999
ATP:PMA_2DG_15m vs. CTP:PMA_2DG_15m	ns	>0.9999
ATP:PMA_2DG_15m vs. CTP:PMA_6AN_15m	ns	>0.9999
ATP:PMA_2DG_15m vs. CTP:PMA_DPI_15m	ns	>0.9999
ATP:PMA_2DG_15m vs. CTP:PMA_AA_15m	ns	>0.9999
ATP:PMA_2DG_15m vs. CTP:PMA_IAA_15m	ns	0.8012
ATP:PMA_2DG_15m vs. Uridine:PMA_15m	ns	>0.9999
ATP:PMA_2DG_15m vs. Uridine:PMA_2DG_15m	ns	0.2964
ATP:PMA_2DG_15m vs. Uridine:PMA_6AN_15m	ns	0.295

ATP:PMA_2DG_15m vs. Uridine:PMA_DPI_15m	ns	0.9996
ATP:PMA_2DG_15m vs. Uridine:PMA_AA_15m	ns	0.2105
ATP:PMA_2DG_15m vs. Uridine:PMA_IAA_15m	ns	>0.9999
ATP:PMA_2DG_15m vs. UMP:PMA_15m	ns	0.9934
ATP:PMA_2DG_15m vs. UMP:PMA_2DG_15m	*	0.0221
ATP:PMA_2DG_15m vs. UMP:PMA_6AN_15m	*	0.0319
ATP:PMA_2DG_15m vs. UMP:PMA_DPI_15m	ns	0.8553
ATP:PMA_2DG_15m vs. UMP:PMA_AA_15m	*	0.0194
ATP:PMA_2DG_15m vs. UMP:PMA_IAA_15m	ns	>0.9999
ATP:PMA_2DG_15m vs. UDP:PMA_15m	ns	0.9945
ATP:PMA_2DG_15m vs. UDP:PMA_2DG_15m	*	0.0239
ATP:PMA_2DG_15m vs. UDP:PMA_6AN_15m	*	0.0342
ATP:PMA_2DG_15m vs. UDP:PMA_DPI_15m	ns	0.8666
ATP:PMA_2DG_15m vs. UDP:PMA_AA_15m	*	0.0208
ATP:PMA_2DG_15m vs. UDP:PMA_IAA_15m	ns	>0.9999
ATP:PMA_2DG_15m vs. UTP:PMA_15m	ns	>0.9999
ATP:PMA_2DG_15m vs. UTP:PMA_2DG_15m	ns	>0.9999
ATP:PMA_2DG_15m vs. UTP:PMA_6AN_15m	ns	>0.9999
ATP:PMA_2DG_15m vs. UTP:PMA_DPI_15m	ns	>0.9999
ATP:PMA_2DG_15m vs. UTP:PMA_AA_15m	ns	>0.9999
ATP:PMA_2DG_15m vs. UTP:PMA_IAA_15m	ns	0.6482
ATP:PMA_6AN_15m vs. ATP:PMA_DPI_15m	ns	0.8059
ATP:PMA_6AN_15m vs. ATP:PMA_AA_15m	ns	>0.9999
ATP:PMA_6AN_15m vs. ATP:PMA_IAA_15m	****	<0.0001
ATP:PMA_6AN_15m vs. Xanthine:PMA_15m	ns	>0.9999
ATP:PMA_6AN_15m vs. Xanthine:PMA_2DG_15m	ns	>0.9999
ATP:PMA_6AN_15m vs. Xanthine:PMA_6AN_15m	ns	>0.9999
ATP:PMA_6AN_15m vs. Xanthine:PMA_DPI_15m	ns	>0.9999
ATP:PMA_6AN_15m vs. Xanthine:PMA_AA_15m	ns	>0.9999
ATP:PMA_6AN_15m vs. CMP:PMA_15m	ns	>0.9999
ATP:PMA_6AN_15m vs. CMP:PMA_2DG_15m	ns	0.9853
ATP:PMA_6AN_15m vs. CMP:PMA_6AN_15m	ns	0.4736
ATP:PMA_6AN_15m vs. CMP:PMA_DPI_15m	ns	>0.9999
ATP:PMA_6AN_15m vs. CMP:PMA_AA_15m	ns	0.7179
ATP:PMA_6AN_15m vs. CMP:PMA_IAA_15m	ns	>0.9999
ATP:PMA_6AN_15m vs. CDP:PMA_15m	ns	>0.9999
ATP:PMA_6AN_15m vs. CDP:PMA_2DG_15m	ns	0.7413
ATP:PMA_6AN_15m vs. CDP:PMA_6AN_15m	ns	0.0978
ATP:PMA_6AN_15m vs. CDP:PMA_DPI_15m	ns	0.9999
ATP:PMA_6AN_15m vs. CDP:PMA_AA_15m	ns	0.2646
ATP:PMA_6AN_15m vs. CDP:PMA_IAA_15m	ns	>0.9999
ATP:PMA_6AN_15m vs. CTP:PMA_15m	ns	>0.9999
ATP:PMA_6AN_15m vs. CTP:PMA_2DG_15m	ns	>0.9999
ATP:PMA_6AN_15m vs. CTP:PMA_6AN_15m	ns	>0.9999
ATP:PMA_6AN_15m vs. CTP:PMA_DPI_15m	ns	>0.9999
ATP:PMA_6AN_15m vs. CTP:PMA_AA_15m	ns	>0.9999
ATP:PMA_6AN_15m vs. CTP:PMA_IAA_15m	ns	0.4266

ATP:PMA_6AN_15m vs. Uridine:PMA_15m	ns	>0.9999
ATP:PMA_6AN_15m vs. Uridine:PMA_2DG_15m	ns	0.944
ATP:PMA_6AN_15m vs. Uridine:PMA_6AN_15m	ns	0.2964
ATP:PMA_6AN_15m vs. Uridine:PMA_DPI_15m	ns	>0.9999
ATP:PMA_6AN_15m vs. Uridine:PMA_AA_15m	ns	0.5467
ATP:PMA_6AN_15m vs. Uridine:PMA_IAA_15m	ns	>0.9999
ATP:PMA_6AN_15m vs. UMP:PMA_15m	ns	>0.9999
ATP:PMA_6AN_15m vs. UMP:PMA_2DG_15m	ns	0.4123
ATP:PMA_6AN_15m vs. UMP:PMA_6AN_15m	*	0.0221
ATP:PMA_6AN_15m vs. UMP:PMA_DPI_15m	ns	0.9908
ATP:PMA_6AN_15m vs. UMP:PMA_AA_15m	ns	0.0907
ATP:PMA_6AN_15m vs. UMP:PMA_IAA_15m	ns	>0.9999
ATP:PMA_6AN_15m vs. UDP:PMA_15m	ns	>0.9999
ATP:PMA_6AN_15m vs. UDP:PMA_2DG_15m	ns	0.4283
ATP:PMA_6AN_15m vs. UDP:PMA_6AN_15m	*	0.0239
ATP:PMA_6AN_15m vs. UDP:PMA_DPI_15m	ns	0.9922
ATP:PMA_6AN_15m vs. UDP:PMA_AA_15m	ns	0.0963
ATP:PMA_6AN_15m vs. UDP:PMA_IAA_15m	ns	>0.9999
ATP:PMA_6AN_15m vs. UTP:PMA_15m	ns	>0.9999
ATP:PMA_6AN_15m vs. UTP:PMA_2DG_15m	ns	>0.9999
ATP:PMA_6AN_15m vs. UTP:PMA_6AN_15m	ns	>0.9999
ATP:PMA_6AN_15m vs. UTP:PMA_DPI_15m	ns	>0.9999
ATP:PMA_6AN_15m vs. UTP:PMA_AA_15m	ns	>0.9999
ATP:PMA_6AN_15m vs. UTP:PMA_IAA_15m	ns	0.2818
ATP:PMA_DPI_15m vs. ATP:PMA_AA_15m	ns	0.5731
ATP:PMA_DPI_15m vs. ATP:PMA_IAA_15m	***	0.0004
ATP:PMA_DPI_15m vs. Xanthine:PMA_15m	ns	>0.9999
ATP:PMA_DPI_15m vs. Xanthine:PMA_2DG_15m	ns	>0.9999
ATP:PMA_DPI_15m vs. Xanthine:PMA_6AN_15m	ns	0.9958
ATP:PMA_DPI_15m vs. Xanthine:PMA_DPI_15m	ns	>0.9999
ATP:PMA_DPI_15m vs. Xanthine:PMA_AA_15m	ns	0.9864
ATP:PMA_DPI_15m vs. CMP:PMA_15m	ns	0.9892
ATP:PMA_DPI_15m vs. CMP:PMA_2DG_15m	ns	0.116
ATP:PMA_DPI_15m vs. CMP:PMA_6AN_15m	*	0.0261
ATP:PMA_DPI_15m vs. CMP:PMA_DPI_15m	ns	0.4736
ATP:PMA_DPI_15m vs. CMP:PMA_AA_15m	*	0.0157
ATP:PMA_DPI_15m vs. CMP:PMA_IAA_15m	ns	>0.9999
ATP:PMA_DPI_15m vs. CDP:PMA_15m	ns	0.7705
ATP:PMA_DPI_15m vs. CDP:PMA_2DG_15m	*	0.0176
ATP:PMA_DPI_15m vs. CDP:PMA_6AN_15m	**	0.0029
ATP:PMA_DPI_15m vs. CDP:PMA_DPI_15m	ns	0.0978
ATP:PMA_DPI_15m vs. CDP:PMA_AA_15m	**	0.0017
ATP:PMA_DPI_15m vs. CDP:PMA_IAA_15m	ns	>0.9999
ATP:PMA_DPI_15m vs. CTP:PMA_15m	ns	>0.9999
ATP:PMA_DPI_15m vs. CTP:PMA_2DG_15m	ns	>0.9999
ATP:PMA_DPI_15m vs. CTP:PMA_6AN_15m	ns	>0.9999
ATP:PMA_DPI_15m vs. CTP:PMA_DPI_15m	ns	>0.9999

ATP:PMA_DPI_15m vs. CTP:PMA_AA_15m	ns	0.9995
ATP:PMA_DPI_15m vs. CTP:PMA_IAA_15m	ns	>0.9999
ATP:PMA_DPI_15m vs. Uridine:PMA_15m	ns	0.9552
ATP:PMA_DPI_15m vs. Uridine:PMA_2DG_15m	ns	0.0622
ATP:PMA_DPI_15m vs. Uridine:PMA_6AN_15m	*	0.0124
ATP:PMA_DPI_15m vs. Uridine:PMA_DPI_15m	ns	0.2964
ATP:PMA_DPI_15m vs. Uridine:PMA_AA_15m	**	0.0073
ATP:PMA_DPI_15m vs. Uridine:PMA_IAA_15m	ns	>0.9999
ATP:PMA_DPI_15m vs. UMP:PMA_15m	ns	0.4438
ATP:PMA_DPI_15m vs. UMP:PMA_2DG_15m	**	0.0039
ATP:PMA_DPI_15m vs. UMP:PMA_6AN_15m	***	0.0006
ATP:PMA_DPI_15m vs. UMP:PMA_DPI_15m	*	0.0221
ATP:PMA_DPI_15m vs. UMP:PMA_AA_15m	***	0.0003
ATP:PMA_DPI_15m vs. UMP:PMA_IAA_15m	ns	>0.9999
ATP:PMA_DPI_15m vs. UDP:PMA_15m	ns	0.4602
ATP:PMA_DPI_15m vs. UDP:PMA_2DG_15m	**	0.0042
ATP:PMA_DPI_15m vs. UDP:PMA_6AN_15m	***	0.0006
ATP:PMA_DPI_15m vs. UDP:PMA_DPI_15m	*	0.0239
ATP:PMA_DPI_15m vs. UDP:PMA_AA_15m	***	0.0003
ATP:PMA_DPI_15m vs. UDP:PMA_IAA_15m	ns	>0.9999
ATP:PMA_DPI_15m vs. UTP:PMA_15m	ns	>0.9999
ATP:PMA_DPI_15m vs. UTP:PMA_2DG_15m	ns	>0.9999
ATP:PMA_DPI_15m vs. UTP:PMA_6AN_15m	ns	>0.9999
ATP:PMA_DPI_15m vs. UTP:PMA_DPI_15m	ns	>0.9999
ATP:PMA_DPI_15m vs. UTP:PMA_AA_15m	ns	>0.9999
ATP:PMA_DPI_15m vs. UTP:PMA_IAA_15m	ns	0.9995
ATP:PMA_AA_15m vs. ATP:PMA_IAA_15m	****	<0.0001
ATP:PMA_AA_15m vs. Xanthine:PMA_15m	ns	>0.9999
ATP:PMA_AA_15m vs. Xanthine:PMA_2DG_15m	ns	>0.9999
ATP:PMA_AA_15m vs. Xanthine:PMA_6AN_15m	ns	>0.9999
ATP:PMA_AA_15m vs. Xanthine:PMA_DPI_15m	ns	>0.9999
ATP:PMA_AA_15m vs. Xanthine:PMA_AA_15m	ns	>0.9999
ATP:PMA_AA_15m vs. CMP:PMA_15m	ns	>0.9999
ATP:PMA_AA_15m vs. CMP:PMA_2DG_15m	ns	0.9956
ATP:PMA_AA_15m vs. CMP:PMA_6AN_15m	ns	0.8986
ATP:PMA_AA_15m vs. CMP:PMA_DPI_15m	ns	>0.9999
ATP:PMA_AA_15m vs. CMP:PMA_AA_15m	ns	0.4736
ATP:PMA_AA_15m vs. CMP:PMA_IAA_15m	ns	>0.9999
ATP:PMA_AA_15m vs. CDP:PMA_15m	ns	>0.9999
ATP:PMA_AA_15m vs. CDP:PMA_2DG_15m	ns	0.8389
ATP:PMA_AA_15m vs. CDP:PMA_6AN_15m	ns	0.473
ATP:PMA_AA_15m vs. CDP:PMA_DPI_15m	ns	>0.9999
ATP:PMA_AA_15m vs. CDP:PMA_AA_15m	ns	0.0978
ATP:PMA_AA_15m vs. CDP:PMA_IAA_15m	ns	>0.9999
ATP:PMA_AA_15m vs. CTP:PMA_15m	ns	>0.9999
ATP:PMA_AA_15m vs. CTP:PMA_2DG_15m	ns	>0.9999
ATP:PMA_AA_15m vs. CTP:PMA_6AN_15m	ns	>0.9999

ATP:PMA_AA_15m vs. CTP:PMA_DPI_15m	ns	>0.9999
ATP:PMA_AA_15m vs. CTP:PMA_AA_15m	ns	>0.9999
ATP:PMA_AA_15m vs. CTP:PMA_IAA_15m	ns	0.3206
ATP:PMA_AA_15m vs. Uridine:PMA_15m	ns	>0.9999
ATP:PMA_AA_15m vs. Uridine:PMA_2DG_15m	ns	0.9767
ATP:PMA_AA_15m vs. Uridine:PMA_6AN_15m	ns	0.7764
ATP:PMA_AA_15m vs. Uridine:PMA_DPI_15m	ns	>0.9999
ATP:PMA_AA_15m vs. Uridine:PMA_AA_15m	ns	0.2964
ATP:PMA_AA_15m vs. Uridine:PMA_IAA_15m	ns	>0.9999
ATP:PMA_AA_15m vs. UMP:PMA_15m	ns	>0.9999
ATP:PMA_AA_15m vs. UMP:PMA_2DG_15m	ns	0.5291
ATP:PMA_AA_15m vs. UMP:PMA_6AN_15m	ns	0.1998
ATP:PMA_AA_15m vs. UMP:PMA_DPI_15m	ns	0.9975
ATP:PMA_AA_15m vs. UMP:PMA_AA_15m	*	0.0221
ATP:PMA_AA_15m vs. UMP:PMA_IAA_15m	ns	>0.9999
ATP:PMA_AA_15m vs. UDP:PMA_15m	ns	>0.9999
ATP:PMA_AA_15m vs. UDP:PMA_2DG_15m	ns	0.5462
ATP:PMA_AA_15m vs. UDP:PMA_6AN_15m	ns	0.2102
ATP:PMA_AA_15m vs. UDP:PMA_DPI_15m	ns	0.998
ATP:PMA_AA_15m vs. UDP:PMA_AA_15m	*	0.0239
ATP:PMA_AA_15m vs. UDP:PMA_IAA_15m	ns	>0.9999
ATP:PMA_AA_15m vs. UTP:PMA_15m	ns	>0.9999
ATP:PMA_AA_15m vs. UTP:PMA_2DG_15m	ns	>0.9999
ATP:PMA_AA_15m vs. UTP:PMA_6AN_15m	ns	>0.9999
ATP:PMA_AA_15m vs. UTP:PMA_DPI_15m	ns	>0.9999
ATP:PMA_AA_15m vs. UTP:PMA_AA_15m	ns	>0.9999
ATP:PMA_AA_15m vs. UTP:PMA_IAA_15m	ns	0.2003
ATP:PMA_IAA_15m vs. Xanthine:PMA_15m	ns	0.6869
ATP:PMA_IAA_15m vs. Xanthine:PMA_2DG_15m	*	0.0132
ATP:PMA_IAA_15m vs. Xanthine:PMA_6AN_15m	**	0.0022
ATP:PMA_IAA_15m vs. Xanthine:PMA_DPI_15m	ns	0.292
ATP:PMA_IAA_15m vs. Xanthine:PMA_AA_15m	**	0.0013
ATP:PMA_IAA_15m vs. CMP:PMA_15m	**	0.0015
ATP:PMA_IAA_15m vs. CMP:PMA_2DG_15m	****	<0.0001
ATP:PMA_IAA_15m vs. CMP:PMA_6AN_15m	****	<0.0001
ATP:PMA_IAA_15m vs. CMP:PMA_DPI_15m	***	0.0002
ATP:PMA_IAA_15m vs. CMP:PMA_AA_15m	****	<0.0001
ATP:PMA_IAA_15m vs. CMP:PMA_IAA_15m	ns	0.4736
ATP:PMA_IAA_15m vs. CDP:PMA_15m	***	0.0001
ATP:PMA_IAA_15m vs. CDP:PMA_2DG_15m	****	<0.0001
ATP:PMA_IAA_15m vs. CDP:PMA_6AN_15m	****	<0.0001
ATP:PMA_IAA_15m vs. CDP:PMA_DPI_15m	****	<0.0001
ATP:PMA_IAA_15m vs. CDP:PMA_AA_15m	****	<0.0001
ATP:PMA_IAA_15m vs. CDP:PMA_IAA_15m	ns	0.0978
ATP:PMA_IAA_15m vs. CTP:PMA_15m	ns	0.9216
ATP:PMA_IAA_15m vs. CTP:PMA_2DG_15m	*	0.0453
ATP:PMA_IAA_15m vs. CTP:PMA_6AN_15m	**	0.0087

ATP:PMA_IAA_15m vs. CTP:PMA_DPI_15m	ns	0.581
ATP:PMA_IAA_15m vs. CTP:PMA_AA_15m	**	0.005
ATP:PMA_IAA_15m vs. CTP:PMA_IAA_15m	ns	>0.9999
ATP:PMA_IAA_15m vs. Uridine:PMA_15m	***	0.0006
ATP:PMA_IAA_15m vs. Uridine:PMA_2DG_15m	****	<0.0001
ATP:PMA_IAA_15m vs. Uridine:PMA_6AN_15m	****	<0.0001
ATP:PMA_IAA_15m vs. Uridine:PMA_DPI_15m	****	<0.0001
ATP:PMA_IAA_15m vs. Uridine:PMA_AA_15m	****	<0.0001
ATP:PMA_IAA_15m vs. Uridine:PMA_IAA_15m	ns	0.2964
ATP:PMA_IAA_15m vs. UMP:PMA_15m	****	<0.0001
ATP:PMA_IAA_15m vs. UMP:PMA_2DG_15m	****	<0.0001
ATP:PMA_IAA_15m vs. UMP:PMA_6AN_15m	****	<0.0001
ATP:PMA_IAA_15m vs. UMP:PMA_DPI_15m	****	<0.0001
ATP:PMA_IAA_15m vs. UMP:PMA_AA_15m	****	<0.0001
ATP:PMA_IAA_15m vs. UMP:PMA_IAA_15m	*	0.0221
ATP:PMA_IAA_15m vs. UDP:PMA_15m	****	<0.0001
ATP:PMA_IAA_15m vs. UDP:PMA_2DG_15m	****	<0.0001
ATP:PMA_IAA_15m vs. UDP:PMA_6AN_15m	****	<0.0001
ATP:PMA_IAA_15m vs. UDP:PMA_DPI_15m	****	<0.0001
ATP:PMA_IAA_15m vs. UDP:PMA_AA_15m	****	<0.0001
ATP:PMA_IAA_15m vs. UDP:PMA_IAA_15m	*	0.0239
ATP:PMA_IAA_15m vs. UTP:PMA_15m	ns	0.9756
ATP:PMA_IAA_15m vs. UTP:PMA_2DG_15m	ns	0.0853
ATP:PMA_IAA_15m vs. UTP:PMA_6AN_15m	*	0.0181
ATP:PMA_IAA_15m vs. UTP:PMA_DPI_15m	ns	0.744
ATP:PMA_IAA_15m vs. UTP:PMA_AA_15m	*	0.0108
ATP:PMA_IAA_15m vs. UTP:PMA_IAA_15m	ns	>0.9999
Xanthine:PMA_15m vs. Xanthine:PMA_2DG_15m	ns	0.7475
Xanthine:PMA_15m vs. Xanthine:PMA_6AN_15m	ns	0.1037
Xanthine:PMA_15m vs. Xanthine:PMA_DPI_15m	ns	>0.9999
Xanthine:PMA_15m vs. Xanthine:PMA_AA_15m	*	0.0409
Xanthine:PMA_15m vs. CMP:PMA_15m	ns	0.9999
Xanthine:PMA_15m vs. CMP:PMA_2DG_15m	ns	0.5737
Xanthine:PMA_15m vs. CMP:PMA_6AN_15m	ns	0.2378
Xanthine:PMA_15m vs. CMP:PMA_DPI_15m	ns	0.9978
Xanthine:PMA_15m vs. CMP:PMA_AA_15m	ns	0.1681
Xanthine:PMA_15m vs. CMP:PMA_IAA_15m	ns	>0.9999
Xanthine:PMA_15m vs. CDP:PMA_15m	ns	0.9531
Xanthine:PMA_15m vs. CDP:PMA_2DG_15m	ns	0.1816
Xanthine:PMA_15m vs. CDP:PMA_6AN_15m	*	0.0482
Xanthine:PMA_15m vs. CDP:PMA_DPI_15m	ns	0.8899
Xanthine:PMA_15m vs. CDP:PMA_AA_15m	*	0.0304
Xanthine:PMA_15m vs. CDP:PMA_IAA_15m	ns	>0.9999
Xanthine:PMA_15m vs. CTP:PMA_15m	ns	>0.9999
Xanthine:PMA_15m vs. CTP:PMA_2DG_15m	ns	>0.9999
Xanthine:PMA_15m vs. CTP:PMA_6AN_15m	ns	>0.9999
Xanthine:PMA_15m vs. CTP:PMA_DPI_15m	ns	>0.9999

Xanthine:PMA_15m vs. CTP:PMA_AA_15m	ns	>0.9999
Xanthine:PMA_15m vs. CTP:PMA_IAA_15m	ns	0.9851
Xanthine:PMA_15m vs. Uridine:PMA_15m	ns	0.9982
Xanthine:PMA_15m vs. Uridine:PMA_2DG_15m	ns	0.4095
Xanthine:PMA_15m vs. Uridine:PMA_6AN_15m	ns	0.1423
Xanthine:PMA_15m vs. Uridine:PMA_DPI_15m	ns	0.9869
Xanthine:PMA_15m vs. Uridine:PMA_AA_15m	ns	0.0962
Xanthine:PMA_15m vs. Uridine:PMA_IAA_15m	ns	>0.9999
Xanthine:PMA_15m vs. UMP:PMA_15m	ns	0.7184
Xanthine:PMA_15m vs. UMP:PMA_2DG_15m	ns	0.0593
Xanthine:PMA_15m vs. UMP:PMA_6AN_15m	*	0.0126
Xanthine:PMA_15m vs. UMP:PMA_DPI_15m	ns	0.6211
Xanthine:PMA_15m vs. UMP:PMA_AA_15m	**	0.0075
Xanthine:PMA_15m vs. UMP:PMA_IAA_15m	ns	>0.9999
Xanthine:PMA_15m vs. UDP:PMA_15m	ns	0.7353
Xanthine:PMA_15m vs. UDP:PMA_2DG_15m	ns	0.0631
Xanthine:PMA_15m vs. UDP:PMA_6AN_15m	*	0.0135
Xanthine:PMA_15m vs. UDP:PMA_DPI_15m	ns	0.6374
Xanthine:PMA_15m vs. UDP:PMA_AA_15m	**	0.0081
Xanthine:PMA_15m vs. UDP:PMA_IAA_15m	ns	>0.9999
Xanthine:PMA_15m vs. UTP:PMA_15m	ns	>0.9999
Xanthine:PMA_15m vs. UTP:PMA_2DG_15m	ns	>0.9999
Xanthine:PMA_15m vs. UTP:PMA_6AN_15m	ns	>0.9999
Xanthine:PMA_15m vs. UTP:PMA_DPI_15m	ns	>0.9999
Xanthine:PMA_15m vs. UTP:PMA_AA_15m	ns	>0.9999
Xanthine:PMA_15m vs. UTP:PMA_IAA_15m	ns	0.9463
Xanthine:PMA_2DG_15m vs. Xanthine:PMA_6AN_15m	ns	>0.9999
Xanthine:PMA_2DG_15m vs. Xanthine:PMA_DPI_15m	ns	>0.9999
Xanthine:PMA_2DG_15m vs. Xanthine:PMA_AA_15m	ns	>0.9999
Xanthine:PMA_2DG_15m vs. CMP:PMA_15m	ns	>0.9999
Xanthine:PMA_2DG_15m vs. CMP:PMA_2DG_15m	ns	0.9999
Xanthine:PMA_2DG_15m vs. CMP:PMA_6AN_15m	ns	0.9986
Xanthine:PMA_2DG_15m vs. CMP:PMA_DPI_15m	ns	>0.9999
Xanthine:PMA_2DG_15m vs. CMP:PMA_AA_15m	ns	0.9943
Xanthine:PMA_2DG_15m vs. CMP:PMA_IAA_15m	ns	>0.9999
Xanthine:PMA_2DG_15m vs. CDP:PMA_15m	ns	>0.9999
Xanthine:PMA_2DG_15m vs. CDP:PMA_2DG_15m	ns	0.9531
Xanthine:PMA_2DG_15m vs. CDP:PMA_6AN_15m	ns	0.9074
Xanthine:PMA_2DG_15m vs. CDP:PMA_DPI_15m	ns	>0.9999
Xanthine:PMA_2DG_15m vs. CDP:PMA_AA_15m	ns	0.8356
Xanthine:PMA_2DG_15m vs. CDP:PMA_IAA_15m	ns	>0.9999
Xanthine:PMA_2DG_15m vs. CTP:PMA_15m	ns	>0.9999
Xanthine:PMA_2DG_15m vs. CTP:PMA_2DG_15m	ns	>0.9999
Xanthine:PMA_2DG_15m vs. CTP:PMA_6AN_15m	ns	>0.9999
Xanthine:PMA_2DG_15m vs. CTP:PMA_DPI_15m	ns	>0.9999
Xanthine:PMA_2DG_15m vs. CTP:PMA_AA_15m	ns	>0.9999
Xanthine:PMA_2DG_15m vs. CTP:PMA_IAA_15m	ns	0.1122

Xanthine:PMA_2DG_15m vs. Uridine:PMA_15m	ns	>0.9999
Xanthine:PMA_2DG_15m vs. Uridine:PMA_2DG_15m	ns	0.9982
Xanthine:PMA_2DG_15m vs. Uridine:PMA_6AN_15m	ns	0.9904
Xanthine:PMA_2DG_15m vs. Uridine:PMA_DPI_15m	ns	>0.9999
Xanthine:PMA_2DG_15m vs. Uridine:PMA_AA_15m	ns	0.9734
Xanthine:PMA_2DG_15m vs. Uridine:PMA_IAA_15m	ns	>0.9999
Xanthine:PMA_2DG_15m vs. UMP:PMA_15m	ns	>0.9999
Xanthine:PMA_2DG_15m vs. UMP:PMA_2DG_15m	ns	0.7184
Xanthine:PMA_2DG_15m vs. UMP:PMA_6AN_15m	ns	0.6528
Xanthine:PMA_2DG_15m vs. UMP:PMA_DPI_15m	ns	>0.9999
Xanthine:PMA_2DG_15m vs. UMP:PMA_AA_15m	ns	0.5373
Xanthine:PMA_2DG_15m vs. UMP:PMA_IAA_15m	ns	>0.9999
Xanthine:PMA_2DG_15m vs. UDP:PMA_15m	ns	>0.9999
Xanthine:PMA_2DG_15m vs. UDP:PMA_2DG_15m	ns	0.7353
Xanthine:PMA_2DG_15m vs. UDP:PMA_6AN_15m	ns	0.6688
Xanthine:PMA_2DG_15m vs. UDP:PMA_DPI_15m	ns	>0.9999
Xanthine:PMA_2DG_15m vs. UDP:PMA_AA_15m	ns	0.5538
Xanthine:PMA_2DG_15m vs. UDP:PMA_IAA_15m	ns	>0.9999
Xanthine:PMA_2DG_15m vs. UTP:PMA_15m	ns	>0.9999
Xanthine:PMA_2DG_15m vs. UTP:PMA_2DG_15m	ns	>0.9999
Xanthine:PMA_2DG_15m vs. UTP:PMA_6AN_15m	ns	>0.9999
Xanthine:PMA_2DG_15m vs. UTP:PMA_DPI_15m	ns	>0.9999
Xanthine:PMA_2DG_15m vs. UTP:PMA_AA_15m	ns	>0.9999
Xanthine:PMA_2DG_15m vs. UTP:PMA_IAA_15m	ns	0.0618
Xanthine:PMA_6AN_15m vs. Xanthine:PMA_DPI_15m	ns	0.8059
Xanthine:PMA_6AN_15m vs. Xanthine:PMA_AA_15m	ns	>0.9999
Xanthine:PMA_6AN_15m vs. CMP:PMA_15m	ns	>0.9999
Xanthine:PMA_6AN_15m vs. CMP:PMA_2DG_15m	ns	>0.9999
Xanthine:PMA_6AN_15m vs. CMP:PMA_6AN_15m	ns	0.9999
Xanthine:PMA_6AN_15m vs. CMP:PMA_DPI_15m	ns	>0.9999
Xanthine:PMA_6AN_15m vs. CMP:PMA_AA_15m	ns	>0.9999
Xanthine:PMA_6AN_15m vs. CMP:PMA_IAA_15m	ns	0.9998
Xanthine:PMA_6AN_15m vs. CDP:PMA_15m	ns	>0.9999
Xanthine:PMA_6AN_15m vs. CDP:PMA_2DG_15m	ns	>0.9999
Xanthine:PMA_6AN_15m vs. CDP:PMA_6AN_15m	ns	0.9531
Xanthine:PMA_6AN_15m vs. CDP:PMA_DPI_15m	ns	>0.9999
Xanthine:PMA_6AN_15m vs. CDP:PMA_AA_15m	ns	0.9861
Xanthine:PMA_6AN_15m vs. CDP:PMA_IAA_15m	ns	>0.9999
Xanthine:PMA_6AN_15m vs. CTP:PMA_15m	ns	0.9991
Xanthine:PMA_6AN_15m vs. CTP:PMA_2DG_15m	ns	>0.9999
Xanthine:PMA_6AN_15m vs. CTP:PMA_6AN_15m	ns	>0.9999
Xanthine:PMA_6AN_15m vs. CTP:PMA_DPI_15m	ns	>0.9999
Xanthine:PMA_6AN_15m vs. CTP:PMA_AA_15m	ns	>0.9999
Xanthine:PMA_6AN_15m vs. CTP:PMA_IAA_15m	*	0.0259
Xanthine:PMA_6AN_15m vs. Uridine:PMA_15m	ns	>0.9999
Xanthine:PMA_6AN_15m vs. Uridine:PMA_2DG_15m	ns	>0.9999
Xanthine:PMA_6AN_15m vs. Uridine:PMA_6AN_15m	ns	0.9982

Xanthine:PMA_6AN_15m vs. Uridine:PMA_DPI_15m	ns	>0.9999
Xanthine:PMA_6AN_15m vs. Uridine:PMA_AA_15m	ns	0.9996
Xanthine:PMA_6AN_15m vs. Uridine:PMA_IAA_15m	ns	>0.9999
Xanthine:PMA_6AN_15m vs. UMP:PMA_15m	ns	>0.9999
Xanthine:PMA_6AN_15m vs. UMP:PMA_2DG_15m	ns	0.9977
Xanthine:PMA_6AN_15m vs. UMP:PMA_6AN_15m	ns	0.7184
Xanthine:PMA_6AN_15m vs. UMP:PMA_DPI_15m	ns	>0.9999
Xanthine:PMA_6AN_15m vs. UMP:PMA_AA_15m	ns	0.8724
Xanthine:PMA_6AN_15m vs. UMP:PMA_IAA_15m	ns	>0.9999
Xanthine:PMA_6AN_15m vs. UDP:PMA_15m	ns	>0.9999
Xanthine:PMA_6AN_15m vs. UDP:PMA_2DG_15m	ns	0.9981
Xanthine:PMA_6AN_15m vs. UDP:PMA_6AN_15m	ns	0.7353
Xanthine:PMA_6AN_15m vs. UDP:PMA_DPI_15m	ns	>0.9999
Xanthine:PMA_6AN_15m vs. UDP:PMA_AA_15m	ns	0.8825
Xanthine:PMA_6AN_15m vs. UDP:PMA_IAA_15m	ns	>0.9999
Xanthine:PMA_6AN_15m vs. UTP:PMA_15m	ns	0.9936
Xanthine:PMA_6AN_15m vs. UTP:PMA_2DG_15m	ns	>0.9999
Xanthine:PMA_6AN_15m vs. UTP:PMA_6AN_15m	ns	>0.9999
Xanthine:PMA_6AN_15m vs. UTP:PMA_DPI_15m	ns	>0.9999
Xanthine:PMA_6AN_15m vs. UTP:PMA_AA_15m	ns	>0.9999
Xanthine:PMA_6AN_15m vs. UTP:PMA_IAA_15m	*	0.0127
Xanthine:PMA_DPI_15m vs. Xanthine:PMA_AA_15m	ns	0.5731
Xanthine:PMA_DPI_15m vs. CMP:PMA_15m	ns	>0.9999
Xanthine:PMA_DPI_15m vs. CMP:PMA_2DG_15m	ns	0.911
Xanthine:PMA_DPI_15m vs. CMP:PMA_6AN_15m	ns	0.6059
Xanthine:PMA_DPI_15m vs. CMP:PMA_DPI_15m	ns	0.9999
Xanthine:PMA_DPI_15m vs. CMP:PMA_AA_15m	ns	0.4902
Xanthine:PMA_DPI_15m vs. CMP:PMA_IAA_15m	ns	>0.9999
Xanthine:PMA_DPI_15m vs. CDP:PMA_15m	ns	>0.9999
Xanthine:PMA_DPI_15m vs. CDP:PMA_2DG_15m	ns	0.5148
Xanthine:PMA_DPI_15m vs. CDP:PMA_6AN_15m	ns	0.2002
Xanthine:PMA_DPI_15m vs. CDP:PMA_DPI_15m	ns	0.9531
Xanthine:PMA_DPI_15m vs. CDP:PMA_AA_15m	ns	0.1392
Xanthine:PMA_DPI_15m vs. CDP:PMA_IAA_15m	ns	>0.9999
Xanthine:PMA_DPI_15m vs. CTP:PMA_15m	ns	>0.9999
Xanthine:PMA_DPI_15m vs. CTP:PMA_2DG_15m	ns	>0.9999
Xanthine:PMA_DPI_15m vs. CTP:PMA_6AN_15m	ns	>0.9999
Xanthine:PMA_DPI_15m vs. CTP:PMA_DPI_15m	ns	>0.9999
Xanthine:PMA_DPI_15m vs. CTP:PMA_AA_15m	ns	>0.9999
Xanthine:PMA_DPI_15m vs. CTP:PMA_IAA_15m	ns	0.799
Xanthine:PMA_DPI_15m vs. Uridine:PMA_15m	ns	>0.9999
Xanthine:PMA_DPI_15m vs. Uridine:PMA_2DG_15m	ns	0.8013
Xanthine:PMA_DPI_15m vs. Uridine:PMA_6AN_15m	ns	0.4396
Xanthine:PMA_DPI_15m vs. Uridine:PMA_DPI_15m	ns	0.9982
Xanthine:PMA_DPI_15m vs. Uridine:PMA_AA_15m	ns	0.3355
Xanthine:PMA_DPI_15m vs. Uridine:PMA_IAA_15m	ns	>0.9999
Xanthine:PMA_DPI_15m vs. UMP:PMA_15m	ns	0.9985

Xanthine:PMA_DPI_15m vs. UMP:PMA_2DG_15m	ns	0.2349
Xanthine:PMA_DPI_15m vs. UMP:PMA_6AN_15m	ns	0.0669
Xanthine:PMA_DPI_15m vs. UMP:PMA_DPI_15m	ns	0.7184
Xanthine:PMA_DPI_15m vs. UMP:PMA_AA_15m	*	0.043
Xanthine:PMA_DPI_15m vs. UMP:PMA_IAA_15m	ns	>0.9999
Xanthine:PMA_DPI_15m vs. UDP:PMA_15m	ns	0.9988
Xanthine:PMA_DPI_15m vs. UDP:PMA_2DG_15m	ns	0.2461
Xanthine:PMA_DPI_15m vs. UDP:PMA_6AN_15m	ns	0.0711
Xanthine:PMA_DPI_15m vs. UDP:PMA_DPI_15m	ns	0.7353
Xanthine:PMA_DPI_15m vs. UDP:PMA_AA_15m	*	0.0458
Xanthine:PMA_DPI_15m vs. UDP:PMA_IAA_15m	ns	>0.9999
Xanthine:PMA_DPI_15m vs. UTP:PMA_15m	ns	>0.9999
Xanthine:PMA_DPI_15m vs. UTP:PMA_2DG_15m	ns	>0.9999
Xanthine:PMA_DPI_15m vs. UTP:PMA_6AN_15m	ns	>0.9999
Xanthine:PMA_DPI_15m vs. UTP:PMA_DPI_15m	ns	>0.9999
Xanthine:PMA_DPI_15m vs. UTP:PMA_AA_15m	ns	>0.9999
Xanthine:PMA_DPI_15m vs. UTP:PMA_IAA_15m	ns	0.6482
Xanthine:PMA_AA_15m vs. CMP:PMA_15m	ns	>0.9999
Xanthine:PMA_AA_15m vs. CMP:PMA_2DG_15m	ns	>0.9999
Xanthine:PMA_AA_15m vs. CMP:PMA_6AN_15m	ns	>0.9999
Xanthine:PMA_AA_15m vs. CMP:PMA_DPI_15m	ns	>0.9999
Xanthine:PMA_AA_15m vs. CMP:PMA_AA_15m	ns	0.9999
Xanthine:PMA_AA_15m vs. CMP:PMA_IAA_15m	ns	0.9991
Xanthine:PMA_AA_15m vs. CDP:PMA_15m	ns	>0.9999
Xanthine:PMA_AA_15m vs. CDP:PMA_2DG_15m	ns	>0.9999
Xanthine:PMA_AA_15m vs. CDP:PMA_6AN_15m	ns	0.999
Xanthine:PMA_AA_15m vs. CDP:PMA_DPI_15m	ns	>0.9999
Xanthine:PMA_AA_15m vs. CDP:PMA_AA_15m	ns	0.9531
Xanthine:PMA_AA_15m vs. CDP:PMA_IAA_15m	ns	>0.9999
Xanthine:PMA_AA_15m vs. CTP:PMA_15m	ns	0.9962
Xanthine:PMA_AA_15m vs. CTP:PMA_2DG_15m	ns	>0.9999
Xanthine:PMA_AA_15m vs. CTP:PMA_6AN_15m	ns	>0.9999
Xanthine:PMA_AA_15m vs. CTP:PMA_DPI_15m	ns	>0.9999
Xanthine:PMA_AA_15m vs. CTP:PMA_AA_15m	ns	>0.9999
Xanthine:PMA_AA_15m vs. CTP:PMA_IAA_15m	*	0.0157
Xanthine:PMA_AA_15m vs. Uridine:PMA_15m	ns	>0.9999
Xanthine:PMA_AA_15m vs. Uridine:PMA_2DG_15m	ns	>0.9999
Xanthine:PMA_AA_15m vs. Uridine:PMA_6AN_15m	ns	>0.9999
Xanthine:PMA_AA_15m vs. Uridine:PMA_DPI_15m	ns	>0.9999
Xanthine:PMA_AA_15m vs. Uridine:PMA_AA_15m	ns	0.9982
Xanthine:PMA_AA_15m vs. Uridine:PMA_IAA_15m	ns	>0.9999
Xanthine:PMA_AA_15m vs. UMP:PMA_15m	ns	>0.9999
Xanthine:PMA_AA_15m vs. UMP:PMA_2DG_15m	ns	0.9995
Xanthine:PMA_AA_15m vs. UMP:PMA_6AN_15m	ns	0.9696
Xanthine:PMA_AA_15m vs. UMP:PMA_DPI_15m	ns	>0.9999
Xanthine:PMA_AA_15m vs. UMP:PMA_AA_15m	ns	0.7184
Xanthine:PMA_AA_15m vs. UMP:PMA_IAA_15m	ns	>0.9999

Xanthine:PMA_AA_15m vs. UDP:PMA_15m	ns	>0.9999
Xanthine:PMA_AA_15m vs. UDP:PMA_2DG_15m	ns	0.9996
Xanthine:PMA_AA_15m vs. UDP:PMA_6AN_15m	ns	0.9733
Xanthine:PMA_AA_15m vs. UDP:PMA_DPI_15m	ns	>0.9999
Xanthine:PMA_AA_15m vs. UDP:PMA_AA_15m	ns	0.7353
Xanthine:PMA_AA_15m vs. UDP:PMA_IAA_15m	ns	>0.9999
Xanthine:PMA_AA_15m vs. UTP:PMA_15m	ns	0.9808
Xanthine:PMA_AA_15m vs. UTP:PMA_2DG_15m	ns	>0.9999
Xanthine:PMA_AA_15m vs. UTP:PMA_6AN_15m	ns	>0.9999
Xanthine:PMA_AA_15m vs. UTP:PMA_DPI_15m	ns	0.9999
Xanthine:PMA_AA_15m vs. UTP:PMA_AA_15m	ns	>0.9999
Xanthine:PMA_AA_15m vs. UTP:PMA_IAA_15m	**	0.0075
CMP:PMA_15m vs. CMP:PMA_2DG_15m	ns	0.7475
CMP:PMA_15m vs. CMP:PMA_6AN_15m	ns	0.1037
CMP:PMA_15m vs. CMP:PMA_DPI_15m	ns	>0.9999
CMP:PMA_15m vs. CMP:PMA_AA_15m	*	0.0409
CMP:PMA_15m vs. CMP:PMA_IAA_15m	*	0.0236
CMP:PMA_15m vs. CDP:PMA_15m	ns	>0.9999
CMP:PMA_15m vs. CDP:PMA_2DG_15m	ns	>0.9999
CMP:PMA_15m vs. CDP:PMA_6AN_15m	ns	0.9904
CMP:PMA_15m vs. CDP:PMA_DPI_15m	ns	>0.9999
CMP:PMA_15m vs. CDP:PMA_AA_15m	ns	0.9724
CMP:PMA_15m vs. CDP:PMA_IAA_15m	ns	>0.9999
CMP:PMA_15m vs. CTP:PMA_15m	ns	0.9591
CMP:PMA_15m vs. CTP:PMA_2DG_15m	ns	>0.9999
CMP:PMA_15m vs. CTP:PMA_6AN_15m	ns	>0.9999
CMP:PMA_15m vs. CTP:PMA_DPI_15m	ns	>0.9999
CMP:PMA_15m vs. CTP:PMA_AA_15m	ns	>0.9999
CMP:PMA_15m vs. CTP:PMA_IAA_15m	*	0.0194
CMP:PMA_15m vs. Uridine:PMA_15m	ns	>0.9999
CMP:PMA_15m vs. Uridine:PMA_2DG_15m	ns	>0.9999
CMP:PMA_15m vs. Uridine:PMA_6AN_15m	ns	0.9998
CMP:PMA_15m vs. Uridine:PMA_DPI_15m	ns	>0.9999
CMP:PMA_15m vs. Uridine:PMA_AA_15m	ns	0.999
CMP:PMA_15m vs. Uridine:PMA_IAA_15m	ns	>0.9999
CMP:PMA_15m vs. UMP:PMA_15m	ns	>0.9999
CMP:PMA_15m vs. UMP:PMA_2DG_15m	ns	0.9946
CMP:PMA_15m vs. UMP:PMA_6AN_15m	ns	0.8881
CMP:PMA_15m vs. UMP:PMA_DPI_15m	ns	>0.9999
CMP:PMA_15m vs. UMP:PMA_AA_15m	ns	0.8054
CMP:PMA_15m vs. UMP:PMA_IAA_15m	ns	>0.9999
CMP:PMA_15m vs. UDP:PMA_15m	ns	>0.9999
CMP:PMA_15m vs. UDP:PMA_2DG_15m	ns	0.9955
CMP:PMA_15m vs. UDP:PMA_6AN_15m	ns	0.8977
CMP:PMA_15m vs. UDP:PMA_DPI_15m	ns	>0.9999
CMP:PMA_15m vs. UDP:PMA_AA_15m	ns	0.8187
CMP:PMA_15m vs. UDP:PMA_IAA_15m	ns	>0.9999

CMP:PMA_15m vs. UTP:PMA_15m	ns	0.871
CMP:PMA_15m vs. UTP:PMA_2DG_15m	ns	>0.9999
CMP:PMA_15m vs. UTP:PMA_6AN_15m	ns	>0.9999
CMP:PMA_15m vs. UTP:PMA_DPI_15m	ns	>0.9999
CMP:PMA_15m vs. UTP:PMA_AA_15m	ns	>0.9999
CMP:PMA_15m vs. UTP:PMA_IAA_15m	**	0.0093
CMP:PMA_2DG_15m vs. CMP:PMA_6AN_15m	ns	>0.9999
CMP:PMA_2DG_15m vs. CMP:PMA_DPI_15m	ns	>0.9999
CMP:PMA_2DG_15m vs. CMP:PMA_AA_15m	ns	>0.9999
CMP:PMA_2DG_15m vs. CMP:PMA_IAA_15m	****	<0.0001
CMP:PMA_2DG_15m vs. CDP:PMA_15m	ns	>0.9999
CMP:PMA_2DG_15m vs. CDP:PMA_2DG_15m	ns	>0.9999
CMP:PMA_2DG_15m vs. CDP:PMA_6AN_15m	ns	>0.9999
CMP:PMA_2DG_15m vs. CDP:PMA_DPI_15m	ns	>0.9999
CMP:PMA_2DG_15m vs. CDP:PMA_AA_15m	ns	>0.9999
CMP:PMA_2DG_15m vs. CDP:PMA_IAA_15m	ns	0.744
CMP:PMA_2DG_15m vs. CTP:PMA_15m	ns	0.1765
CMP:PMA_2DG_15m vs. CTP:PMA_2DG_15m	ns	0.9591
CMP:PMA_2DG_15m vs. CTP:PMA_6AN_15m	ns	>0.9999
CMP:PMA_2DG_15m vs. CTP:PMA_DPI_15m	ns	0.5218
CMP:PMA_2DG_15m vs. CTP:PMA_AA_15m	ns	>0.9999
CMP:PMA_2DG_15m vs. CTP:PMA_IAA_15m	****	<0.0001
CMP:PMA_2DG_15m vs. Uridine:PMA_15m	ns	>0.9999
CMP:PMA_2DG_15m vs. Uridine:PMA_2DG_15m	ns	>0.9999
CMP:PMA_2DG_15m vs. Uridine:PMA_6AN_15m	ns	>0.9999
CMP:PMA_2DG_15m vs. Uridine:PMA_DPI_15m	ns	>0.9999
CMP:PMA_2DG_15m vs. Uridine:PMA_AA_15m	ns	>0.9999
CMP:PMA_2DG_15m vs. Uridine:PMA_IAA_15m	ns	0.4381
CMP:PMA_2DG_15m vs. UMP:PMA_15m	ns	>0.9999
CMP:PMA_2DG_15m vs. UMP:PMA_2DG_15m	ns	>0.9999
CMP:PMA_2DG_15m vs. UMP:PMA_6AN_15m	ns	>0.9999
CMP:PMA_2DG_15m vs. UMP:PMA_DPI_15m	ns	>0.9999
CMP:PMA_2DG_15m vs. UMP:PMA_AA_15m	ns	>0.9999
CMP:PMA_2DG_15m vs. UMP:PMA_IAA_15m	ns	0.9526
CMP:PMA_2DG_15m vs. UDP:PMA_15m	ns	>0.9999
CMP:PMA_2DG_15m vs. UDP:PMA_2DG_15m	ns	>0.9999
CMP:PMA_2DG_15m vs. UDP:PMA_6AN_15m	ns	>0.9999
CMP:PMA_2DG_15m vs. UDP:PMA_DPI_15m	ns	>0.9999
CMP:PMA_2DG_15m vs. UDP:PMA_AA_15m	ns	>0.9999
CMP:PMA_2DG_15m vs. UDP:PMA_IAA_15m	ns	0.9468
CMP:PMA_2DG_15m vs. UTP:PMA_15m	ns	0.1009
CMP:PMA_2DG_15m vs. UTP:PMA_2DG_15m	ns	0.871
CMP:PMA_2DG_15m vs. UTP:PMA_6AN_15m	ns	0.9999
CMP:PMA_2DG_15m vs. UTP:PMA_DPI_15m	ns	0.3612
CMP:PMA_2DG_15m vs. UTP:PMA_AA_15m	ns	>0.9999
CMP:PMA_2DG_15m vs. UTP:PMA_IAA_15m	****	<0.0001
CMP:PMA_6AN_15m vs. CMP:PMA_DPI_15m	ns	0.8059

CMP:PMA_6AN_15m vs. CMP:PMA_AA_15m	ns	>0.9999
CMP:PMA_6AN_15m vs. CMP:PMA_IAA_15m	****	<0.0001
CMP:PMA_6AN_15m vs. CDP:PMA_15m	ns	>0.9999
CMP:PMA_6AN_15m vs. CDP:PMA_2DG_15m	ns	>0.9999
CMP:PMA_6AN_15m vs. CDP:PMA_6AN_15m	ns	>0.9999
CMP:PMA_6AN_15m vs. CDP:PMA_DPI_15m	ns	>0.9999
CMP:PMA_6AN_15m vs. CDP:PMA_AA_15m	ns	>0.9999
CMP:PMA_6AN_15m vs. CDP:PMA_IAA_15m	ns	0.3651
CMP:PMA_6AN_15m vs. CTP:PMA_15m	*	0.0438
CMP:PMA_6AN_15m vs. CTP:PMA_2DG_15m	ns	0.9199
CMP:PMA_6AN_15m vs. CTP:PMA_6AN_15m	ns	0.9591
CMP:PMA_6AN_15m vs. CTP:PMA_DPI_15m	ns	0.1955
CMP:PMA_6AN_15m vs. CTP:PMA_AA_15m	ns	0.9995
CMP:PMA_6AN_15m vs. CTP:PMA_IAA_15m	****	<0.0001
CMP:PMA_6AN_15m vs. Uridine:PMA_15m	ns	>0.9999
CMP:PMA_6AN_15m vs. Uridine:PMA_2DG_15m	ns	>0.9999
CMP:PMA_6AN_15m vs. Uridine:PMA_6AN_15m	ns	>0.9999
CMP:PMA_6AN_15m vs. Uridine:PMA_DPI_15m	ns	>0.9999
CMP:PMA_6AN_15m vs. Uridine:PMA_AA_15m	ns	>0.9999
CMP:PMA_6AN_15m vs. Uridine:PMA_IAA_15m	ns	0.1503
CMP:PMA_6AN_15m vs. UMP:PMA_15m	ns	>0.9999
CMP:PMA_6AN_15m vs. UMP:PMA_2DG_15m	ns	>0.9999
CMP:PMA_6AN_15m vs. UMP:PMA_6AN_15m	ns	>0.9999
CMP:PMA_6AN_15m vs. UMP:PMA_DPI_15m	ns	>0.9999
CMP:PMA_6AN_15m vs. UMP:PMA_AA_15m	ns	>0.9999
CMP:PMA_6AN_15m vs. UMP:PMA_IAA_15m	ns	0.6919
CMP:PMA_6AN_15m vs. UDP:PMA_15m	ns	>0.9999
CMP:PMA_6AN_15m vs. UDP:PMA_2DG_15m	ns	>0.9999
CMP:PMA_6AN_15m vs. UDP:PMA_6AN_15m	ns	>0.9999
CMP:PMA_6AN_15m vs. UDP:PMA_DPI_15m	ns	>0.9999
CMP:PMA_6AN_15m vs. UDP:PMA_AA_15m	ns	>0.9999
CMP:PMA_6AN_15m vs. UDP:PMA_IAA_15m	ns	0.6756
CMP:PMA_6AN_15m vs. UTP:PMA_15m	*	0.022
CMP:PMA_6AN_15m vs. UTP:PMA_2DG_15m	ns	0.8143
CMP:PMA_6AN_15m vs. UTP:PMA_6AN_15m	ns	0.871
CMP:PMA_6AN_15m vs. UTP:PMA_DPI_15m	ns	0.1132
CMP:PMA_6AN_15m vs. UTP:PMA_AA_15m	ns	0.9952
CMP:PMA_6AN_15m vs. UTP:PMA_IAA_15m	****	<0.0001
CMP:PMA_DPI_15m vs. CMP:PMA_AA_15m	ns	0.5731
CMP:PMA_DPI_15m vs. CMP:PMA_IAA_15m	***	0.0004
CMP:PMA_DPI_15m vs. CDP:PMA_15m	ns	>0.9999
CMP:PMA_DPI_15m vs. CDP:PMA_2DG_15m	ns	>0.9999
CMP:PMA_DPI_15m vs. CDP:PMA_6AN_15m	ns	>0.9999
CMP:PMA_DPI_15m vs. CDP:PMA_DPI_15m	ns	>0.9999
CMP:PMA_DPI_15m vs. CDP:PMA_AA_15m	ns	0.9998
CMP:PMA_DPI_15m vs. CDP:PMA_IAA_15m	ns	0.9999
CMP:PMA_DPI_15m vs. CTP:PMA_15m	ns	0.9033

CMP:PMA_DPI_15m vs. CTP:PMA_2DG_15m	ns	>0.9999
CMP:PMA_DPI_15m vs. CTP:PMA_6AN_15m	ns	>0.9999
CMP:PMA_DPI_15m vs. CTP:PMA_DPI_15m	ns	0.9591
CMP:PMA_DPI_15m vs. CTP:PMA_AA_15m	ns	>0.9999
CMP:PMA_DPI_15m vs. CTP:PMA_IAA_15m	**	0.0028
CMP:PMA_DPI_15m vs. Uridine:PMA_15m	ns	>0.9999
CMP:PMA_DPI_15m vs. Uridine:PMA_2DG_15m	ns	>0.9999
CMP:PMA_DPI_15m vs. Uridine:PMA_6AN_15m	ns	>0.9999
CMP:PMA_DPI_15m vs. Uridine:PMA_DPI_15m	ns	>0.9999
CMP:PMA_DPI_15m vs. Uridine:PMA_AA_15m	ns	>0.9999
CMP:PMA_DPI_15m vs. Uridine:PMA_IAA_15m	ns	0.9928
CMP:PMA_DPI_15m vs. UMP:PMA_15m	ns	>0.9999
CMP:PMA_DPI_15m vs. UMP:PMA_2DG_15m	ns	>0.9999
CMP:PMA_DPI_15m vs. UMP:PMA_6AN_15m	ns	0.9963
CMP:PMA_DPI_15m vs. UMP:PMA_DPI_15m	ns	>0.9999
CMP:PMA_DPI_15m vs. UMP:PMA_AA_15m	ns	0.9872
CMP:PMA_DPI_15m vs. UMP:PMA_IAA_15m	ns	>0.9999
CMP:PMA_DPI_15m vs. UDP:PMA_15m	ns	>0.9999
CMP:PMA_DPI_15m vs. UDP:PMA_2DG_15m	ns	>0.9999
CMP:PMA_DPI_15m vs. UDP:PMA_6AN_15m	ns	0.9969
CMP:PMA_DPI_15m vs. UDP:PMA_DPI_15m	ns	>0.9999
CMP:PMA_DPI_15m vs. UDP:PMA_AA_15m	ns	0.9891
CMP:PMA_DPI_15m vs. UDP:PMA_IAA_15m	ns	>0.9999
CMP:PMA_DPI_15m vs. UTP:PMA_15m	ns	0.7876
CMP:PMA_DPI_15m vs. UTP:PMA_2DG_15m	ns	>0.9999
CMP:PMA_DPI_15m vs. UTP:PMA_6AN_15m	ns	>0.9999
CMP:PMA_DPI_15m vs. UTP:PMA_DPI_15m	ns	0.871
CMP:PMA_DPI_15m vs. UTP:PMA_AA_15m	ns	>0.9999
CMP:PMA_DPI_15m vs. UTP:PMA_IAA_15m	**	0.0012
CMP:PMA_AA_15m vs. CMP:PMA_IAA_15m	****	<0.0001
CMP:PMA_AA_15m vs. CDP:PMA_15m	ns	>0.9999
CMP:PMA_AA_15m vs. CDP:PMA_2DG_15m	ns	>0.9999
CMP:PMA_AA_15m vs. CDP:PMA_6AN_15m	ns	>0.9999
CMP:PMA_AA_15m vs. CDP:PMA_DPI_15m	ns	>0.9999
CMP:PMA_AA_15m vs. CDP:PMA_AA_15m	ns	>0.9999
CMP:PMA_AA_15m vs. CDP:PMA_IAA_15m	ns	0.2681
CMP:PMA_AA_15m vs. CTP:PMA_15m	*	0.027
CMP:PMA_AA_15m vs. CTP:PMA_2DG_15m	ns	0.8504
CMP:PMA_AA_15m vs. CTP:PMA_6AN_15m	ns	0.9901
CMP:PMA_AA_15m vs. CTP:PMA_DPI_15m	ns	0.1336
CMP:PMA_AA_15m vs. CTP:PMA_AA_15m	ns	0.9591
CMP:PMA_AA_15m vs. CTP:PMA_IAA_15m	****	<0.0001
CMP:PMA_AA_15m vs. Uridine:PMA_15m	ns	>0.9999
CMP:PMA_AA_15m vs. Uridine:PMA_2DG_15m	ns	>0.9999
CMP:PMA_AA_15m vs. Uridine:PMA_6AN_15m	ns	>0.9999
CMP:PMA_AA_15m vs. Uridine:PMA_DPI_15m	ns	>0.9999
CMP:PMA_AA_15m vs. Uridine:PMA_AA_15m	ns	>0.9999

CMP:PMA_AA_15m vs. Uridine:PMA_IAA_15m	ns	0.1005
CMP:PMA_AA_15m vs. UMP:PMA_15m	ns	>0.9999
CMP:PMA_AA_15m vs. UMP:PMA_2DG_15m	ns	>0.9999
CMP:PMA_AA_15m vs. UMP:PMA_6AN_15m	ns	>0.9999
CMP:PMA_AA_15m vs. UMP:PMA_DPI_15m	ns	>0.9999
CMP:PMA_AA_15m vs. UMP:PMA_AA_15m	ns	>0.9999
CMP:PMA_AA_15m vs. UMP:PMA_IAA_15m	ns	0.5738
CMP:PMA_AA_15m vs. UDP:PMA_15m	ns	>0.9999
CMP:PMA_AA_15m vs. UDP:PMA_2DG_15m	ns	>0.9999
CMP:PMA_AA_15m vs. UDP:PMA_6AN_15m	ns	>0.9999
CMP:PMA_AA_15m vs. UDP:PMA_DPI_15m	ns	>0.9999
CMP:PMA_AA_15m vs. UDP:PMA_AA_15m	ns	>0.9999
CMP:PMA_AA_15m vs. UDP:PMA_IAA_15m	ns	0.5567
CMP:PMA_AA_15m vs. UTP:PMA_15m	*	0.0132
CMP:PMA_AA_15m vs. UTP:PMA_2DG_15m	ns	0.711
CMP:PMA_AA_15m vs. UTP:PMA_6AN_15m	ns	0.9592
CMP:PMA_AA_15m vs. UTP:PMA_DPI_15m	ns	0.0739
CMP:PMA_AA_15m vs. UTP:PMA_AA_15m	ns	0.871
CMP:PMA_AA_15m vs. UTP:PMA_IAA_15m	****	<0.0001
CMP:PMA_IAA_15m vs. CDP:PMA_15m	ns	0.9486
CMP:PMA_IAA_15m vs. CDP:PMA_2DG_15m	ns	0.0587
CMP:PMA_IAA_15m vs. CDP:PMA_6AN_15m	*	0.0117
CMP:PMA_IAA_15m vs. CDP:PMA_DPI_15m	ns	0.6482
CMP:PMA_IAA_15m vs. CDP:PMA_AA_15m	**	0.0068
CMP:PMA_IAA_15m vs. CDP:PMA_IAA_15m	ns	>0.9999
CMP:PMA_IAA_15m vs. CTP:PMA_15m	ns	>0.9999
CMP:PMA_IAA_15m vs. CTP:PMA_2DG_15m	ns	>0.9999
CMP:PMA_IAA_15m vs. CTP:PMA_6AN_15m	ns	>0.9999
CMP:PMA_IAA_15m vs. CTP:PMA_DPI_15m	ns	>0.9999
CMP:PMA_IAA_15m vs. CTP:PMA_AA_15m	ns	>0.9999
CMP:PMA_IAA_15m vs. CTP:PMA_IAA_15m	ns	0.9591
CMP:PMA_IAA_15m vs. Uridine:PMA_15m	ns	0.997
CMP:PMA_IAA_15m vs. Uridine:PMA_2DG_15m	ns	0.1737
CMP:PMA_IAA_15m vs. Uridine:PMA_6AN_15m	*	0.0432
CMP:PMA_IAA_15m vs. Uridine:PMA_DPI_15m	ns	0.8988
CMP:PMA_IAA_15m vs. Uridine:PMA_AA_15m	*	0.0267
CMP:PMA_IAA_15m vs. Uridine:PMA_IAA_15m	ns	>0.9999
CMP:PMA_IAA_15m vs. UMP:PMA_15m	ns	0.7333
CMP:PMA_IAA_15m vs. UMP:PMA_2DG_15m	*	0.0149
CMP:PMA_IAA_15m vs. UMP:PMA_6AN_15m	**	0.0025
CMP:PMA_IAA_15m vs. UMP:PMA_DPI_15m	ns	0.3265
CMP:PMA_IAA_15m vs. UMP:PMA_AA_15m	**	0.0014
CMP:PMA_IAA_15m vs. UMP:PMA_IAA_15m	ns	>0.9999
CMP:PMA_IAA_15m vs. UDP:PMA_15m	ns	0.7486
CMP:PMA_IAA_15m vs. UDP:PMA_2DG_15m	*	0.0161
CMP:PMA_IAA_15m vs. UDP:PMA_6AN_15m	**	0.0027
CMP:PMA_IAA_15m vs. UDP:PMA_DPI_15m	ns	0.3408

CMP:PMA_IAA_15m vs. UDP:PMA_AA_15m	**	0.0015
CMP:PMA_IAA_15m vs. UDP:PMA_IAA_15m	ns	>0.9999
CMP:PMA_IAA_15m vs. UTP:PMA_15m	ns	>0.9999
CMP:PMA_IAA_15m vs. UTP:PMA_2DG_15m	ns	>0.9999
CMP:PMA_IAA_15m vs. UTP:PMA_6AN_15m	ns	>0.9999
CMP:PMA_IAA_15m vs. UTP:PMA_DPI_15m	ns	>0.9999
CMP:PMA_IAA_15m vs. UTP:PMA_AA_15m	ns	>0.9999
CMP:PMA_IAA_15m vs. UTP:PMA_IAA_15m	ns	0.871
CDP:PMA_15m vs. CDP:PMA_2DG_15m	ns	0.7475
CDP:PMA_15m vs. CDP:PMA_6AN_15m	ns	0.1037
CDP:PMA_15m vs. CDP:PMA_DPI_15m	ns	>0.9999
CDP:PMA_15m vs. CDP:PMA_AA_15m	*	0.0409
CDP:PMA_15m vs. CDP:PMA_IAA_15m	*	0.0236
CDP:PMA_15m vs. CTP:PMA_15m	ns	0.5503
CDP:PMA_15m vs. CTP:PMA_2DG_15m	ns	>0.9999
CDP:PMA_15m vs. CTP:PMA_6AN_15m	ns	>0.9999
CDP:PMA_15m vs. CTP:PMA_DPI_15m	ns	0.9946
CDP:PMA_15m vs. CTP:PMA_AA_15m	ns	>0.9999
CDP:PMA_15m vs. CTP:PMA_IAA_15m	**	0.0021
CDP:PMA_15m vs. Uridine:PMA_15m	ns	>0.9999
CDP:PMA_15m vs. Uridine:PMA_2DG_15m	ns	>0.9999
CDP:PMA_15m vs. Uridine:PMA_6AN_15m	ns	>0.9999
CDP:PMA_15m vs. Uridine:PMA_DPI_15m	ns	>0.9999
CDP:PMA_15m vs. Uridine:PMA_AA_15m	ns	>0.9999
CDP:PMA_15m vs. Uridine:PMA_IAA_15m	ns	0.9869
CDP:PMA_15m vs. UMP:PMA_15m	ns	>0.9999
CDP:PMA_15m vs. UMP:PMA_2DG_15m	ns	>0.9999
CDP:PMA_15m vs. UMP:PMA_6AN_15m	ns	0.9982
CDP:PMA_15m vs. UMP:PMA_DPI_15m	ns	>0.9999
CDP:PMA_15m vs. UMP:PMA_AA_15m	ns	0.9929
CDP:PMA_15m vs. UMP:PMA_IAA_15m	ns	>0.9999
CDP:PMA_15m vs. UDP:PMA_15m	ns	>0.9999
CDP:PMA_15m vs. UDP:PMA_2DG_15m	ns	>0.9999
CDP:PMA_15m vs. UDP:PMA_6AN_15m	ns	0.9986
CDP:PMA_15m vs. UDP:PMA_DPI_15m	ns	>0.9999
CDP:PMA_15m vs. UDP:PMA_AA_15m	ns	0.9941
CDP:PMA_15m vs. UDP:PMA_IAA_15m	ns	>0.9999
CDP:PMA_15m vs. UTP:PMA_15m	ns	0.3652
CDP:PMA_15m vs. UTP:PMA_2DG_15m	ns	>0.9999
CDP:PMA_15m vs. UTP:PMA_6AN_15m	ns	>0.9999
CDP:PMA_15m vs. UTP:PMA_DPI_15m	ns	0.9739
CDP:PMA_15m vs. UTP:PMA_AA_15m	ns	>0.9999
CDP:PMA_15m vs. UTP:PMA_IAA_15m	***	0.0009
CDP:PMA_2DG_15m vs. CDP:PMA_6AN_15m	ns	>0.9999
CDP:PMA_2DG_15m vs. CDP:PMA_DPI_15m	ns	>0.9999
CDP:PMA_2DG_15m vs. CDP:PMA_AA_15m	ns	>0.9999
CDP:PMA_2DG_15m vs. CDP:PMA_IAA_15m	****	<0.0001

CDP:PMA_2DG_15m vs. CTP:PMA_15m	*	0.03
CDP:PMA_2DG_15m vs. CTP:PMA_2DG_15m	ns	0.5503
CDP:PMA_2DG_15m vs. CTP:PMA_6AN_15m	ns	0.9923
CDP:PMA_2DG_15m vs. CTP:PMA_DPI_15m	ns	0.1453
CDP:PMA_2DG_15m vs. CTP:PMA_AA_15m	ns	0.998
CDP:PMA_2DG_15m vs. CTP:PMA_IAA_15m	****	<0.0001
CDP:PMA_2DG_15m vs. Uridine:PMA_15m	ns	>0.9999
CDP:PMA_2DG_15m vs. Uridine:PMA_2DG_15m	ns	>0.9999
CDP:PMA_2DG_15m vs. Uridine:PMA_6AN_15m	ns	>0.9999
CDP:PMA_2DG_15m vs. Uridine:PMA_DPI_15m	ns	>0.9999
CDP:PMA_2DG_15m vs. Uridine:PMA_AA_15m	ns	>0.9999
CDP:PMA_2DG_15m vs. Uridine:PMA_IAA_15m	ns	0.1099
CDP:PMA_2DG_15m vs. UMP:PMA_15m	ns	>0.9999
CDP:PMA_2DG_15m vs. UMP:PMA_2DG_15m	ns	>0.9999
CDP:PMA_2DG_15m vs. UMP:PMA_6AN_15m	ns	>0.9999
CDP:PMA_2DG_15m vs. UMP:PMA_DPI_15m	ns	>0.9999
CDP:PMA_2DG_15m vs. UMP:PMA_AA_15m	ns	>0.9999
CDP:PMA_2DG_15m vs. UMP:PMA_IAA_15m	ns	0.5995
CDP:PMA_2DG_15m vs. UDP:PMA_15m	ns	>0.9999
CDP:PMA_2DG_15m vs. UDP:PMA_2DG_15m	ns	>0.9999
CDP:PMA_2DG_15m vs. UDP:PMA_6AN_15m	ns	>0.9999
CDP:PMA_2DG_15m vs. UDP:PMA_DPI_15m	ns	>0.9999
CDP:PMA_2DG_15m vs. UDP:PMA_AA_15m	ns	>0.9999
CDP:PMA_2DG_15m vs. UDP:PMA_IAA_15m	ns	0.5825
CDP:PMA_2DG_15m vs. UTP:PMA_15m	*	0.0147
CDP:PMA_2DG_15m vs. UTP:PMA_2DG_15m	ns	0.3652
CDP:PMA_2DG_15m vs. UTP:PMA_6AN_15m	ns	0.9662
CDP:PMA_2DG_15m vs. UTP:PMA_DPI_15m	ns	0.0812
CDP:PMA_2DG_15m vs. UTP:PMA_AA_15m	ns	0.9876
CDP:PMA_2DG_15m vs. UTP:PMA_IAA_15m	****	<0.0001
CDP:PMA_6AN_15m vs. CDP:PMA_DPI_15m	ns	0.8059
CDP:PMA_6AN_15m vs. CDP:PMA_AA_15m	ns	>0.9999
CDP:PMA_6AN_15m vs. CDP:PMA_IAA_15m	****	<0.0001
CDP:PMA_6AN_15m vs. CTP:PMA_15m	**	0.0054
CDP:PMA_6AN_15m vs. CTP:PMA_2DG_15m	ns	0.5145
CDP:PMA_6AN_15m vs. CTP:PMA_6AN_15m	ns	0.5503
CDP:PMA_6AN_15m vs. CTP:PMA_DPI_15m	*	0.0344
CDP:PMA_6AN_15m vs. CTP:PMA_AA_15m	ns	0.9313
CDP:PMA_6AN_15m vs. CTP:PMA_IAA_15m	****	<0.0001
CDP:PMA_6AN_15m vs. Uridine:PMA_15m	ns	0.9987
CDP:PMA_6AN_15m vs. Uridine:PMA_2DG_15m	ns	>0.9999
CDP:PMA_6AN_15m vs. Uridine:PMA_6AN_15m	ns	>0.9999
CDP:PMA_6AN_15m vs. Uridine:PMA_DPI_15m	ns	>0.9999
CDP:PMA_6AN_15m vs. Uridine:PMA_AA_15m	ns	>0.9999
CDP:PMA_6AN_15m vs. Uridine:PMA_IAA_15m	*	0.0246
CDP:PMA_6AN_15m vs. UMP:PMA_15m	ns	>0.9999
CDP:PMA_6AN_15m vs. UMP:PMA_2DG_15m	ns	>0.9999

CDP:PMA_6AN_15m vs. UMP:PMA_6AN_15m	ns	>0.9999
CDP:PMA_6AN_15m vs. UMP:PMA_DPI_15m	ns	>0.9999
CDP:PMA_6AN_15m vs. UMP:PMA_AA_15m	ns	>0.9999
CDP:PMA_6AN_15m vs. UMP:PMA_IAA_15m	ns	0.2461
CDP:PMA_6AN_15m vs. UDP:PMA_15m	ns	>0.9999
CDP:PMA_6AN_15m vs. UDP:PMA_2DG_15m	ns	>0.9999
CDP:PMA_6AN_15m vs. UDP:PMA_6AN_15m	ns	>0.9999
CDP:PMA_6AN_15m vs. UDP:PMA_DPI_15m	ns	>0.9999
CDP:PMA_6AN_15m vs. UDP:PMA_AA_15m	ns	>0.9999
CDP:PMA_6AN_15m vs. UDP:PMA_IAA_15m	ns	0.2345
CDP:PMA_6AN_15m vs. UTP:PMA_15m	**	0.0024
CDP:PMA_6AN_15m vs. UTP:PMA_2DG_15m	ns	0.3547
CDP:PMA_6AN_15m vs. UTP:PMA_6AN_15m	ns	0.3652
CDP:PMA_6AN_15m vs. UTP:PMA_DPI_15m	*	0.017
CDP:PMA_6AN_15m vs. UTP:PMA_AA_15m	ns	0.8336
CDP:PMA_6AN_15m vs. UTP:PMA_IAA_15m	****	<0.0001
CDP:PMA_DPI_15m vs. CDP:PMA_AA_15m	ns	0.5731
CDP:PMA_DPI_15m vs. CDP:PMA_IAA_15m	***	0.0004
CDP:PMA_DPI_15m vs. CTP:PMA_15m	ns	0.4815
CDP:PMA_DPI_15m vs. CTP:PMA_2DG_15m	ns	>0.9999
CDP:PMA_DPI_15m vs. CTP:PMA_6AN_15m	ns	>0.9999
CDP:PMA_DPI_15m vs. CTP:PMA_DPI_15m	ns	0.5503
CDP:PMA_DPI_15m vs. CTP:PMA_AA_15m	ns	>0.9999
CDP:PMA_DPI_15m vs. CTP:PMA_IAA_15m	***	0.0002
CDP:PMA_DPI_15m vs. Uridine:PMA_15m	ns	>0.9999
CDP:PMA_DPI_15m vs. Uridine:PMA_2DG_15m	ns	>0.9999
CDP:PMA_DPI_15m vs. Uridine:PMA_6AN_15m	ns	>0.9999
CDP:PMA_DPI_15m vs. Uridine:PMA_DPI_15m	ns	>0.9999
CDP:PMA_DPI_15m vs. Uridine:PMA_AA_15m	ns	>0.9999
CDP:PMA_DPI_15m vs. Uridine:PMA_IAA_15m	ns	0.805
CDP:PMA_DPI_15m vs. UMP:PMA_15m	ns	>0.9999
CDP:PMA_DPI_15m vs. UMP:PMA_2DG_15m	ns	>0.9999
CDP:PMA_DPI_15m vs. UMP:PMA_6AN_15m	ns	>0.9999
CDP:PMA_DPI_15m vs. UMP:PMA_DPI_15m	ns	>0.9999
CDP:PMA_DPI_15m vs. UMP:PMA_AA_15m	ns	>0.9999
CDP:PMA_DPI_15m vs. UMP:PMA_IAA_15m	ns	0.999
CDP:PMA_DPI_15m vs. UDP:PMA_15m	ns	>0.9999
CDP:PMA_DPI_15m vs. UDP:PMA_2DG_15m	ns	>0.9999
CDP:PMA_DPI_15m vs. UDP:PMA_6AN_15m	ns	>0.9999
CDP:PMA_DPI_15m vs. UDP:PMA_DPI_15m	ns	>0.9999
CDP:PMA_DPI_15m vs. UDP:PMA_AA_15m	ns	>0.9999
CDP:PMA_DPI_15m vs. UDP:PMA_IAA_15m	ns	0.9987
CDP:PMA_DPI_15m vs. UTP:PMA_15m	ns	0.3265
CDP:PMA_DPI_15m vs. UTP:PMA_2DG_15m	ns	0.9999
CDP:PMA_DPI_15m vs. UTP:PMA_6AN_15m	ns	>0.9999
CDP:PMA_DPI_15m vs. UTP:PMA_DPI_15m	ns	0.3652
CDP:PMA_DPI_15m vs. UTP:PMA_AA_15m	ns	>0.9999

CDP:PMA_DPI_15m vs. UTP:PMA_IAA_15m	***	0.0001
CDP:PMA_AA_15m vs. CDP:PMA_IAA_15m	****	<0.0001
CDP:PMA_AA_15m vs. CTP:PMA_15m	**	0.0031
CDP:PMA_AA_15m vs. CTP:PMA_2DG_15m	ns	0.3988
CDP:PMA_AA_15m vs. CTP:PMA_6AN_15m	ns	0.7778
CDP:PMA_AA_15m vs. CTP:PMA_DPI_15m	*	0.021
CDP:PMA_AA_15m vs. CTP:PMA_AA_15m	ns	0.5503
CDP:PMA_AA_15m vs. CTP:PMA_IAA_15m	****	<0.0001
CDP:PMA_AA_15m vs. Uridine:PMA_15m	ns	0.9945
CDP:PMA_AA_15m vs. Uridine:PMA_2DG_15m	ns	>0.9999
CDP:PMA_AA_15m vs. Uridine:PMA_6AN_15m	ns	>0.9999
CDP:PMA_AA_15m vs. Uridine:PMA_DPI_15m	ns	>0.9999
CDP:PMA_AA_15m vs. Uridine:PMA_AA_15m	ns	>0.9999
CDP:PMA_AA_15m vs. Uridine:PMA_IAA_15m	*	0.0148
CDP:PMA_AA_15m vs. UMP:PMA_15m	ns	>0.9999
CDP:PMA_AA_15m vs. UMP:PMA_2DG_15m	ns	>0.9999
CDP:PMA_AA_15m vs. UMP:PMA_6AN_15m	ns	>0.9999
CDP:PMA_AA_15m vs. UMP:PMA_DPI_15m	ns	>0.9999
CDP:PMA_AA_15m vs. UMP:PMA_AA_15m	ns	>0.9999
CDP:PMA_AA_15m vs. UMP:PMA_IAA_15m	ns	0.1724
CDP:PMA_AA_15m vs. UDP:PMA_15m	ns	>0.9999
CDP:PMA_AA_15m vs. UDP:PMA_2DG_15m	ns	>0.9999
CDP:PMA_AA_15m vs. UDP:PMA_6AN_15m	ns	>0.9999
CDP:PMA_AA_15m vs. UDP:PMA_DPI_15m	ns	>0.9999
CDP:PMA_AA_15m vs. UDP:PMA_AA_15m	ns	>0.9999
CDP:PMA_AA_15m vs. UDP:PMA_IAA_15m	ns	0.1634
CDP:PMA_AA_15m vs. UTP:PMA_15m	**	0.0014
CDP:PMA_AA_15m vs. UTP:PMA_2DG_15m	ns	0.2592
CDP:PMA_AA_15m vs. UTP:PMA_6AN_15m	ns	0.6194
CDP:PMA_AA_15m vs. UTP:PMA_DPI_15m	*	0.0101
CDP:PMA_AA_15m vs. UTP:PMA_AA_15m	ns	0.3652
CDP:PMA_AA_15m vs. UTP:PMA_IAA_15m	****	<0.0001
CDP:PMA_IAA_15m vs. CTP:PMA_15m	ns	>0.9999
CDP:PMA_IAA_15m vs. CTP:PMA_2DG_15m	ns	>0.9999
CDP:PMA_IAA_15m vs. CTP:PMA_6AN_15m	ns	>0.9999
CDP:PMA_IAA_15m vs. CTP:PMA_DPI_15m	ns	>0.9999
CDP:PMA_IAA_15m vs. CTP:PMA_AA_15m	ns	>0.9999
CDP:PMA_IAA_15m vs. CTP:PMA_IAA_15m	ns	0.5503
CDP:PMA_IAA_15m vs. Uridine:PMA_15m	ns	>0.9999
CDP:PMA_IAA_15m vs. Uridine:PMA_2DG_15m	ns	0.5762
CDP:PMA_IAA_15m vs. Uridine:PMA_6AN_15m	ns	0.2304
CDP:PMA_IAA_15m vs. Uridine:PMA_DPI_15m	ns	0.9986
CDP:PMA_IAA_15m vs. Uridine:PMA_AA_15m	ns	0.1602
CDP:PMA_IAA_15m vs. Uridine:PMA_IAA_15m	ns	>0.9999
CDP:PMA_IAA_15m vs. UMP:PMA_15m	ns	0.9839
CDP:PMA_IAA_15m vs. UMP:PMA_2DG_15m	ns	0.1014
CDP:PMA_IAA_15m vs. UMP:PMA_6AN_15m	*	0.0223

CDP:PMA_IAA_15m vs. UMP:PMA_DPI_15m	ns	0.7862
CDP:PMA_IAA_15m vs. UMP:PMA_AA_15m	*	0.0134
CDP:PMA_IAA_15m vs. UMP:PMA_IAA_15m	ns	>0.9999
CDP:PMA_IAA_15m vs. UDP:PMA_15m	ns	0.9862
CDP:PMA_IAA_15m vs. UDP:PMA_2DG_15m	ns	0.1075
CDP:PMA_IAA_15m vs. UDP:PMA_6AN_15m	*	0.0239
CDP:PMA_IAA_15m vs. UDP:PMA_DPI_15m	ns	0.8001
CDP:PMA_IAA_15m vs. UDP:PMA_AA_15m	*	0.0144
CDP:PMA_IAA_15m vs. UDP:PMA_IAA_15m	ns	>0.9999
CDP:PMA_IAA_15m vs. UTP:PMA_15m	ns	>0.9999
CDP:PMA_IAA_15m vs. UTP:PMA_2DG_15m	ns	>0.9999
CDP:PMA_IAA_15m vs. UTP:PMA_6AN_15m	ns	>0.9999
CDP:PMA_IAA_15m vs. UTP:PMA_DPI_15m	ns	>0.9999
CDP:PMA_IAA_15m vs. UTP:PMA_AA_15m	ns	>0.9999
CDP:PMA_IAA_15m vs. UTP:PMA_IAA_15m	ns	0.3652
CTP:PMA_15m vs. CTP:PMA_2DG_15m	ns	0.7475
CTP:PMA_15m vs. CTP:PMA_6AN_15m	ns	0.1037
CTP:PMA_15m vs. CTP:PMA_DPI_15m	ns	>0.9999
CTP:PMA_15m vs. CTP:PMA_AA_15m	*	0.0409
CTP:PMA_15m vs. CTP:PMA_IAA_15m	*	0.0236
CTP:PMA_15m vs. Uridine:PMA_15m	ns	0.8675
CTP:PMA_15m vs. Uridine:PMA_2DG_15m	ns	0.0992
CTP:PMA_15m vs. Uridine:PMA_6AN_15m	*	0.0216
CTP:PMA_15m vs. Uridine:PMA_DPI_15m	ns	0.7836
CTP:PMA_15m vs. Uridine:PMA_AA_15m	*	0.0129
CTP:PMA_15m vs. Uridine:PMA_IAA_15m	ns	>0.9999
CTP:PMA_15m vs. UMP:PMA_15m	ns	0.2186
CTP:PMA_15m vs. UMP:PMA_2DG_15m	**	0.007
CTP:PMA_15m vs. UMP:PMA_6AN_15m	**	0.0011
CTP:PMA_15m vs. UMP:PMA_DPI_15m	ns	0.205
CTP:PMA_15m vs. UMP:PMA_AA_15m	***	0.0006
CTP:PMA_15m vs. UMP:PMA_IAA_15m	ns	>0.9999
CTP:PMA_15m vs. UDP:PMA_15m	ns	0.2313
CTP:PMA_15m vs. UDP:PMA_2DG_15m	**	0.0075
CTP:PMA_15m vs. UDP:PMA_6AN_15m	**	0.0012
CTP:PMA_15m vs. UDP:PMA_DPI_15m	ns	0.2156
CTP:PMA_15m vs. UDP:PMA_AA_15m	***	0.0006
CTP:PMA_15m vs. UDP:PMA_IAA_15m	ns	>0.9999
CTP:PMA_15m vs. UTP:PMA_15m	ns	>0.9999
CTP:PMA_15m vs. UTP:PMA_2DG_15m	ns	>0.9999
CTP:PMA_15m vs. UTP:PMA_6AN_15m	ns	>0.9999
CTP:PMA_15m vs. UTP:PMA_DPI_15m	ns	>0.9999
CTP:PMA_15m vs. UTP:PMA_AA_15m	ns	>0.9999
CTP:PMA_15m vs. UTP:PMA_IAA_15m	ns	0.9971
CTP:PMA_2DG_15m vs. CTP:PMA_6AN_15m	ns	>0.9999
CTP:PMA_2DG_15m vs. CTP:PMA_DPI_15m	ns	>0.9999
CTP:PMA_2DG_15m vs. CTP:PMA_AA_15m	ns	>0.9999

CTP:PMA_2DG_15m vs. CTP:PMA_IAA_15m	****	<0.0001
CTP:PMA_2DG_15m vs. Uridine:PMA_15m	ns	>0.9999
CTP:PMA_2DG_15m vs. Uridine:PMA_2DG_15m	ns	0.8675
CTP:PMA_2DG_15m vs. Uridine:PMA_6AN_15m	ns	0.8105
CTP:PMA_2DG_15m vs. Uridine:PMA_DPI_15m	ns	>0.9999
CTP:PMA_2DG_15m vs. Uridine:PMA_AA_15m	ns	0.7065
CTP:PMA_2DG_15m vs. Uridine:PMA_IAA_15m	ns	>0.9999
CTP:PMA_2DG_15m vs. UMP:PMA_15m	ns	>0.9999
CTP:PMA_2DG_15m vs. UMP:PMA_2DG_15m	ns	0.2186
CTP:PMA_2DG_15m vs. UMP:PMA_6AN_15m	ns	0.2261
CTP:PMA_2DG_15m vs. UMP:PMA_DPI_15m	ns	0.9985
CTP:PMA_2DG_15m vs. UMP:PMA_AA_15m	ns	0.1568
CTP:PMA_2DG_15m vs. UMP:PMA_IAA_15m	ns	>0.9999
CTP:PMA_2DG_15m vs. UDP:PMA_15m	ns	>0.9999
CTP:PMA_2DG_15m vs. UDP:PMA_2DG_15m	ns	0.2313
CTP:PMA_2DG_15m vs. UDP:PMA_6AN_15m	ns	0.2375
CTP:PMA_2DG_15m vs. UDP:PMA_DPI_15m	ns	0.9988
CTP:PMA_2DG_15m vs. UDP:PMA_AA_15m	ns	0.1655
CTP:PMA_2DG_15m vs. UDP:PMA_IAA_15m	ns	>0.9999
CTP:PMA_2DG_15m vs. UTP:PMA_15m	ns	>0.9999
CTP:PMA_2DG_15m vs. UTP:PMA_2DG_15m	ns	>0.9999
CTP:PMA_2DG_15m vs. UTP:PMA_6AN_15m	ns	>0.9999
CTP:PMA_2DG_15m vs. UTP:PMA_DPI_15m	ns	>0.9999
CTP:PMA_2DG_15m vs. UTP:PMA_AA_15m	ns	>0.9999
CTP:PMA_2DG_15m vs. UTP:PMA_IAA_15m	ns	0.1763
CTP:PMA_6AN_15m vs. CTP:PMA_DPI_15m	ns	0.8059
CTP:PMA_6AN_15m vs. CTP:PMA_AA_15m	ns	>0.9999
CTP:PMA_6AN_15m vs. CTP:PMA_IAA_15m	****	<0.0001
CTP:PMA_6AN_15m vs. Uridine:PMA_15m	ns	>0.9999
CTP:PMA_6AN_15m vs. Uridine:PMA_2DG_15m	ns	0.9999
CTP:PMA_6AN_15m vs. Uridine:PMA_6AN_15m	ns	0.8675
CTP:PMA_6AN_15m vs. Uridine:PMA_DPI_15m	ns	>0.9999
CTP:PMA_6AN_15m vs. Uridine:PMA_AA_15m	ns	0.9578
CTP:PMA_6AN_15m vs. Uridine:PMA_IAA_15m	ns	>0.9999
CTP:PMA_6AN_15m vs. UMP:PMA_15m	ns	>0.9999
CTP:PMA_6AN_15m vs. UMP:PMA_2DG_15m	ns	0.9009
CTP:PMA_6AN_15m vs. UMP:PMA_6AN_15m	ns	0.2186
CTP:PMA_6AN_15m vs. UMP:PMA_DPI_15m	ns	>0.9999
CTP:PMA_6AN_15m vs. UMP:PMA_AA_15m	ns	0.4521
CTP:PMA_6AN_15m vs. UMP:PMA_IAA_15m	ns	>0.9999
CTP:PMA_6AN_15m vs. UDP:PMA_15m	ns	>0.9999
CTP:PMA_6AN_15m vs. UDP:PMA_2DG_15m	ns	0.9097
CTP:PMA_6AN_15m vs. UDP:PMA_6AN_15m	ns	0.2313
CTP:PMA_6AN_15m vs. UDP:PMA_DPI_15m	ns	>0.9999
CTP:PMA_6AN_15m vs. UDP:PMA_AA_15m	ns	0.4686
CTP:PMA_6AN_15m vs. UDP:PMA_IAA_15m	ns	>0.9999
CTP:PMA_6AN_15m vs. UTP:PMA_15m	ns	0.9998

CTP:PMA_6AN_15m vs. UTP:PMA_2DG_15m	ns	>0.9999
CTP:PMA_6AN_15m vs. UTP:PMA_6AN_15m	ns	>0.9999
CTP:PMA_6AN_15m vs. UTP:PMA_DPI_15m	ns	>0.9999
CTP:PMA_6AN_15m vs. UTP:PMA_AA_15m	ns	>0.9999
CTP:PMA_6AN_15m vs. UTP:PMA_IAA_15m	*	0.044
CTP:PMA_DPI_15m vs. CTP:PMA_AA_15m	ns	0.5731
CTP:PMA_DPI_15m vs. CTP:PMA_IAA_15m	***	0.0004
CTP:PMA_DPI_15m vs. Uridine:PMA_15m	ns	>0.9999
CTP:PMA_DPI_15m vs. Uridine:PMA_2DG_15m	ns	0.357
CTP:PMA_DPI_15m vs. Uridine:PMA_6AN_15m	ns	0.1113
CTP:PMA_DPI_15m vs. Uridine:PMA_DPI_15m	ns	0.8675
CTP:PMA_DPI_15m vs. Uridine:PMA_AA_15m	ns	0.0727
CTP:PMA_DPI_15m vs. Uridine:PMA_IAA_15m	ns	>0.9999
CTP:PMA_DPI_15m vs. UMP:PMA_15m	ns	0.9177
CTP:PMA_DPI_15m vs. UMP:PMA_2DG_15m	*	0.043
CTP:PMA_DPI_15m vs. UMP:PMA_6AN_15m	**	0.0081
CTP:PMA_DPI_15m vs. UMP:PMA_DPI_15m	ns	0.2186
CTP:PMA_DPI_15m vs. UMP:PMA_AA_15m	**	0.0047
CTP:PMA_DPI_15m vs. UMP:PMA_IAA_15m	ns	>0.9999
CTP:PMA_DPI_15m vs. UDP:PMA_15m	ns	0.9256
CTP:PMA_DPI_15m vs. UDP:PMA_2DG_15m	*	0.046
CTP:PMA_DPI_15m vs. UDP:PMA_6AN_15m	**	0.0088
CTP:PMA_DPI_15m vs. UDP:PMA_DPI_15m	ns	0.2313
CTP:PMA_DPI_15m vs. UDP:PMA_AA_15m	**	0.0051
CTP:PMA_DPI_15m vs. UDP:PMA_IAA_15m	ns	>0.9999
CTP:PMA_DPI_15m vs. UTP:PMA_15m	ns	>0.9999
CTP:PMA_DPI_15m vs. UTP:PMA_2DG_15m	ns	>0.9999
CTP:PMA_DPI_15m vs. UTP:PMA_6AN_15m	ns	>0.9999
CTP:PMA_DPI_15m vs. UTP:PMA_DPI_15m	ns	>0.9999
CTP:PMA_DPI_15m vs. UTP:PMA_AA_15m	ns	>0.9999
CTP:PMA_DPI_15m vs. UTP:PMA_IAA_15m	ns	0.9014
CTP:PMA_AA_15m vs. CTP:PMA_IAA_15m	****	<0.0001
CTP:PMA_AA_15m vs. Uridine:PMA_15m	ns	>0.9999
CTP:PMA_AA_15m vs. Uridine:PMA_2DG_15m	ns	>0.9999
CTP:PMA_AA_15m vs. Uridine:PMA_6AN_15m	ns	0.995
CTP:PMA_AA_15m vs. Uridine:PMA_DPI_15m	ns	>0.9999
CTP:PMA_AA_15m vs. Uridine:PMA_AA_15m	ns	0.8675
CTP:PMA_AA_15m vs. Uridine:PMA_IAA_15m	ns	>0.9999
CTP:PMA_AA_15m vs. UMP:PMA_15m	ns	>0.9999
CTP:PMA_AA_15m vs. UMP:PMA_2DG_15m	ns	0.9524
CTP:PMA_AA_15m vs. UMP:PMA_6AN_15m	ns	0.6899
CTP:PMA_AA_15m vs. UMP:PMA_DPI_15m	ns	>0.9999
CTP:PMA_AA_15m vs. UMP:PMA_AA_15m	ns	0.2186
CTP:PMA_AA_15m vs. UMP:PMA_IAA_15m	ns	>0.9999
CTP:PMA_AA_15m vs. UDP:PMA_15m	ns	>0.9999
CTP:PMA_AA_15m vs. UDP:PMA_2DG_15m	ns	0.9577
CTP:PMA_AA_15m vs. UDP:PMA_6AN_15m	ns	0.706

CTP:PMA_AA_15m vs. UDP:PMA_DPI_15m	ns	>0.9999
CTP:PMA_AA_15m vs. UDP:PMA_AA_15m	ns	0.2313
CTP:PMA_AA_15m vs. UDP:PMA_IAA_15m	ns	>0.9999
CTP:PMA_AA_15m vs. UTP:PMA_15m	ns	0.9991
CTP:PMA_AA_15m vs. UTP:PMA_2DG_15m	ns	>0.9999
CTP:PMA_AA_15m vs. UTP:PMA_6AN_15m	ns	>0.9999
CTP:PMA_AA_15m vs. UTP:PMA_DPI_15m	ns	>0.9999
CTP:PMA_AA_15m vs. UTP:PMA_AA_15m	ns	>0.9999
CTP:PMA_AA_15m vs. UTP:PMA_IAA_15m	*	0.0272
CTP:PMA_IAA_15m vs. Uridine:PMA_15m	**	0.0091
CTP:PMA_IAA_15m vs. Uridine:PMA_2DG_15m	****	<0.0001
CTP:PMA_IAA_15m vs. Uridine:PMA_6AN_15m	****	<0.0001
CTP:PMA_IAA_15m vs. Uridine:PMA_DPI_15m	**	0.0012
CTP:PMA_IAA_15m vs. Uridine:PMA_AA_15m	****	<0.0001
CTP:PMA_IAA_15m vs. Uridine:PMA_IAA_15m	ns	0.8675
CTP:PMA_IAA_15m vs. UMP:PMA_15m	***	0.0004
CTP:PMA_IAA_15m vs. UMP:PMA_2DG_15m	****	<0.0001
CTP:PMA_IAA_15m vs. UMP:PMA_6AN_15m	****	<0.0001
CTP:PMA_IAA_15m vs. UMP:PMA_DPI_15m	****	<0.0001
CTP:PMA_IAA_15m vs. UMP:PMA_AA_15m	****	<0.0001
CTP:PMA_IAA_15m vs. UMP:PMA_IAA_15m	ns	0.2186
CTP:PMA_IAA_15m vs. UDP:PMA_15m	***	0.0004
CTP:PMA_IAA_15m vs. UDP:PMA_2DG_15m	****	<0.0001
CTP:PMA_IAA_15m vs. UDP:PMA_6AN_15m	****	<0.0001
CTP:PMA_IAA_15m vs. UDP:PMA_DPI_15m	****	<0.0001
CTP:PMA_IAA_15m vs. UDP:PMA_AA_15m	****	<0.0001
CTP:PMA_IAA_15m vs. UDP:PMA_IAA_15m	ns	0.2313
CTP:PMA_IAA_15m vs. UTP:PMA_15m	ns	>0.9999
CTP:PMA_IAA_15m vs. UTP:PMA_2DG_15m	ns	0.4336
CTP:PMA_IAA_15m vs. UTP:PMA_6AN_15m	ns	0.148
CTP:PMA_IAA_15m vs. UTP:PMA_DPI_15m	ns	0.9924
CTP:PMA_IAA_15m vs. UTP:PMA_AA_15m	ns	0.0989
CTP:PMA_IAA_15m vs. UTP:PMA_IAA_15m	ns	>0.9999
Uridine:PMA_15m vs. Uridine:PMA_2DG_15m	ns	0.7475
Uridine:PMA_15m vs. Uridine:PMA_6AN_15m	ns	0.1037
Uridine:PMA_15m vs. Uridine:PMA_DPI_15m	ns	>0.9999
Uridine:PMA_15m vs. Uridine:PMA_AA_15m	*	0.0409
Uridine:PMA_15m vs. Uridine:PMA_IAA_15m	*	0.0236
Uridine:PMA_15m vs. UMP:PMA_15m	ns	>0.9999
Uridine:PMA_15m vs. UMP:PMA_2DG_15m	ns	0.9994
Uridine:PMA_15m vs. UMP:PMA_6AN_15m	ns	0.9618
Uridine:PMA_15m vs. UMP:PMA_DPI_15m	ns	>0.9999
Uridine:PMA_15m vs. UMP:PMA_AA_15m	ns	0.9168
Uridine:PMA_15m vs. UMP:PMA_IAA_15m	ns	>0.9999
Uridine:PMA_15m vs. UDP:PMA_15m	ns	>0.9999
Uridine:PMA_15m vs. UDP:PMA_2DG_15m	ns	0.9995
Uridine:PMA_15m vs. UDP:PMA_6AN_15m	ns	0.9663

Uridine:PMA_15m vs. UDP:PMA_DPI_15m	ns	>0.9999
Uridine:PMA_15m vs. UDP:PMA_AA_15m	ns	0.9247
Uridine:PMA_15m vs. UDP:PMA_IAA_15m	ns	>0.9999
Uridine:PMA_15m vs. UTP:PMA_15m	ns	0.7128
Uridine:PMA_15m vs. UTP:PMA_2DG_15m	ns	>0.9999
Uridine:PMA_15m vs. UTP:PMA_6AN_15m	ns	>0.9999
Uridine:PMA_15m vs. UTP:PMA_DPI_15m	ns	0.9991
Uridine:PMA_15m vs. UTP:PMA_AA_15m	ns	>0.9999
Uridine:PMA_15m vs. UTP:PMA_IAA_15m	**	0.0042
Uridine:PMA_2DG_15m vs. Uridine:PMA_6AN_15m	ns	>0.9999
Uridine:PMA_2DG_15m vs. Uridine:PMA_DPI_15m	ns	>0.9999
Uridine:PMA_2DG_15m vs. Uridine:PMA_AA_15m	ns	>0.9999
Uridine:PMA_2DG_15m vs. Uridine:PMA_IAA_15m	****	<0.0001
Uridine:PMA_2DG_15m vs. UMP:PMA_15m	ns	>0.9999
Uridine:PMA_2DG_15m vs. UMP:PMA_2DG_15m	ns	>0.9999
Uridine:PMA_2DG_15m vs. UMP:PMA_6AN_15m	ns	>0.9999
Uridine:PMA_2DG_15m vs. UMP:PMA_DPI_15m	ns	>0.9999
Uridine:PMA_2DG_15m vs. UMP:PMA_AA_15m	ns	>0.9999
Uridine:PMA_2DG_15m vs. UMP:PMA_IAA_15m	ns	0.8699
Uridine:PMA_2DG_15m vs. UDP:PMA_15m	ns	>0.9999
Uridine:PMA_2DG_15m vs. UDP:PMA_2DG_15m	ns	>0.9999
Uridine:PMA_2DG_15m vs. UDP:PMA_6AN_15m	ns	>0.9999
Uridine:PMA_2DG_15m vs. UDP:PMA_DPI_15m	ns	>0.9999
Uridine:PMA_2DG_15m vs. UDP:PMA_AA_15m	ns	>0.9999
Uridine:PMA_2DG_15m vs. UDP:PMA_IAA_15m	ns	0.8588
Uridine:PMA_2DG_15m vs. UTP:PMA_15m	ns	0.0533
Uridine:PMA_2DG_15m vs. UTP:PMA_2DG_15m	ns	0.7128
Uridine:PMA_2DG_15m vs. UTP:PMA_6AN_15m	ns	0.9986
Uridine:PMA_2DG_15m vs. UTP:PMA_DPI_15m	ns	0.227
Uridine:PMA_2DG_15m vs. UTP:PMA_AA_15m	ns	0.9997
Uridine:PMA_2DG_15m vs. UTP:PMA_IAA_15m	****	<0.0001
Uridine:PMA_6AN_15m vs. Uridine:PMA_DPI_15m	ns	0.8059
Uridine:PMA_6AN_15m vs. Uridine:PMA_AA_15m	ns	>0.9999
Uridine:PMA_6AN_15m vs. Uridine:PMA_IAA_15m	****	<0.0001
Uridine:PMA_6AN_15m vs. UMP:PMA_15m	ns	>0.9999
Uridine:PMA_6AN_15m vs. UMP:PMA_2DG_15m	ns	>0.9999
Uridine:PMA_6AN_15m vs. UMP:PMA_6AN_15m	ns	>0.9999
Uridine:PMA_6AN_15m vs. UMP:PMA_DPI_15m	ns	>0.9999
Uridine:PMA_6AN_15m vs. UMP:PMA_AA_15m	ns	>0.9999
Uridine:PMA_6AN_15m vs. UMP:PMA_IAA_15m	ns	0.5197
Uridine:PMA_6AN_15m vs. UDP:PMA_15m	ns	>0.9999
Uridine:PMA_6AN_15m vs. UDP:PMA_2DG_15m	ns	>0.9999
Uridine:PMA_6AN_15m vs. UDP:PMA_6AN_15m	ns	>0.9999
Uridine:PMA_6AN_15m vs. UDP:PMA_DPI_15m	ns	>0.9999
Uridine:PMA_6AN_15m vs. UDP:PMA_AA_15m	ns	>0.9999
Uridine:PMA_6AN_15m vs. UDP:PMA_IAA_15m	ns	0.5027
Uridine:PMA_6AN_15m vs. UTP:PMA_15m	*	0.0104

Uridine:PMA_6AN_15m vs. UTP:PMA_2DG_15m	ns	0.6592
Uridine:PMA_6AN_15m vs. UTP:PMA_6AN_15m	ns	0.7128
Uridine:PMA_6AN_15m vs. UTP:PMA_DPI_15m	ns	0.0605
Uridine:PMA_6AN_15m vs. UTP:PMA_AA_15m	ns	0.9753
Uridine:PMA_6AN_15m vs. UTP:PMA_IAA_15m	****	<0.0001
Uridine:PMA_DPI_15m vs. Uridine:PMA_AA_15m	ns	0.5731
Uridine:PMA_DPI_15m vs. Uridine:PMA_IAA_15m	***	0.0004
Uridine:PMA_DPI_15m vs. UMP:PMA_15m	ns	>0.9999
Uridine:PMA_DPI_15m vs. UMP:PMA_2DG_15m	ns	>0.9999
Uridine:PMA_DPI_15m vs. UMP:PMA_6AN_15m	ns	0.9996
Uridine:PMA_DPI_15m vs. UMP:PMA_DPI_15m	ns	>0.9999
Uridine:PMA_DPI_15m vs. UMP:PMA_AA_15m	ns	0.9981
Uridine:PMA_DPI_15m vs. UMP:PMA_IAA_15m	ns	>0.9999
Uridine:PMA_DPI_15m vs. UDP:PMA_15m	ns	>0.9999
Uridine:PMA_DPI_15m vs. UDP:PMA_2DG_15m	ns	>0.9999
Uridine:PMA_DPI_15m vs. UDP:PMA_6AN_15m	ns	0.9997
Uridine:PMA_DPI_15m vs. UDP:PMA_DPI_15m	ns	>0.9999
Uridine:PMA_DPI_15m vs. UDP:PMA_AA_15m	ns	0.9984
Uridine:PMA_DPI_15m vs. UDP:PMA_IAA_15m	ns	>0.9999
Uridine:PMA_DPI_15m vs. UTP:PMA_15m	ns	0.6263
Uridine:PMA_DPI_15m vs. UTP:PMA_2DG_15m	ns	>0.9999
Uridine:PMA_DPI_15m vs. UTP:PMA_6AN_15m	ns	>0.9999
Uridine:PMA_DPI_15m vs. UTP:PMA_DPI_15m	ns	0.7128
Uridine:PMA_DPI_15m vs. UTP:PMA_AA_15m	ns	>0.9999
Uridine:PMA_DPI_15m vs. UTP:PMA_IAA_15m	***	0.0005
Uridine:PMA_AA_15m vs. Uridine:PMA_IAA_15m	****	<0.0001
Uridine:PMA_AA_15m vs. UMP:PMA_15m	ns	>0.9999
Uridine:PMA_AA_15m vs. UMP:PMA_2DG_15m	ns	>0.9999
Uridine:PMA_AA_15m vs. UMP:PMA_6AN_15m	ns	>0.9999
Uridine:PMA_AA_15m vs. UMP:PMA_DPI_15m	ns	>0.9999
Uridine:PMA_AA_15m vs. UMP:PMA_AA_15m	ns	>0.9999
Uridine:PMA_AA_15m vs. UMP:PMA_IAA_15m	ns	0.404
Uridine:PMA_AA_15m vs. UDP:PMA_15m	ns	>0.9999
Uridine:PMA_AA_15m vs. UDP:PMA_2DG_15m	ns	>0.9999
Uridine:PMA_AA_15m vs. UDP:PMA_6AN_15m	ns	>0.9999
Uridine:PMA_AA_15m vs. UDP:PMA_DPI_15m	ns	>0.9999
Uridine:PMA_AA_15m vs. UDP:PMA_AA_15m	ns	>0.9999
Uridine:PMA_AA_15m vs. UDP:PMA_IAA_15m	ns	0.3884
Uridine:PMA_AA_15m vs. UTP:PMA_15m	**	0.006
Uridine:PMA_AA_15m vs. UTP:PMA_2DG_15m	ns	0.5393
Uridine:PMA_AA_15m vs. UTP:PMA_6AN_15m	ns	0.8829
Uridine:PMA_AA_15m vs. UTP:PMA_DPI_15m	*	0.038
Uridine:PMA_AA_15m vs. UTP:PMA_AA_15m	ns	0.7128
Uridine:PMA_AA_15m vs. UTP:PMA_IAA_15m	****	<0.0001
Uridine:PMA_IAA_15m vs. UMP:PMA_15m	ns	0.8692
Uridine:PMA_IAA_15m vs. UMP:PMA_2DG_15m	*	0.031
Uridine:PMA_IAA_15m vs. UMP:PMA_6AN_15m	**	0.0056

Uridine:PMA_IAA_15m vs. UMP:PMA_DPI_15m	ns	0.4857
Uridine:PMA_IAA_15m vs. UMP:PMA_AA_15m	**	0.0032
Uridine:PMA_IAA_15m vs. UMP:PMA_IAA_15m	ns	>0.9999
Uridine:PMA_IAA_15m vs. UDP:PMA_15m	ns	0.8798
Uridine:PMA_IAA_15m vs. UDP:PMA_2DG_15m	*	0.0332
Uridine:PMA_IAA_15m vs. UDP:PMA_6AN_15m	**	0.0061
Uridine:PMA_IAA_15m vs. UDP:PMA_DPI_15m	ns	0.5025
Uridine:PMA_IAA_15m vs. UDP:PMA_AA_15m	**	0.0035
Uridine:PMA_IAA_15m vs. UDP:PMA_IAA_15m	ns	>0.9999
Uridine:PMA_IAA_15m vs. UTP:PMA_15m	ns	>0.9999
Uridine:PMA_IAA_15m vs. UTP:PMA_2DG_15m	ns	>0.9999
Uridine:PMA_IAA_15m vs. UTP:PMA_6AN_15m	ns	>0.9999
Uridine:PMA_IAA_15m vs. UTP:PMA_DPI_15m	ns	>0.9999
Uridine:PMA_IAA_15m vs. UTP:PMA_AA_15m	ns	>0.9999
Uridine:PMA_IAA_15m vs. UTP:PMA_IAA_15m	ns	0.7128
UMP:PMA_15m vs. UMP:PMA_2DG_15m	ns	0.7475
UMP:PMA_15m vs. UMP:PMA_6AN_15m	ns	0.1037
UMP:PMA_15m vs. UMP:PMA_DPI_15m	ns	>0.9999
UMP:PMA_15m vs. UMP:PMA_AA_15m	*	0.0409
UMP:PMA_15m vs. UMP:PMA_IAA_15m	*	0.0236
UMP:PMA_15m vs. UDP:PMA_15m	ns	>0.9999
UMP:PMA_15m vs. UDP:PMA_2DG_15m	ns	>0.9999
UMP:PMA_15m vs. UDP:PMA_6AN_15m	ns	>0.9999
UMP:PMA_15m vs. UDP:PMA_DPI_15m	ns	>0.9999
UMP:PMA_15m vs. UDP:PMA_AA_15m	ns	>0.9999
UMP:PMA_15m vs. UDP:PMA_IAA_15m	ns	0.9996
UMP:PMA_15m vs. UTP:PMA_15m	ns	0.1186
UMP:PMA_15m vs. UTP:PMA_2DG_15m	ns	>0.9999
UMP:PMA_15m vs. UTP:PMA_6AN_15m	ns	>0.9999
UMP:PMA_15m vs. UTP:PMA_DPI_15m	ns	0.8108
UMP:PMA_15m vs. UTP:PMA_AA_15m	ns	>0.9999
UMP:PMA_15m vs. UTP:PMA_IAA_15m	***	0.0002
UMP:PMA_2DG_15m vs. UMP:PMA_6AN_15m	ns	>0.9999
UMP:PMA_2DG_15m vs. UMP:PMA_DPI_15m	ns	>0.9999
UMP:PMA_2DG_15m vs. UMP:PMA_AA_15m	ns	>0.9999
UMP:PMA_2DG_15m vs. UMP:PMA_IAA_15m	****	<0.0001
UMP:PMA_2DG_15m vs. UDP:PMA_15m	ns	>0.9999
UMP:PMA_2DG_15m vs. UDP:PMA_2DG_15m	ns	>0.9999
UMP:PMA_2DG_15m vs. UDP:PMA_6AN_15m	ns	>0.9999
UMP:PMA_2DG_15m vs. UDP:PMA_DPI_15m	ns	>0.9999
UMP:PMA_2DG_15m vs. UDP:PMA_AA_15m	ns	>0.9999
UMP:PMA_2DG_15m vs. UDP:PMA_IAA_15m	ns	0.2745
UMP:PMA_2DG_15m vs. UTP:PMA_15m	**	0.0032
UMP:PMA_2DG_15m vs. UTP:PMA_2DG_15m	ns	0.1186
UMP:PMA_2DG_15m vs. UTP:PMA_6AN_15m	ns	0.7839
UMP:PMA_2DG_15m vs. UTP:PMA_DPI_15m	*	0.0216
UMP:PMA_2DG_15m vs. UTP:PMA_AA_15m	ns	0.8721

UMP:PMA_2DG_15m vs. UTP:PMA_IAA_15m	****	<0.0001
UMP:PMA_6AN_15m vs. UMP:PMA_DPI_15m	ns	0.8059
UMP:PMA_6AN_15m vs. UMP:PMA_AA_15m	ns	>0.9999
UMP:PMA_6AN_15m vs. UMP:PMA_IAA_15m	****	<0.0001
UMP:PMA_6AN_15m vs. UDP:PMA_15m	ns	>0.9999
UMP:PMA_6AN_15m vs. UDP:PMA_2DG_15m	ns	>0.9999
UMP:PMA_6AN_15m vs. UDP:PMA_6AN_15m	ns	>0.9999
UMP:PMA_6AN_15m vs. UDP:PMA_DPI_15m	ns	>0.9999
UMP:PMA_6AN_15m vs. UDP:PMA_AA_15m	ns	>0.9999
UMP:PMA_6AN_15m vs. UDP:PMA_IAA_15m	ns	0.0781
UMP:PMA_6AN_15m vs. UTP:PMA_15m	***	0.0005
UMP:PMA_6AN_15m vs. UTP:PMA_2DG_15m	ns	0.1335
UMP:PMA_6AN_15m vs. UTP:PMA_6AN_15m	ns	0.1186
UMP:PMA_6AN_15m vs. UTP:PMA_DPI_15m	**	0.0037
UMP:PMA_6AN_15m vs. UTP:PMA_AA_15m	ns	0.5217
UMP:PMA_6AN_15m vs. UTP:PMA_IAA_15m	****	<0.0001
UMP:PMA_DPI_15m vs. UMP:PMA_AA_15m	ns	0.5731
UMP:PMA_DPI_15m vs. UMP:PMA_IAA_15m	***	0.0004
UMP:PMA_DPI_15m vs. UDP:PMA_15m	ns	>0.9999
UMP:PMA_DPI_15m vs. UDP:PMA_2DG_15m	ns	>0.9999
UMP:PMA_DPI_15m vs. UDP:PMA_6AN_15m	ns	>0.9999
UMP:PMA_DPI_15m vs. UDP:PMA_DPI_15m	ns	>0.9999
UMP:PMA_DPI_15m vs. UDP:PMA_AA_15m	ns	>0.9999
UMP:PMA_DPI_15m vs. UDP:PMA_IAA_15m	ns	0.9622
UMP:PMA_DPI_15m vs. UTP:PMA_15m	ns	0.1194
UMP:PMA_DPI_15m vs. UTP:PMA_2DG_15m	ns	0.9901
UMP:PMA_DPI_15m vs. UTP:PMA_6AN_15m	ns	>0.9999
UMP:PMA_DPI_15m vs. UTP:PMA_DPI_15m	ns	0.1186
UMP:PMA_DPI_15m vs. UTP:PMA_AA_15m	ns	>0.9999
UMP:PMA_DPI_15m vs. UTP:PMA_IAA_15m	****	<0.0001
UMP:PMA_AA_15m vs. UMP:PMA_IAA_15m	****	<0.0001
UMP:PMA_AA_15m vs. UDP:PMA_15m	ns	>0.9999
UMP:PMA_AA_15m vs. UDP:PMA_2DG_15m	ns	>0.9999
UMP:PMA_AA_15m vs. UDP:PMA_6AN_15m	ns	>0.9999
UMP:PMA_AA_15m vs. UDP:PMA_DPI_15m	ns	>0.9999
UMP:PMA_AA_15m vs. UDP:PMA_AA_15m	ns	>0.9999
UMP:PMA_AA_15m vs. UDP:PMA_IAA_15m	*	0.0499
UMP:PMA_AA_15m vs. UTP:PMA_15m	***	0.0002
UMP:PMA_AA_15m vs. UTP:PMA_2DG_15m	ns	0.0883
UMP:PMA_AA_15m vs. UTP:PMA_6AN_15m	ns	0.302
UMP:PMA_AA_15m vs. UTP:PMA_DPI_15m	**	0.0021
UMP:PMA_AA_15m vs. UTP:PMA_AA_15m	ns	0.1186
UMP:PMA_AA_15m vs. UTP:PMA_IAA_15m	****	<0.0001
UMP:PMA_IAA_15m vs. UDP:PMA_15m	ns	0.9998
UMP:PMA_IAA_15m vs. UDP:PMA_2DG_15m	ns	0.3006
UMP:PMA_IAA_15m vs. UDP:PMA_6AN_15m	ns	0.0882
UMP:PMA_IAA_15m vs. UDP:PMA_DPI_15m	ns	0.9707

UMP:PMA_IAA_15m vs. UDP:PMA_AA_15m	ns	0.0568
UMP:PMA_IAA_15m vs. UDP:PMA_IAA_15m	ns	>0.9999
UMP:PMA_IAA_15m vs. UTP:PMA_15m	ns	>0.9999
UMP:PMA_IAA_15m vs. UTP:PMA_2DG_15m	ns	>0.9999
UMP:PMA_IAA_15m vs. UTP:PMA_6AN_15m	ns	>0.9999
UMP:PMA_IAA_15m vs. UTP:PMA_DPI_15m	ns	>0.9999
UMP:PMA_IAA_15m vs. UTP:PMA_AA_15m	ns	>0.9999
UMP:PMA_IAA_15m vs. UTP:PMA_IAA_15m	ns	0.1186
UDP:PMA_15m vs. UDP:PMA_2DG_15m	ns	0.7475
UDP:PMA_15m vs. UDP:PMA_6AN_15m	ns	0.1037
UDP:PMA_15m vs. UDP:PMA_DPI_15m	ns	>0.9999
UDP:PMA_15m vs. UDP:PMA_AA_15m	*	0.0409
UDP:PMA_15m vs. UDP:PMA_IAA_15m	*	0.0236
UDP:PMA_15m vs. UTP:PMA_15m	ns	0.1266
UDP:PMA_15m vs. UTP:PMA_2DG_15m	ns	>0.9999
UDP:PMA_15m vs. UTP:PMA_6AN_15m	ns	>0.9999
UDP:PMA_15m vs. UTP:PMA_DPI_15m	ns	0.8238
UDP:PMA_15m vs. UTP:PMA_AA_15m	ns	>0.9999
UDP:PMA_15m vs. UTP:PMA_IAA_15m	***	0.0002
UDP:PMA_2DG_15m vs. UDP:PMA_6AN_15m	ns	>0.9999
UDP:PMA_2DG_15m vs. UDP:PMA_DPI_15m	ns	>0.9999
UDP:PMA_2DG_15m vs. UDP:PMA_AA_15m	ns	>0.9999
UDP:PMA_2DG_15m vs. UDP:PMA_IAA_15m	****	<0.0001
UDP:PMA_2DG_15m vs. UTP:PMA_15m	**	0.0034
UDP:PMA_2DG_15m vs. UTP:PMA_2DG_15m	ns	0.1266
UDP:PMA_2DG_15m vs. UTP:PMA_6AN_15m	ns	0.7978
UDP:PMA_2DG_15m vs. UTP:PMA_DPI_15m	*	0.0232
UDP:PMA_2DG_15m vs. UTP:PMA_AA_15m	ns	0.8825
UDP:PMA_2DG_15m vs. UTP:PMA_IAA_15m	****	<0.0001
UDP:PMA_6AN_15m vs. UDP:PMA_DPI_15m	ns	0.8059
UDP:PMA_6AN_15m vs. UDP:PMA_AA_15m	ns	>0.9999
UDP:PMA_6AN_15m vs. UDP:PMA_IAA_15m	****	<0.0001
UDP:PMA_6AN_15m vs. UTP:PMA_15m	***	0.0005
UDP:PMA_6AN_15m vs. UTP:PMA_2DG_15m	ns	0.1412
UDP:PMA_6AN_15m vs. UTP:PMA_6AN_15m	ns	0.1266
UDP:PMA_6AN_15m vs. UTP:PMA_DPI_15m	**	0.004
UDP:PMA_6AN_15m vs. UTP:PMA_AA_15m	ns	0.5388
UDP:PMA_6AN_15m vs. UTP:PMA_IAA_15m	****	<0.0001
UDP:PMA_DPI_15m vs. UDP:PMA_AA_15m	ns	0.5731
UDP:PMA_DPI_15m vs. UDP:PMA_IAA_15m	***	0.0004
UDP:PMA_DPI_15m vs. UTP:PMA_15m	ns	0.1265
UDP:PMA_DPI_15m vs. UTP:PMA_2DG_15m	ns	0.9916
UDP:PMA_DPI_15m vs. UTP:PMA_6AN_15m	ns	>0.9999
UDP:PMA_DPI_15m vs. UTP:PMA_DPI_15m	ns	0.1266
UDP:PMA_DPI_15m vs. UTP:PMA_AA_15m	ns	>0.9999
UDP:PMA_DPI_15m vs. UTP:PMA_IAA_15m	****	<0.0001
UDP:PMA_AA_15m vs. UDP:PMA_IAA_15m	****	<0.0001

UDP:PMA_AA_15m vs. UTP:PMA_15m	***	0.0003
UDP:PMA_AA_15m vs. UTP:PMA_2DG_15m	ns	0.0938
UDP:PMA_AA_15m vs. UTP:PMA_6AN_15m	ns	0.3157
UDP:PMA_AA_15m vs. UTP:PMA_DPI_15m	**	0.0023
UDP:PMA_AA_15m vs. UTP:PMA_AA_15m	ns	0.1266
UDP:PMA_AA_15m vs. UTP:PMA_IAA_15m	****	<0.0001
UDP:PMA_IAA_15m vs. UTP:PMA_15m	ns	>0.9999
UDP:PMA_IAA_15m vs. UTP:PMA_2DG_15m	ns	>0.9999
UDP:PMA_IAA_15m vs. UTP:PMA_6AN_15m	ns	>0.9999
UDP:PMA_IAA_15m vs. UTP:PMA_DPI_15m	ns	>0.9999
UDP:PMA_IAA_15m vs. UTP:PMA_AA_15m	ns	>0.9999
UDP:PMA_IAA_15m vs. UTP:PMA_IAA_15m	ns	0.1266
UTP:PMA_15m vs. UTP:PMA_2DG_15m	ns	0.7475
UTP:PMA_15m vs. UTP:PMA_6AN_15m	ns	0.1037
UTP:PMA_15m vs. UTP:PMA_DPI_15m	ns	>0.9999
UTP:PMA_15m vs. UTP:PMA_AA_15m	*	0.0409
UTP:PMA_15m vs. UTP:PMA_IAA_15m	*	0.0236
UTP:PMA_2DG_15m vs. UTP:PMA_6AN_15m	ns	>0.9999
UTP:PMA_2DG_15m vs. UTP:PMA_DPI_15m	ns	>0.9999
UTP:PMA_2DG_15m vs. UTP:PMA_AA_15m	ns	>0.9999
UTP:PMA_2DG_15m vs. UTP:PMA_IAA_15m	****	<0.0001
UTP:PMA_6AN_15m vs. UTP:PMA_DPI_15m	ns	0.8059
UTP:PMA_6AN_15m vs. UTP:PMA_AA_15m	ns	>0.9999
UTP:PMA_6AN_15m vs. UTP:PMA_IAA_15m	****	<0.0001
UTP:PMA_DPI_15m vs. UTP:PMA_AA_15m	ns	0.5731
UTP:PMA_DPI_15m vs. UTP:PMA_IAA_15m	***	0.0004
UTP:PMA_AA_15m vs. UTP:PMA_IAA_15m	****	<0.0001
